# Design of Small Circularly Polarized Luminescence-Active Planar-Chiral Azaboroles

**DOI:** 10.1021/acs.orglett.6c03789

**Published:** 2026-09-30

**Authors:** Jakub Koudelka, Timothée Cadart, Ivana Císařová, Róbert Gyepes, Miroslav Štěpánek, Jan Fischer, Jhon Sebastian Oviedo Ortiz, Jeanne Crassous, Martin Kotora

**Affiliations:** † Department of Organic Chemistry, Faculty of Science, 37740 Charles University, Hlavova 8, 128 00 Praha 2, Czech Republic; ‡ Department of Inorganic Chemistry, Faculty of Science, Charles University, Hlavova 8, 128 00 Praha 2, Czech Republic; § Department of Chemistry, Faculty of Education, J. Selye University, Bratislavská 3322, 945 01 Komárno, Slovakia; ∥ Department of Physical and Macromolecular Chemistry, Faculty of Science, Charles University, Hlavova 8, 128 00 Prague 2, Czech Republic; ⊥ Department of Analytical Chemistry, Faculty of Science, Charles University, Hlavova 8, 128 00 Prague 2, Czech Republic; # 27079 University of Rennes, CNRS, ISCR-UMR 6226, Rennes 35000, France

## Abstract

We report an enantioselective approach toward new planar-chiral azaboroles bearing the [2.2]­paracyclophane unit and studies of their (chir)­optical properties. Fusing the planar-chiral scaffold with a B,N-heterocycle led to the formation of a set of three substances, all of which have interesting chiroptical properties (*g*_lum_ up to 4.7 × 10^–3^ and *B*_CPL_ up to 3.1 M^–1^ cm^–1^). **1a** exhibits superior fluorescence in the solid state unlike the rest of the series.

## Graphical abstract

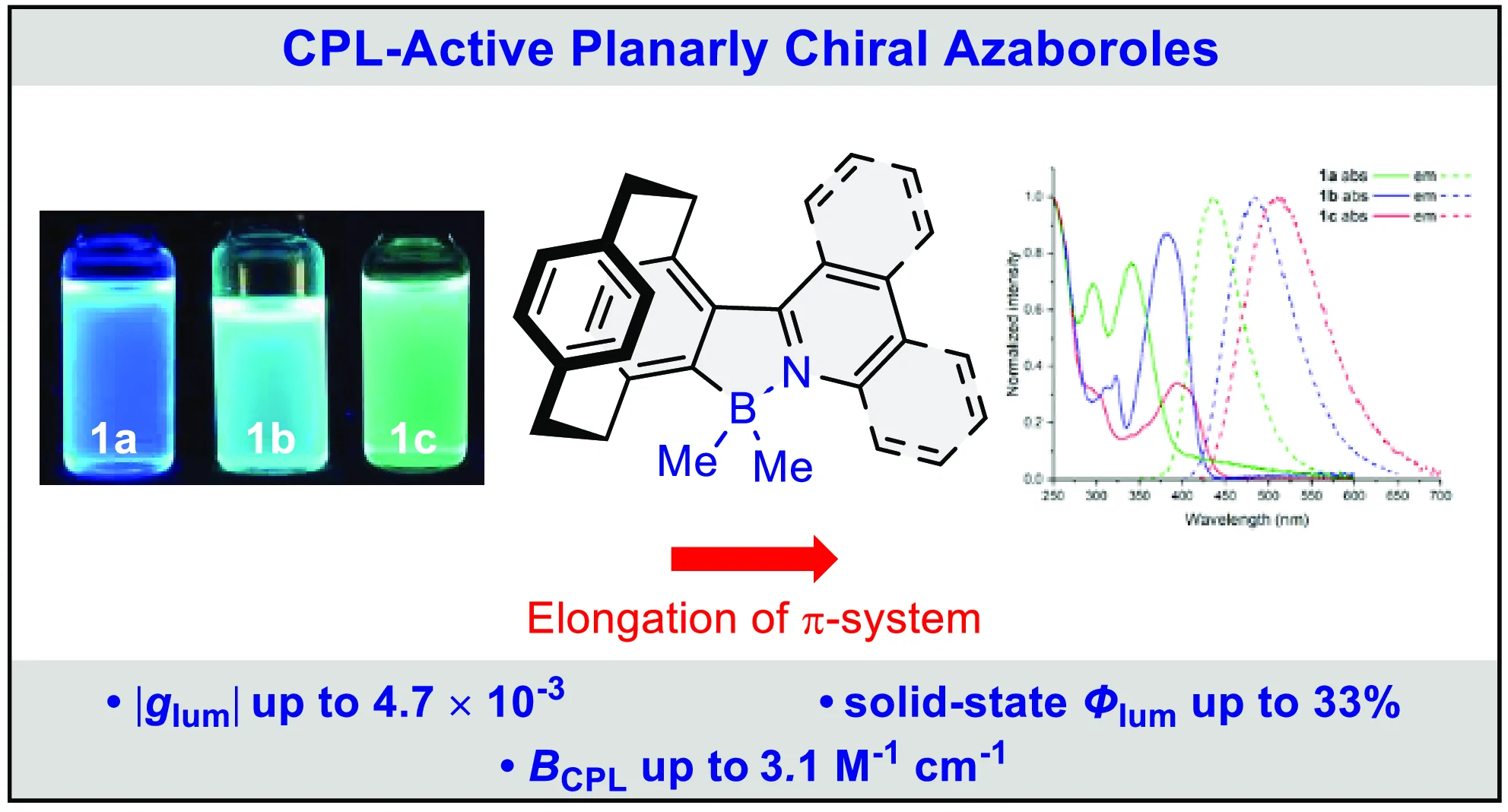

Three-dimensional (3D) polycyclic aromatic molecules exhibiting inherent chirality and studies of their physical properties have emerged as a pivotal concept in the field of material science over the past two decades.^1^ Such substances include various heteroaromatic compounds, distinguished by structural architectures and heteroatom doping, contributing to their unique chemicophysical properties (e.g., solid state packing, rigid frameworks, and chiroptical properties).^2^ Although substantial advances regarding chiroptical properties have been achieved for high-molecular weight substances, general attention has recently shifted to the design of small organic molecules, because of their facile structural modification and, often, easier manufacturing processes.^3^ Along those lines, considerable effort has been spent on compounds featuring an intramolecular B–N dative bond, the so-called azaboroles.^4^ Upon exchanging a C=C bond within the helical framework for an isoelectronic B–N counterpart, their molecular aromaticity remains preserved but allows for a fine-tuning of their electronic properties (high dissymmetric factors (*g*_lum_) and strong fluorescence emission).^5^

A typical example is a helical compound reported by the Nowak-Król group exhibiting a *g*_lum_ of up to 3.2 × 10^–3^ (Figure 1A).^5d^ Despite these promising results, their low configurational stability necessitates the exploration of alternative scaffolds.^5f^ In this respect, switching to a more rigid and configurationally stable scaffold, like the [2.2]­paracyclophane (pCp) unit,^6,7^ might offer a solution to such a problem as demonstrated by Morisaki et al. for efficient CPL emitters.^8^ Additionally, heteroaryl mono-pCp compounds with small π-conjugated systems have also been prepared, e.g., compounds possessing imidazole,^9^ lactone,^10^ and azaborine^11^ units with *g*_lum_ values of up to 5 × 10^–3^ (Figure 1B).

**Figure 1:**
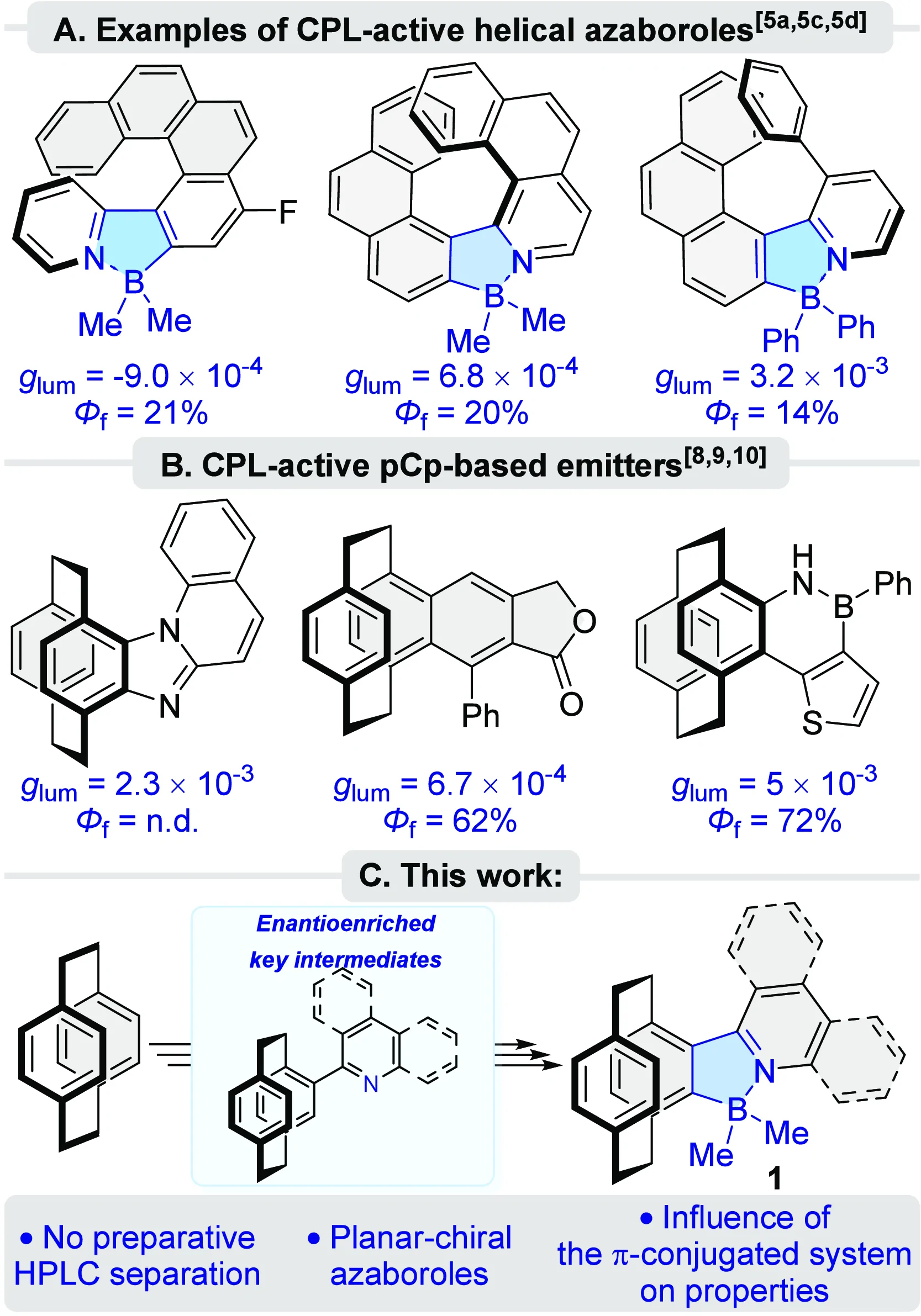
(A) Previously reported helical azaboroles, (B) pCp-containing conjugated heterocycles, and (C) newly designed pCp-based azaboroles **1**.

Interestingly, enantioenriched azaborole derivatives possessing fully carbon-based planar-chiral scaffolds have not been reported to the best of our knowledge. Therefore, we aimed to develop a simple route toward a series of novel polyaromatic azaboroles **1** by combining the pCp and the azaboracycle moieties and to study their (chir)­optical properties to pave the way for a new class of small 3D molecules (Figure 1C).

We anticipated that the crucial heteroaryl pCps depicted in Figure 1C could be synthesized by cross-coupling of a nucleophile prepared from pCp-bromide **5** and a suitable heteroaryl partner. Our initial efforts focused on the efficient preparation of enantioenriched pCp-bromides **5**, evaluating two distinct routes starting from pCp-carboxaldehyde **2** (Scheme 1, pathways A and B).

**Scheme 1:**
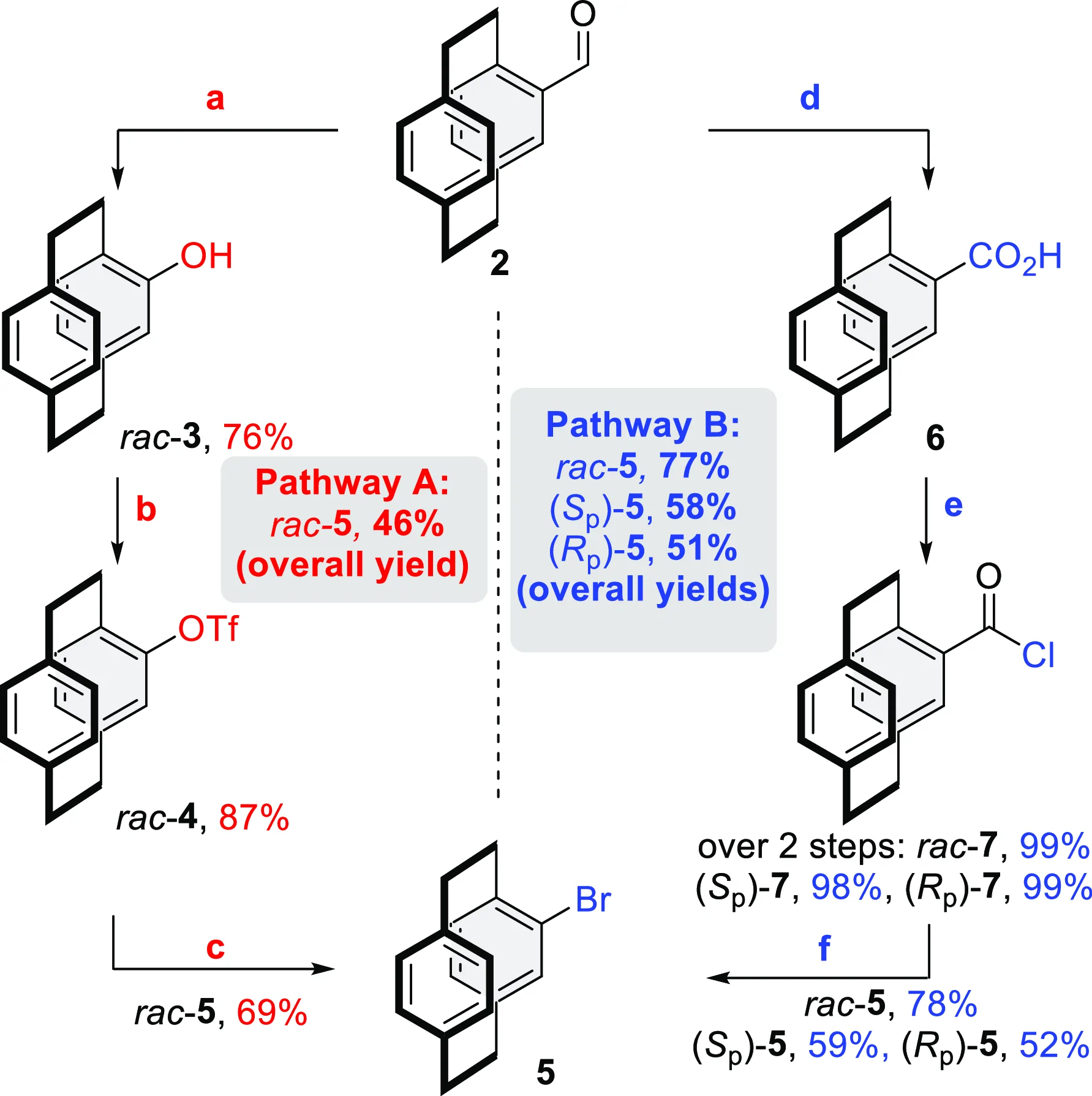
Two Synthetic Pathways from pCp-Aldehyde **2** to pCp-Bromide **5** ^a^

a Reaction conditions: (a) H_2_SO_4_, H_2_O_2_, MeOH/CH_2_Cl_2_, 25 °C; (b) Tf_2_O, Pyr, CH_2_Cl_2_, 0 °C; (c) KF, KBr, BrettPhosPdG3 (5 mol %), 1,4-dioxane, 130 °C, 16 h; (d) NaClO_2_, KH_2_PO_4_, DMSO, acetone/H_2_O, 25 °C; (e) SOCl_2_, 25 °C; (f) LiBr, [PdCl­(cinnamyl)]_2_ (2.5 mol %), Xantphos (12.5 mol %), toluene, 160 °C, 24 h.

Pathway A began with Dakin oxidation (H_2_SO_4_ and H_2_O_2_) of aldehyde **2** to alcohol **3** (76%) followed by triflation with Tf_2_O yielding pCp-triflate **4** (87%). Finally, application of a modified Buchwald’s protocol^12^ by using BrettPhosPdG3 catalyst furnished **5** in 69% yield (46% overall yield after three steps from **2**). With regard to pathway B, it started by a quantitative Pinnick oxidation^13^ of **2** to acid **6** followed by halogenation (SOCl_2_) to acyl chloride **7** (97% yield over two steps).

Then, decarbonylative bromination^14^ catalyzed by [PdCl­(cinnamyl)]_2_/Xanthphos afforded **5** in 78% isolated yield (76% yield over three steps from **2**). Subsequently, the enantiopurity of (*S*_p_)- and (*R*_p_)-**2** (ee ≥99%) was established via the kinetic resolution of *rac*-**2**,^15^ and the reaction sequence B^16^ allowed us to synthesize enantioenriched (*S*_p_)- and (*R*_p_)-**5** in 58% and 51% overall yields, respectively.

In the next step, pCp-bromides (*S*_p_)- and (*R*_p_)-**5** were converted into the corresponding zincate intermediates via *n*-BuLi/ZnBr_2_ exchange, which were directly used in Negishi cross-coupling with heteroaryl halides **8** (Scheme 2A).^17^ Gratifyingly, the cross-coupling reactions with 2-bromopyridine (**8a**) and 1-bromoisoquinoline (**8b**) catalyzed by PdCl_2_(dppf) in THF^18^ proceeded uneventfully, furnishing desired compounds (*S*_p_)- and (*R*_p_)-**9a** and (*S*_p_)- and (*R*_p_)-**9b** in 70%, 63%, 46%, and 34% yields, respectively.^19^ We assumed that a lower yield of **9b** might be caused by the steric bulkiness of both reactants, hindering the coupling process. In fact, the Negishi coupling failed to proceed when 6-chlorophenanthridine (**8c**) was employed. Alternatively, (*S*_p_)- and (*R*_p_)-**9c** were synthesized via condensations of (*S*_p_)- and (*R*_p_)-**2**, respectively, with 2-phenylaniline in TFA (140 °C, 4 days) in 44% yield (Scheme 2B).^20^

**Scheme 2:**
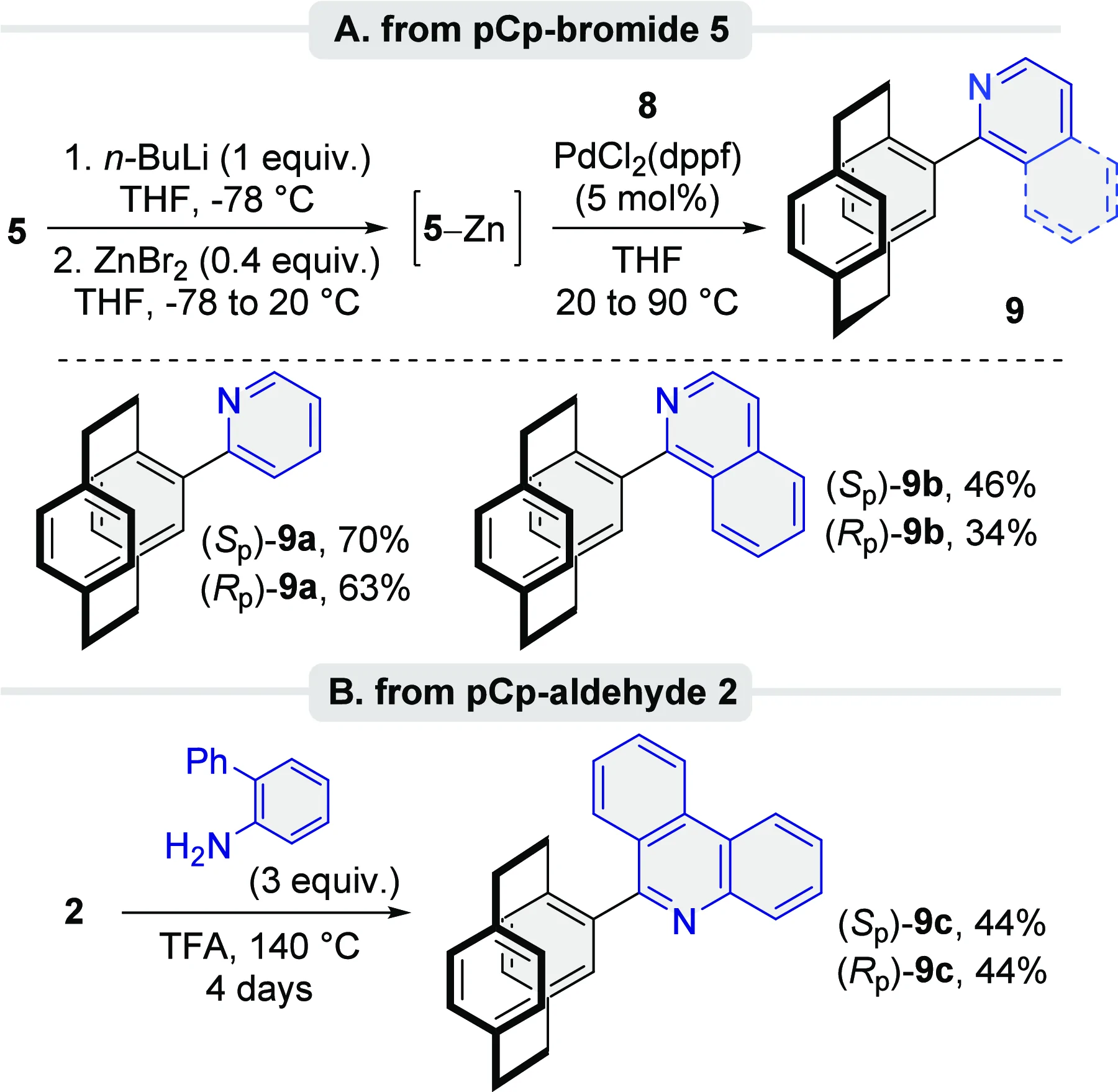
Synthesis of Enantioenriched (*S*_p_)- and (*R*_p_)-**9a–c**

Finally, the introduction of the boron atom into the scaffold was performed by intramolecular electrophilic borylation of **9** with BBr_3_, which was followed by methylation using Me_3_Al under the previously reported conditions,^21^ furnishing target azaboroles **1** (Scheme 3). It is worth mentioning that a better efficiency of electrophilic borylation was observed in chlorobenzene. Desired azaboroles (*S*_p_)- and (*R*_p_)-**1a–c** were obtained in yields of 36–53% after two steps and maintained high enantiopurity (>99% ee).

**Scheme 3:**
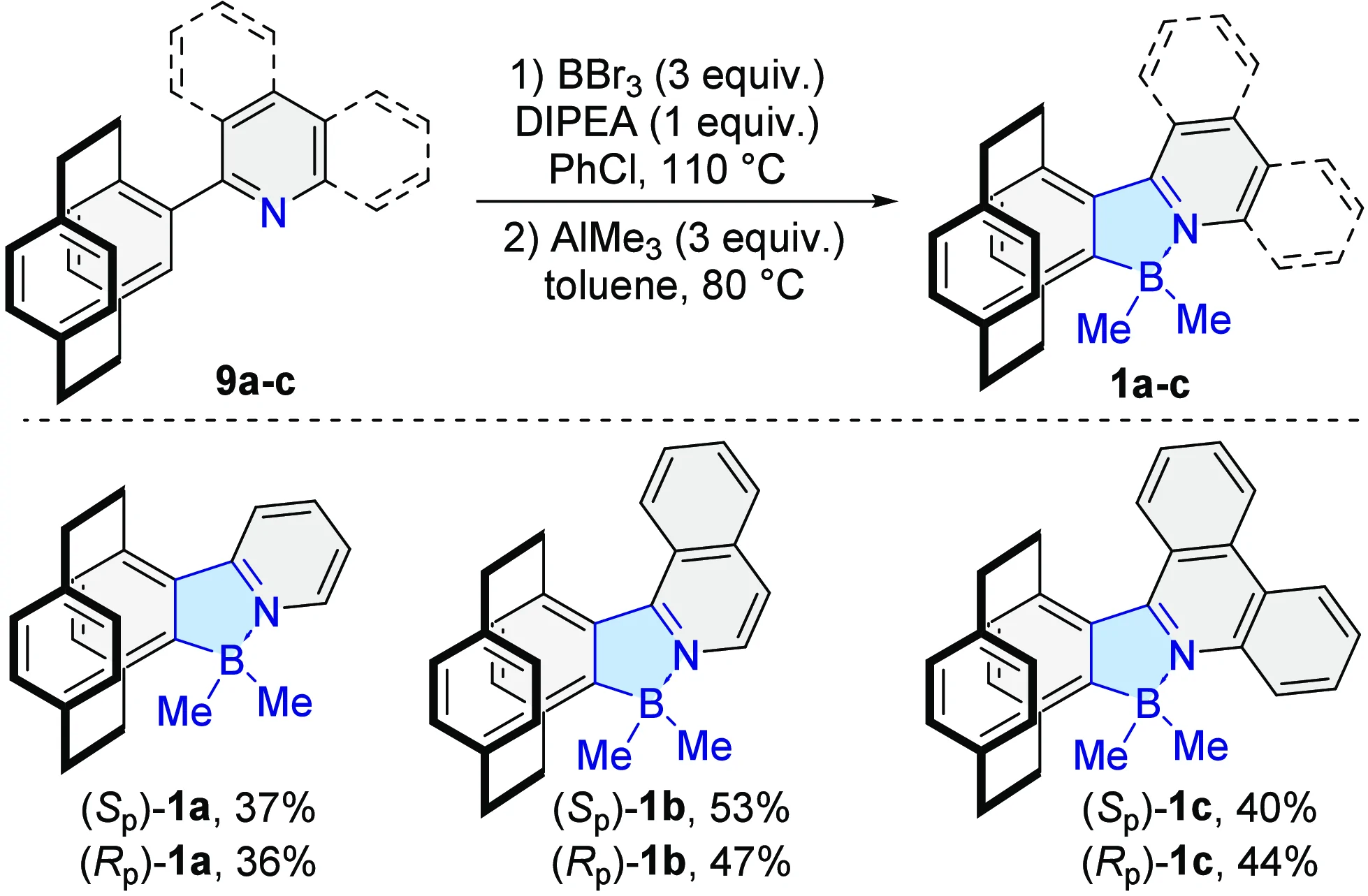
Synthesis of Planar-Chiral pCp-Azaboroles **1a–c**

Crystals suitable for single-crystal X-ray diffraction analysis were obtained by slow evaporation from either a DCM/hexane solution (**1a**) (Figure 2) or DCM solutions (**1b** and **1c**) (Figures S2 and S3). **1a** and **1c** crystallized in the *P*2_1_/*c* space group, whereas **1b** in *Pbca*. The B–N bond lengths in **1a–c** were 1.624(2), 1.619(2), and 1.642(1) Å, respectively, confirming strong Lewis-pair interactions and stable azaborole rings. The condensed aromatic ring frameworks adopt a slightly helical conformation in the solid state; the dihedral angle in **1a** for the inner helicene rim is 11.1(2)° [smaller than for the pCp-[3]­helicene (19.1°)].^22^ As the π-conjugated system is extended in **1b** and **1c**, the helicity increases, inducing a larger sum of two dihedral angles for **1b** and **1c** of 30.2(3)° and 30.6(2)°, respectively. The crystal packing of **1a** adopts a herringbone pattern, driven by T-shaped aromatic interactions. These “edge-to-face” interactions exhibit distances of 2.89–2.93 Å between the peripheral hydrogen atoms and the centroids of the neighboring aromatic rings, suggesting a robust network of C–H···π associations (Figure 2B). In contrast, π-extended systems in **1b** and **1c** display more prominent π–π stacking interactions reaching up to 3.47 Å in the case of **1c** (Figures S6 and S7).

**Figure 2:**
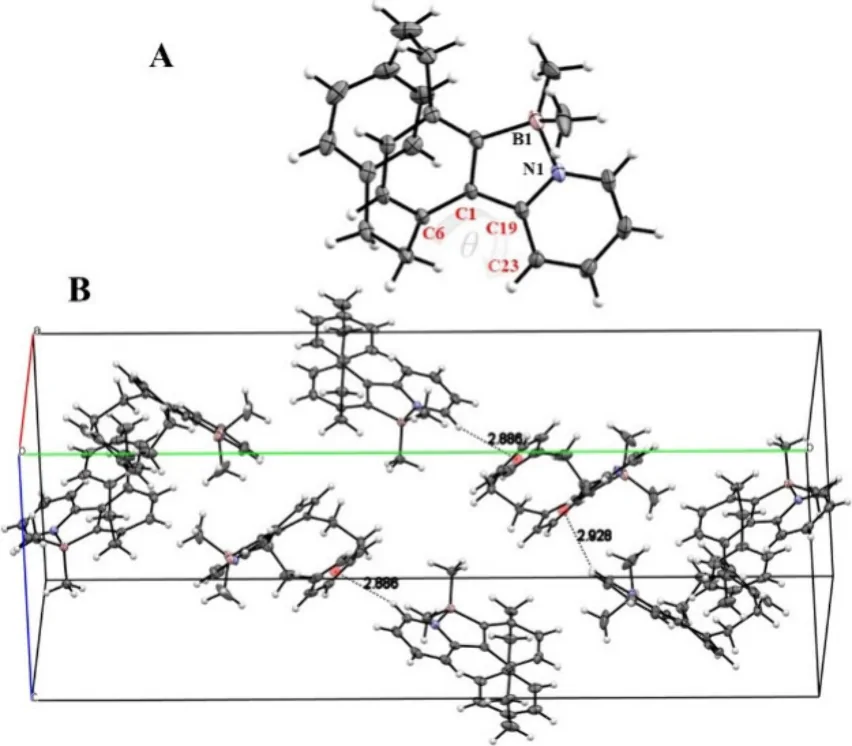
(A) Single-crystal X-ray diffraction structure of **1a** and (b) its packing pattern with C–H···π interactions. Heavy atoms are drawn with thermal ellipsoids at the 30% probability level.

DFT and TD-DFT computations were conducted to provide comprehensive insight into the influence of the azaborole ring on electronic properties of the aromatic conjugated systems in **1a–c** (Table S8). NICS(0) values indicated a low aromaticity of the azaborole rings (ring C) in all cases (1.17–1.65), while the computed values for ring A of the pCp scaffold (−8.57 to −8.67) corresponded to those reported for pristine pCp (ca. −9).^23^ For ring B, these values indicated a slightly lower aromaticity (−7.48 to −7.52) likely due to the nitrogen atom.

The photophysical properties of azaboroles **1a–c** recorded in CH_2_Cl_2_ solutions are summarized in Table 1. They show moderate molar absorption coefficients (*ε*) (8.4, 9.1, and 11.6 × 10^3^ M^–1^ cm^–1^, respectively) for the lowest-energy absorption bands at 340, 380, and 394 nm, respectively (Figure 3A). The fluorescence maxima are positioned at 437, 484, and 510 nm, respectively, with quantum yields (Φ_f_) of 4–17%. As expected, increasing the π-conjugated system resulted in a 73 nm bathochromic shift to the green-yellow region. It is worth mentioning that the emission maxima of **1c** in different solvents revealed a red-shifted solvatochromic effect from 479 nm (cyclohexane) to 518 nm (acetonitrile) (Figure S10), which indicated B–N dative bond stabilization in the excited state upon employing a more polar solvent. Interestingly, the measured solid state Φ_f_ of **1a** is twice as high (33%) as its value in the CH_2_Cl_2_ solution. With regard to **1b** and **1c**, solid state Φ_f_ measurements gave comparable results as in solutions (further details in section 5 of the Supporting Information). It should be emphasized that planar-chiral azaboroles **1** do not undergo aggregation-induced fluorescence quenching as previously reported for some tropos-helical azaboroles.^5d^ The higher fluorescence of **1a** can be attributed to its T-shaped packing in the solid state (Figure 2) coupled with the restriction of intramolecular rotation, which can significantly diminish nonradiative decay pathways leading to enhanced fluorescence.

**Figure 3:**
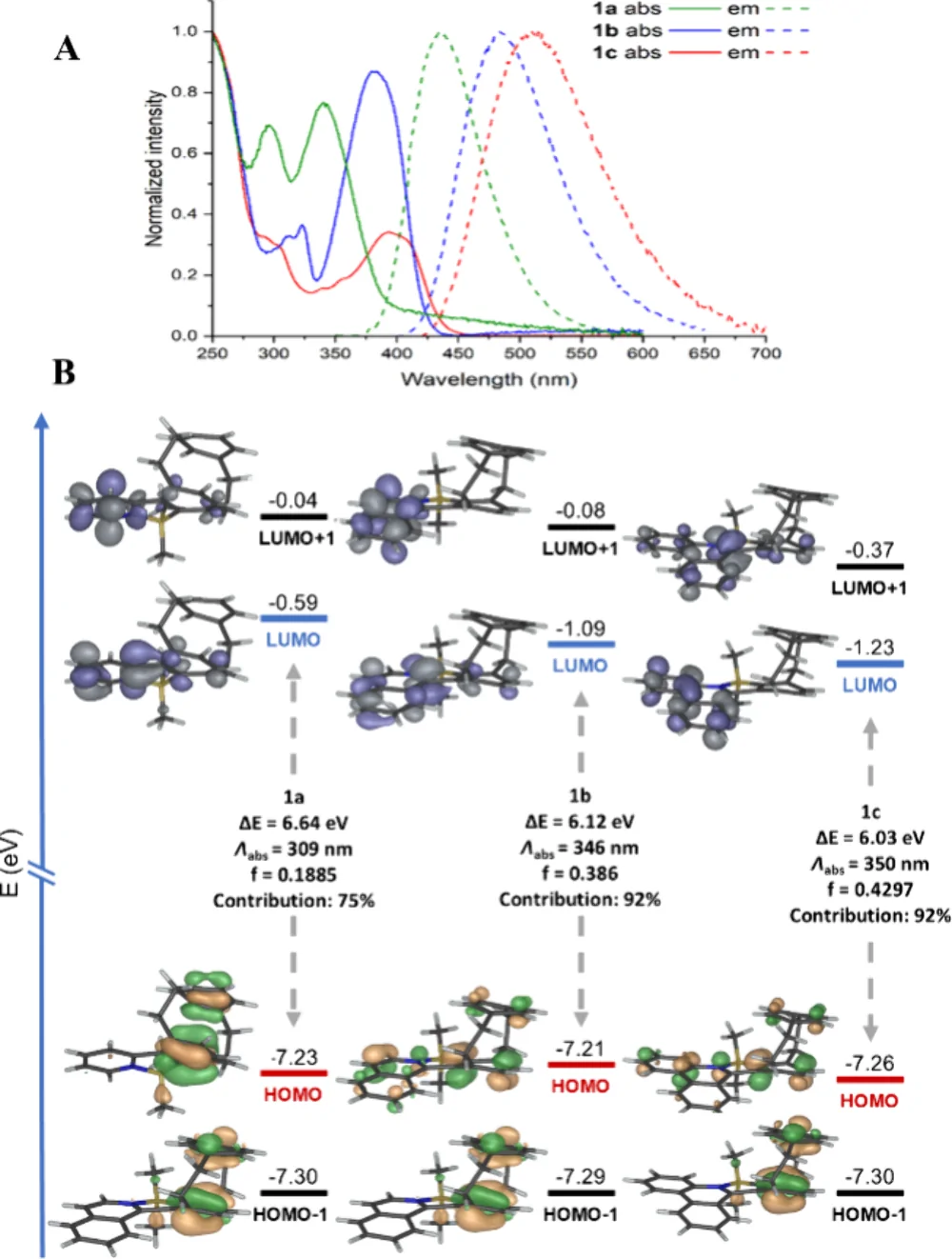
(A) Normalized absorbance and emission spectra of **1a–c** recorded in CH_2_Cl_2_ solutions. (B) TD-DFT calculations of the charge distribution of the HOMO–LUMO of **1a–c** (*f*, oscillator strength).

**Table 1:** Photophysical Properties of Compounds **1a–c**

	λ_abs_ (nm)^a^	λ_em_ (nm)^b^	Δν (cm^–1^)	τ_f_ (ns)^c^	$\frac{k_{nr}}{k_{r}}$ ^d^ ^,^ ^e^	Φ_f_ (%)^f^ ^,^ ^g^
**1a**	294, 340	437	6528	6.57 (97)	5.6	15 (33)
**1b**	380	484	5655	6.63 (100)	4.8	17 (17)
**1c**	394	510	5773	7.46 (94)	22.4	4 (4)

a Absorption maximum in the CH_2_Cl_2_ solution.

b Emission maximum in the CH_2_Cl_2_ solution.

c Fluorescence lifetime. Only the main decay component is given (fractional contribution in percent).

d Radiative decay constant (*k*_r_ = Φ_fl_/τ_fl_).

e Nonradiative decay constant (*k*_nr_ = (1 – Φ_fl_)/τ_fl_).

f Fluorescence quantum yield in the CH_2_Cl_2_ solution.

g Fluorescence quantum yields in the solid state are in parentheses.

For additional insight, the fluorescence lifetimes of **1a–c** were measured by time-correlated single-photon counting and are in the range of 6.57 (**1a**) to 7.46 ns (**1c**) (Table 1 and section 5.2 of the Supporting Information). Then, radiative (*k*_r_) and nonradiative (*k*_nr_) decay constants were calculated, and the *k*_nr_/*k*_r_ = (1 – Φ_fl_)/Φ_fl_ is comparable between **1a** (5.6) and **1b** (4.8). However, for **1c**, *k*_r_ is lower; therefore, it has a longer life and lower quantum yield. Furthermore, TD-DFT calculations at the M06-2X/def2-TZVP level of theory (including D3 dispersion and CH_2_Cl_2_ solvation effects) (Figure 3B and section 6 of the Supporting Information) indicate that the lowest-energy singlet electronic transition (S_0_ → S_1_) is characterized predominantly by the HOMO → LUMO transition (with contributions of 75–92%). Although this transition remains strongly favorable, the LUMO electron density in **1c** is fully localized on the phenanthrene moiety compared to **1a** and **1b**. Due to this shift, **1c** becomes highly polarized when it relaxes in the excited state. This assignment is supported by the experimentally observed red-shifted solvatochromism of **1c**.

The electrochemical properties were investigated in acetonitrile, evaluated versus ferrocenium/ferrocene (Fc^+^/Fc) (section 8 of the Supporting Information). The CV measurements display irreversible oxidation peaks in the narrow range of 0.69–0.74 V for all three compounds, suggesting that the HOMO energy level is localized on the common molecular backbone and becomes affected minimally by variations in the aromatic system. In contrast, the reduction processes show a clear dependence on the structure. **1a** exhibits a quasi-reversible reduction peak at −2.99 V. Extending the π-conjugation from **1a** to **1b** and **1c** results in a significant positive shift of the reduction potential to −2.50 and −2.36 V, respectively. This is in good accordance with data measured by DPV. Consequently, the calculated electrochemical band gap narrows progressively from 3.30 V (**1a**) to 2.81 V (**1c**), derived from CV onset potentials. This trend implies that the expansion of the fused aromatic system stabilizes primarily the LUMO energy level, increasing the electron deficiency of the compounds and making the reduction more feasible.

(*R*_p_)- and (*S*_p_)-**1a–c** display mirror images with positive and negative Cotton effects for the range of 230–450 nm in the CD profiles. The CD spectrum of (*R*_p_)-**1a** exhibits a strong positive Cotton effect at 259 nm (Δε = 82 M^–1^ cm^–1^) and a small negative one at 340 nm (Δε = −30 M^–1^ cm^–1^; |*g*_abs_| = 2.7 × 10^–3^) (Figure 4, left), while (*S*_p_)-**1a** CD spectrum remains in a mirror-image relationship. Data for **1b** and **1c** are available in section 5.3 of the Supporting Information. Strong signals of (*R*_p_)- and (*S*_p_)-**1a** appeared at 450 nm with a maximum CPL intensity (Δ*I*) of 0.005, and |*g*_lum_| values reached 4.7 × 10^–3^, surpassing the highest reported value for helical azaborole^5d^ (those for **1b** and **1c** were 1.3 and 1.6 × 10^–3^, respectively) but remaining within the typical range for pCp-based molecules.^24^ It is noteworthy that there is good agreement with DFT simulations of 4.20, 0.59, and 0.72 × 10^–3^, respectively (Table S7 and Figures S26–S28). Then, to quantify the chiroptical emission efficiency, the CPL brightness (*B*_CPL_ ^25^) values were calculated for **1a–c** (from 0.35 to 3.0 M^–1^ cm^–1^). Notably, a maximum value of 3.0 M^–1^ cm^–1^ was observed for **1a**, suggesting a robust translation of molecular chirality into the excited state, a significant value for such a small organoboron fluorophore.

**Figure 4:**
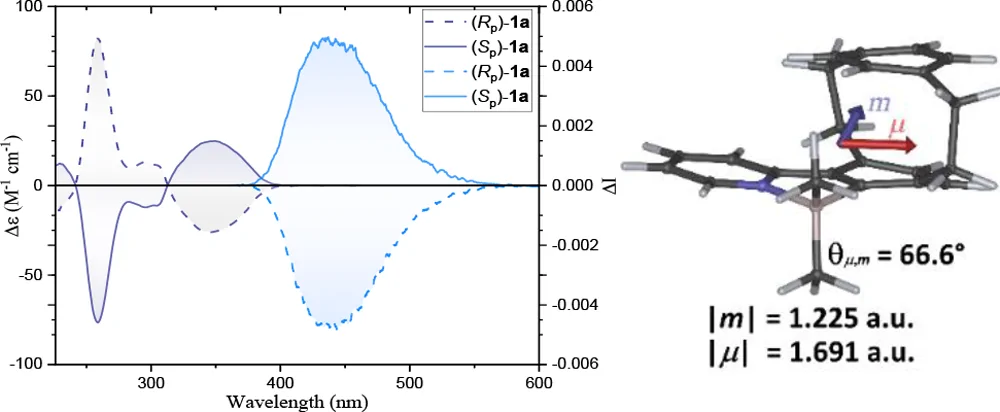
ECD (dark blue) and CPL (light blue) spectra (left) and transition dipole moments (right) of **1a** (CH_2_Cl_2_, rt). The electric (μ) and magnetic (*m*) transition dipole moments are shown as red and blue arrows, respectively. Vector lengths are scaled up for the sake of clarity.

In conclusion, we have developed an efficient method for synthesizing highly enantioenriched planar-chiral azaboroles featuring extended π-conjugated scaffolds. Combining a [2.2]­paracyclophane moiety and a B–N dative bond not only ensures configurational stability but also significantly enhances dipole moments. Additionally, it is worth mentioning that pyridine-based azaborole **1a** exhibits a significantly high |*g*_lum_| value of 4.7 × 10^–3^ (in solution) and a solid state Φ_f_ of 33%. Insights from single-crystal X-ray analyses reveal that the second phenyl ring from the pCp moiety prevents intermolecular aggregation, a critical factor for maintaining efficiency in solid state devices. Last but not least, taking into the account several key factors such as the (i) solid state Φ_f_, (ii) competitive |*g*_lum_| values, (iii) *B*_CPL_, and (iv) molecular weight, we believe that such small planar-chiral organic azaboroles may represent a promising class of materials for next-generation chiroptical applications.

## Supplementary Material



## Data Availability

The data underlying the study are available in the published article and its Supporting Information.

## Notes

Experimental details, analytical data, crystallographic data, NMR spectra, HPLC data, and computational details (PDF)
