# Supplementary material for: Design of Small Circularly Polarized Luminescence-Active Planar-Chiral Azaboroles
Source: Org Lett. 2026 Sep 30;28(41):12953–8. doi: 10.1021/acs.orglett.6c03789 (PMC13682213; doi:10.1021/acs.orglett.6c03789)
Supplement: Supplementary file 1 [file ol6c03789_si_001.pdf]

Supporting Information

for

Design of Small Circularly Polarized Luminescence-Active Planar-Chiral Azaboroles

Jakub Koudelka,[a] Timothée Cadart,*,[a] Ivana Císařová,[b] Róbert Gyepes,[c] Miroslav Štěpánek,[d] Jan Fischer,[e] Jhon Sebastian Oviedo Ortiz,[f] Jeanne Crassous,[f] and Martin Kotora*,[a]

[a] Department of Organic Chemistry, Faculty of Science, Charles University, Hlavova 8, 128 00 Prague 2, Czech Republic

[b] Department of Inorganic Chemistry, Faculty of Science, Charles University, Hlavova 8, 128 00 Prague 2, Czech Republic

[c] Department of Chemistry, Faculty of Education, J. Selye University, Bratislavská 3322, 945 01 Komárno, Slovakia

[d] Department of Physical and Macromolecular Chemistry, Faculty of Science, Charles University, Hlavova 8, 128 00 Prague 2, Czech Republic

[e] Department of Analytical Chemistry, Faculty of Science, Charles University, Hlavova 8, 128 00 Prague 2, Czech Republic

[f] University of Rennes, CNRS, ISCR−UMR 6226, 35000 Rennes, France

Contents

1 General information 3

2 Optimization of reaction conditions 5

2.1 Screening of conditions for the condensation reaction 5

2.2 Screening of solvent for the borylation reaction 6

3 Synthetic procedures 7

3.1 Synthetic procedures of starting compounds 7

3.2 Synthesis of 9 16

3.3 Synthesis of azabora-[2.2]paracyclophanes 1 22

4 Crystallographic data 30

5 Photophysical properties 35

5.1 Absorption and emission 35

5.2 Fluorescence decay measurements 40

5.3 Chiroptical properties 42

6 HOMO-LUMO 52

7 NICS 55

8 Electrochemistry 56

9 Optimized xyz coordinates 59

10 Copies of ^{1}H NMR and ^{13}C NMR spectra 69

1 General information

All commercially available reagents were purchased from Sigma-Aldrich, BLD Pharmatech, Acros Organics, Fluorochem, Alfa Aesar, TCI Chemicals, and Strem Chemicals. Solvents were purified and dried by distillation: tetrahydrofuran (THF) and toluene from sodium/benzophenone, dichloromethane and 1,2-dichloroethane from calcium hydride. Other solvents and reagents were used without further purification. All reactions were performed under inert atmosphere by using a vacuum/argon flushing system, unless it is mentioned. Column chromatography was performed on Silica gel 60A (40-60 µm) from SiliCycle by using a compact automated preparative device from ECOM (Czech Republic). Thin layer chromatography was performed on SiliCycle silica gel 60 F_{254} pre-coated aluminum sheets.

NMR spectra were recorded on Bruker AVANCE III Spectrometer (^{1}H at 400 MHz, ^{11}B at 128 MHz, ^{13}C at 101 MHz, and ^{19}F at 376 MHz). All NMR spectra were measured as solutions in $CDCl_3$ or/and C_6D_6 and referenced to residual solvent signals: $CDCl_3$ (^{1}H, δ_H = 7.26 ppm; ^{13}C, δ_C = 77.16 ppm) and C_6D_6 (^{1}H, δ_H = 7.16 ppm). Coupling constants J are given in Hz. Signals corresponding to the carbon atoms bound to the boron atom are not visible. The observation corresponds to the previously reported one (Full, J.; Panchal, S. P.; Götz, J.; Krause, A.-M.; Nowak-Król, A. *Angew. Chem. Int. Ed.* **2021**, *60*, 4350-4357).

Infrared spectra were recorded with a Nicolet Avatar 370 FTIR spectrophotometer. The method used for measuring was Attenuated Total Reflectance (ATR) with a Ge crystal. IR absorptions are given in wavenumbers as cm^{-1}.

MS spectra were recorded on an Agilent Technologies 6530 Accurate-Mass Q-TOF LC/MS spectrometer. Samples were ionized by electrospray ionization (ESI) or atmospheric pressure chemical ionization (APCI) and detected by quadrupole or TOF. Drying and nebulizer gas was nitrogen.

Melting points are uncorrected and were determined on a Büchi B-545 device.

HPLC analyses were performed with a Shimadzu liquid chromatograph with a spectrophotometric detector (SPD-M20A). Chiral columns Daicel Chiralpak® IB and IC were used for the separation of enantiomers.

Specific optical rotations were measured with AUTOMATIC polarimetry, Autopol III (Rudolph Research, Flandres, New Jersey). Specific optical rotations are given in concentrations c (g/100 mL).

The UV/vis absorption spectra were recorded in CH_2Cl_2 solutions (≈ 1 × 10^{-5}) using a Unicam 340 spectrophotometer. Steady-state fluorescence spectra were monitored on an FLS 980 spectrofluorometer (Edinburgh Instruments). Absolute fluorescence quantum yields were determined using a Quantaurus-QY Plus spectrofluorometer equipped with an integrating sphere (Hamamatsu C13534-33). Fluorescence of air-saturated solutions of samples in HPLC solvents was measured in 1 cm path-length cuvettes using samples with an absorbance of 0.1 or less at the excitation wavelength. Fluorescence decay measurements were conducted with a Fluorolog FL 3-22 fluorometer (Horiba Jobin Yvon) equipped with double grating excitation and emission monochromators, a TBX picosecond photon detection module and a FluoroHub time-correlated single photon counting (TC-SPC) module.

Names of compounds were generated in the ChemDraw application.

2 Optimization of reaction conditions

2.1 Screening of conditions for the condensation reaction

Table S1. Optimization of **9c** synthesis.

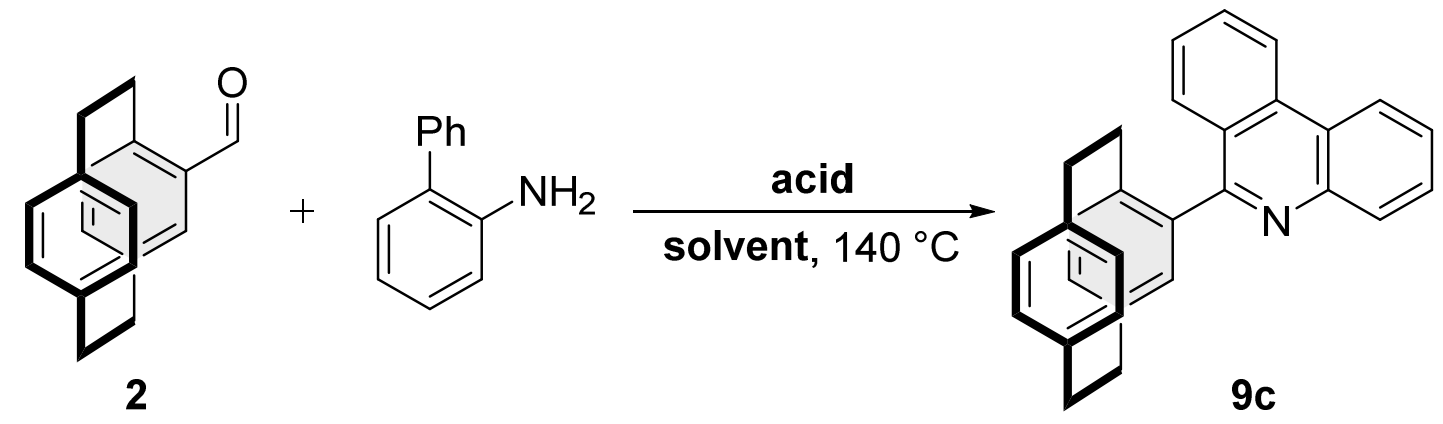

Entry[a]	Acid	Solvent	Amine (equiv.)	*t* (h)	Conversion (%)	Yield (%)[b,c]
1	TFA	–	1.0	24	39	25 (31)
2	TFA	–	2.0	24	56	(43)
3	TFA	–	2.0	72	89	42 (59)
4	TFA	–	3.0	96	–	45[d]
5	TsOH[e]	toluene	1.0	24	50	19
6	TsOH[e]	DCE	3.0	24	–	12
7	TsOH[e]	HFIP	1.0	48	–	14
8	MsOH	–	2.0	24	–	–[f]
9	TfOH	–	1.0	24	–	–[f]
10	TfOH[g]	toluene	1.0	24	8	4
11	H_2SO_4	–	1.0	24	–	–[f]
12	PPA	–	1.0	24	–	No reaction
13	AcOH	–	1.0	24	–	–[f]
14	H_3PO_4	–	1.0	24	–	traces

[a] Reactions were performed in a pressure tube, using 0.2 mmol of **2** and 1 mL of solvent.

[b] Isolated yields.

[c] In parentheses are NMR yields (1,1,2,2-tetrachloroethane).

[d] 2 mmol scale.

[e] 5.0 equiv.

[f] A complex reaction mixture was formed.

[g] 10 mol%.

2.2 Screening of solvent for the borylation reaction

Table S2. Optimization of borylation of **9c**.

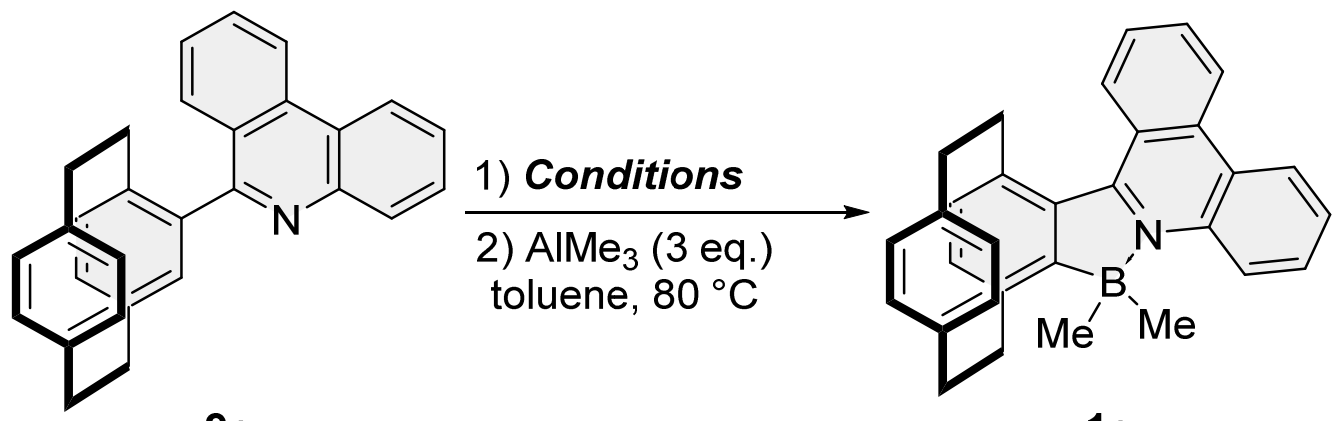

9c **1c**

Entry	BBr_3 (eq.)	DIPEA (eq.)	Solvent	*T* (°C)	Yield (%)[a]
1	3.0	1.0	toluene	110	40
2	3.0	1.0	DCE	60	36
3	4.0	2.0	toluene	110	30
4	4.0	2.0	DCE	80	27
5	3.0	1.0	chlorobenzene	110	57

[a] Isolated yields.

3 Synthetic procedures

3.1 Synthetic procedures of starting compounds

***rac*-4-Formyl[2.2]paracyclophane (*rac*-2).**

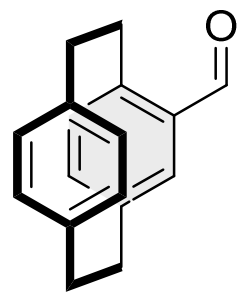

To a cooled suspension of [2.2]paracyclophane (20.0 g, 96 mmol, 1 equiv.) in CH_2Cl_2 (800 mL, 0.12 M), were added titanium (IV) chloride (21 mL, 192 mmol, 2 equiv.) and dichloromethyl methyl ether (9.1 mL, 101 mmol, 1.05 equiv.) at 0 °C. Then, the mixture was stirred at 25 °C overnight, then poured onto ice and stirred for additional 2 hours. The organic phase was extracted with CH_2Cl_2 (3 × 200 mL). The combined organic phases were dried over $MgSO_4$, filtered, and concentrated under reduced pressure. Column chromatography of the residue (220 g silica gel cartridge, flow rate: 110 mL/min, linear gradient: hexanes/CH_2Cl_2 75:25→50:50→0:100) provided 16.1 g (71%) of the title compound as an off-white solid.

R_f (hexanes/CH_2Cl_2 1:1) = 0.42.

^{1}H NMR (400 MHz, $CDCl_3$) δ 9.95 (s, 1H), 7.01 (d, J = 2.0 Hz, 1H), 6.73 (dd, J = 7.8, 2.0 Hz, 1H), 6.62–6.54 (m, 2H), 6.50 (dd, J = 7.9, 1.9 Hz, 1H), 6.45–6.35 (m, 2H), 4.16–4.05 (m, 1H), 3.32–2.89 (m, 7H).

The recorded data agree with the reported values.[1]

(R_p)-Formyl[2.2]paracyclophane ((R_p)-2).

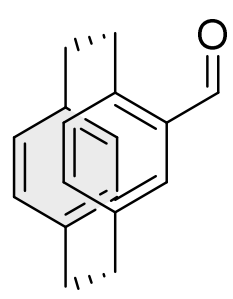

rac-4-Formyl-[2.2]paracyclophane (*rac*)-**2** (1.0 g, 4.2 mmol, 1 equiv.) was dissolved in a mixture of *i*-PrOH/MeCN (1:1, 190 mL, 0.022 M). To the mixture was added a solution of RuCl(*p*-cymene)[(*R*,*R*)-Ts-DPEN] (54 mg, 85 µmol, 2 mol%) and *t*-BuOK (47 mg, 0.42 mmol, 10 mol%) in a mixture of *i*-PrOH/MeCN (1:1, 30 mL, 0.003 M) at 0 °C. The reaction mixture was stirred at 0 °C for 1 hour, then it was quenched with the saturated aq. solution of NH_4Cl (50 mL). The reaction mixture was concentrated under reduced pressure, extracted with EtOAc (3 × 100 mL), the combined organic phases were washed with brine, dried over anhydrous $MgSO_4$, filtered, and concentrated under reduced pressure. Column chromatography of the residue (80 g silica gel cartridge, flow rate: 60 mL/min, linear gradient: hexanes/CH_2Cl_2 75:25→50:50→0:100) gave 360 mg (36%) of the title product as a colorless solid.

---

[1] Kramer, J. J. P.; Yildiz, C.; Nieger, M.; Bräse, S. Eur. *J. Org. Chem.* **2013**, *2014*, 1287–1295.

HPLC conditions: Daicel Chiralpak® IC, heptane/*i*-PrOH 98:2, 1 mL/min, I = 254 nm, t_{major} = 10.74 min, t_{minor} = 13.67 min, ee > 99%.

$[\alpha]_D^{20}$ = -171 (*c* 0.4, $CHCl_3$).

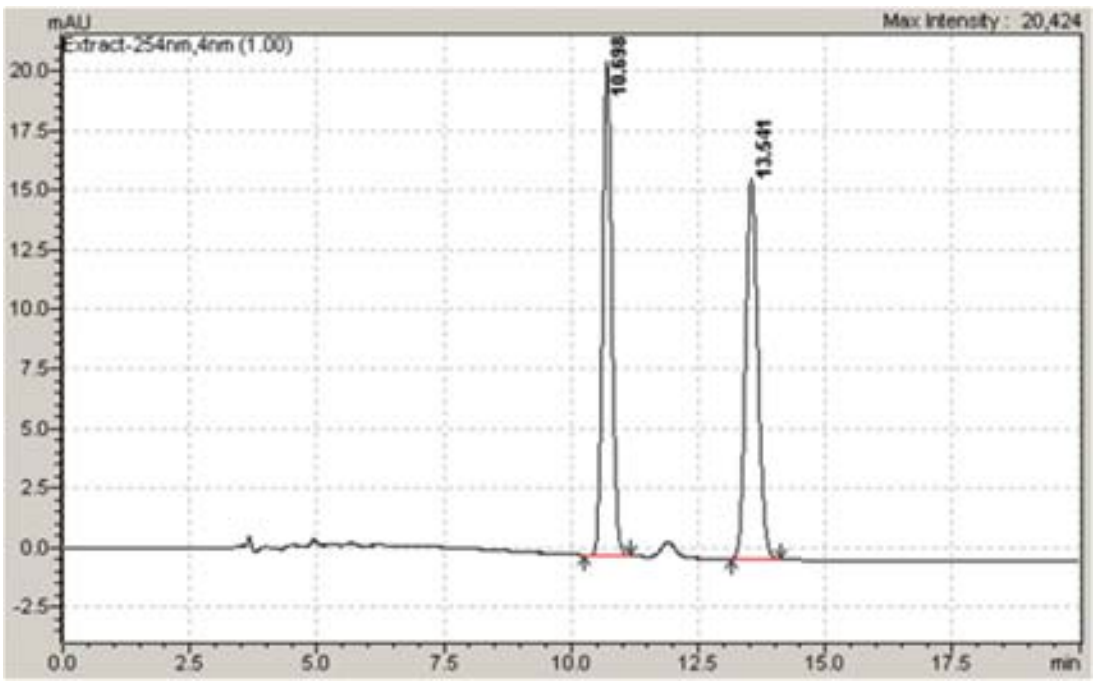

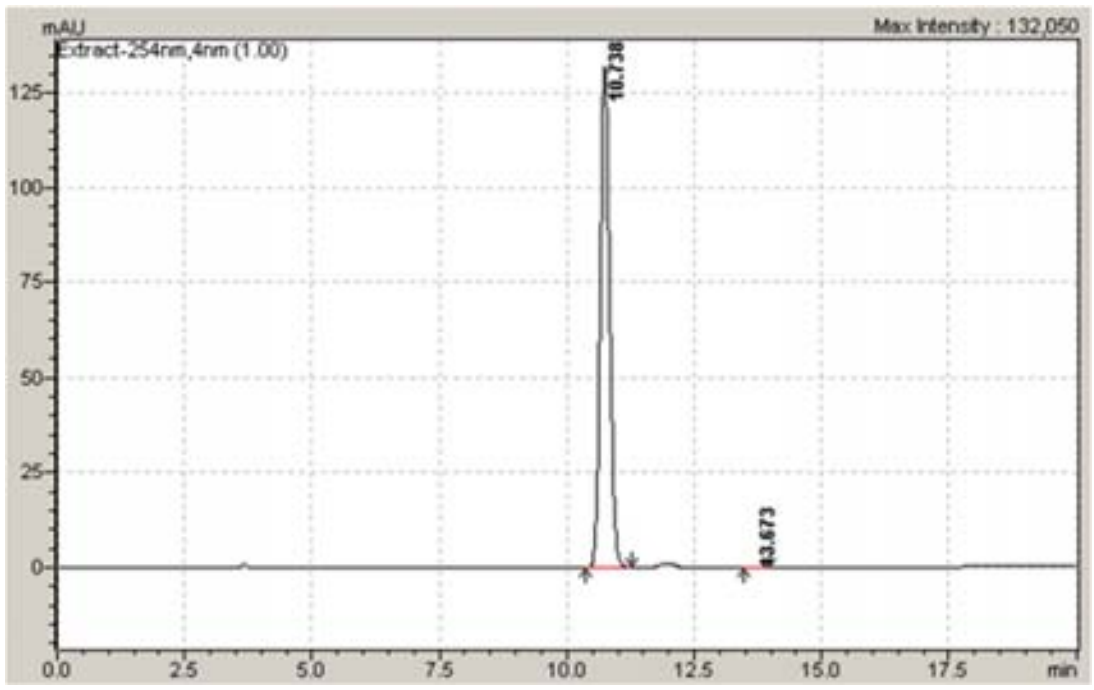

Compd.	Retention time (min)	Area (%)	Compd.	Retention t (min)	Area (%)
(R_p)-**2**	10.70	50	(R_p)-**2**	10.74	99.9
(S_p)-**2**	13.54	50	(S_p)-**2**	13.67	0.1

(S_p)-4-Formyl[2.2]paracyclophane ((S_p)-2).

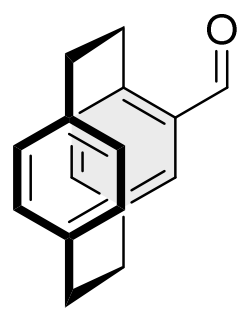

rac-4-Formyl-[2.2]paracyclophane (*rac*)-**2** (2 g, 8.46 mmol, 1 equiv.) was dissolved in a mixture of *i*-PrOH/MeCN (1:1, 380 mL, 0.22 M). To the solution was added a solution of RuCl(*p*-cymene)[(*S*,*S*)-Ts-DPEN] (108 mg, 0.17 mmol, 2 mol%) and *t*-BuOK (95 mg, 0.85 mmol, 10 mol%) in a mixture of *i*-PrOH/MeCN (1:1, 50 mL, 0.003 M) at 0 °C. The reaction mixture was stirred at 0 °C for 1 hour then it was quenched with the saturated aq. solution of NH_4Cl (100 mL). The mixture was concentrated under reduced pressure, extracted with EtOAc (3 × 250 mL), the combined organic phases were washed with brine, dried over anhydrous $MgSO_4$, filtered, concentrated under reduced pressure. Column chromatography of the residue (80 g silica gel cartridge, flow rate: 60 mL/min, linear gradient: hexanes/CH_2Cl_2 75:25→50:50→0:100) gave 760 mg (38%) of the title compound as a colorless solid.

HPLC conditions: Daicel Chiralpak® IC, heptane/*i*-PrOH 98:2, 1 mL/min, I = 254 nm, t_{minor} = 10.26 min, t_{major} = 13.86 min, ee > 99%.

$[\alpha]_D^{20}$ = +198 (*c* 0.4, $CHCl_3$).

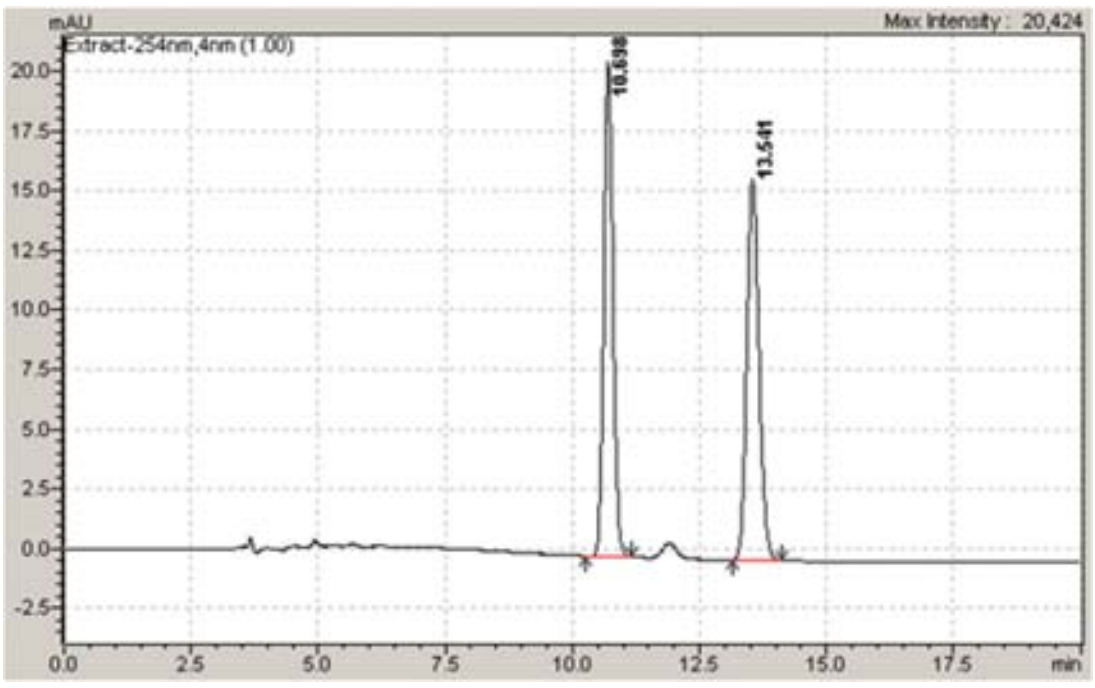

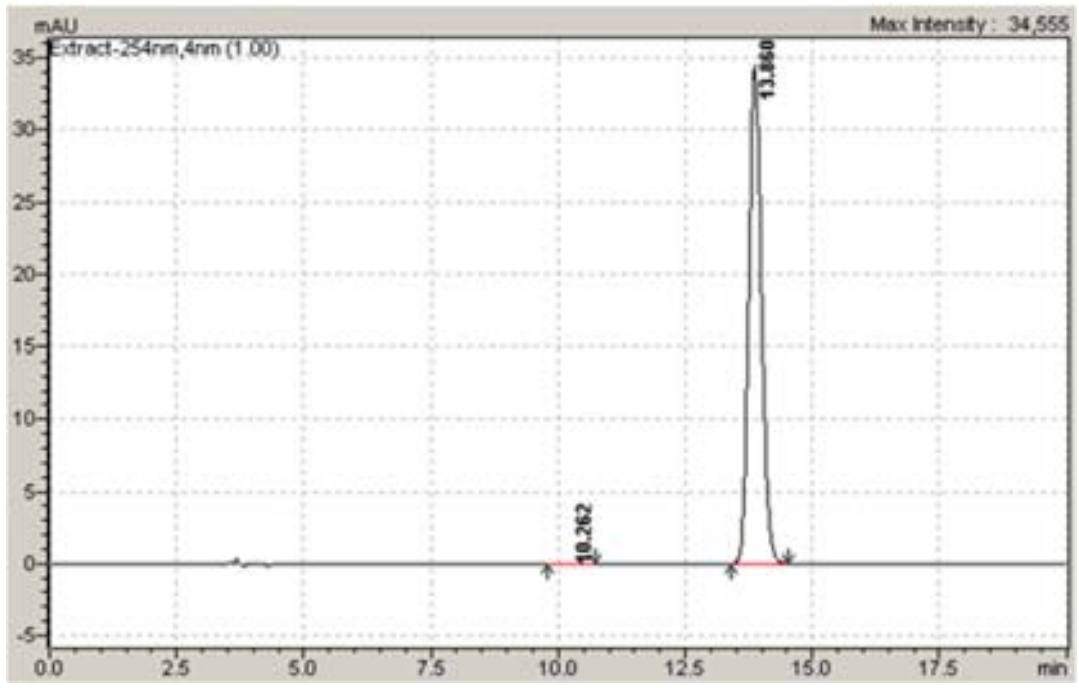

	Retention time (min)	Area (%)
(R_p)-**2**	10.70	50
(S_p)-**2**	13.54	50

Compd	Retention t (min)	Area (%)
(R_p)-**2**	10.26	0.1
(S_p)-**2**	13.86	99.9

Note: Kinetic resolutions were carried out in HPLC grade solvents.

Pathway A:

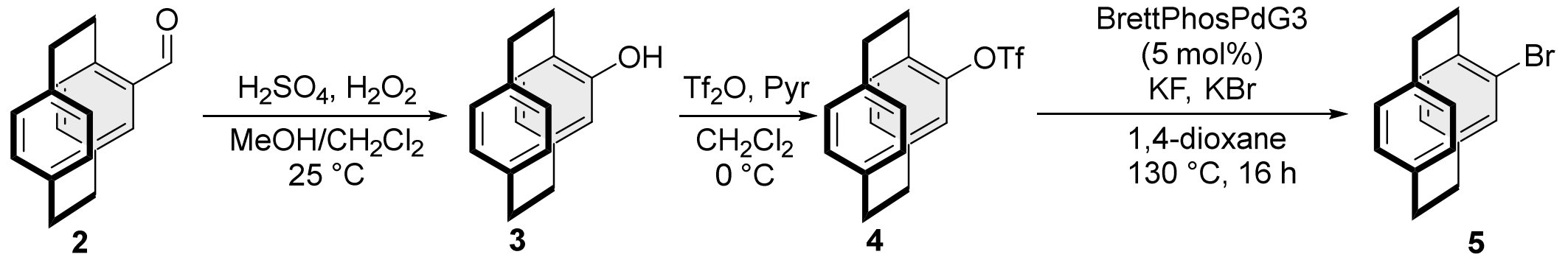

***rac*-4-Hydroxy[2.2]paracyclophane (*rac*-3).**

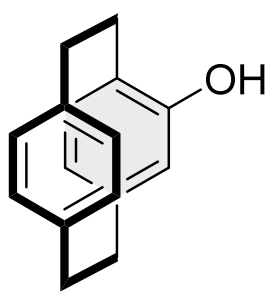

4-Formyl[2.2]paracyclophane *rac*-**2** (528 mg, 2.2 mmol, 1 equiv.) was dissolved in a mixture of CH_2Cl_2/MeOH (1:1, 60 mL, 0.037 M). Then, H_2SO_4 (0.1 mL, 96% aq., 1.8 mmol, 0.8 equiv.) and H_2O_2 (0.31 mL, 30% aq., 3 mmol, 1.4 equiv.) were added subsequently, and the reaction mixture was stirred at 25 °C for 16 hours. Afterwards, it was concentrated under reduced pressure, and the residue was dissolved in CH_2Cl_2 and washed with water. The organic phase was dried over anhydrous Na_2SO_4, filtered, and concentrated under reduced pressure. Column chromatography of the residue (80 g silica gel cartridge, flow rate: 60 mL/min; linear gradient: hexanes/CH_2Cl_2 80:20→50:50→0:100) gave 0.383 g (76%) of the title compound as a colorless solid.

R_f (hexanes/CH_2Cl_2 1:1) = 0.33.

^{1}H NMR (400 MHz, $CDCl_3$) δ 7.00 (dd, J = 7.8, 2.0 Hz, 1H), 6.55 (dd, J = 7.8, 2.0 Hz, 1H), 6.45 (dd, J = 7.9, 2.0 Hz, 1H), 6.42–6.36 (m, 2H), 6.26 (dd, J = 7.7, 1.8 Hz, 1H), 5.54 (d, J = 1.7 Hz, 1H), 4.38 (s, 1H), 3.38–3.27 (m, 1H), 3.16–2.86 (m, 6H), 2.71–2.61 (m, 1H).

The recorded data agree with the reported values.[2]

***rac*-1,4(1,4)-Dibenzenacyclohexaphane-1^2-yl trifluoromethanesulfonate (*rac*-4).**

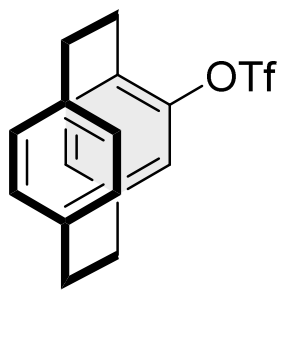

4-Hydroxy[2.2]paracyclophane *rac*-**3** (864 mg, 3.85 mmol, 1 equiv.) and pyridine (1.9 mL, 23.6 mmol, 6.13 equiv.) were dissolved in CH_2Cl_2 (8 mL, 0.5 M) and trifluoromethanesulfonic anhydride (1.3 mL, 7.7 mmol, 2 equiv.) was added dropwise at 0 °C. The reaction mixture was stirred overnight at 23 °C. Afterwards, the reaction was quenched by HCl (1M) followed by extraction with CH_2Cl_2 (100 mL). The organic phase was separated, washed with water and brine, dried over anhydrous Na_2SO_4, filtered, and concentrated under reduced pressure. Column chromatography of the residue (40g silica gel cartridge, flow rate:

[2] Friedmann, C. J.; Ay, S.; Bräse, S. *J. Org. Chem.* **2010**, *75*, 4612–4614.

40 mL/min, linear gradient: hexanes/EtOAc 98:2→90:10) gave 1.197 g (87%) of the title compound as a colorless solid.

R_f (hexanes/EtOAc 9:1) = 0.47.

^{1}H NMR (400 MHz, $CDCl_3$) δ 6.90 (dd, J = 7.9, 2.0 Hz, 1H), 6.63–6.53 (m, 3H), 6.48 (td, J = 7.9, 2.0 Hz, 2H), 6.17 (d, J = 1.0 Hz, 1H), 3.41 (ddd, J = 13.4, 8.8, 3.9 Hz, 1H), 3.22–2.98 (m, 6H), 2.81 (ddd, J = 13.8, 9.5, 6.7 Hz, 1H).

^{13}C{^{1}H} NMR (101 MHz, $CDCl_3$) δ 148.3, 143.1, 139.5, 139.3, 136.2, 133.7, 133.2, 132.7, 132.5, 132.1, 129.7, 127.9, 118.9 (q, J = 320.8 Hz), 35.3, 34.9, 34.2, 31.7.

^{19}F NMR (376 MHz, $CDCl_3$) δ -73.4.

The recorded data are in accordance with the reported values.[3]

rac-4-Bromo[2.2]paracyclophane (_rac_-5).

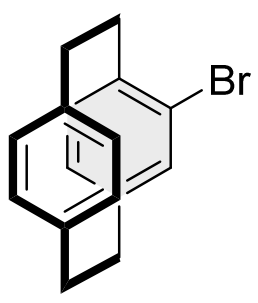

Into a dried MW vial were added *rac*-**4** (70 mg, 0.2 mmol, 1 equiv.), BrettPhosPdG3 (9 mg, 10 µmol, 5 mol%), KF (6 mg, 0.1 mmol, 0.5 equiv.) and KBr (48 mg, 0.4 mmol, 2 equiv.). After filling the tube with argon, dry 1,4-dioxane (1 mL, 0.2 M) was added and the reaction mixture was stirred at 130 °C (oil bath) for 16 hours. Then, the mixture was cooled down, filtered over silica gel pad (EtOAc eluent), and concentrated under reduced pressure. Column chromatography of the residue (12 g silica gel cartridge, flow rate: 20 mL/min, linear gradient: hexanes/CH_2Cl_2 100:0→90:10) provided 39 mg (69%) of the title compound as a colorless solid.

---

[3] Zhang, T.-Z.; Dai, L.-X.; Hou, X.-L. *Tetrahedron: Asymmetry* **2007**, *18*, 251–259.

Pathway B

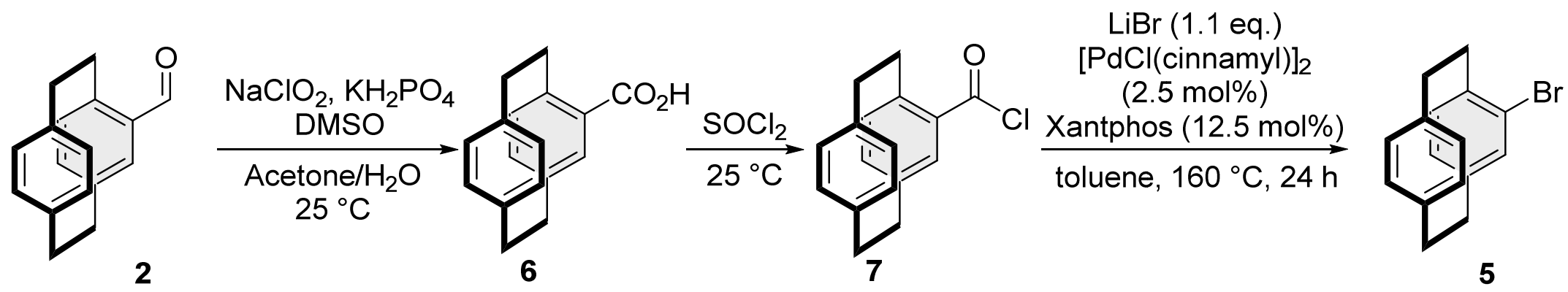

***rac*-1,4(1,4)-Dibenzenacyclohexaphane-1^2-carboxylic acid (*rac*-6).**

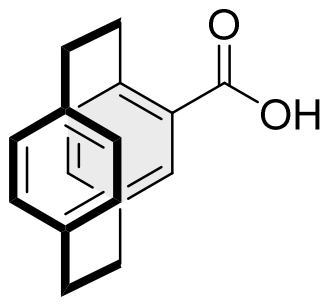

To a suspension of 4-formyl[2.2]paracyclophane *rac*-**2** (218 mg, 0.92 mmol, 1 equiv.) in a mixture of acetone/H_2O/DMSO (8:6:3.2 mL, 0.05 M) were added by portion of KH_2PO_4 (0.626 g, 4.6 mmol, 5 equiv.) and 80% $NaClO_2$ (0.520 g, 4.6 mmol, 5 equiv.) at 25 °C and the reaction mixture was stirred overnight. Then, it was concentrated under reduced pressure. The residue was extracted with Et_2O (2 × 50 mL), the combined organic phases were washed with brine, dried over $MgSO_4$, filtered, and concentrated under reduced pressure. The residue was flushed through a small silica gel pad (Et_2O eluent) and after concentration, the carboxylic acid *rac*-**6** (232 mg, quantitative) was directly used into the next step without further purification.

^{1}H NMR (400 MHz, $CDCl_3$) δ 7.31 (d, J = 2.0 Hz, 1H), 6.72 (dd, J = 7.8, 2.0 Hz, 1H), 6.65–6.57 (m, 3H), 6.55–6.50 (m, 2H), 4.23 (ddd, J = 12.9, 8.8, 2.7 Hz, 1H), 3.27–3.14 (m, 4H), 3.12–3.00 (m, 2H), 2.91 (ddd, J = 12.9, 9.8, 7.3 Hz, 1H).

The recorded data agree with the reported values.[4]

***rac*-1,4(1,4)-Dibenzenacyclohexaphane-1^2-carbonyl chloride (*rac*-7).**

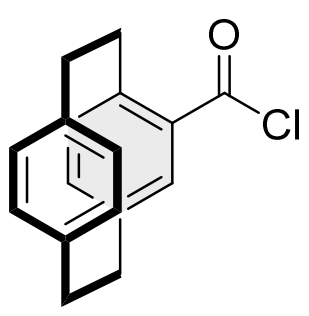

[2.2]Paracyclophane-4-carboxylic acid *rac*-**6** (228 mg, 0.90 mmol, 1 equiv.) was dissolved in thionyl chloride (5 mL, 0.18 M) and stirred at 25 °C for 4 hours. Then, excess of thionyl chloride was removed under reduced pressure. To the reaction mixture toluene (5 mL) was added and evaporated to give 241 mg (99%) of the title compound, which was used without further purification in the next step.

^{1}H NMR (400 MHz, $CDCl_3$) δ 7.40 (d, J = 1.9 Hz, 1H), 6.77 (dd, J = 7.8, 1.9 Hz, 1H), 6.62–6.54 (m, 3H), 6.53–6.45 (m, 2H), 3.90 (ddd, J = 13.0, 9.7, 1.8 Hz, 1H), 3.27–3.00 (m, 6H), 2.91 (ddd, J = 13.1, 10.2, 6.9 Hz, 1H).

---

[4] Braun, C.; Nieger, M.; Thiel, W. R.; Bräse, S. *Chem. Eur. J.* **2017**, *23*, 15474–15483.

$^{13}C\{^1H\}$ NMR (101 MHz, $CDCl_3$) δ 167.1, 144.5, 141.1, 139.8, 139.6, 138.9, 138.4, 136.5, 133.6, 133.3, 133.1, 132.5, 131.6, 36.2, 35.3, 35.2, 34.5.
The recorded data agree with the reported values.[5]

rac-4-Bromo[2.2]paracyclophane (_rac_-5).

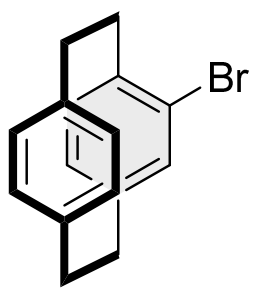

Into a dried Schlenk pressure tube, [2.2]paracyclophane-4-carboxylic acid chloride *rac*-**7** (213 mg, 0.79 mmol, 1 equiv.), [PdCl(cinnamyl)]$_2$ (10 mg, 19 µmol, 2.5 mol%), XanthPhos (57 mg, 0.10 mmol, 12.5 mol%) and anhydrous LiBr (75 mg, 0.86 mmol, 1.1 equiv.) were added. After flushing the tube with argon, dry toluene (4 mL, 0.2 M) was added, and the reaction mixture was stirred at 160 °C (oil bath) for 24 hours. Then, the mixture was cooled down, filtered over Celite® pad, which was washed with CH_2Cl_2, and concentrated under reduced pressure. Column chromatography of the residue (40 g silica gel cartridge, flow rate: 40 mL/min, linear gradient: hexanes/CH_2Cl_2 100:0→90:10) gave 176 mg (78%) of the title compound as a colorless solid.
R_f (hexanes/CH_2Cl_2 9:1) = 0.45
^1H NMR (400 MHz, $CDCl_3$) δ 7.16 (dd, J = 7.9, 2.0 Hz, 1H), 6.56 (td, J = 8.0, 1.9 Hz, 1H), 6.51 (s, 2H), 6.50–6.43 (m, 3H), 3.46 (ddd, J = 13.4, 10.1, 2.2 Hz, 1H), 3.20 (ddd, J = 13.0, 10.0, 6.0 Hz, 1H), 3.15–3.02 (m, 4H), 2.96–2.89 (m, 1H), 2.82 (ddd, J = 13.3, 10.5, 6.0 Hz, 1H).
$^{13}C\{^1H\}$ NMR (101 MHz, $CDCl_3$) δ 141.7, 139.5, 139.2 (2C), 137.4, 135.2, 133.4, 133.0, 132.4, 131.6, 128.8, 127.1, 36.0, 35.6, 35.0, 33.6.
The recorded data agree with the reported values.[6]

(R_p)-4-Bromo[2.2]paracyclophane ((R_p)-5).

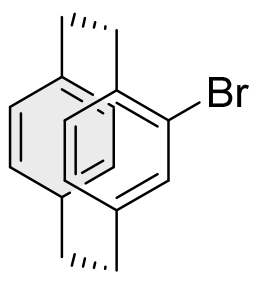

The title compound was prepared according to the above-mentioned procedure for preparation of ***rac*-5** from (R_p)-4-formyl[2.2]paracyclophane (R_p)-**2** (320 mg, 1.35 mmol). Intermediate acid (R_p)-**6** and acyl chloride (R_p)-**7** were obtained in quantitative and 99% yields, respectively. Column chromatography of the crude pCp-bromide (40 g silica gel cartridge, flow rate: 40 mL/min, linear gradient: hexanes/CH_2Cl_2 100:0→90:10) gave 183 mg (52%) of the title compound as a colorless solid.

---
5 Psiorz, M.; Schmid, R. *Chem. Ber.* **1987**, *120*, 1825–1828.
6 Braun, C.; Nieger, M.; Thiel, W. R.; Bräse, S. *Chem. Eur. J.* **2017**, *23*, 15474–15483.

HPLC conditions: Daicel Chiralpak® OD-H, heptane/*i*-PrOH 99.5:0.5, 0.3 mL/min, I = 254 nm, t_{minor} = 47.60 min, t_{major} = 44.48 min, ee > 99%.

Its spectral characteristics (^{1}H and ^{13}C NMR, IR, MS) were identical with those of ***rac*-5**.

$[\alpha]_D^{20} =$ - 128 (*c* 0.4, $CHCl_3$).

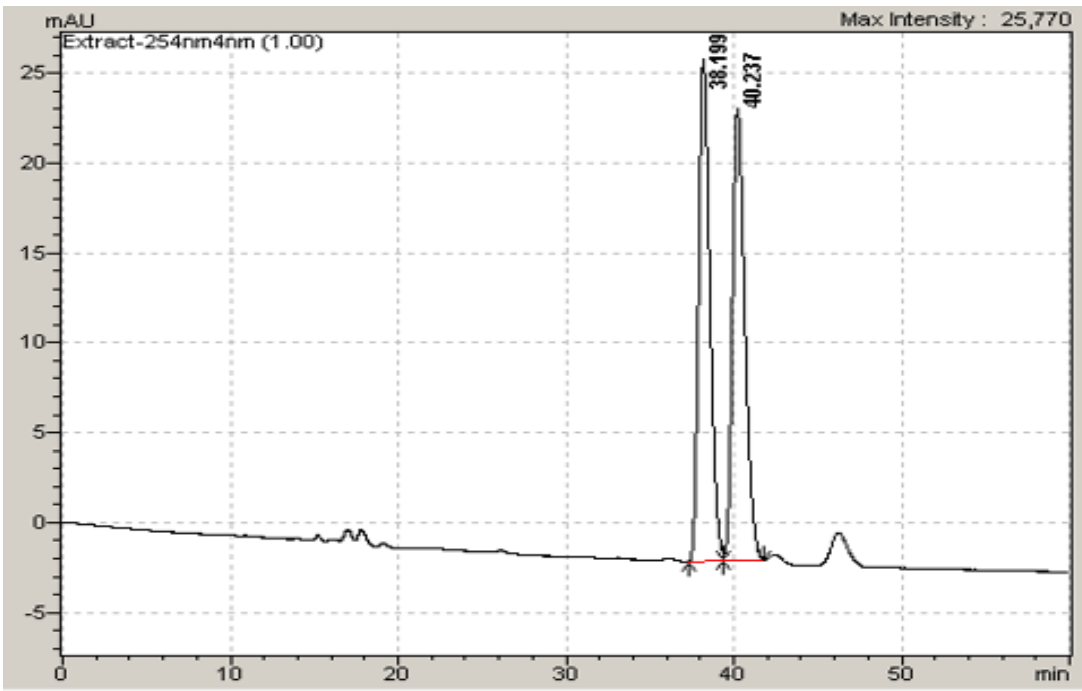

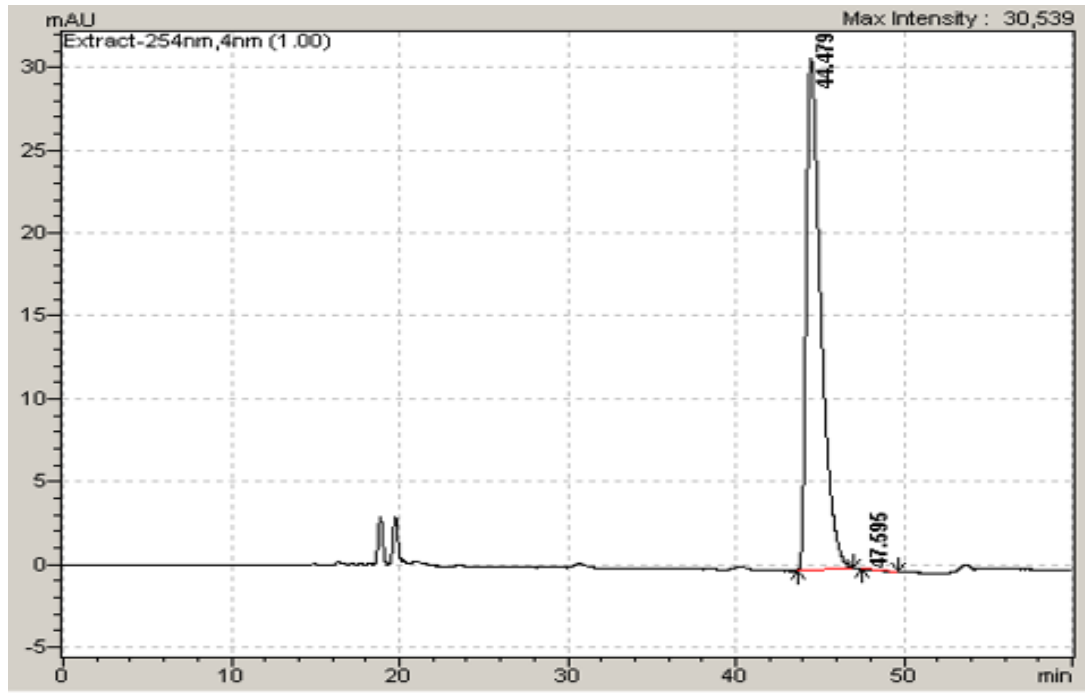

	Retention time (min)	Area (%)
(R_p)-**5**	38.20	50
(S_p)-**5**	40.24	50

Compd	Retention t (min)	Area (%)
(R_p)-**5**	44.48	99.9
(S_p)-**5**	47.60	0.1

(S_p)-4-Bromo[2.2]paracyclophane ((S_p)-5).

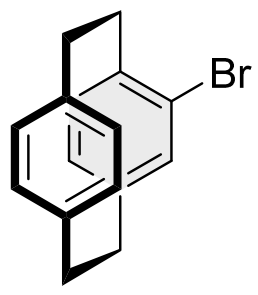

The title compound was prepared according to the above-mentioned procedure for preparation of ***rac*-5** from (S_p)-4-formyl[2.2]paracyclophane (S_p)-**2** (410 mg, 1.73 mmol). Intermediate acid (S_p)-**6** and acyl chloride (S_p)-**7** were obtained in quantitative and 98% yields, , respectively. Column chromatography of the crude pCp-bromide (40 g silica gel cartridge, flow rate: 40 mL/min, linear gradient: hexanes/CH_2Cl_2 100:0→90:10) gave 276 mg (59%) of the title compound as a colorless solid.

HPLC conditions: Daicel Chiralpak® OD-H, heptane/*i*-PrOH 99.5:0.5, 0.3 mL/min, I = 254 nm, t_{minor} = 45.21 min, t_{major} = 47.00 min, ee > 99%.

Its spectral characteristics (^{1}H and ^{13}C NMR, IR, MS) were identical with those of ***rac*-5**.

$[\alpha]_D^{20} =$ + 132 (*c* 0.4, $CHCl_3$).

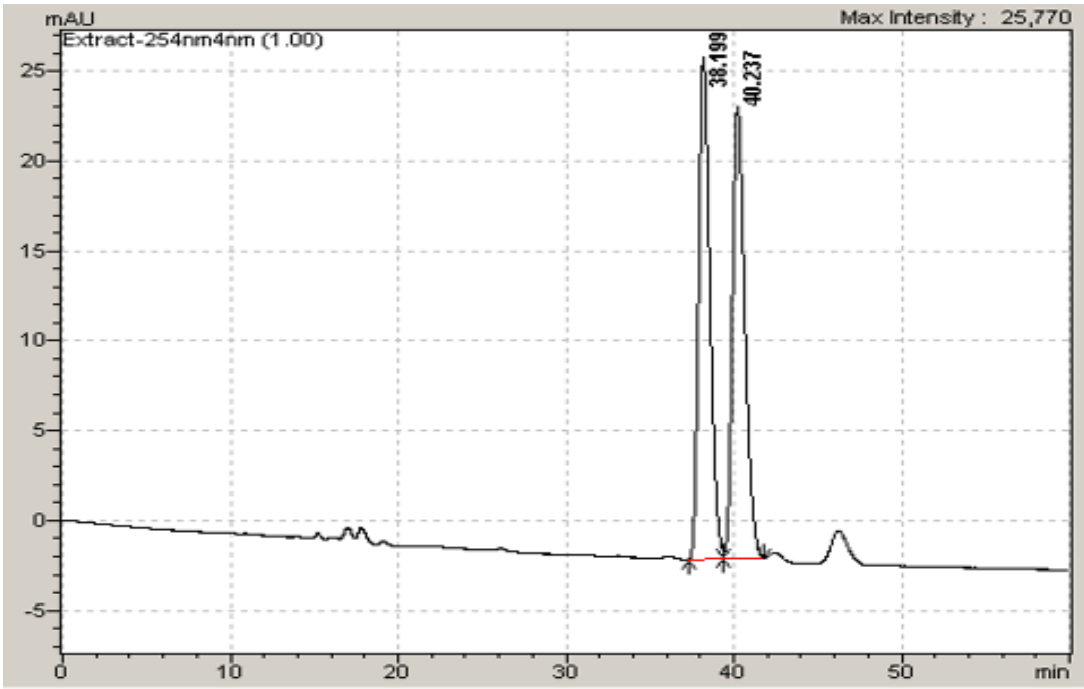

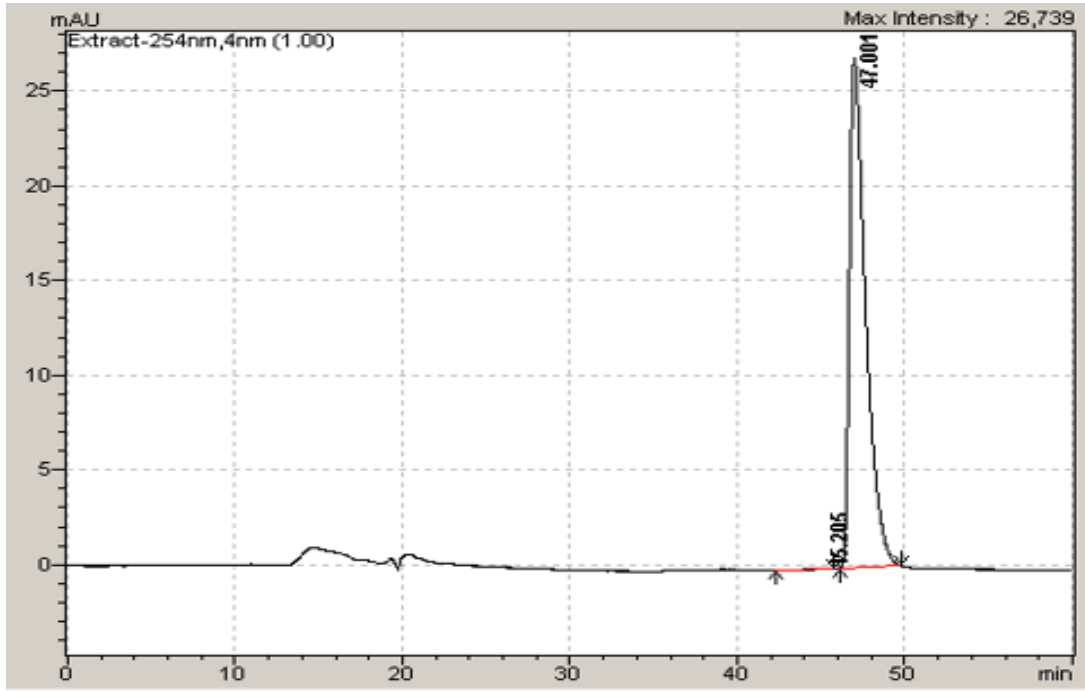

	Retention time (min)	Area (%)
(R_p)-**5**	38.20	50
(S_p)-**5**	40.24	50

Compd	Retention t (min)	Area (%)
(R_p)-**5**	45.21	0.1
(S_p)-**5**	47.00	99.9

Notes regarding syntheses of **5**.

It should be noted that yields of the decarbonylative halogenation step depend on a reaction scale (the large scale the lower yields) and the size of a reaction flask. This associated with reversible of CO insertion into the organopalladium intermediate. Since the syntheses of (S_p)- and (R_p)-**5** were carried out at 1.6 and 1.2 mmol scale (whereas that for *rac*-**5** in 0.8 mmol one) a somewhat lower yields are not surprising. We are pleased to demonstrate that the pathway B allow the preparation of highly enantioenriched pCp-bromide **5** without using any chiral resolution of enantiomers on preparative scale on HPLC.

3.2 Synthesis of 9

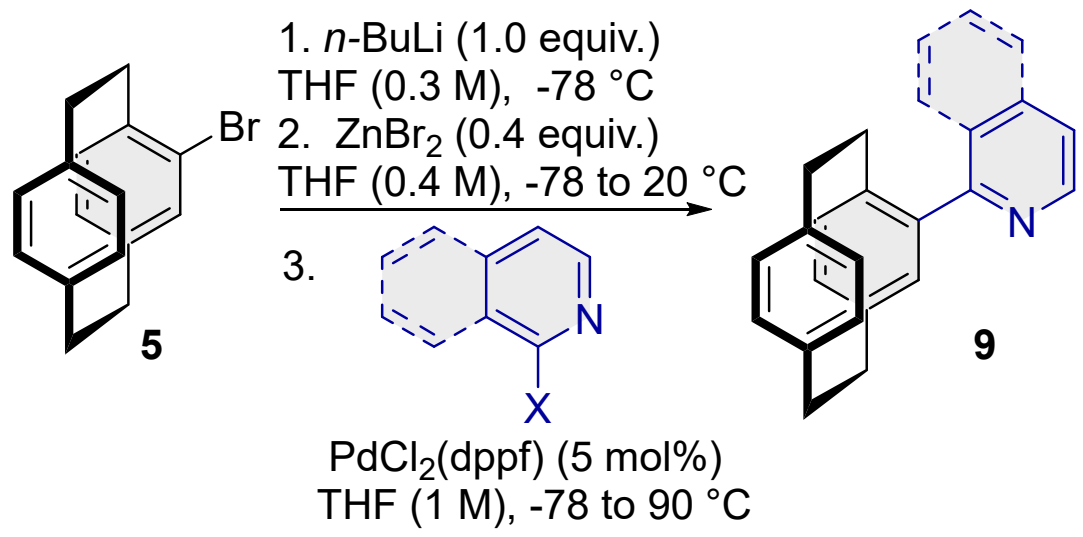

General procedure 1 (GP1). A general procedure for Negishi coupling.

In a flame-dried Schlenk flask, 4-Bromo[2.2]paracyclophane **5** (1.0 equiv.) was dissolved in dry THF (~0.3 M). Then, *n*-butyllithium (1.0 equiv.) was added at -78 °C and the reaction mixture was stirred for 1 h. Afterwards, a solution of freshly sublimated $ZnBr_2$ in THF (~0.4 M, 0.4 equiv.) was added, and the reaction mixture was warmed up to room temperature (23 °C) and stirred for an additional hour. The prepared organozinc reagent was then added to a solution of heteroaryl halide **8** (1.2 equiv.) and $PdCl_2$dppf (5 mol%) in THF (1 M). The resulting reaction mixture was stirred at 90 °C (oil bath) for 16 hours, quenched with water, and extracted with Et_2O (2 × 30 mL/mmol). The combined organic fractions were washed with brine, dried over anhydrous $MgSO_4$, and concentrated under reduced pressure. The residue was subjected to column chromatography.

***rac*-2-([2.2]Paracyclophan-4-yl)pyridine (*rac*-9a).**

A synthesis of *rac*-9a from triflate *rac*-4:

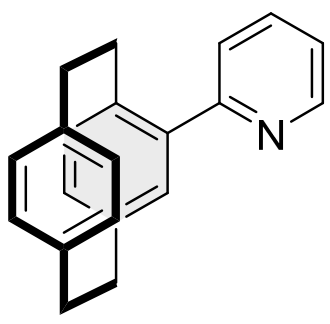

Into a dried MW vial, [2.2]paracyclophan-4-yl trifluoromethanesulfonate *rac*-**4** (367 mg, 1.0 mmol, 1 equiv.), $Pd(PPh_3)_4$ (116 mg, 0.1 mmol, 10 mol%), LiCl (85 mg, 2 mmol, 2 equiv.) and CuCl (99 mg, 1.0 mmol, 1 equiv.) were added. The vial was flushed with argon and dry DMF (5 mL, 0.2 M) and 2-(tributylstannyl)pyridine (442 mg, 1.2 mmol, 1.2 equiv.) were added. Argon was bubbled into the reaction mixture and stirred at 100 °C (oil bath) for 16 hours. After cooling down, 3 M aqueous solution of KF (1.3 mL) was added and stirred for additional 30 min. The mixture was filtered over a pad of Celite®, the filtrate was extracted with dichloromethane (3 × 50 mL), the combined organic phases were dried over Na_2SO_4, filtered, and concentrated under reduced pressure. Column chromatography of the residue (40 g silica gel cartridge, flow rate: 40 mL/min, linear gradient: hexanes/EtOAc

100:0→95:5→90:10) and subsequent recrystallization from hexanes gave 104 mg (35%) of the pure title product as a colorless solid.

A synthesis of *rac*-9a from bromide *rac*-5:

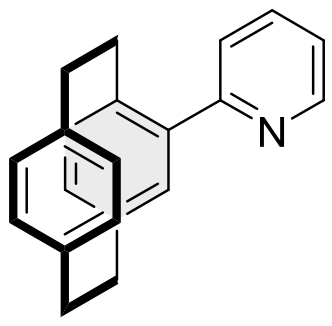

The title compound was prepared according to general procedure **GP1**. The organozinc reagent was prepared from 4-bromo[2.2]paracyclophane *rac*-**5** (287 mg, 1.0 mmol, 1 equiv.), *n*-BuLi (0.68 mL, 1.47 M, 1 mmol, 1 equiv.), THF (3 mL, 0.3 M) and solution of $ZnBr_2$ (90 mg, 0.4 mmol, 0.4 equiv.) in THF (1.0 mL, 0.4 M). For the Negishi coupling was used 2-bromopyridine (**8a**) (0.11 ml, 1.2 mmol, 1.2 equiv.), $PdCl_2$dppf (37 mg, 51 µmol, 5 mol%) and THF (1 mL, 1.2 M). Column chromatography of the residue (40 g silica gel cartridge, flow rate: 40 mL/min, linear gradient: hexanes/EtOAc 100:0→95:5→90:10) gave 202 mg (71%) of the title product as a colorless solid.

R_f (hexanes/EtOAc 9:1) = 0.20.

^{1}H NMR (400 MHz, $CDCl_3$) δ 8.76 (dt, J = 4.9, 1.4 Hz, 1H), 7.78 (td, J = 7.7, 1.9 Hz, 1H), 7.52 (dd, J = 8.0, 1.3 Hz, 1H), 7.25–7.22 (m, 1H), 6.83 (d, J = 1.9 Hz, 1H), 6.64–6.53 (m, 6H), 3.75–3.62 (m, 1H), 3.26–3.13 (m, 2H), 3.12–3.01 (m, 2H), 3.01–2.90 (m, 2H), 2.72–2.60 (m, 1H).

^{13}C{^{1}H} NMR (101 MHz, $CDCl_3$) δ 159.3, 149.8, 140.7, 139.9, 139.7, 139.5, 138.3, 136.4, 136.3, 133.3, 133.0, 132.8, 132.7, 132.5, 130.8, 124.3, 121.5, 35.6, 35.4, 35.2, 34.6.

The recorded data agree with the reported values.[7]

(R_p)-2-([2.2]Paracyclophan-4-yl)pyridine ((R_p)-9a).

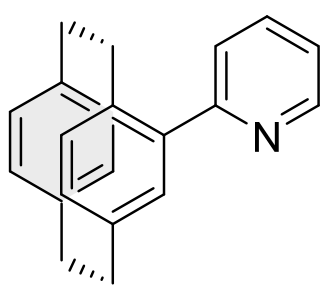

The title compound was prepared according to general procedure **GP1**. The organozinc reagent was prepared from (R_P)-4-bromo[2.2]paracyclophane (R_P)-**5** (178 mg, 0.62 mmol, 1 equiv.), *n*-BuLi (0.42 mL, 1.47 M, 0.62 mmol, 1 equiv.), THF (1.5 mL, 0.4 M) and solution of $ZnBr_2$ (56 mg, 0.25 mmol, 0.4 equiv.) in THF (0.5 mL, 0.5 M). For the Negishi coupling was used 2-bromopyridine (**8a**) (70 µl, 0.73 mmol, 1.2 equiv.), $PdCl_2$dppf (23 mg, 31 µmol, 5 mol%) and THF (0.5 mL, 1.5 M). Column chromatography of the residue (40 g silica gel cartridge, flow rate: 40 mL/min, linear gradient: hexanes/EtOAc 100:0→95:5→90:10) gave 111 mg (63%) of the title compound as a colorless solid.

Its spectral characteristics (^{1}H and ^{13}C NMR, IR, MS) were identical with those of ***rac*-9a**.

$[\alpha]_D^{20}$ = - 68 (c 0.25, $CHCl_3$).

[7] Braun, C.; Nieger, M.; Bräse, S. *Chem. Eur. J.* **2017**, *23*, 16452–16455.

(S_p)-2-([2.2]Paracyclophan-4-yl)pyridine ((S_p)-9a).

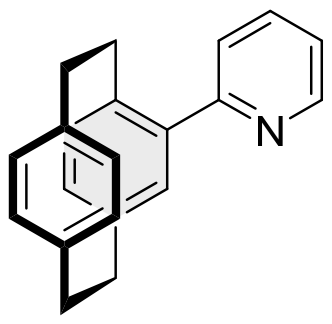

The title compound was prepared according to general procedure **GP1**. The organozinc reagent was prepared from (S_p)-4-bromo[2.2]paracyclophane (S_p)-**5** (267 mg, 0.93 mmol, 1 equiv.), *n*-BuLi (0.63 mL, 1.47 M, 0.93 mmol, 1 equiv.), THF (3 mL, 0.3 M) and solution of $ZnBr_2$ (83 mg, 0.37 mmol, 0.4 equiv.) in THF (1 mL, 0.4 M). For the Negishi coupling was used 2-bromopyridine (**8a**) (0.11 ml, 1.2 mmol, 1.2 equiv.), $PdCl_2$dppf (34 mg, 47 µmol, 5 mol%) and THF (1 mL, 1.2 M). Column chromatography of the residue (40 g silica gel cartridge, flow rate: 40 mL/min, linear gradient: hexanes/EtOAc 100:0→95:5→90:10) gave 187 mg (70%) of the title compound as a colorless solid.

Its spectral characteristics (^{1}H and ^{13}C NMR, IR, MS) were identical with those of ***rac*-9a**.

$[\alpha]_D^{20}$ = + 75 (c 0.4, $CHCl_3$).

***rac*-1-([2.2]Paracyclophan-4-yl)isoquinoline (*rac*-9b).**

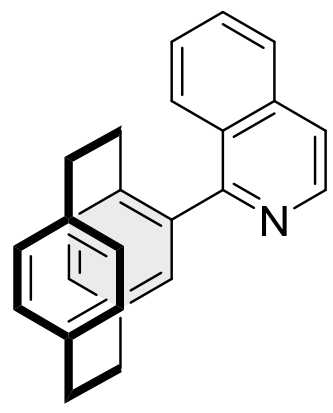

The title compound was prepared according to general procedure **GP1**. The organozinc reagent was prepared from 4-bromo[2.2]paracyclophane *rac*-**5** (287 mg, 1.0 mmol, 1 equiv.), *n*-BuLi (0.68 mL, 1.47 M, 1.0 mmol, 1 equiv.), THF (3 mL, 0.3 M) and solution of $ZnBr_2$ (90 mg, 0.4 mmol, 0.4 equiv.) in THF (1 mL, 0.4 M). For the Negishi coupling was used 1-bromoisoquinoline (**8b**) (250 mg, 1.2 mmol, 1.2 equiv.), $PdCl_2$dppf (37 mg, 51 µmol, 5 mol%) and THF (1 mL, 1.2 M). Column chromatography of the residue (80 g silica gel cartridge, flow rate: 80 mL/min, linear gradient: cyclohexane/EtOAc 100:0→95:5) gave 167 mg (50%, 90% NMR purity) of the title compound as an off-white solid.

R_f (cyclohexane/EtOAc 95:5) = 0.31.

M.p. = 135.0–135.3 °C.

^{1}H NMR (400 MHz, $CDCl_3$) δ 8.79 (d, J = 5.6 Hz, 1H), 7.90 (d, J = 8.5 Hz, 1H), 7.85 (d, J = 8.2 Hz, 1H), 7.69–7.57 (m, 2H), 7.43 (ddd, J = 8.3, 6.8, 1.3 Hz, 1H), 6.96 (dd, J = 7.3, 1.8 Hz, 2H), 6.74–6.53 (m, 5H), 3.32–3.16 (m, 2H), 3.14–3.01 (m, 2H), 2.94–2.66 (m, 4H).

^{13}C{^{1}H} NMR (101 MHz, $CDCl_3$) δ 160.3, 142.8, 139.9, 139.6, 139.5, 139.5, 137.4, 136.5, 134.8, 134.1, 133.8, 133.7, 132.5, 132.2, 132.0, 129.7, 127.71, 127.66, 127.1, 126.9, 119.6, 35.6, 35.4 (2 C), 34.7.

HRMS (ESI) calcd for $C_{25}H_{22}N$ [M+H]$^+$: 336.1747, found: 336.1749.

IR (ATR) ν_{max} 3043, 2927, 2852, 1550, 1348, 823, 806, 754, 733, 644, 633 cm^{-1}.

(R_p)-1-([2.2]Paracyclophan-4-yl)isoquinoline ((R_p)-9b).

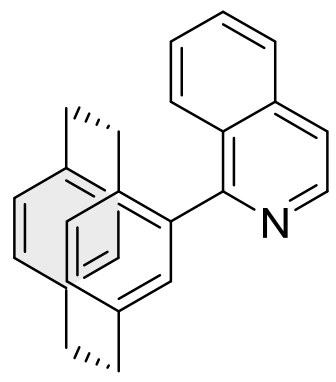

The title compound was prepared according to general procedure **GP1**. The organozinc reagent was prepared from (R_p)-4-bromo[2.2]paracyclophane (R_p)-**5** (158 mg, 0.55 mmol, 1 equiv.), *n*-BuLi (0.37 mL, 1.47 M, 0.55 mmol, 1 equiv.), THF (1.5 mL, 0.37 M) and solution of $ZnBr_2$ (50 mg, 0.22 mmol, 0.4 equiv.) in THF (0.5 mL, 0.44 M). For the Negishi coupling was used 1-bromoisoquinoline (**8b**) (137 mg, 0.66 mmol, 1.2 equiv.), $PdCl_2$dppf (20 mg, 27 µmol, 5 mol%) and THF (0.5 mL, 1.3 M). Column chromatography of the residue (80 g silica gel cartridge, flow rate: 80 mL/min, linear gradient: cyclohexane/EtOAc 100:0→95:5) gave 62 mg (34%, 93% NMR purity) of the title compound as an off-white solid.

Its spectral characteristics (^{1}H and ^{13}C NMR, IR, MS) were identical with those of ***rac*-9b**.

$[\alpha]_D^{20}$ = + 124 (*c* 0.3, $CHCl_3$).

(S_p)-1-([2.2]Paracyclophan-4-yl)isoquinoline ((S_p)-9b).

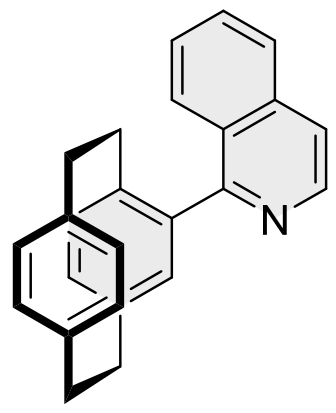

The title compound was prepared according to general procedure **GP1**. The organozinc reagent was prepared from (S_p)-4-bromo[2.2]paracyclophane (S_p)-**5** (258 mg, 0.9 mmol, 1 equiv.), *n*-BuLi (0.61 mL, 1.47 M, 0.9 mmol, 1 equiv.), THF (3 mL, 0.3 M) and solution of $ZnBr_2$ (81 mg, 0.36 mmol, 0.4 equiv.) in THF (1 mL, 0.36 M). For the Negishi coupling was used 1-bromoisoquinoline (**8b**) (225 mg, 1.1 mmol, 1.2 equiv.), $PdCl_2$dppf (33 mg, 45 µmol, 5 mol%) and THF (1 mL, 1.1 M). Column chromatography of the residue (80 g silica gel cartridge, flow rate: 80 mL/min, linear gradient: cyclohexane/EtOAc 100:0→95:5) gave 139 mg (46%, 92% NMR purity) of the title compound as an off-white solid.

Its spectral characteristics (^{1}H and ^{13}C NMR, IR, MS) were identical with those of ***rac*-9b**.

$[\alpha]_D$ = - 135 (*c* 0.4, $CHCl_3$).

***rac*-6-([2.2]Paracyclophan-4-yl)phenanthridine (*rac*-9c).**

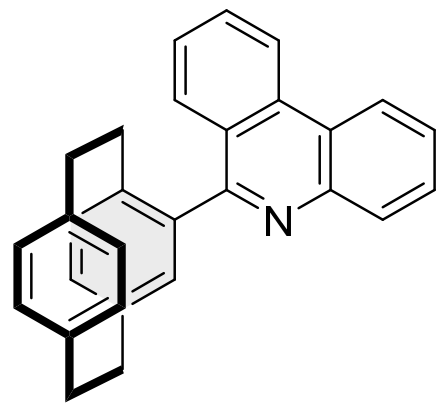

4-Formyl[2.2]paracyclophane *rac*-**2** (473 mg, 2 mmol, 1 equiv.) and 2-phenylaniline (1.0 g, 6 mmol, 3 equiv.) were placed in a pressure tube and 2,2,2-trifluoroacetic acid (10 mL, 0.2 M) was added. The mixture was stirred at 140 °C (oil bath) for four days. The reaction mixture was cooled to room

temperature and concentrated under reduced pressure. 10% aq. solution of NaOH (50 mL) was added to it and the suspension was stirred at 120 °C (oil bath) for 1 hour. After cooling, the suspension was extracted with CH_2Cl_2 (3 × 40 mL), the combined organic phases were dried over anhydrous Na_2SO_4, filtered, and concentrated under reduced pressure. Column chromatography of the residue (80 g silica gel cartridge, flow rate: 60 mL/min, linear gradient: hexanes/CH_2Cl_2 4:1→3:1) gave 345 mg (45%) of the title compound as a colorless solid.
R_f (hexanes/CH_2Cl_2 1:1) = 0.48.
^{1}H NMR (400 MHz, $CDCl_3$) δ 8.72–8.62 (m, 2H), 8.43 (dd, J = 8.2, 1.4 Hz, 1H), 7.91 (d, J = 8.3 Hz, 1H), 7.88–7.68 (m, 3H), 7.50 (ddd, J = 8.2, 6.9, 1.2 Hz, 1H), 7.23 (dd, J = 7.8, 1.9 Hz, 1H), 6.97 (d, J = 1.8 Hz, 1H), 6.73 (dd, J = 7.8, 1.9 Hz, 1H), 6.70–6.63 (m, 2H), 6.61 (d, J = 7.6 Hz, 2H), 3.32–3.17 (m, 2H), 3.14–3.04 (m, 2H), 2.94–2.79 (m, 4H).
$^{13}C\{^1H\}$ NMR (101 MHz, $CDCl_3$) δ 160.6, 144.4, 139.9, 139.7, 139.6, 139.5, 137.1, 134.8, 134.2, 133.83, 133.76, 133.0, 132.6, 132.4, 131.9, 131.0, 130.2, 128.71, 128.65, 127.2, 126.8, 126.2, 123.7, 122.13, 122.06, 35.60, 35.56, 35.4, 34.6.
The recorded data agree with the reported values.[8]

(*R*p)-6-([2.2]Paracyclophan-4-yl)phenanthridine ((*R*p)-9c).

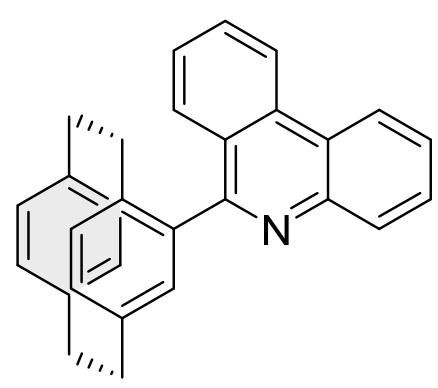

The title compound was prepared according to the above-mentioned procedure for preparation of ***rac*-9c** from (*R*p)-4-formyl[2.2]paracyclophane (*R*p)-**2** (236 mg, 1 mmol, 1 equiv.), 2-phenylaniline (508 mg, 3 mmol, 3 equiv.) and 2,2,2-trifluoroacetic acid (5 mL, 0.2 M). Column chromatography of the residue (80 g silica gel cartridge, flow rate: 60 mL/min, linear gradient: hexanes/CH_2Cl_2 4:1→3:1) gave 169 mg (44%) of the title compound as a colorless solid.
Its spectral characteristics (^{1}H and ^{13}C NMR, IR, MS) were identical with those of ***rac*-9c**.
$[\alpha]_D^{20}$ = + 91 (c 0.35, $CHCl_3$).

---

[8] Feng, J.; Wang, L.; Xue, X.; Chao, Z.; Hong, B.; Gu, Z. *Org. Lett.* **2021**, *23*, 8056–8061.

(S_p)-6-([2.2]Paracyclophan-4-yl)phenanthridine ((S_p)-9c).

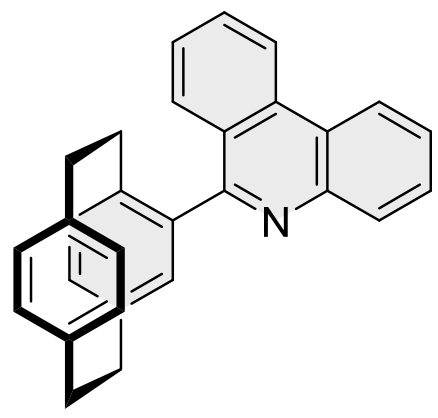

The title compound was prepared according to the above-mentioned procedure for preparation of ***rac*-9c** from (S_p)-4-formyl[2.2]paracyclophane (S_p)-**2** (236 mg, 1 mmol, 1 equiv.), 2-phenylaniline (508 mg, 3 mmol, 3 equiv.) and 2,2,2-trifluoroacetic acid (5 mL, 0.2 M). Column chromatography of the residue (80 g silica gel cartridge, flow rate: 60 mL/min, linear gradient: hexanes/CH_2Cl_2 4:1→3:1) gave 170 mg (44%) of the title product as a colorless solid.

Its spectral characteristics (^{1}H and ^{13}C NMR, IR, MS) were identical with those of ***rac*-9c**.

$[\alpha]_D^{20} =$ -89 (c 0.3, $CHCl_3$).

3.3 Synthesis of azabora-[2.2]paracyclophanes 1

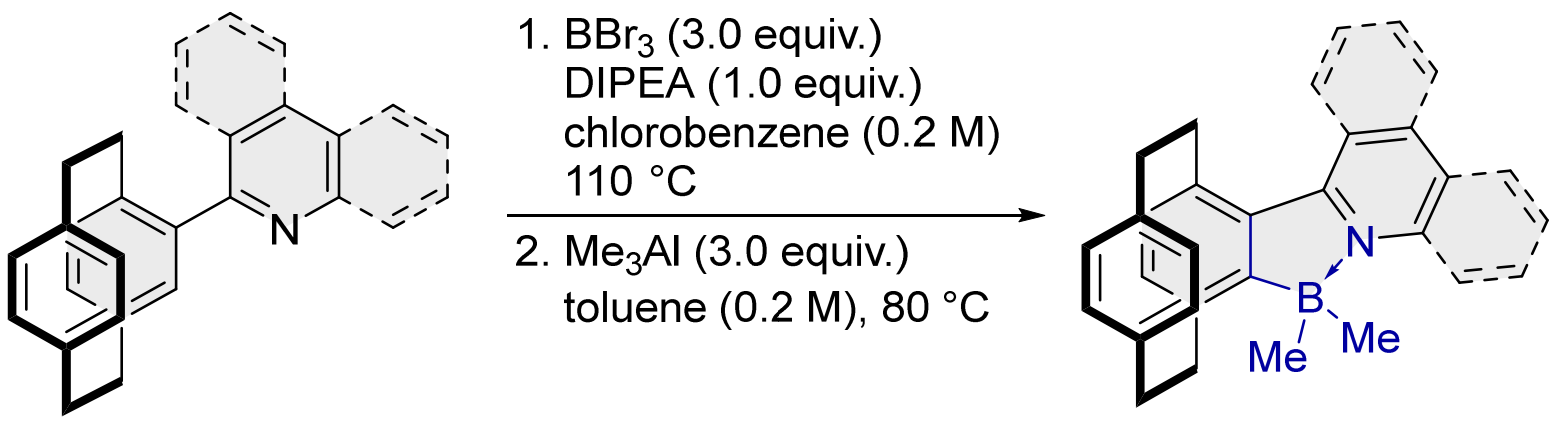

9a–c **1a–c**

General procedure 2 (GP2). Synthesis of azabora-[2.2]paracyclophanes 1a–c.

Into a microwave vial (10 mL size) was placed the corresponding [2.2]paracyclophanyl heteroaromatic compound **9a-c**, then it was flushed with argon, and its content was dissolved in anhydrous chlorobenzene (0.2 M). *N*,*N*-diisopropylethylamine (1.0 equiv.) was added to the solution followed by addition of BBr_3 (3.0 equiv.) at 0 °C. The reaction mixture was stirred at 110 °C (an aluminum block) for 16 hours. After cooling down to room temperature, the saturated aq. solution of Na_2CO_3 was added, and the organic layer was extracted with CH_2Cl_2 (3 × 20 mL). The combined organic phases were dried over anhydrous Na_2SO_4, filtered, and concentrated under reduced pressure. The crude product was used directly without further purification in the next reaction step. A flask charged with the crude product was flushed with argon and, toluene (0.2 M) was added. Then, trimethylaluminium (3.0 equiv.) was added to the slurry solution at 25 °C and the reaction mixture was stirred at 80 °C (oil bath) for 16 hours. Afterwards, water was added (20 mL) and, the organic layer was extracted with CH_2Cl_2 (3 × 20 mL). The combined organic phases were dried over anhydrous Na_2SO_4, filtered, and concentrated under reduced pressure. Column chromatography gave desired product.

***rac*-1^6,1^6-Dimethyl-1^6*H*-$1^5\lambda^4$,$1^6\lambda^4$-1(7,10)-$5\lambda^4$-benzo[3,4][1,2]azaborolo[1,5-*a*]pyridina-4(1,4)-benzenacyclohexaphane (*rac*-1a).**

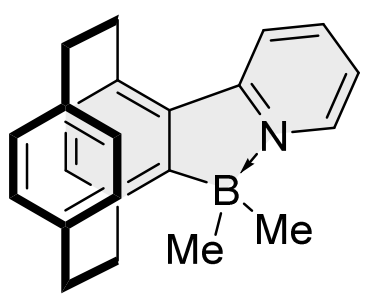

The title compound was prepared according to the general procedure **GP2**. The borylation was carried out with *rac*-**9a** (57 mg, 0.2 mmol, 1 equiv.), *N*,*N*-diisopropylethylamine (40 µL, 0.2 mmol, 1 equiv.) and BBr_3 (60 µL, 0.6 mmol, 3 equiv.) in chlorobenzene (1 mL, 0.2 M) at 0–110 °C for 16 hours. The methylation reaction was carried out with trimethylaluminium (0.3 mL, 2.0 M in hexanes, 0.6 mmol, 3 equiv.) in toluene (1 mL, 0.2 M) at 80 °C for 16 hours. Column chromatography of the residue (40 g silica gel cartridge,

flow rate: 40 mL/ min, linear gradient: hexanes/CH_2Cl_2 100:0→90:10→80:20) gave 25 mg (38%) of the title compound as a colorless solid.

R_f (hexanes/CH_2Cl_2 1:1) = 0.68

M.p. = 134.4–135.4 °C.

^{1}H NMR (400 MHz, $CDCl_3$) δ 8.36 (dd, J = 5.7, 1.5 Hz, 1H), 8.03 (d, J = 8.3 Hz, 1H), 7.91 (td, J = 7.8, 1.6 Hz, 1H), 7.28–7.24 (m, 1H, $CHCl_3$ interfering), 6.65 (qd, J = 7.8, 1.9 Hz, 2H), 6.48–6.33 (m, 3H), 6.01 (dd, J = 7.9, 1.9 Hz, 1H), 3.90–3.77 (m, 1H), 3.48–3.35 (m, 1H), 3.26–2.95 (m, 6H), 0.42 (s, 3H), -0.22 (s, 3H).

^{1}H NMR (400 MHz, C_6D_6) δ 7.86 (dt, J = 5.7, 1.3 Hz, 1H), 7.40 (d, J = 8.3 Hz, 1H), 6.92 (ddd, J = 8.6, 7.4, 1.6 Hz, 1H), 6.65 (d, J = 7.9 Hz, 1H), 6.57 (d, J = 1.2 Hz, 2H), 6.39 (d, J = 7.4 Hz, 1H), 6.31–6.24 (m, 2H), 5.95 (d, J = 7.9 Hz, 1H), 3.61 (ddd, J = 12.6, 10.5, 4.2 Hz, 1H), 3.51–3.42 (m, 1H), 3.19 (ddd, J = 13.4, 10.5, 3.4 Hz, 1H), 3.05 (ddd, J = 13.0, 10.6, 3.4 Hz, 1H), 2.91 (ttd, J = 13.6, 6.6, 3.4 Hz, 3H), 2.82–2.71 (m, 1H), 0.77 (s, 3H), 0.29 (s, 3H).

$^{13}C\{^1H\}$ NMR (101 MHz, $CDCl_3$) δ 157.4, 142.1, 141.5, 140.3, 139.2, 138.7, 135.23, 135.17, 134.9, 134.7, 132.5, 132.4, 132.3, 131.0, 120.4, 119.9, 35.5, 35.1, 34.8, 33.7.

^{11}B NMR (128 MHz, $CDCl_3$) δ -0.21.

HRMS (ESI) calcd for $C_{23}H_{25}BN$ $[M+H]^+$: 326.2075, found: 326.2072.

IR (ATR) ν_{max} 2912, 2848, 2821, 1612, 1479, 1458, 779, 756, 733, 715 cm^{-1}.

(*R*p)-1^6,1^6-Dimethyl-1^6*H*-1$^5\lambda^4$,1$^6\lambda^4$-1(7,10)-5λ^4-benzo[3,4][1,2]azaborolo[1,5-*a*]pyridina-4(1,4)-benzenacyclohexaphane ((*R*p)-1a).

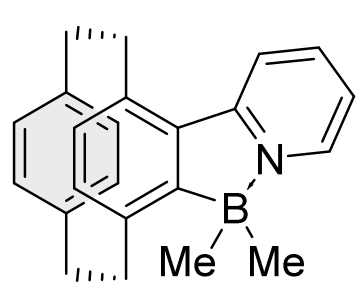

The title compound was prepared according to **GP2** from (*R*p)-**9a** (101 mg, 0.35 mmol, 1 equiv.), *N*,*N*-diisopropylethylamine (60 µL, 0.35mmol, 1 equiv.), BBr_3 (0.10 mL, 1.05 mmol, 3 equiv.) chlorobenzene (2 mL, 0.18 M), trimethylaluminium (0.53 mL, 2.0 M in hexanes, 1.05 mmol, 3 equiv.) and toluene (2 mL, 0.18 M). Column chromatography of the residue (40 g silica gel cartridge, flow rate: 40 mL/ min, linear gradient: hexanes/CH_2Cl_2 100:0→90:10→80:20) gave 41 mg (36%) of the title compound as a colorless solid.

Its spectral characteristics (^{1}H and ^{13}C NMR, IR, MS) were identical with those of ***rac*-1a**.

HPLC conditions: Daicel Chiralpak® IB, heptane/*i*-PrOH 99:1, 0.5 mL/min, I = 254 nm, t_{major} = 21.66 min, t_{minor} = 36.85 min, ee > 99%.

$[\alpha]_D^{20}$ = - 318 (c 0.5, CH_2Cl_2).

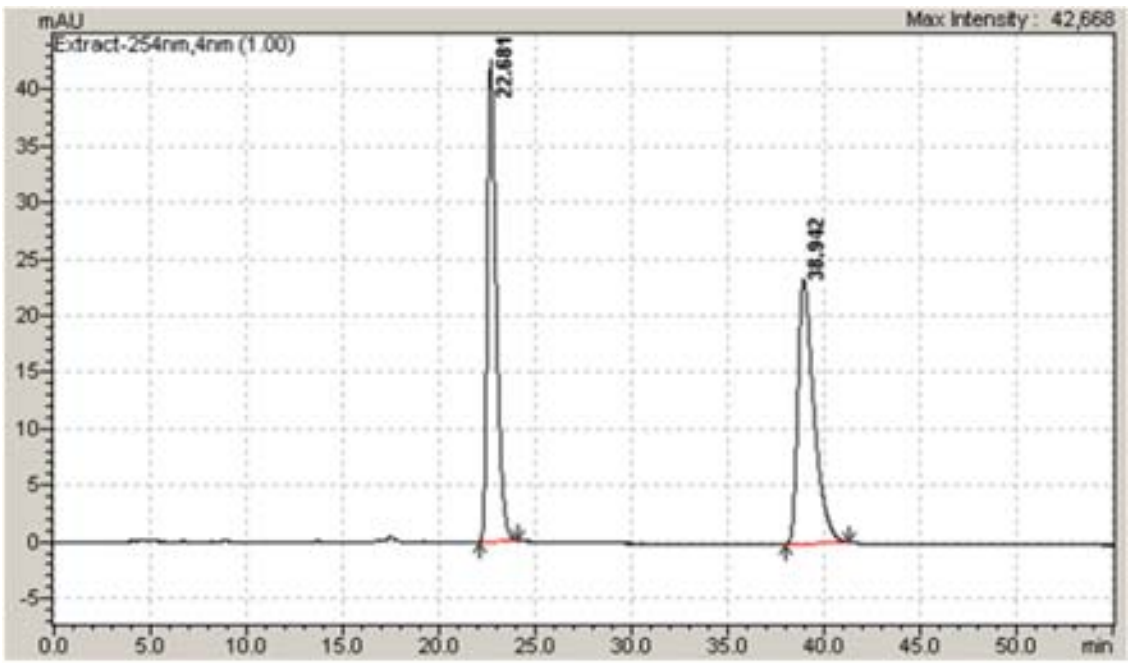

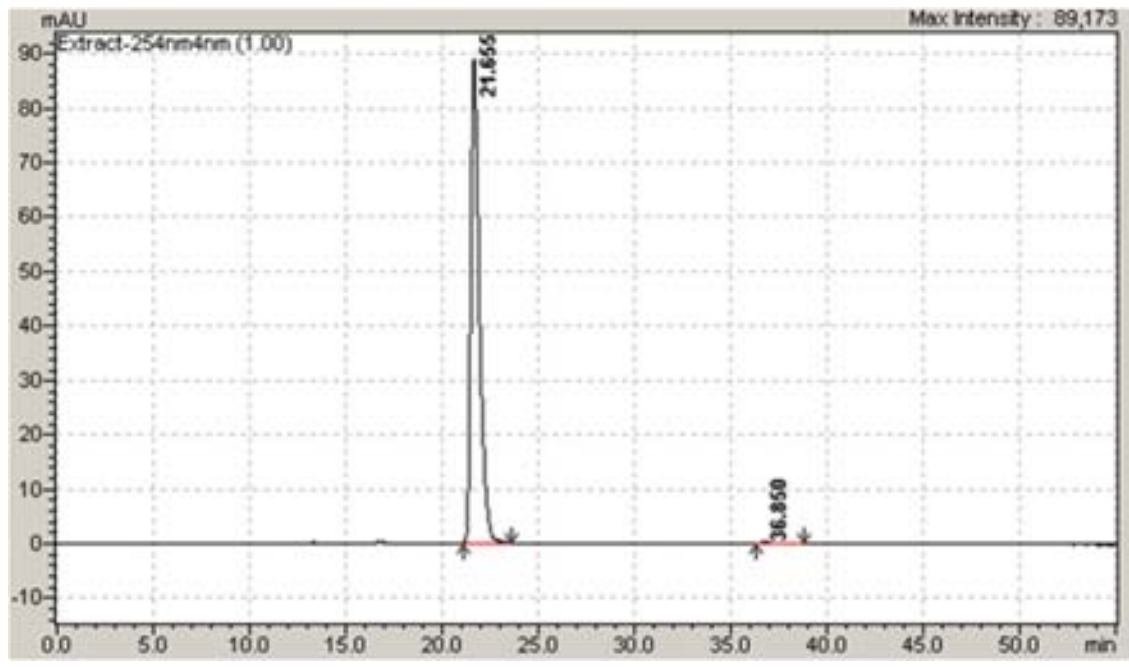

Compd	Retention time (min)	Area (%)	Compd	Retention t (min)	Area (%)
(R_p)-**1a**	22.68	50.3	(R_p)-**1a**	21.66	99.7
(S_p)-**1a**	38.94	49.7	(S_p)-**1a**	36.85	0.3

(S_p)-1^6,1^6-Dimethyl-1^6*H*-$1^5\lambda^4$,$1^6\lambda^4$-1(7,10)-5λ^4-benzo[3,4][1,2]azaborolo[1,5-*a*]pyridina-4(1,4)-benzenacyclohexaphane ((S_p)-1a).

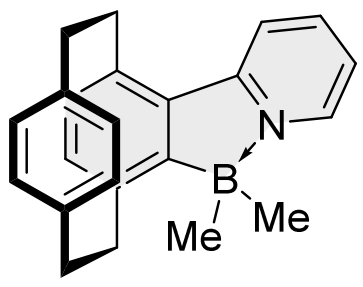

The title compound was prepared according to **GP2** from (S_p)-**9a** (174 mg, 0.61 mmol, 1 equiv.), *N*,*N*-diisopropylethylamine (0.11 mL, 0.61 mmol, 1 equiv.), BBr_3 (0.17 mL, 1.83 mmol, 3 equiv.) chlorobenzene (3 mL, 0.2 M), trimethylaluminium (0.92 mL, 2.0 M in hexanes, 1.83 mmol, 3 equiv.) and toluene (3 mL, 0.2 M). Column chromatography of the residue (40 g silica gel cartridge, flow rate: 40 mL/ min, linear gradient: hexanes/CH_2Cl_2 100:0→90:10→80:20) gave 74 mg (37%) of the title compound as a colorless solid.

Its spectral characteristics (^{1}H and ^{13}C NMR, IR, MS) were identical with those of ***rac*-1a**.

HPLC conditions: Daicel Chiralpak® IB, heptane/*i*-PrOH 99:1, 0.5 mL/min, I = 254 nm, t_{minor} = 22.41 min, t_{major} = 37.73 min, ee > 99%.

$[\alpha]_D^{20}$ = + 365 (c 0.3, CH_2Cl_2).

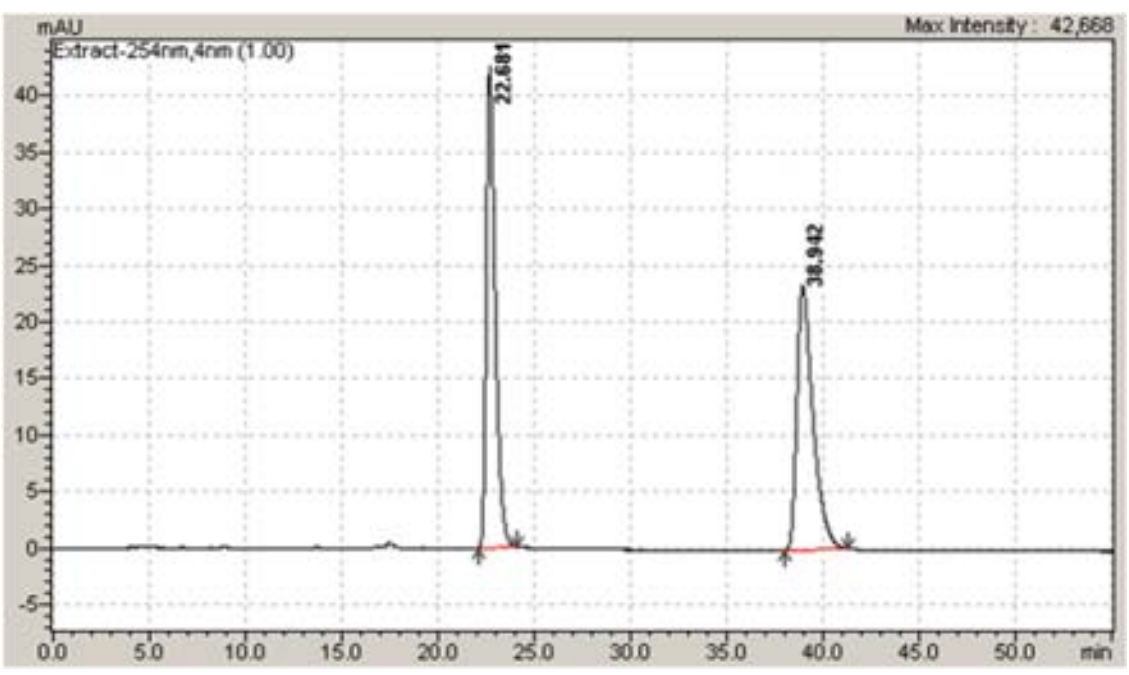

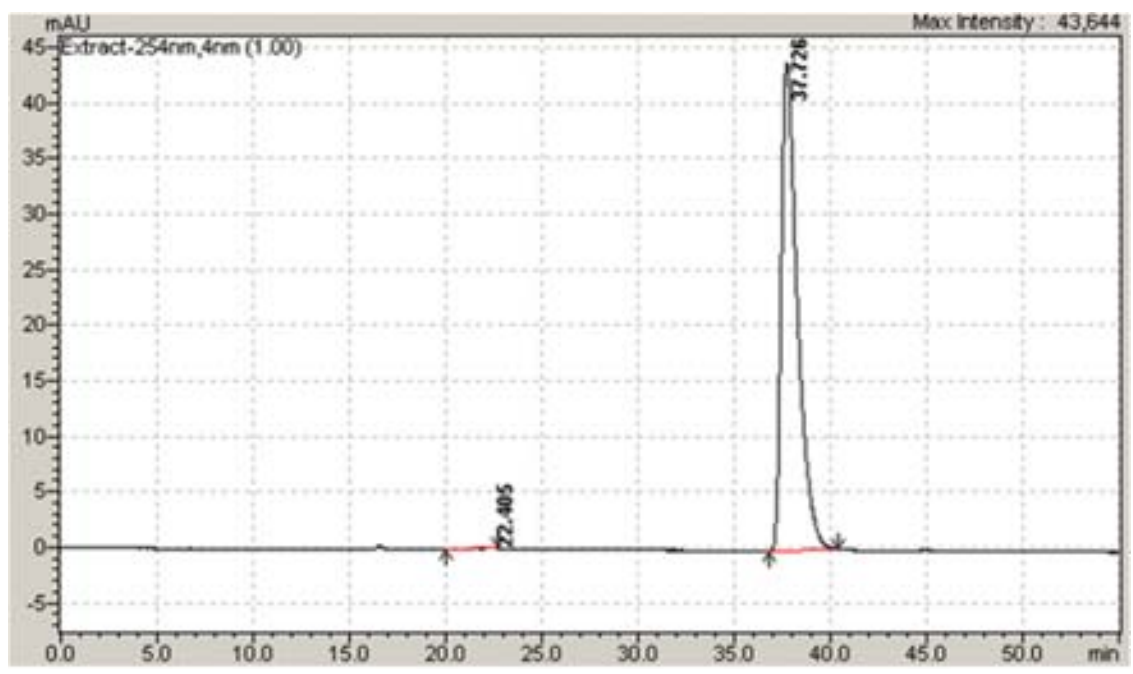

Compd	Retention t (min)	Area (%)
(R_p)-**1a**	22.68	50.3
(S_p)-**1a**	38.94	49.7

Compd	Retention t (min)	Area (%)
(R_p)-**1a**	22.41	0.1
(S_p)-**1a**	37.73	99.9

***rac*-1^8,1^8-Dimethyl-1^8*H*-1$^7\lambda^4$,1$^8\lambda^4$-1(9,12)-7λ^4-benzo[3,4][1,2]azaborolo[5,1-*a*]isoquinolina-4(1,4)-benzenacyclohexaphane (*rac*-1b).**

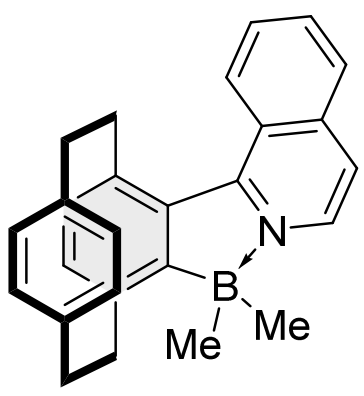

The title compound was prepared according to **GP2**. The borylation reaction was carried out with *rac*-**9b** (67 mg, 0.2 mmol, 1 equiv.), *N*,*N*-diisopropylethylamine (40 µL, 0.2 mmol, 1 equiv.) and BBr_3 (60 µL, 0.6 mmol, 3 equiv.) in chlorobenzene (1 mL, 0.2 M) at 110 °C for 16 hours. The methylation reaction was carried out with trimethylaluminium (0.3 mL, 2.0 M in hexanes, 0.6 mmol, 3 equiv.) in toluene (1 mL, 0.2 M) at 80 °C for 16 hours. Column chromatography of the residue (40 g silica gel cartridge, flow rate: 40 mL/min, linear gradient: hexanes/CH_2Cl_2 100:0→90:10→80:20) gave 45 mg (60%) of the title compound as a yellowish solid.

R_f (hexanes/CH_2Cl_2 1:1) = 0.66.

M.p. = 233.1–234.0 °C.

^{1}H NMR (400 MHz, $CDCl_3$) δ 8.53 (d, J = 8.5 Hz, 1H), 8.23 (d, J = 6.3 Hz, 1H), 7.91 (d, J = 8.2 Hz, 1H), 7.84–7.76 (m, 1H), 7.73–7.65 (m, 1H), 7.63 (d, J = 6.3 Hz, 1H), 6.84–6.70 (m, 2H), 6.51 (d, J = 7.4 Hz, 1H), 6.43 (d, J = 7.5 Hz, 2H), 5.90 (dt, J = 8.0, 1.2 Hz, 1H), 3.73–3.60 (m, 1H), 3.55–3.46 (m, 1H), 3.31–2.99 (m, 5H), 2.92–2.82 (m, 1H), 0.57 (s, 3H), -0.19 (s, 3H).

^{13}C{^{1}H} NMR (101 MHz, $CDCl_3$) δ 157.0, 140.1, 139.5, 138.9, 137.9, 136.6, 136.3, 135.4, 134.6, 133.7, 132.8, 132.6, 131.7, 131.2, 130.6, 130.0, 127.5, 127.0, 124.6, 119.0, 37.0, 36.1, 35.2, 34.1.

^{11}B NMR (128 MHz, $CDCl_3$) δ 0.13.

HRMS (ESI) calcd for $C_{27}H_{27}BN$ [M+H]$^+$: 376.2231, found: 376.2229.

IR (ATR) ν_{max} 2912, 2897, 2752, 2819, 1425, 1404, 1352, 814, 795, 746, 671 cm^{-1}.

(R_p)-1^8,1^8-Dimethyl-1^8*H*-1$^7\lambda^4$,1$^8\lambda^4$-1(9,12)-7λ^4-benzo[3,4][1,2]azaborolo[5,1-*a*]isoquinolina-4(1,4)-benzenacyclohexaphane ((R_p)-1b).

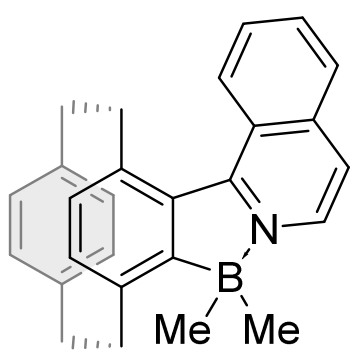

The title compound was prepared according to **GP2** from (R_p)-**9b** (53 mg, 0.16 mmol, 1 equiv.), *N*,*N*-diisopropylethylamine (30 µL, 0.17 mmol, 1 equiv.), BBr_3 (50 µL, 0.5 mmol, 3 equiv.) chlorobenzene (1 mL, 0.16 M), trimethylaluminium

(0.25 mL, 2.0 M in hexanes, 0.5 mmol, 3 equiv.) and toluene (1 mL, 0.16 M). Column chromatography of the residue (40 g silica gel cartridge, flow rate: 40 mL/min, linear gradient: hexanes/CH_2Cl_2 100:0→90:10→80:20) gave 28 mg (47%) of the title compound as a yellowish solid.

Its spectral characteristics (^{1}H and ^{13}C NMR, IR, MS) were identical with those of ***rac*-1b**.

HPLC conditions: Daicel Chiralpak® IB, heptane/*i*-PrOH 99:1, 0.3 mL/min, I = 254 nm, t_{major} = 24.68 min, t_{minor} = 28.99 min, ee > 99%.

$[\alpha]_D^{20}$ = + 85 (c 0.3, CH_2Cl_2).

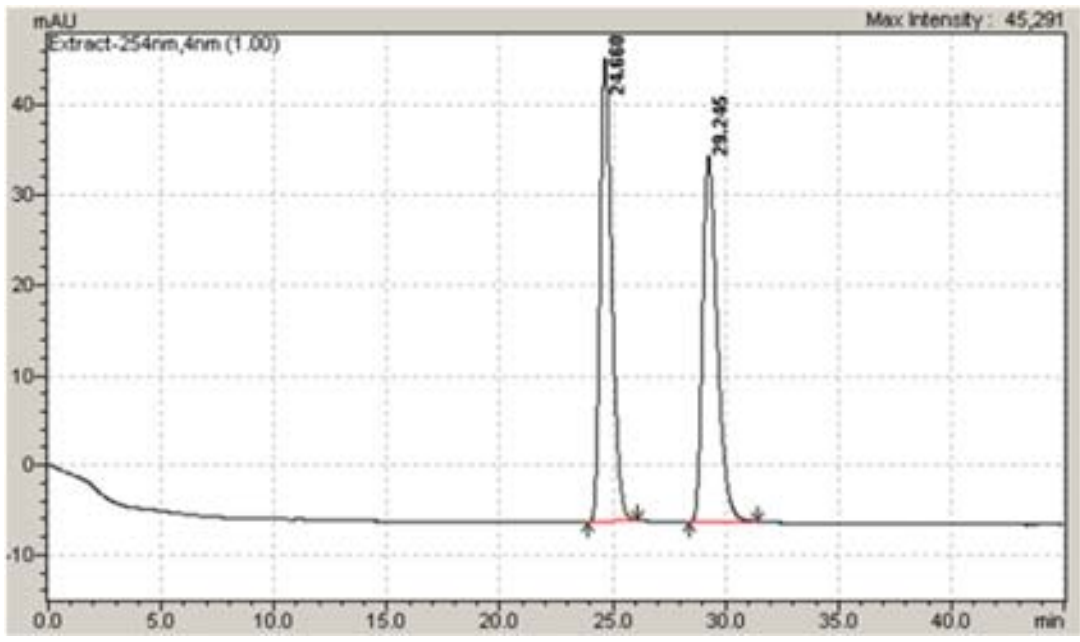

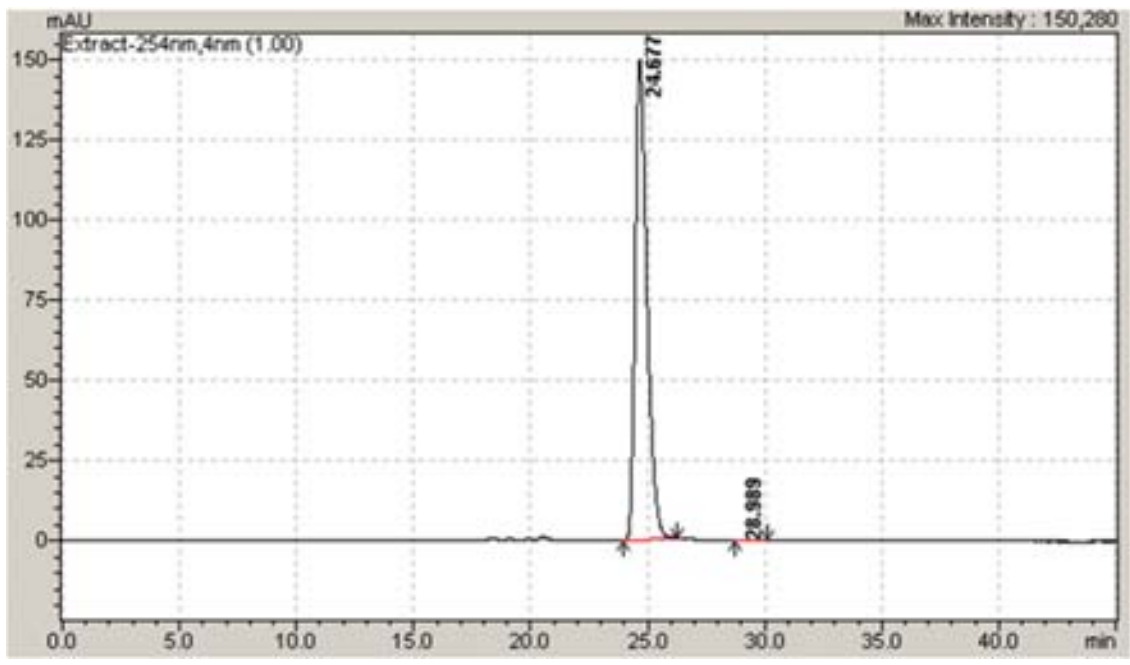

Compd	Retention time (min)	Area (%)	Compd	Retention t (min)	Area (%)
(R_p)-**1b**	24.66	50.1	(R_p)-**1b**	24.68	99.9
(S_p)-**1b**	29.25	49.9	(S_p)-**1b**	28.99	0.1

(S_p)-1^8,1^8-Dimethyl-1^8*H*-1^{7}λ^4,1^{8}λ^4-1(9,12)-7λ^4-benzo[3,4][1,2]azaborolo[5,1-*a*]isoquinolina-4(1,4)-benzenacyclohexaphane ((*S*p)-1b).

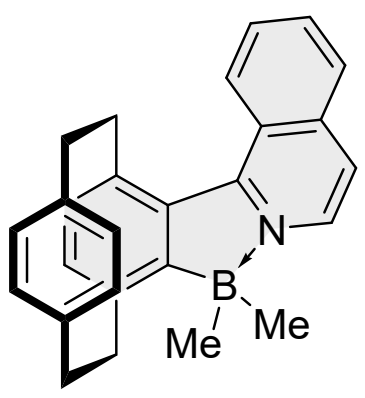

The title compound was prepared according to **GP2** from (S_p)-**9b** (122 mg, 0.36 mmol, 1 equiv.), *N*,*N*-diisopropylethylamine (60 µL, 0.34 mmol, 1 equiv.), BBr_3 (0.1 mL, 1.1 mmol, 3 equiv.) chlorobenzene (2 mL, 0.18 M), trimethylaluminium (0.54 mL, 2.0 M in hexanes, 1.08 mmol, 3 equiv.) and toluene (2 mL, 0.18 M). Column chromatography of the residue (40 g silica gel cartridge, flow rate: 40 mL/min, linear gradient: hexanes/CH_2Cl_2 100:0→90:10→80:20) gave 73 mg (53%) of the title compound as a yellowish solid.

Its spectral characteristics (^{1}H and ^{13}C NMR, IR, MS) were identical with those of ***rac*-1b**.

HPLC conditions: Daicel Chiralpak® IB, heptane/*i*-PrOH 99:1, 0.3 mL/min, I = 254 nm, t_{minor} = 25.67 min, t_{major} = 30.44 min, ee = 99%.

$[\alpha]_D^{20}$ = - 89 (c 0.3, CH_2Cl_2).

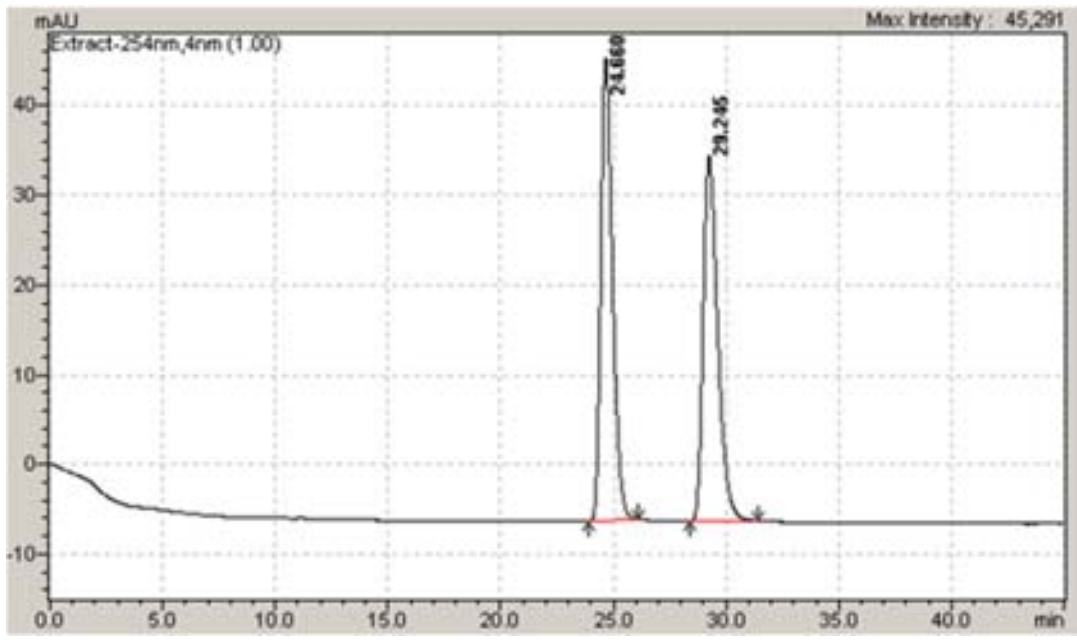

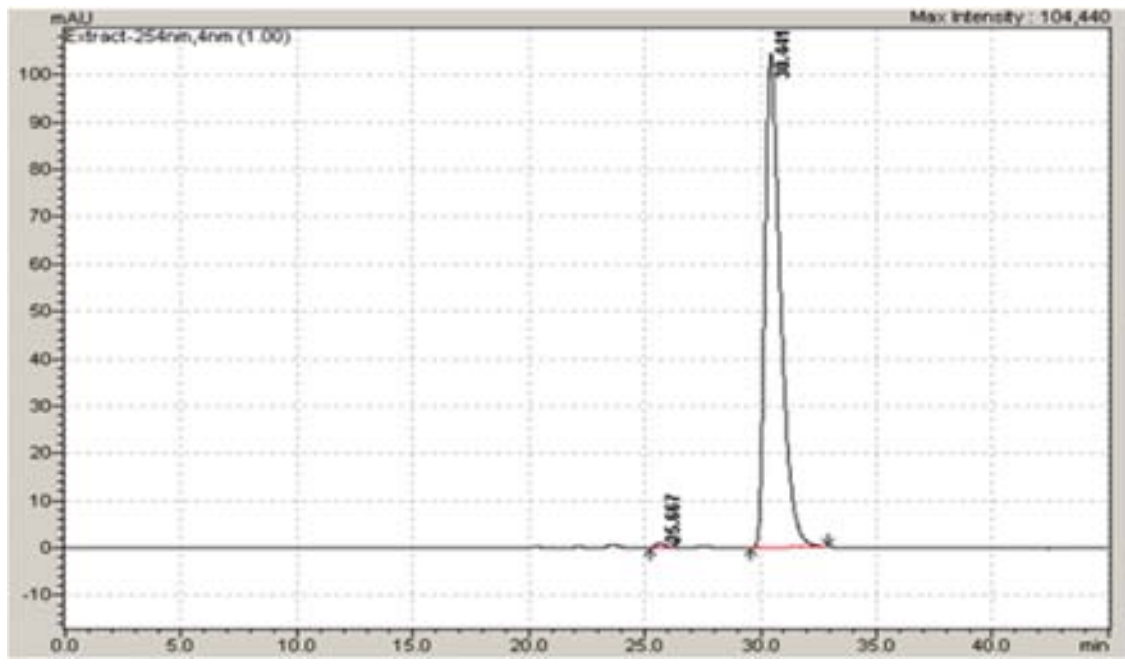

Compd	Retention time (min)	Area (%)	Compd	Retention t (min)	Area (%)
(R_p)-**1b**	24.66	50.1	(R_p)-**1b**	25.67	0.6
(S_p)-**1b**	29.25	49.9	(S_p)-**1b**	30.44	99.4

***rac*-1^{10},1^{10}-Dimethyl-1^{10}*H*-1^{9}λ^{4},1^{10}λ^{4}-1(11,14)-9λ^{4}-benzo[3,4][1,2]azaborolo[1,5-*f*]phenanthridina-4(1,4)-benzenacyclohexaphane (*rac*-1c).**

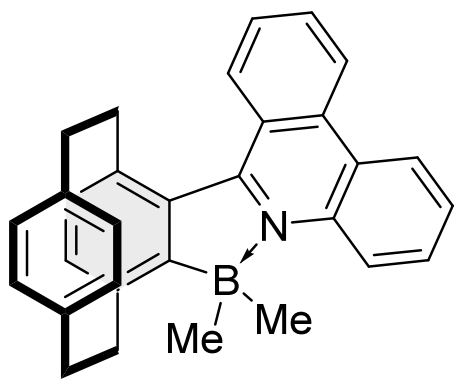

The title compound was prepared according to **GP2**. The borylation reaction was carried out with *rac*-**9c** (30 mg, 0.1 mmol, 1 equiv.), *N*,*N*-diisopropylethylamine (20 µL, 0.1 mmol, 1 equiv.) and BBr_3 (30 µL, 0.3 mmol, 3 equiv.) in chlorobenzene (0.5 mL, 0.2 M) at 110 °C for 16 hours. The methylation reaction was carried out with trimethylaluminium (0.15 mL, 2.0 M in hexanes, 0.3 mmol, 3 equiv.) in toluene (0.5 mL, 0.2 M) at 80 °C for 16 hours. Column chromatography of the residue (40 g silica gel cartridge, flow rate: 40 mL/ min, linear gradient: hexanes/CH_2Cl_2 100:0→90:10→80:20) gave 19 mg (57%) of the title compound as a yellow solid.

R_f (hexanes/CH_2Cl_2 4:1) = 0.20.

M.p. = 211.9–212.9 °C.

^{1}H NMR (400 MHz, $CDCl_3$) δ 8.76 (d, J = 8.6 Hz, 1H), 8.72–8.60 (m, 3H), 7.93 (ddd, J = 8.3, 6.9, 1.3 Hz, 1H), 7.82 (ddd, J = 8.6, 7.0, 1.5 Hz, 1H), 7.70 (dddd, J = 9.9, 8.1, 6.9, 1.2 Hz, 2H), 6.86–6.72 (m, 2H), 6.56–6.42 (m, 3H), 5.90 (dd, J = 8.0, 1.9 Hz, 1H), 3.70–3.59 (m, 2H), 3.31–3.04 (m, 5H), 2.94–2.80 (m, 1H), 0.82 (s, 3H), 0.13 (s, 3H).

^{13}C{^{1}H} NMR (101 MHz, $CDCl_3$) δ 158.7, 139.9, 139.0, 138.9, 138.0, 137.2, 137.1, 135.2, 134.7, 134.6, 132.7, 132.4, 132.3, 132.1, 131.3, 130.7, 129.4, 127.2, 126.3, 124.32, 124.27, 123.3, 122.8, 122.3, 37.2, 35.9, 35.2, 33.6.

^{11}B NMR (128 MHz, $CDCl_3$) δ 2.40.

HRMS (APCI) calcd for $C_{31}H_{27}BN$ $[M-H]^-$: 424.2242, found: 424.2238.

IR (ATR) ν_{max} 2947, 2933, 2914, 2848, 1491, 1410, 1379, 758, 744, 727, 715, 704 cm^{-1}.

(*R*p)-1^{10},1^{10}-Dimethyl-1^{10}H-1$^{9}\lambda^{4}$,1$^{10}\lambda^{4}$-1(11,14)-9λ^{4}-benzo[3,4][1,2]azaborolo[1,5-f]phenanthridina-4(1,4)-benzenacyclohexaphane ((*R*p)-1c).

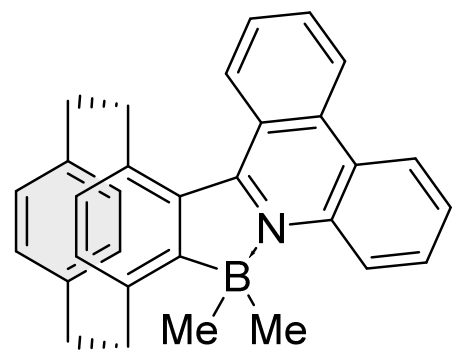

The title compound was prepared according to **GP2** from (*R*p)-**9c** (78 mg, 0.2 mmol, 1 equiv.), *N*,*N*-diisopropylethylamine (40 µL, 0.2 mmol, 1 equiv.), BBr_3 (60 µL, 0.6 mmol, 3 equiv.) chlorobenzene (1 mL, 0.2 M), trimethylaluminium (0.3 mL, 2.0 M in hexanes, 0.6 mmol, 3 equiv.) and toluene (1 mL, 0.2 M). Column chromatography of the residue (40 g silica gel cartridge, flow rate: 40 mL/ min, linear gradient: hexanes/CH_2Cl_2 100:0→90:10→80:20) gave 38 mg (44%) of the title product as a yellow solid.

Its spectral characteristics (^{1}H and ^{13}C NMR, IR, MS) were identical with those of ***rac*-1c**.

HPLC conditions: Daicel Chiralpak® IB, heptane/*i*-PrOH 99:1, 0.3 mL/min, I = 254 nm, t_{major} = 25.94 min, t_{minor} = 28.86 min, ee = 99%.

$[\alpha]_D^{20}$ = - 9 (*c* 0.35, CH_2Cl_2).

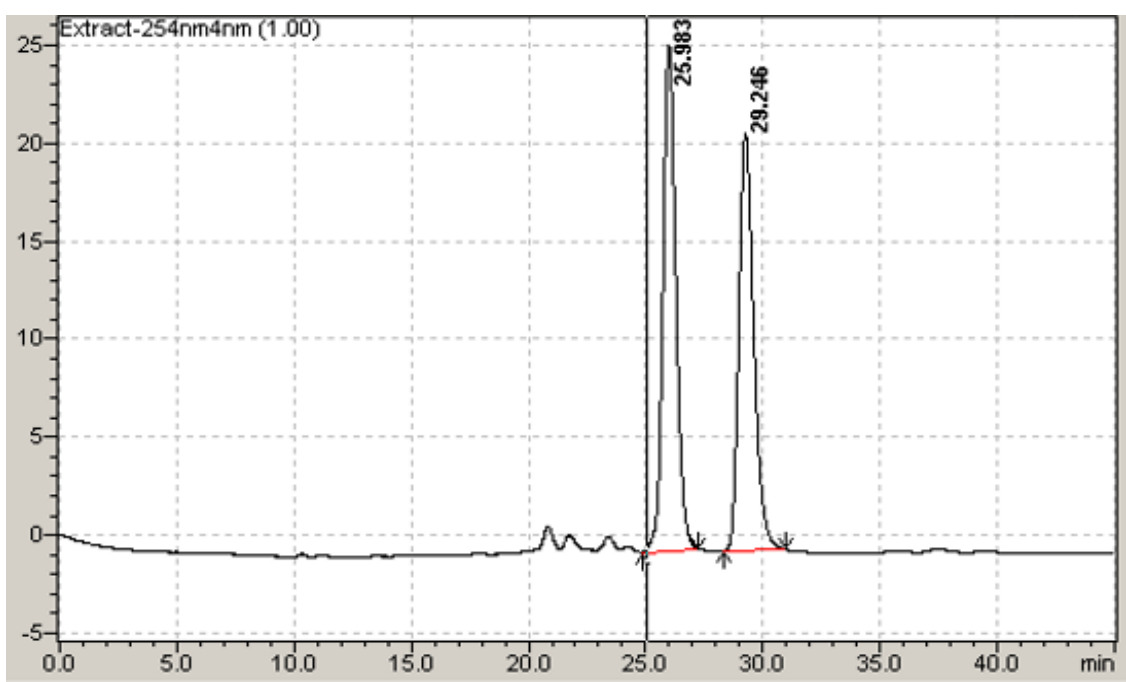

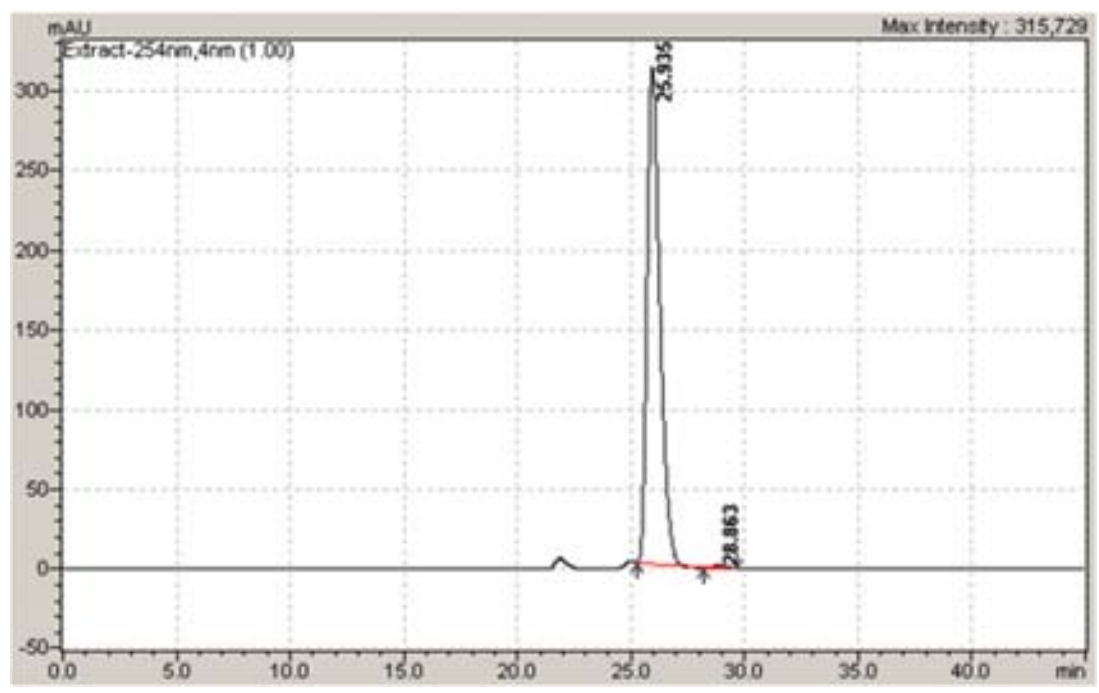

	Retention time (min)	Area (%)	Compd	Retention t (min)	Area (%)
(*R*p)-**1c**	25.98	50.1	(*R*p)-**1c**	25.94	99.4
(*S*p)-**1c**	29.25	49.9	(*S*p)-**1c**	28.86	0.6

(*S*p)-1^{10},1^{10}-Dimethyl-1^{10}H-1$^{9}\lambda^{4}$,1$^{10}\lambda^{4}$-1(11,14)-9λ^{4}-benzo[3,4][1,2]azaborolo[1,5-f]phenanthridina-4(1,4)-benzenacyclohexaphane ((*S*p)-1c)

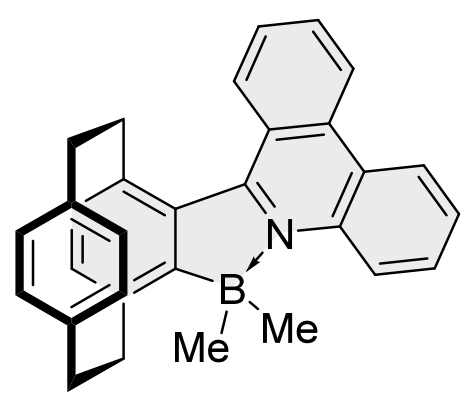

The title compound was prepared according to **GP2** from (*S*p)-**9c** (77 mg, 0.2 mmol, 1 equiv.), *N*,*N*-diisopropylethylamine (40 µL, 0.2 mmol, 1 equiv.), BBr_3 (60 µL, 0.6 mmol, 3 equiv.) chlorobenzene (1 mL, 0.2 M), trimethylaluminium (0.3 mL, 2.0 M in hexanes, 0.6 mmol, 3 equiv.) and

toluene (1 mL, 0.2 M). Column chromatography of the residue (40 g silica gel cartridge, flow rate: 40 mL/ min, linear gradient: hexanes/CH_2Cl_2 100:0→90:10→80:20) gave 34 mg (40%) of the title compound as a yellow solid.

Its spectral characteristics (^{1}H and ^{13}C NMR, IR, MS) were identical with those of ***rac*-1c**.

HPLC conditions: Daicel Chiralpak® IB, heptane/*i*-PrOH 99:1, 0.3 mL/min, I = 254 nm, t_{minor} = 26.67 min, t_{major} = 29.85 min, ee > 99%.

$[\alpha]_D^{20} = +\,12$ (c 0.3, CH_2Cl_2).

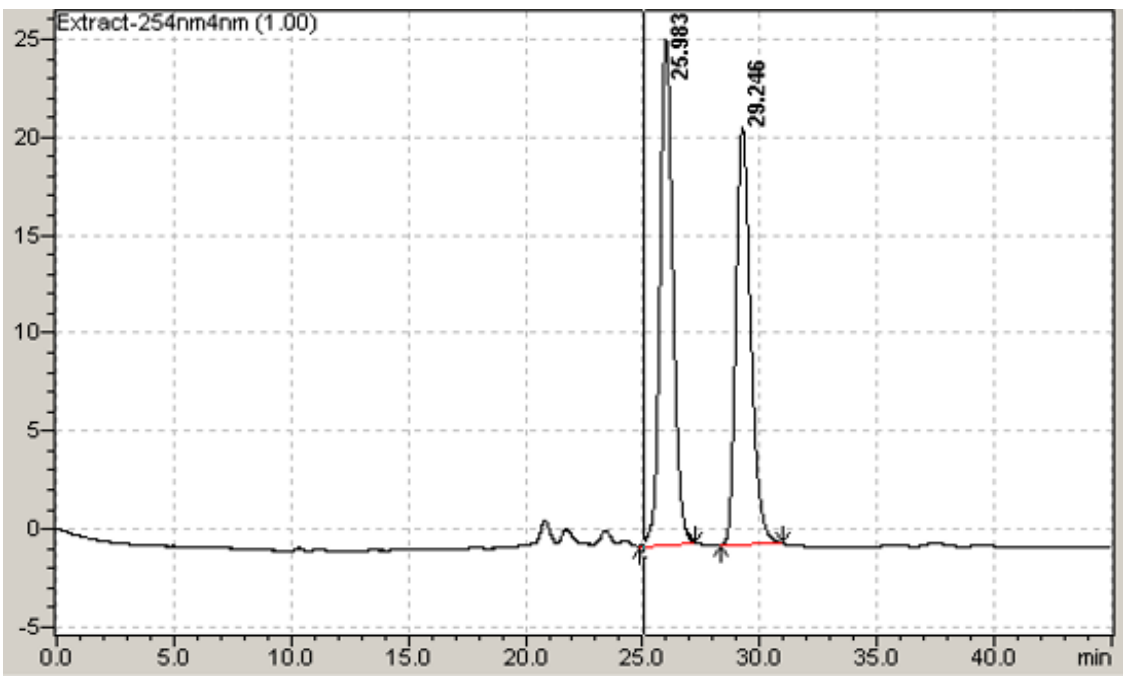

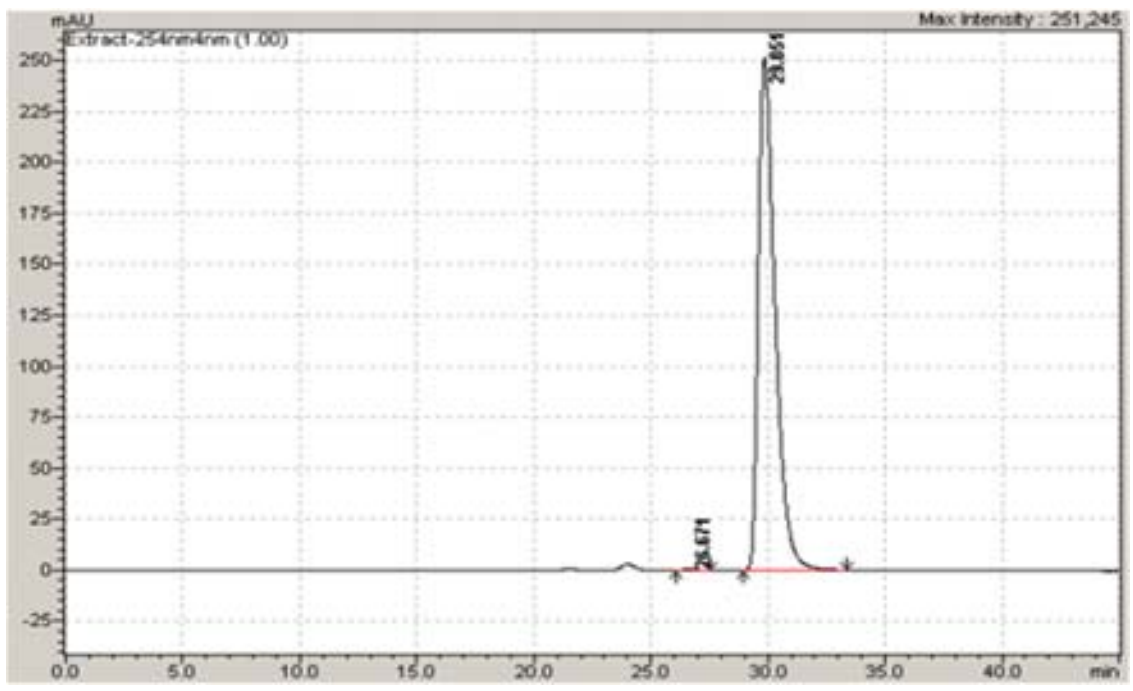

Compd	Retention t (min)	Area (%)
(R_p)-**1c**	25.98	50.1
(S_p)-**1c**	29.25	49.9

Compd	Retention t (min)	Area (%)
(R_p)-**1c**	26.67	0.2
(S_p)-**1c**	29.85	99.8

4 Crystallographic data

The diffraction experiments of **1a**, **1b**, **1c**, and **9c** crystals were performed on Bruker D8 VENTURE Kappa Duo PHOTONIII by IµS micro-focus sealed tube with CuKα (λ= 1.54178Å) radiation at low temperature of the crystal preserved by Cryostream Cooler 800 (Cryostream Cooler 1000 for **1a**). The structures were solved by direct methods (XT[9]) and refined by full matrix least squares based on F^2 (SHELXL2019[10]). The hydrogen atoms on carbon were fixed into idealized positions (riding model) and assigned temperature factors either $H_{iso}(H) = 1.2\ U_{eq}$(pivot atom) or 1.5 U_{eq}(pivot atom) for methyl moiety.

Quality of the most of crystals enable standard procedure for structure determination issuing a good precision of structural parameter. However, single crystal of **9c** cracks under flash cooling to 100K and its measurement was possible to be performed at 210K. The reason of cracking of crystal is probably disorder of whole phenanthridine moiety. Crystallographic data with CCDC numbers are summarized in Table S3.

X-ray crystallographic data have been deposited with the Cambridge Crystallographic Data Centre (CCDC), viz, Table S3 and can be obtained free of charge from the Centre via its website (www.ccdc.cam.ac.uk/structures/).

Crystals suitable for single crystal X-ray diffraction analysis were obtained by slow evaporation from either a DCM/hexane solution (**1a**) or DCM solutions (**1b**, **1c**, **9c**). **1a** and **1c** crystallized in the $P2_1/c$ space group, whereas **1b** in *Pbca*.

Table S3. Crystal data, data collection, and refinement parameters for **1a–c**, and **9c**.

[9] Sheldrick, G. M. *Acta Cryst*. **2015**, *A71*, 3–8.
[10] Sheldrick, G. M. *Acta Cryst.* **2015**, *C71*, 3–8.

**Compound**	**1a**	**1b**	**1c**	**9c**
CCDC	2525994	2525993	2525991	2525992
Formula	$C_{23}H_{24}BN$	$C_{27}H_{26}BN$	$C_{31}H_{28}BN$	$C_{29}H_{23}N$
M.w.	325.24	375.3	425.35	385.48
Crystal system	Monoclinic	Orthorhombic	Monoclinic	Monoclinic
Space group	$P2_1/c$ (No.14)	*Pbca* (No.61)	$P2_1$/c (No.14)	$P2_1/n$ (No. 14)
a [Å]	8.9735 (3)	19.8693 (10)	9.2468 (2)	8.5789 (2)
b [Å]	33.6269 (11)	9.7529 (5)	11.2411 (3)	13.8607 (3)
c [Å]	11.9935 (4)	20.447 (1)	21.8112 (6)	17.0944 (3)
α [°]				
β [°]	96.305 (2)		99.778 (1)	92.238 (1)
γ [°]				
Z	8	8	4	4
$V [Å^3]$	3597.2 (2)	3962.3 (3)	2234.21 (10)	2031.14 (7)
Temperature	100	120	120	210
D_x [g cm^{-3}]	1.201	1.258	1.265	1.261
Wavelength, Å	1.54178	1.54178	1.54178	1.54178
Crystal size [mm]	0.27 × 0.15 × 0.15	0.55 × 0.38 × 0.14	0.24 × 0.23 × 0.09	0.33 × 0.21 × 0.17
Crystal color, shape	Irregular, colourless	Prism, colourless	Plate, yellow	Prism, yellow
μ [mm^{-1}]	0.51	0.54	0.54	0.55
T_{min},T_{max}	0.873, 0.928	0.795, 0.929	0.822, 0.953	0.834, 0.913
Measured reflections	65018	55289	30041	40238
Independent diffractions (R_{int}^{a})	7759, (0.029)	3618, (0.021)	4564, (0.028)	4151, (0.028)
Observed diffract. [I>2σ(I)]	7261	3600	4284	3699
No. of parameters	455	264	300	348
R^{b}	0.052	0.037	0.036	0.050
$wR(F^2)$ for all data	0.135	0.091	0.096	0.135
GOF^{c}	1.02	1.07	1.04	1.06
Residual electron density [e/Å^3]	0.55, -0.58	0.25, -0.20	0.28, −0.19	0.21, −0.26

$^{a}R_{int} = \Sigma|F_o^2 - F_{o,mean}^2|/\Sigma F_o^2$; $^{b}R(F) = \Sigma||F_o| - |F_c||/\Sigma|F_o|$; $wR(F^2) = [\Sigma(w(F_o^2 - F_c^2)^2)/(\Sigma w(F_o^2)^2)]^{1/2}$;
$^{c}GOF = [\Sigma(w(F_o^2 - F_c^2)^2)/(N_{diffrs} - N_{params})]^{1/2}$

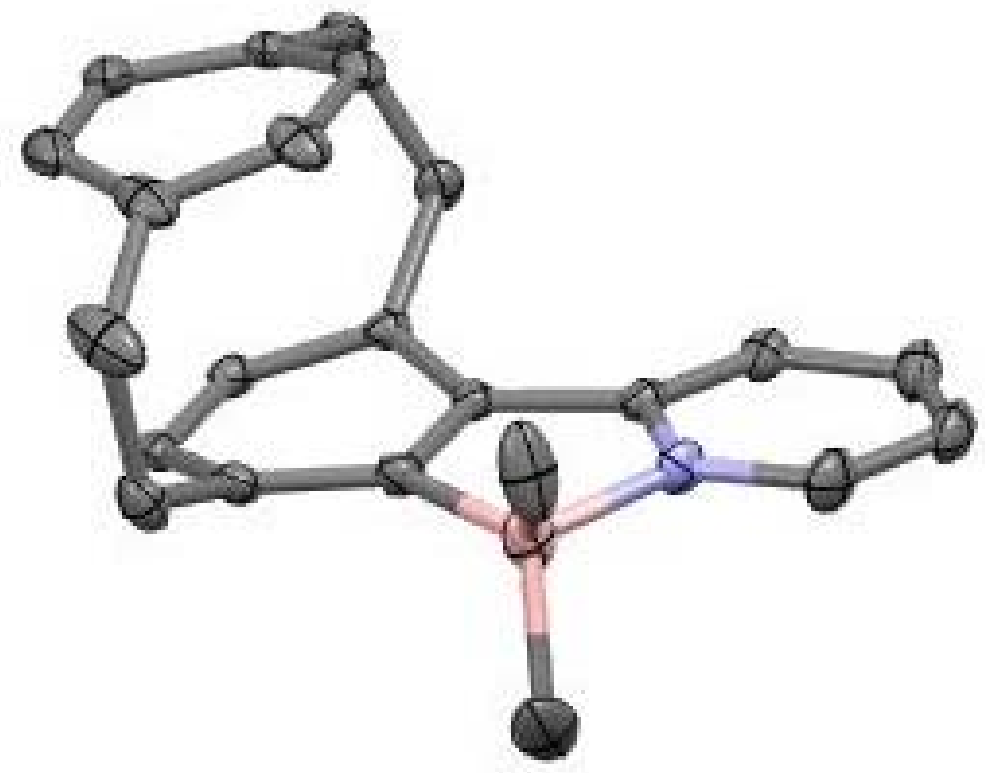

Figure S1. ORTEP drawing of **1a**. Ellipsoids are drawn with 20% probability.

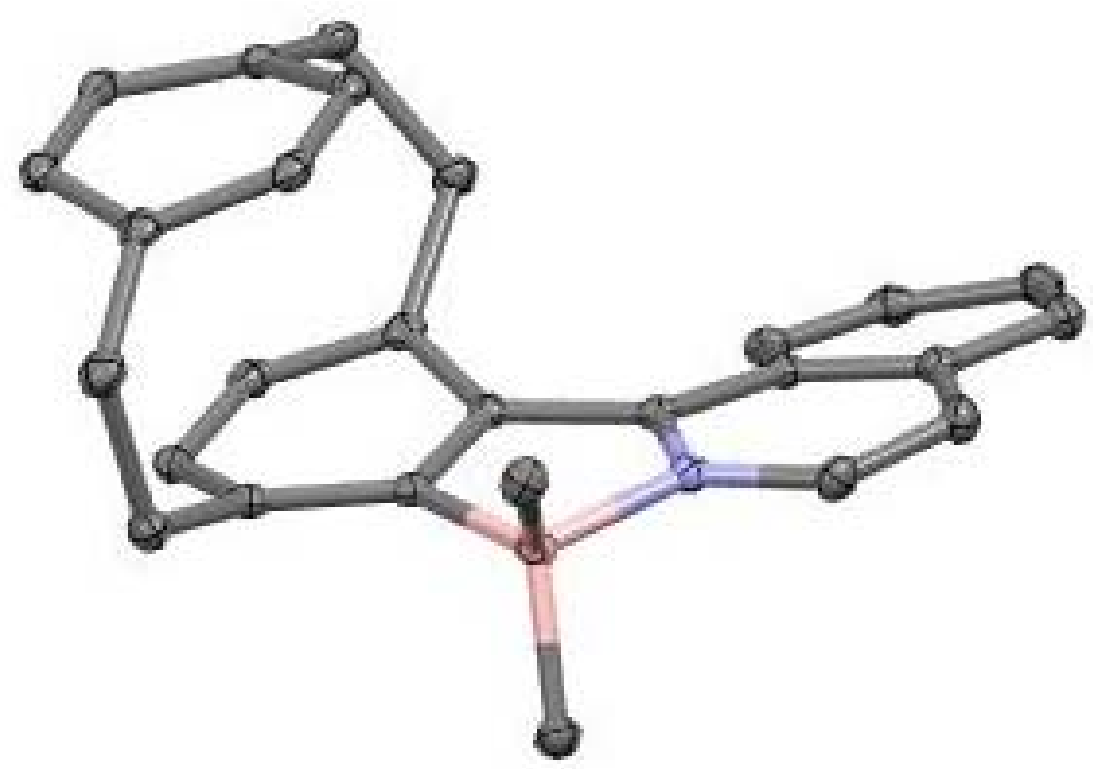

Figure S2. ORTEP drawing of **1b**. Ellipsoids are drawn with 20% probability.

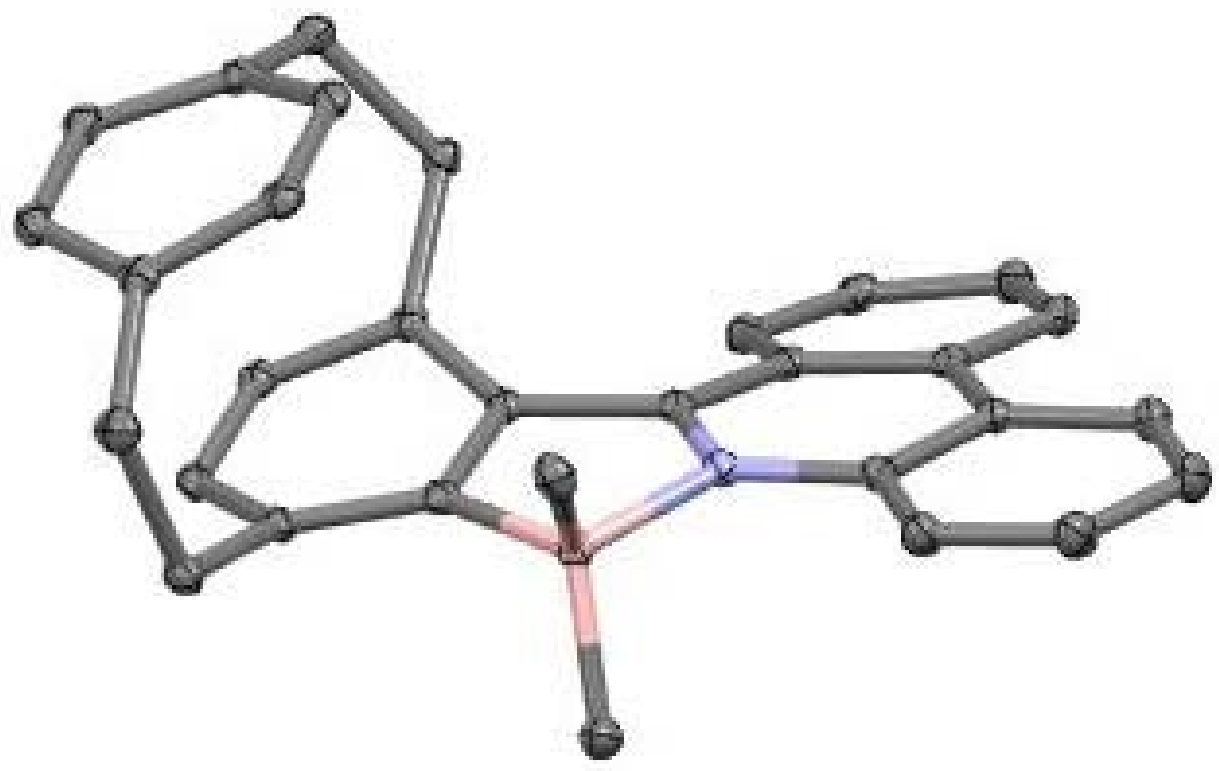

Figure S3. ORTEP drawing of **1c**. Ellipsoids are drawn with 20% probability.

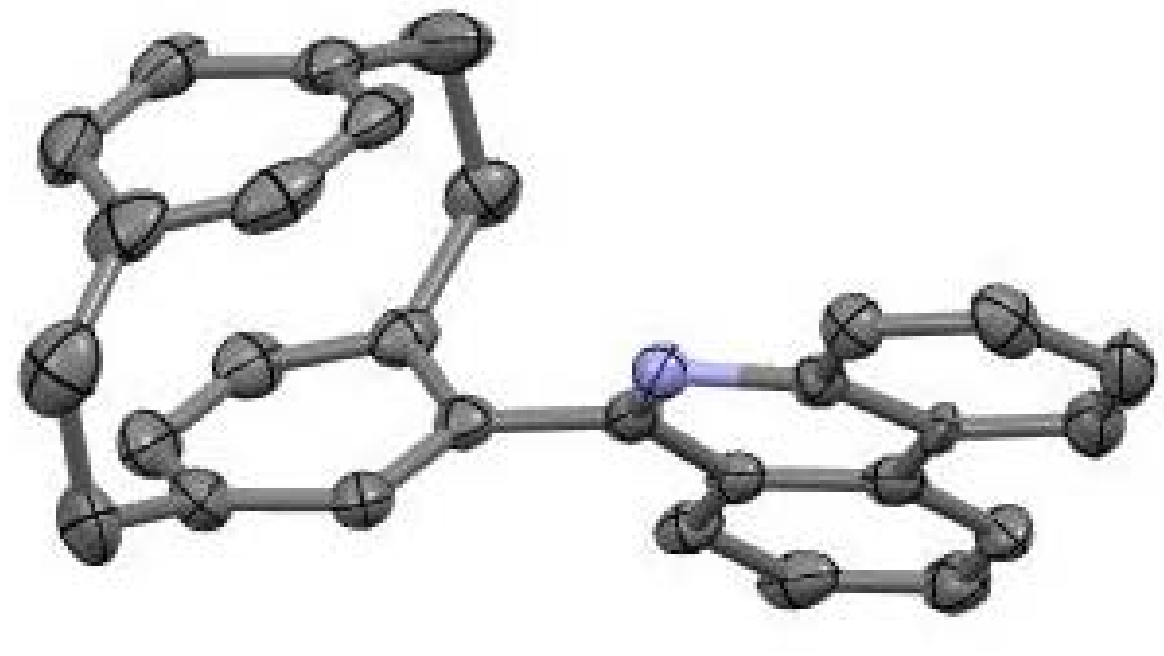

Figure S4. ORTEP drawing of **9c**. Ellipsoid are drawn with 20% probability.

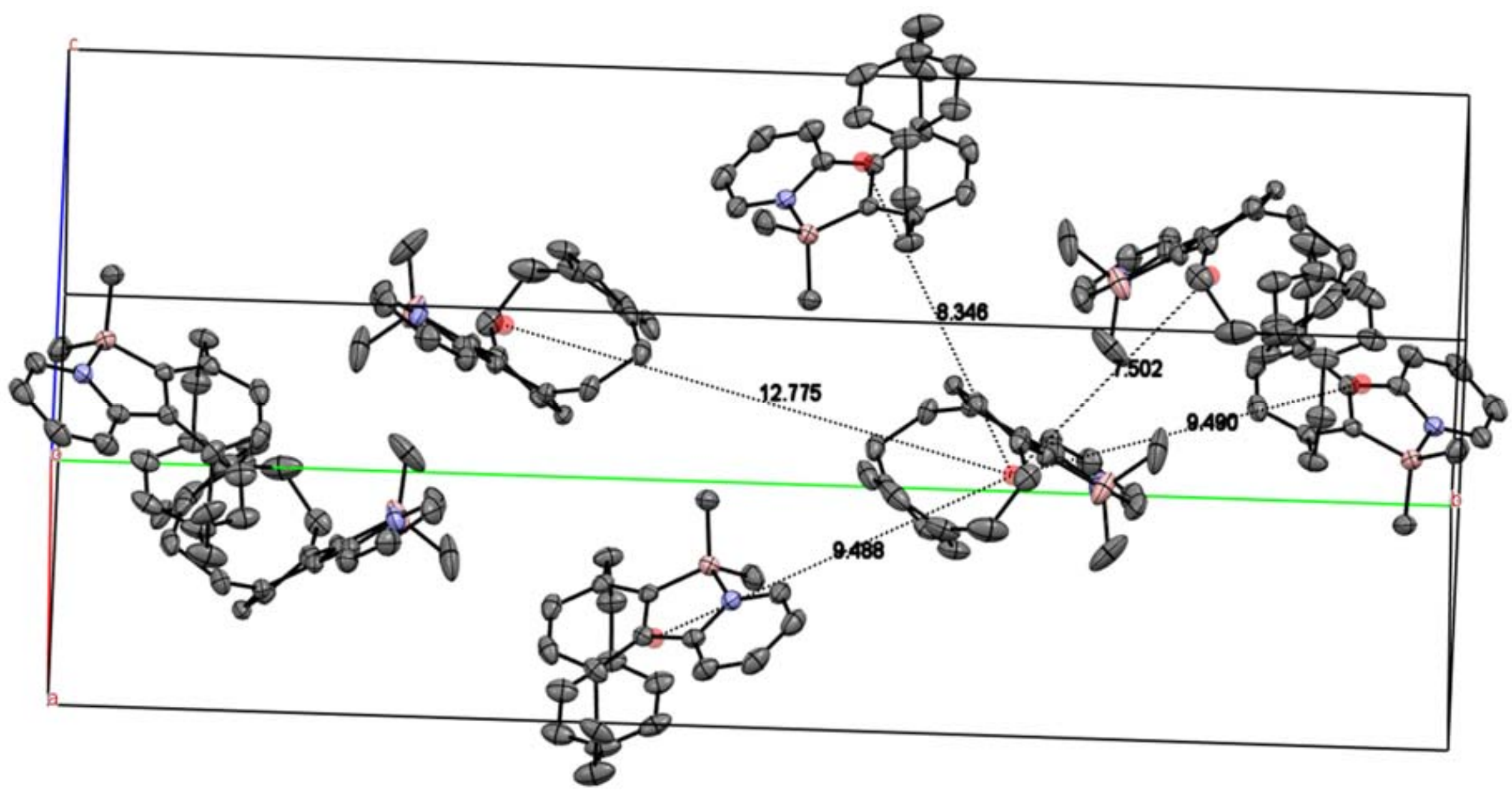

Figure S5. ORTEP drawing of T-shaped packing organization of **1a**.

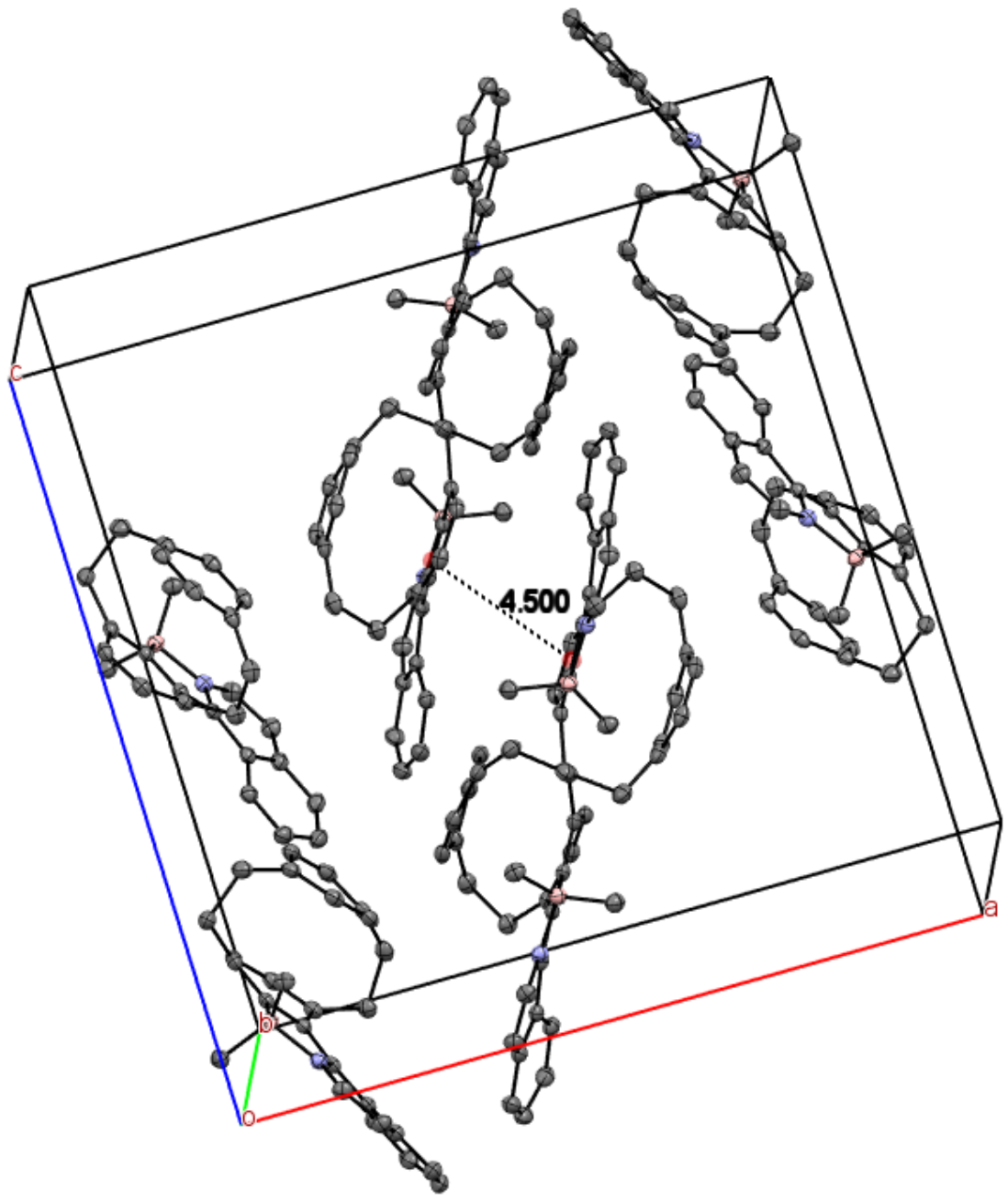

Figure S6. Crystal structure organization of **1b** with shown distance between 2 centroids of the [2.2]paracyclophane rings.

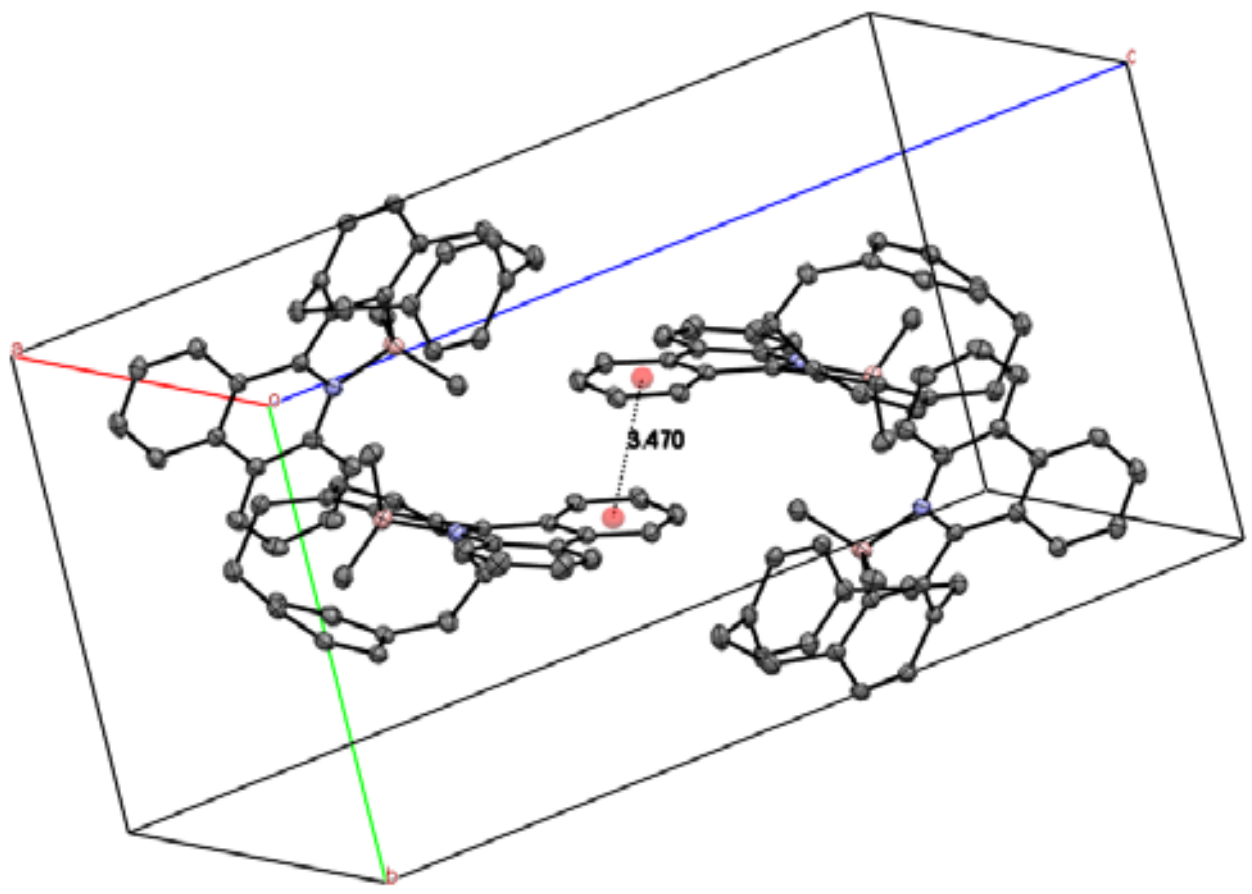

Figure S7. Crystal structure organization of **1c** with shown distance between 2 centroids of aromatic rings the phenanthridine moieties.

Solid-state packing organizations of **1b** and **1c** showed emergence of local periodicity arrangement in their solid states with localized π-stacking interactions. Consistently, enhancing π-contact area into the framework facilitates an incipient supramolecular organization which amplifies interplanar interactions (from 4.5 Å (**1b**) to 3.47 Å (**1c**)).

5 Photophysical properties

5.1 Absorption and emission

The excitation wavelengths used for fluorescent emission and quantum yield are equal to the absorption maxima. (**1a**: 340 nm; **1b**: 380 nm; **1c**: 394 nm).

Table S4. Photophysical properties of **1a**, **1b**, and **1c**.[a]

Comp.	Solvent[a]	λ_{abs} (nm)	ε ($M^{-1}\cdot cm^{-1}$)	λ_{em} (nm)[b]	λ_{em} (nm)[c]	$\Delta\underline{\nu}$ (cm^{-1})	Φ_f (%)
**1a**	CH_2Cl_2	297, 340	6749, 8374	437 (415)[e]	385	6528	15, (33)[e], (17)[d],
**1b**	CH_2Cl_2	380	9084	484 (462)[e]	431	5655	17, (17)[e], (19)[d]
**1c**	CH_2Cl_2	394	11584	510 (494)[e]	430	5773	4, (4)[e], (3)[d]
**1c**	MeCN	390	14431	518	–	6336	6
**1c**	THF	394	16066	505	–	5579	1
**1c**	toluene	398	12430	490	–	4717	1
**1c**	cyclohexane	396	8101	479	–	4376	2

[a] Quantum yields were measured in argon flushed solvents ($c \approx 10^{-5}$).

[b] Recorded emission maxima.

[c] Calculated emission maxima.

[d] In degassed CH_2Cl_2 solution via FPT method.

[e] In solid state.

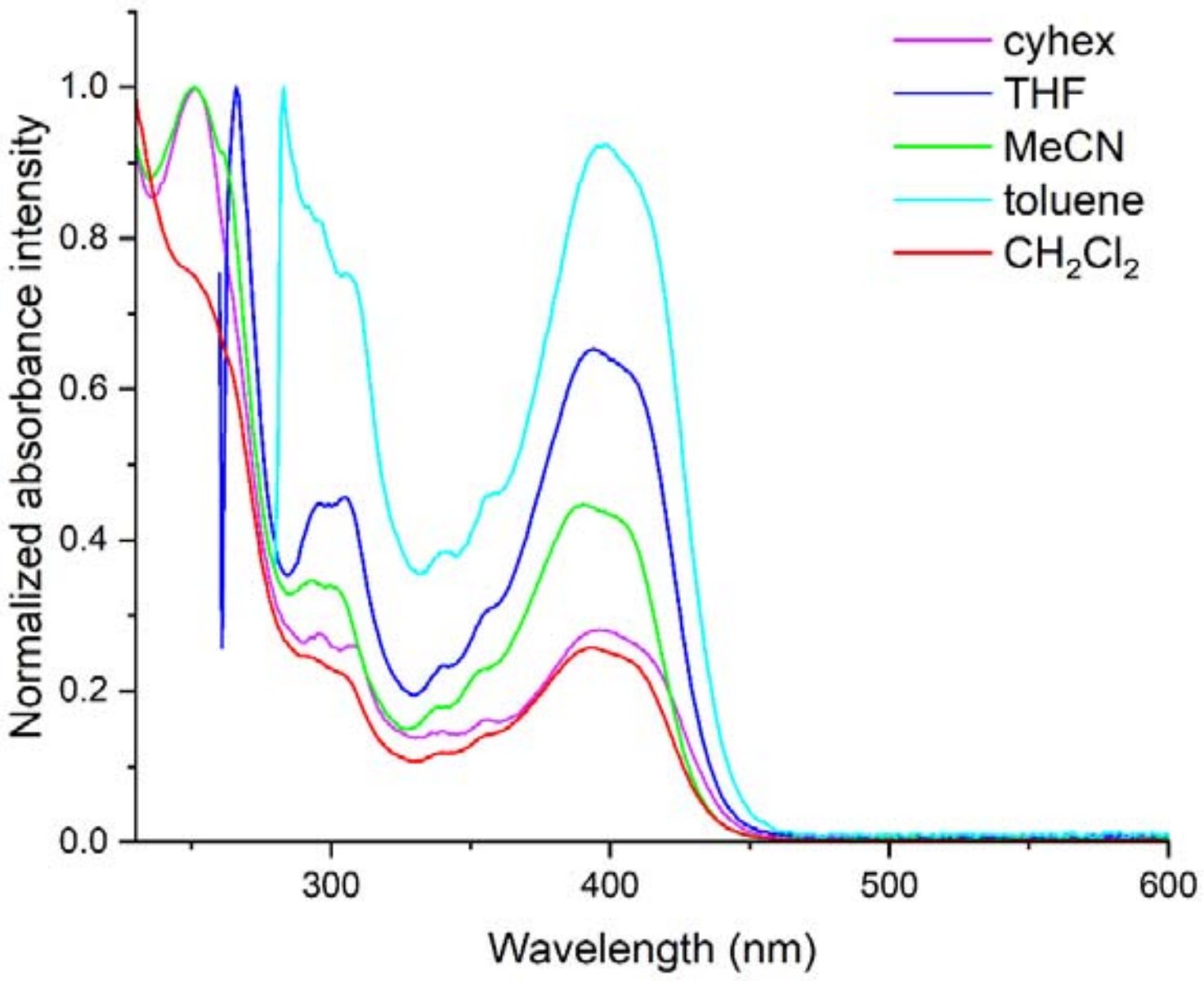

Figure S8. Normalized absorption spectra of **1c** measured as solutions in various solvents (10^{-5} M).

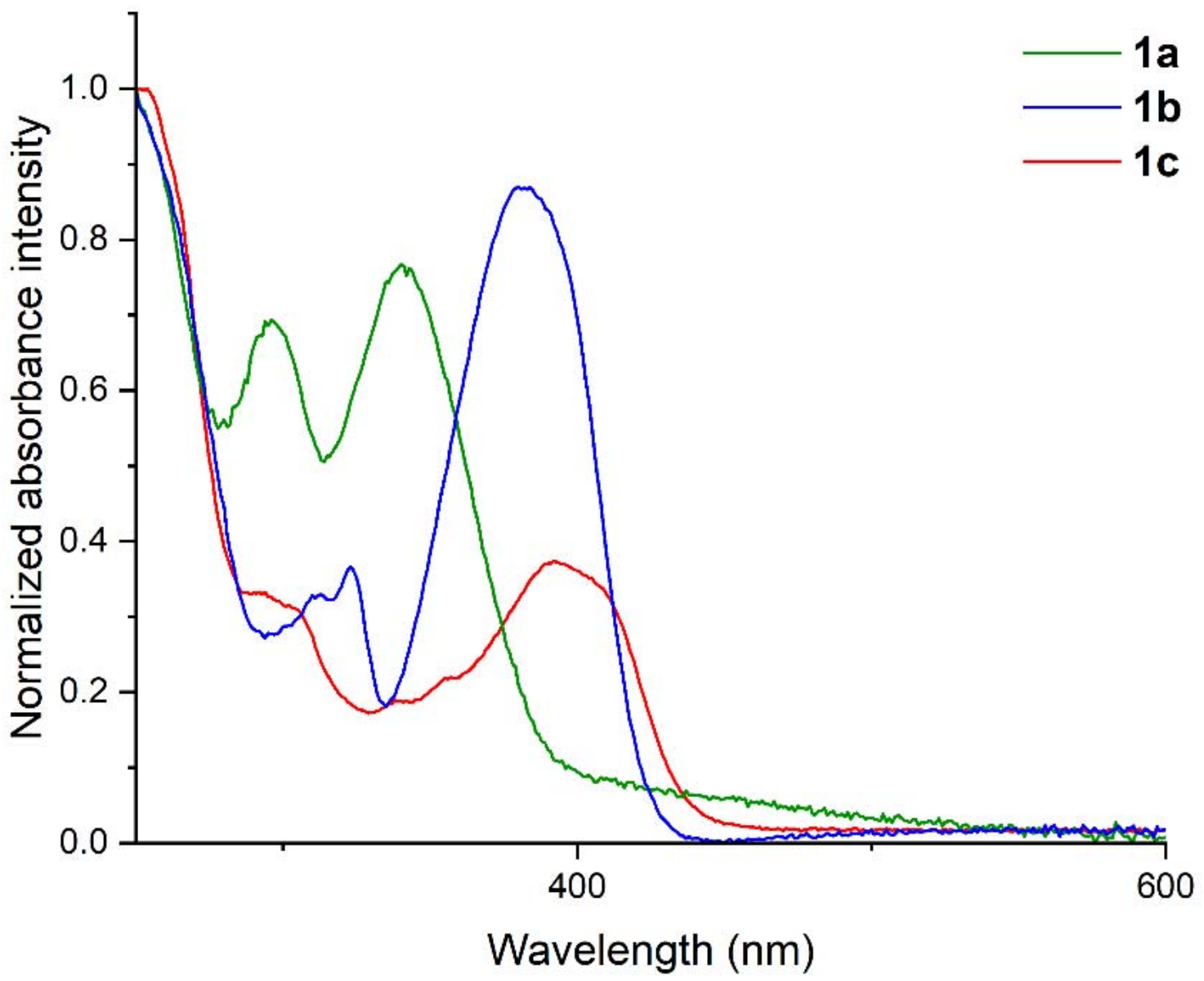

Figure S9. Normalized absorption spectra of **1a, 1b** and **1c** measured as solutions (10^{-5} M, CH_2Cl_2).

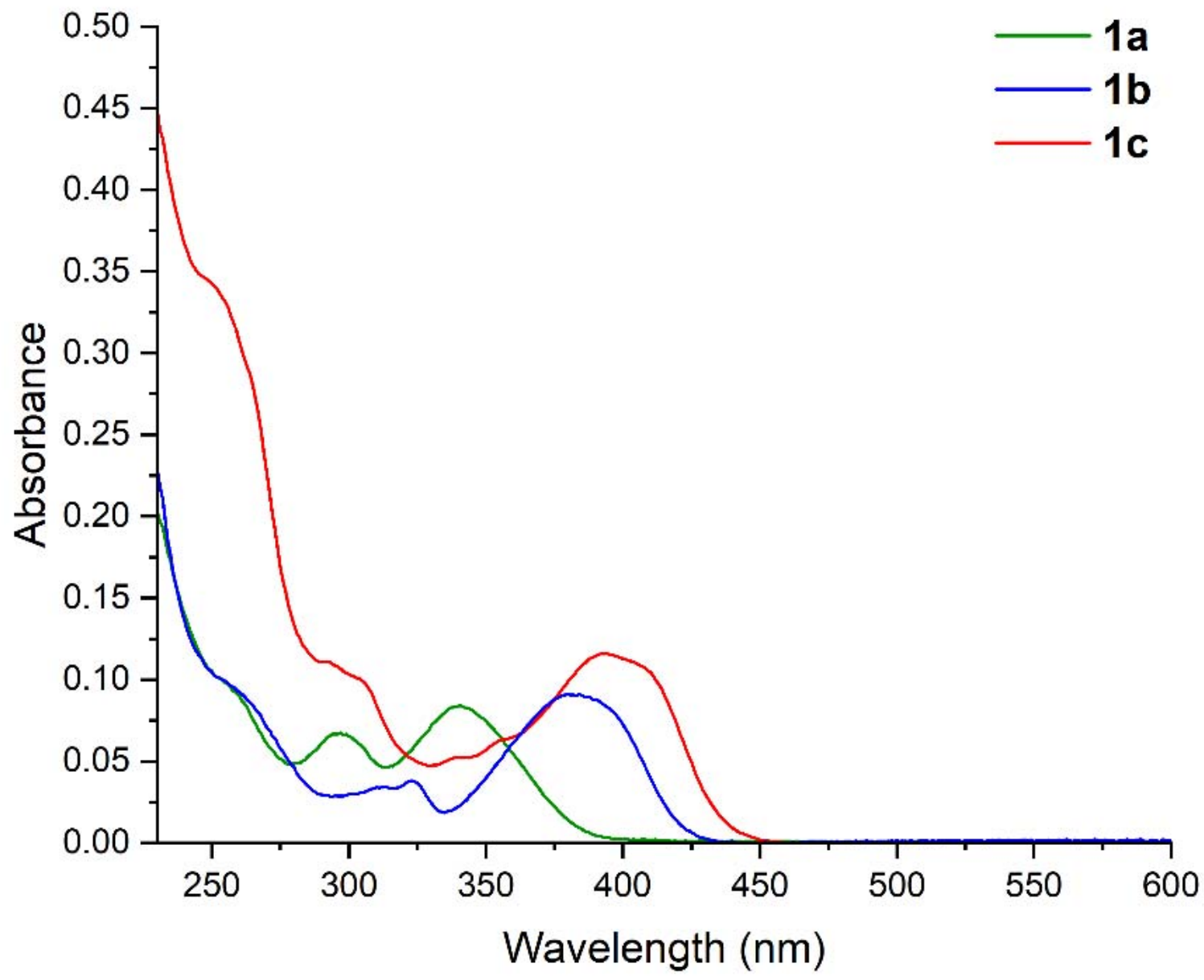

Figure S10. Non-normalized absorption spectra of **1a, 1b** and **1c** measured as solutions (10^{-5} M, CH_2Cl_2).

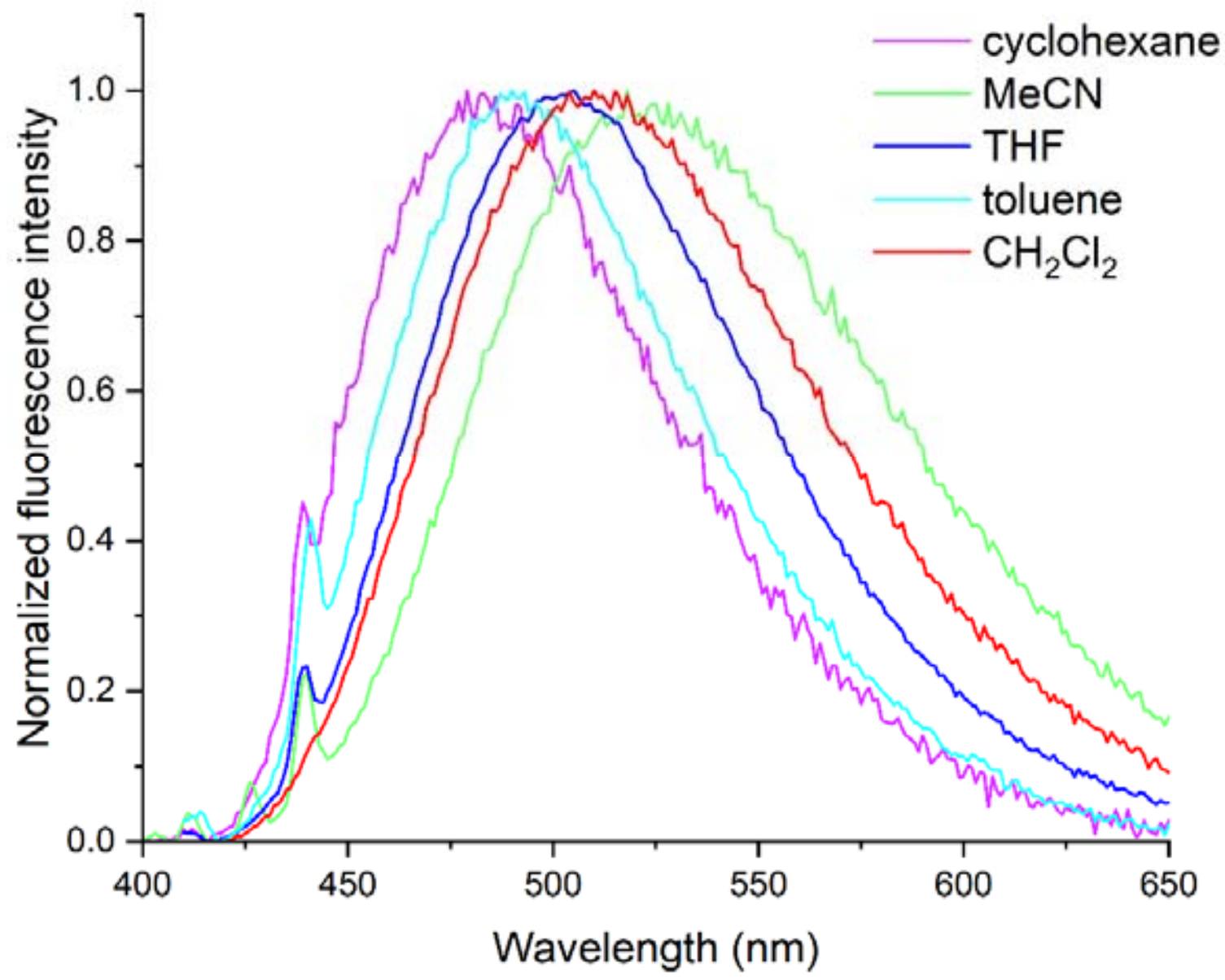

Figure S11. Normalized emission spectra of **1c** measured as solutions in various solvents (10^{-5} M).

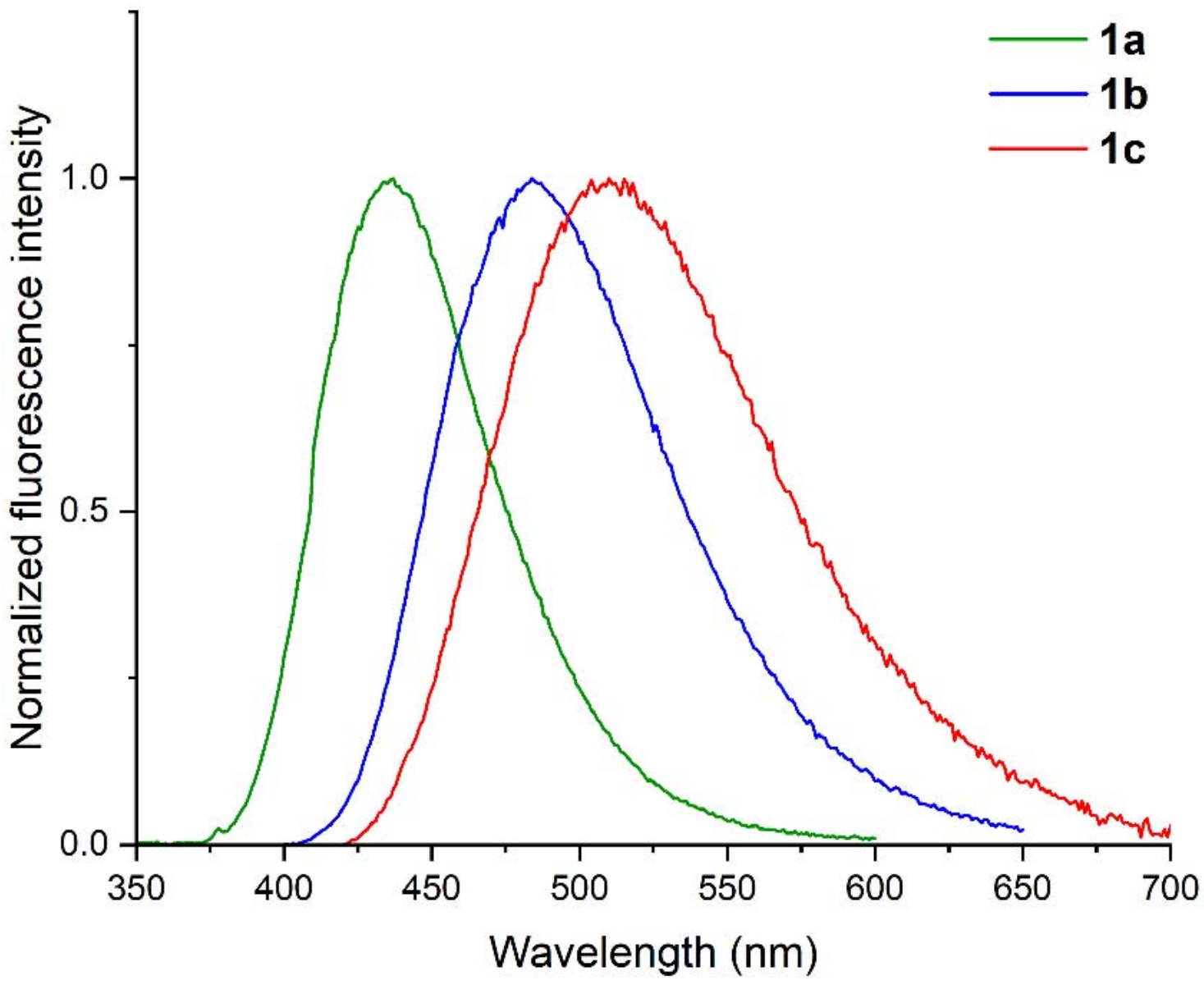

Figure S12. Normalized emission spectra of **1a**, **1b** and **1c** measured as solutions (10^{-5} M, CH_2Cl_2).

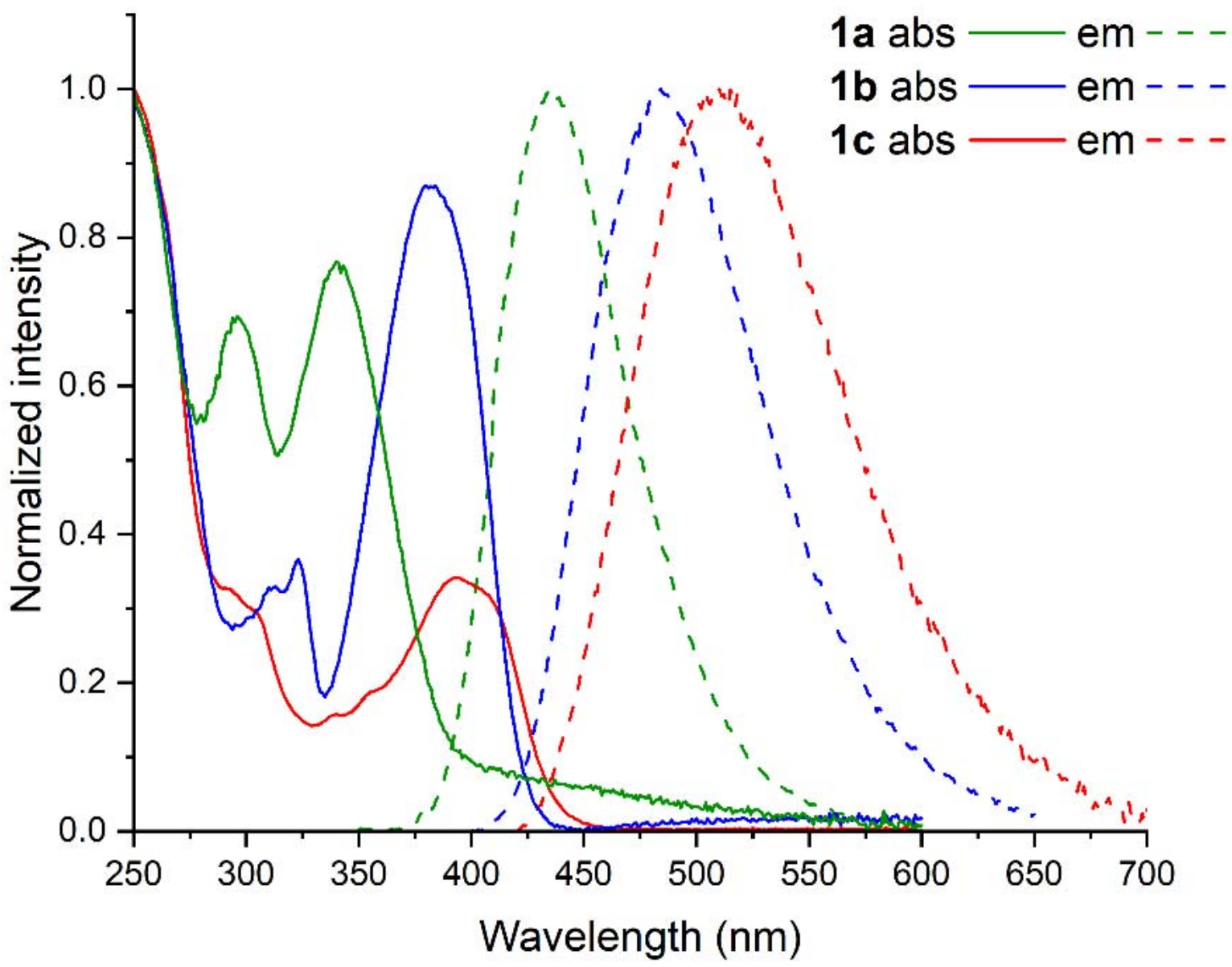

Figure S13. Combined absorption and emission spectra of **1a**, **1b** and **1c**.

5.2 Fluorescence decay measurements

Fluorescence decay measurements were conducted with a Fluorolog FL 3-22 fluorometer (Horiba Jobin Yvon, France) equipped with double grating excitation and emission monochromators, a TBX picosecond photon detection module and a FluoroHub time-correlated single photon counting (TC-SPC) module. Emission decays were measured 1 cm quartz rectangular cuvettes at the maximum emission wavelengths of each sample (Table S4) in a time window of 57 ns with the resolution of 55.82 ps per channel. The excitation source was a 378 nm NanoLED pulsed laser with the pulse width in the half-maximum of 0.2 ns, operated at a repetition frequency of 1 MHz. The instrument response function (IRF) profile was acquired using elastic scattering of dilute Ludox aqueous dispersion. The decays were fitted by single-exponential or double-exponential models using Horiba DAS6 software performing the iterative reconvolution of the fitted model with the IRF by means of the Marquardt−Levenberg nonlinear least squares algorithm.

The fluorescence decays and parameters of the models fitted to the decays are shown in Figure S10 and Table S5, respectively. **1a** and **1c** slightly deviated from a single exponential decay, exhibiting additional faster components with fractional contributions of 3% **1a** and 6% **1c**.

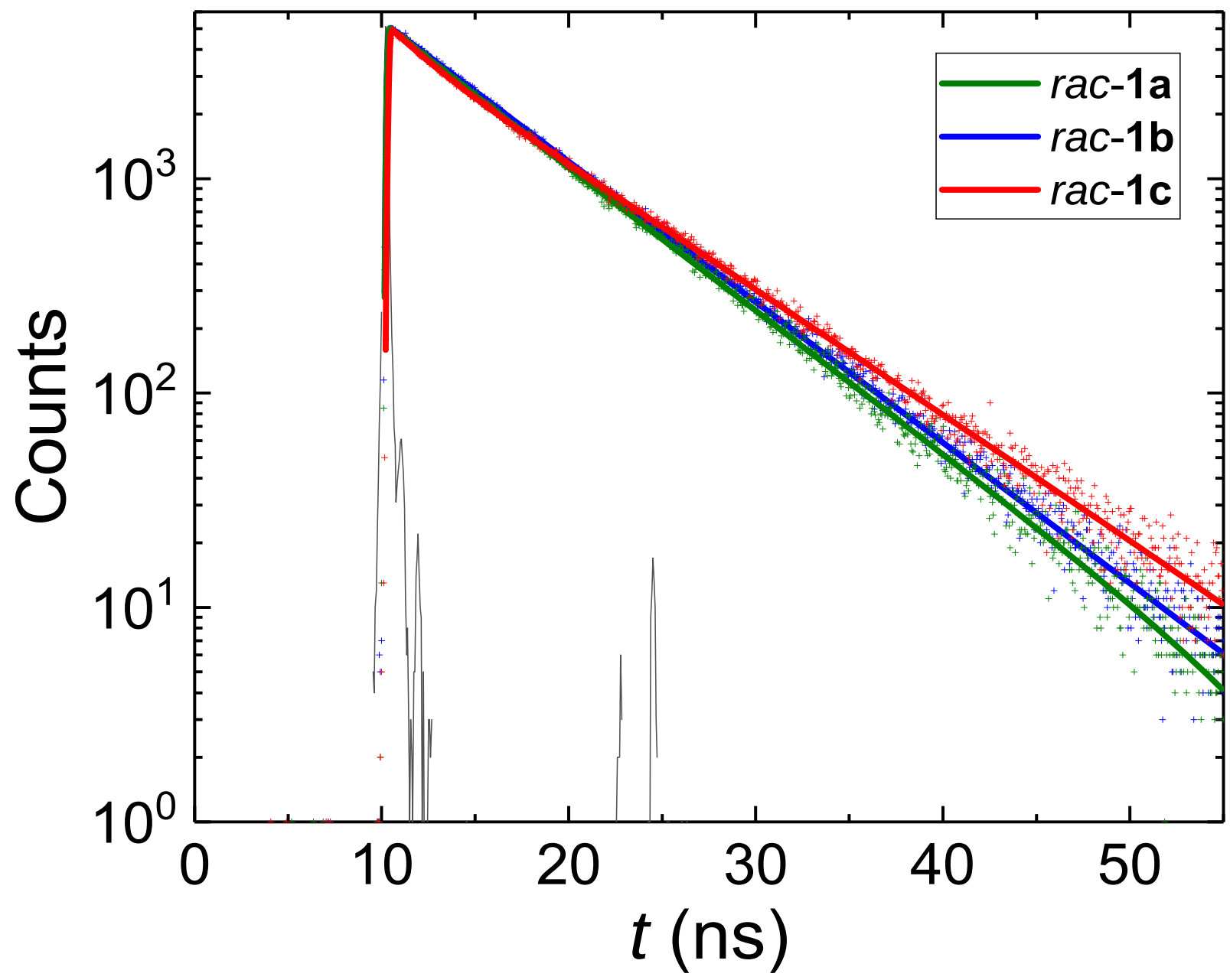

Figure S14. Fluorescence decays of **1a**, **1b** and **1c** as solutions (10^{-5} M, CH_2Cl_2).

Table S5. Parameters of fluorescence decay fits

Cpd	τ_1 (ns)	τ_2 (ns)	F_2 $\times 10^{2\,a}$	τ_f (ns)[b]	$\chi^{2\,c}$	λ_{max} (nm)[d]	$10^7 \times k_r$ (s^{-1})[e]	$10^7 \times k_{nr}$ (s^{-1})[f]	$\frac{k_{nr}}{k_r}$
**1a** [g]	6.57 (97)	3.5 (3)	3.27	6.57	1.379	437	2.3	12.9	5.6
**1b** [h]	6.63 (100)	–	–	6.63	1.428	484	2.6	12.5	4.8
**1c** [g]	7.46 (94)	2.2 (6)	6.13	7.46	1.138	510	0.54	12.1	22.4

[a] Fractional contribution for the fast component τ_2, $F_2 = \alpha_2\tau_2/(\alpha_1\tau_1 + \alpha_2\tau_2)$.

[b] Fluorescence lifetime was determined only based on the main decay component.

[c] χ^2 parameter of the fit.

[d] Emission maximum wavelength.

[e] Radiative decay constant, $k_r = \Phi_f/\tau_f$ (based on the main decay component).

[f] Non-radiative decay constant, $k_{nr} = (1 - \Phi_f)/\tau_f$ (based on the main decay component).

[g] Double-exponential decay, $I_F(t) = \alpha_1 \exp(-t/\tau_1) + \alpha_2 \exp(-t/\tau_2)$.

[h] Single exponential decay, $I_F(t) = \alpha_1 \exp(-t/\tau_1)$.

5.3 Chiroptical properties

ECD spectra of CH_2Cl_2 solutions (10^{-5} M) of (S_p)- and (R_p)-**1a**, (S_p)- and (R_p)-**1b**, and (S_p)- and (R_p)-**1c** were recorded at room temperature using a JASCO J-815 instrument. CPL measurements were performed using a home-built CPL spectrofluoropolarimeter (constructed with the help of the JASCO Company). The samples were excited using a 90° geometry with a 150 W LS Xenon ozone-free lamp. The concentrations of the samples were measured at ca. 10^{-5} M in dichloromethane at room temperature. Sample solutions of **1** were excited at 334 nm (15 accumulations for each enantiomer)

Table S6. Chiroptical properties measured in CH_2Cl_2 (10^{-5} M).

Compound	Recorded g_{abs} ($\times 10^{-3}$)[a] (λ /nm)	Calculated g_{lum} ($\times 10^{-3}$)[b]	Recorded g_{lum} ($\times 10^{-3}$)[c]	B_{CPL} ($M^{-1}\cdot cm^{-1}$)
(R_p)-**1a**	-2.7 (340 nm)	4.20	-4.7	3.0
(S_p)-**1a**	+3.0 (340 nm)	–	4.6	2.9
(R_p)-**1b**	-1.1(380 nm)	0.59	-1.3	1.0
(S_p)-**1b**	+1.2 (380 nm)	–	1.3	1.0
(R_p)-**1c**	-1.1 (400 nm)	0.72	-1.6	0.37
(S_p)-**1c**	+1.2 (400 nm)	–	+1.5	0.35

[a] Recorded g_{lum} given at the excitation wavelength used for CPL.

[b] Calculated g_{lum} factor.

[c] Recorded g_{lum} factor.

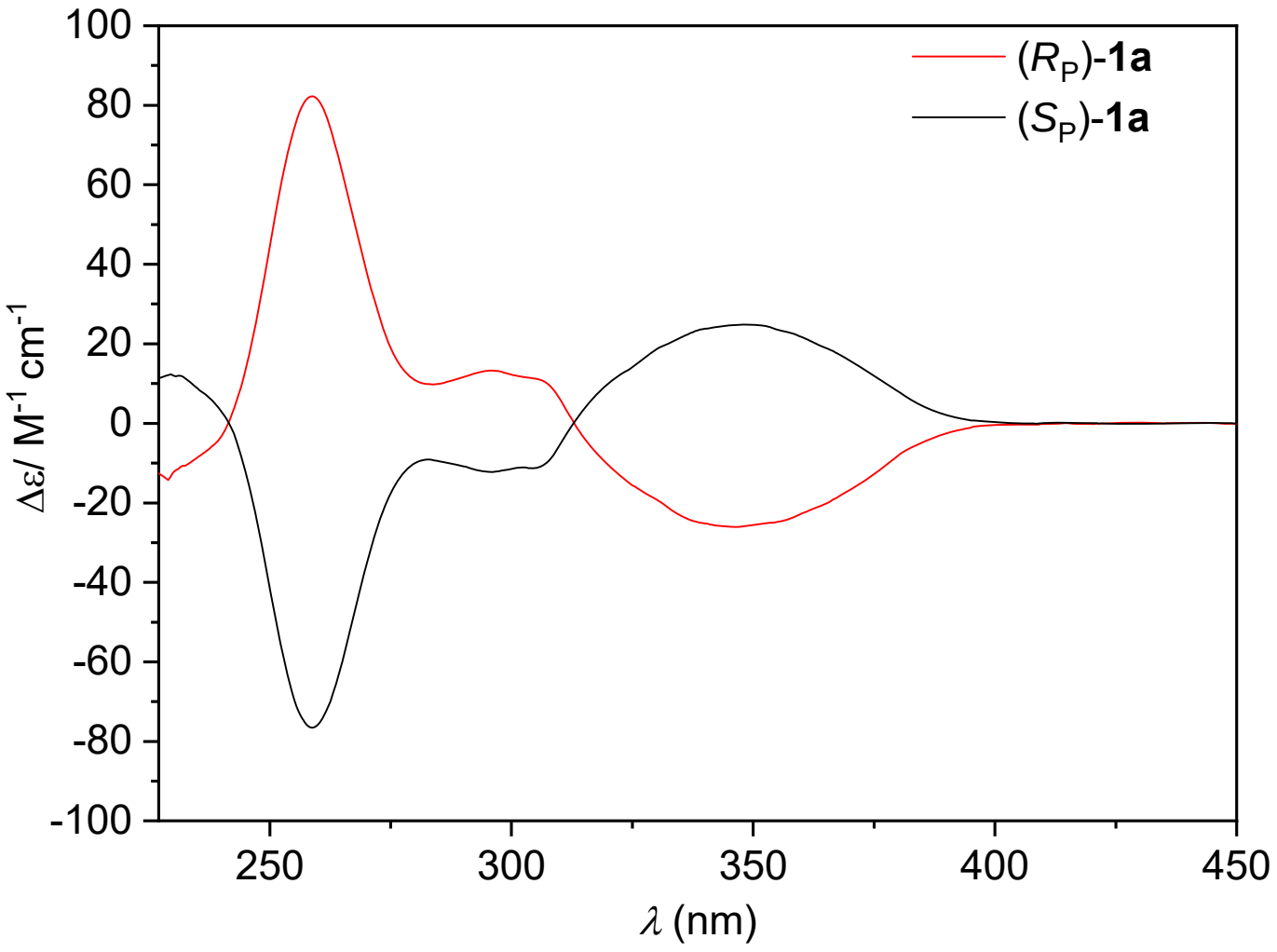

Figure S15. ECD spectra of (R_p)-**1a** (red) and (S_p)-**1a** (black).

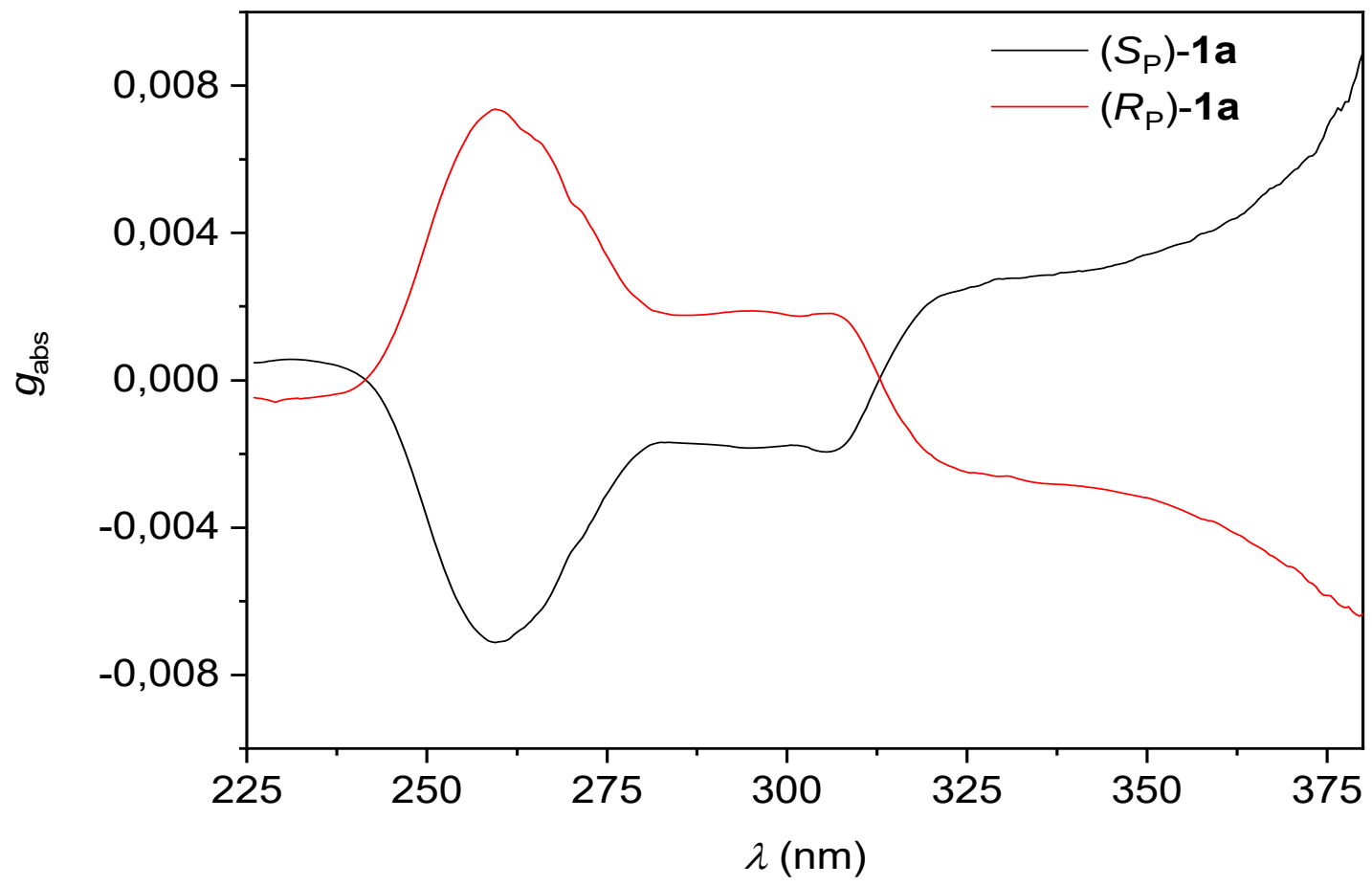

Figure S16. g_{abs} spectra of (R_p)-**1a** (red) and (S_p)-**1a** (black).

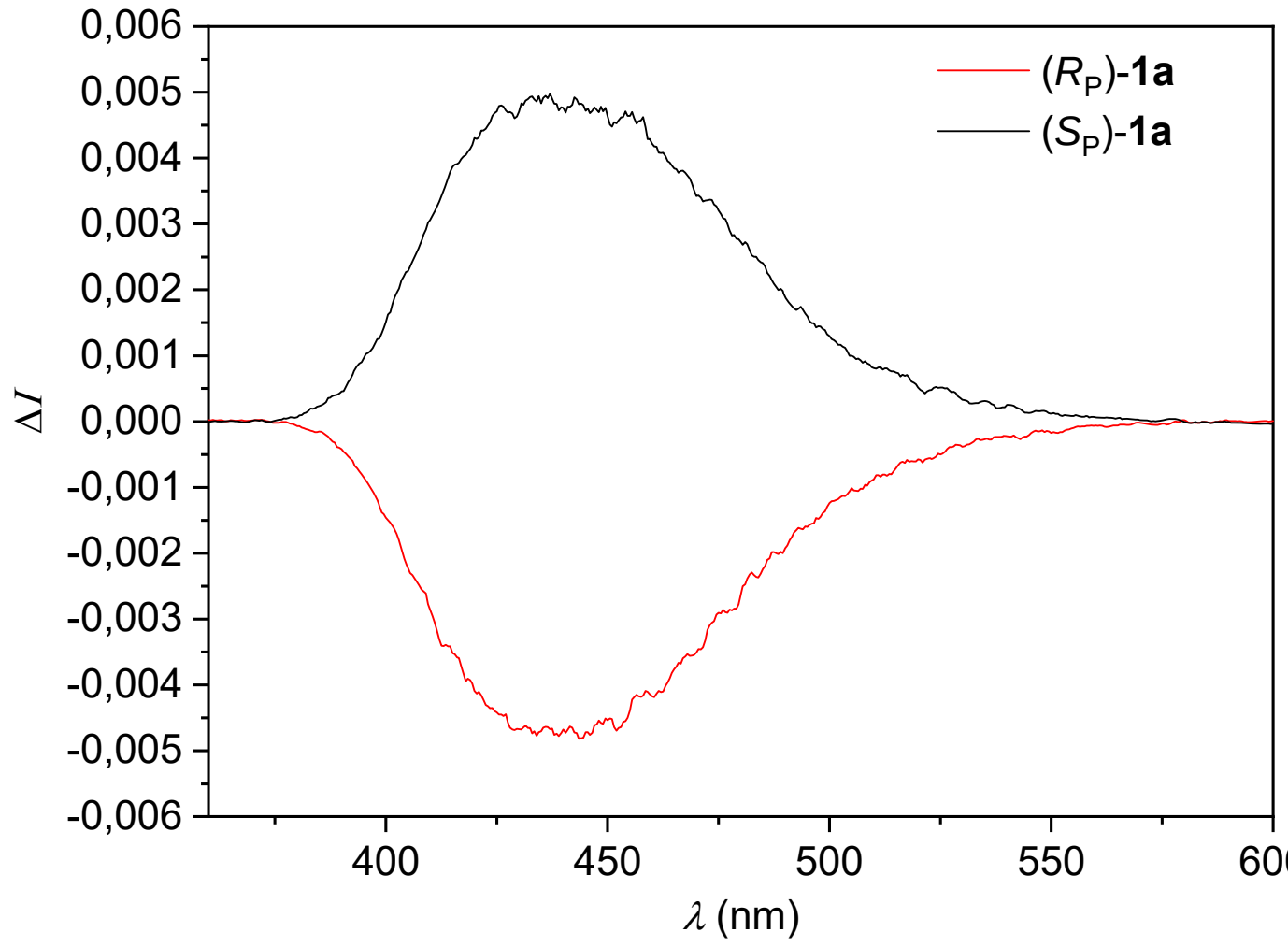

Figure S17. CPL spectra of (R_p)-**1a** (red) and (S_p)-**1a** (black) in CH_2Cl_2 (λ_{exc}: 340 nm).

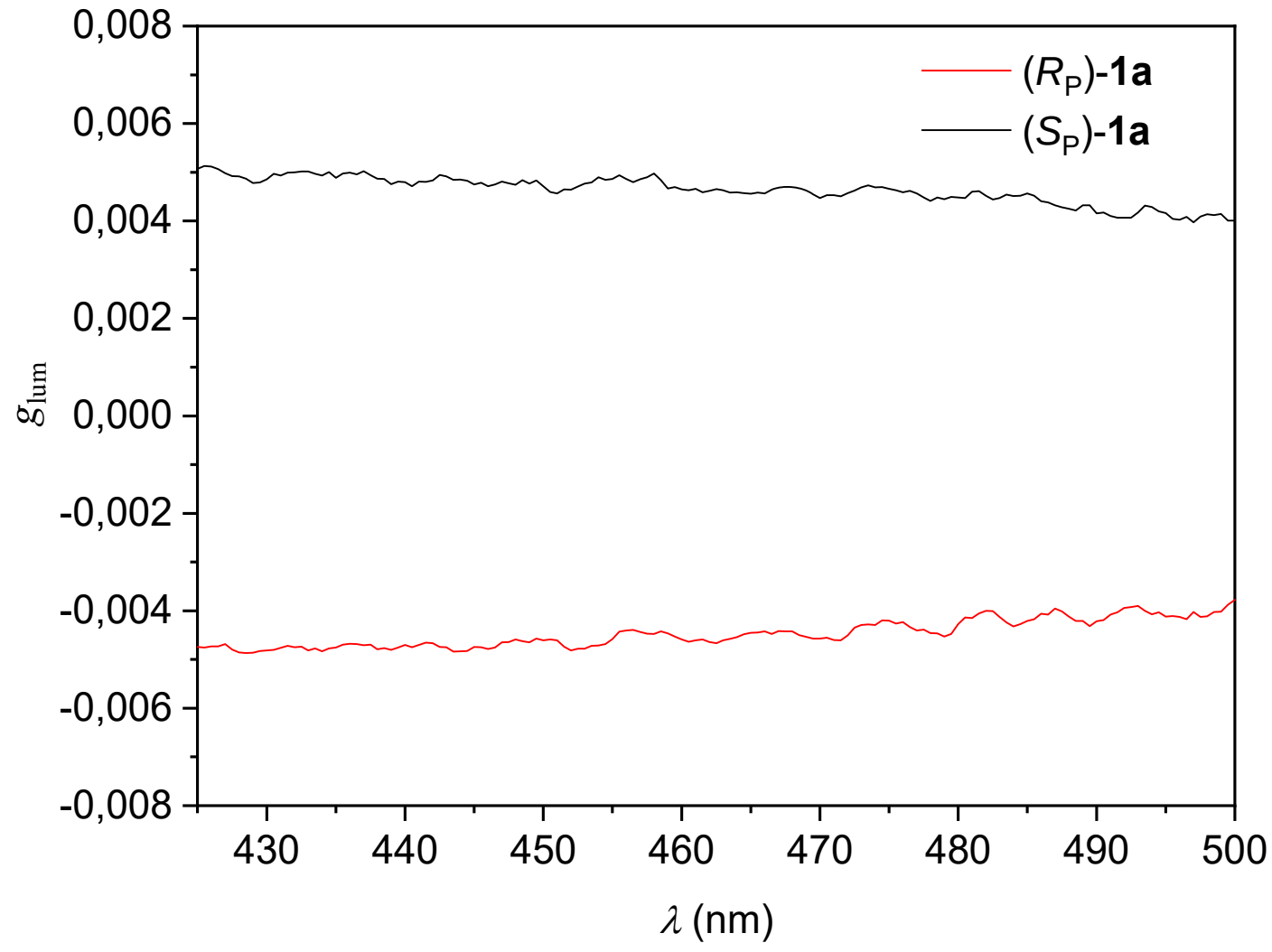

Figure S18. Emission dissymmetry factor spectra for (R_p)-**1a** (red) and (S_p)-**1a** (black).

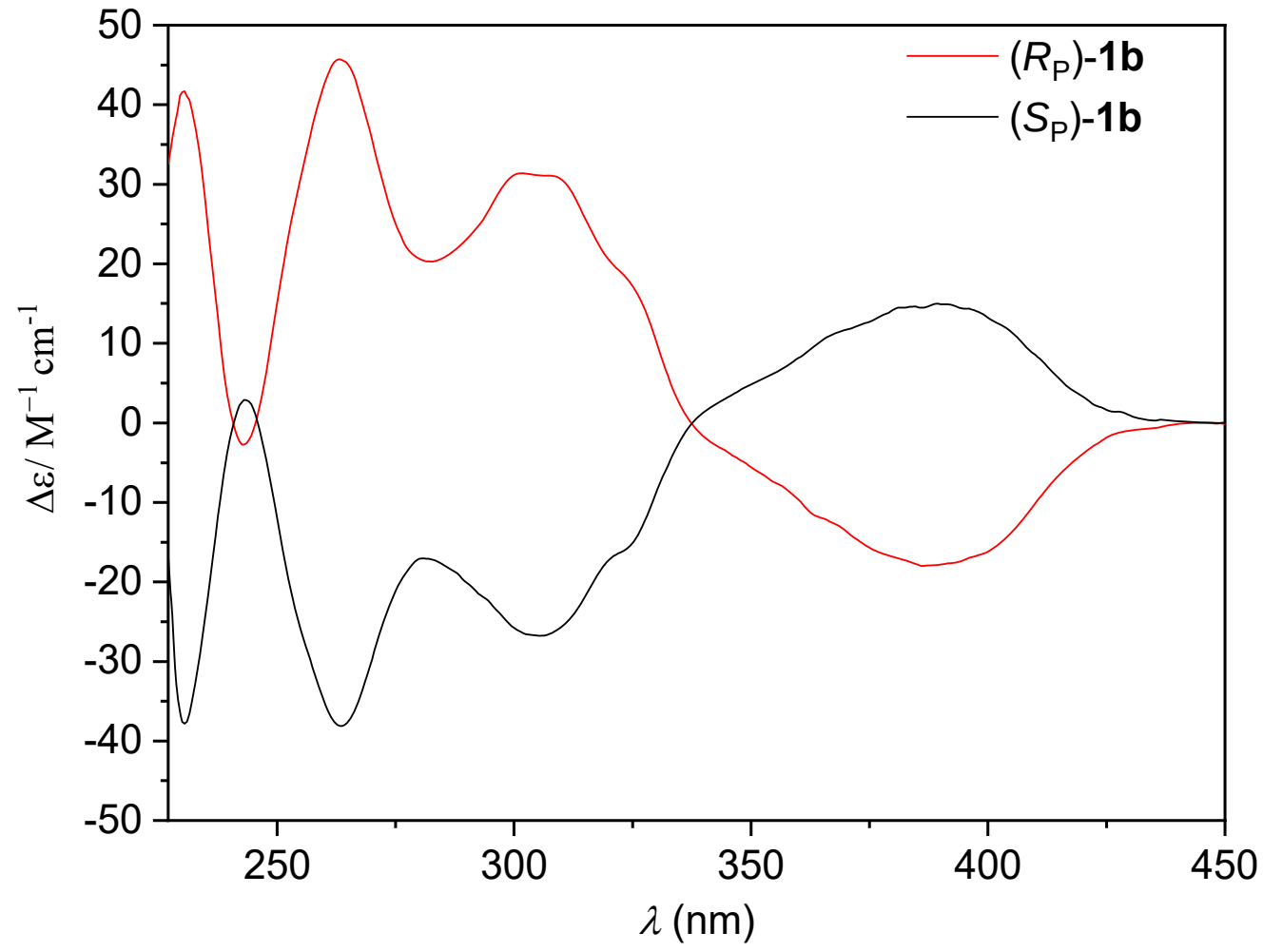

Figure S19.ECD spectra of (R_p)-**1b** (red) and (S_p)-**1b** (black).

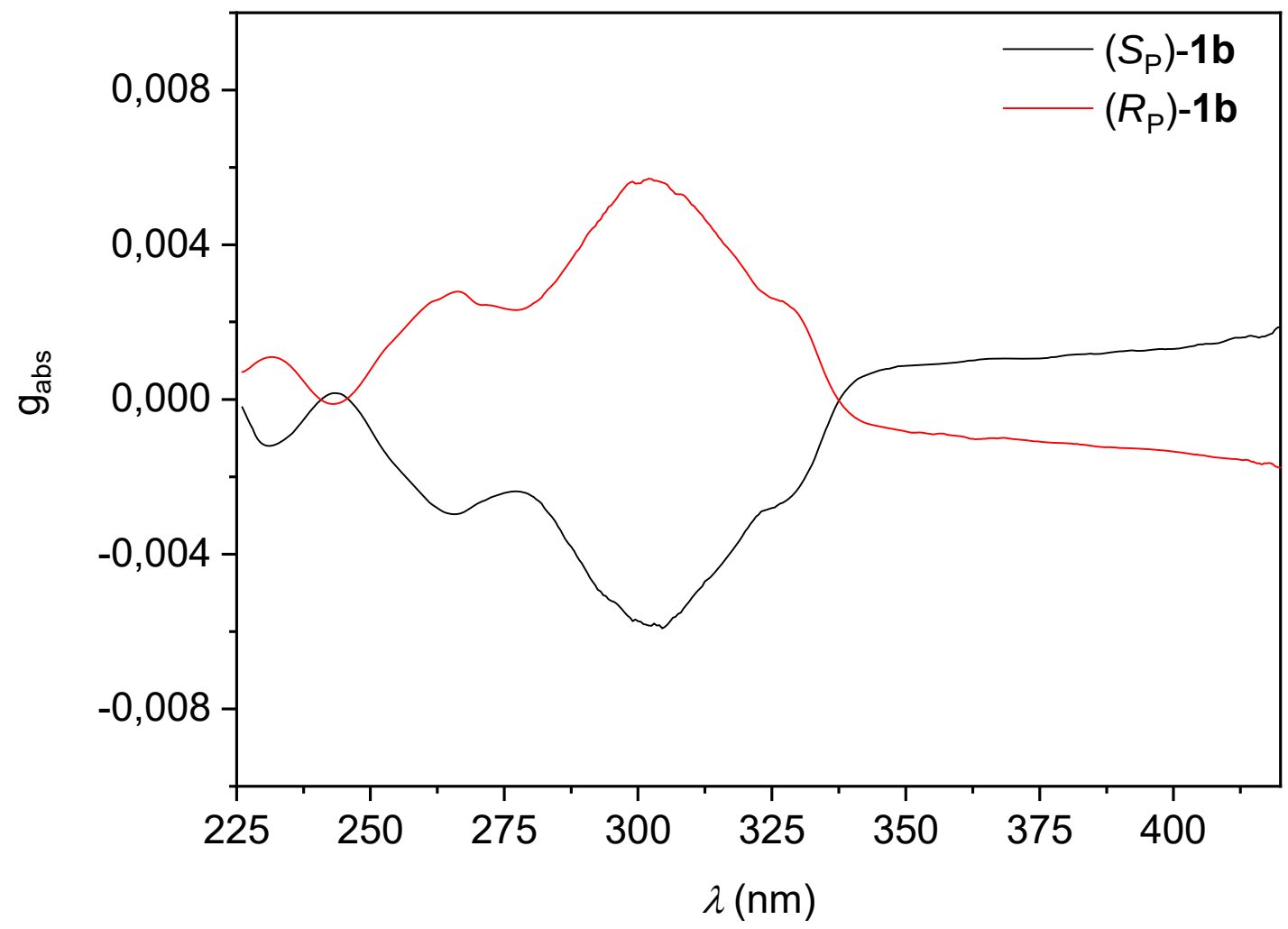

Figure S20.g_{abs} spectra of (R_p)-**1b** (red) and (S_p)-**1b** (black).

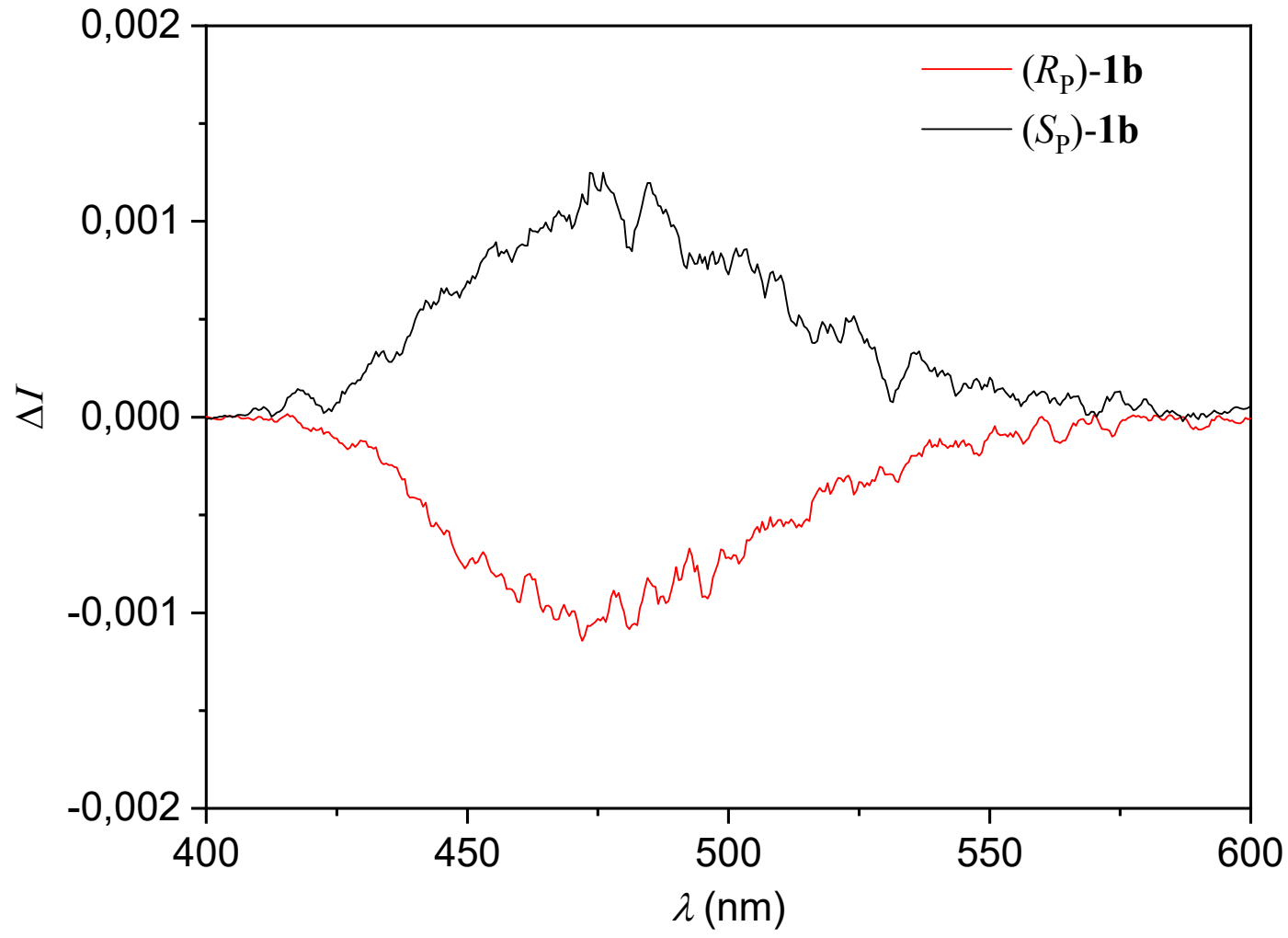

Figure S21. CPL spectra of (R_p)-**1b** (red) and (S_p)-**1b** (black) (λ_{exc}: 380 nm).

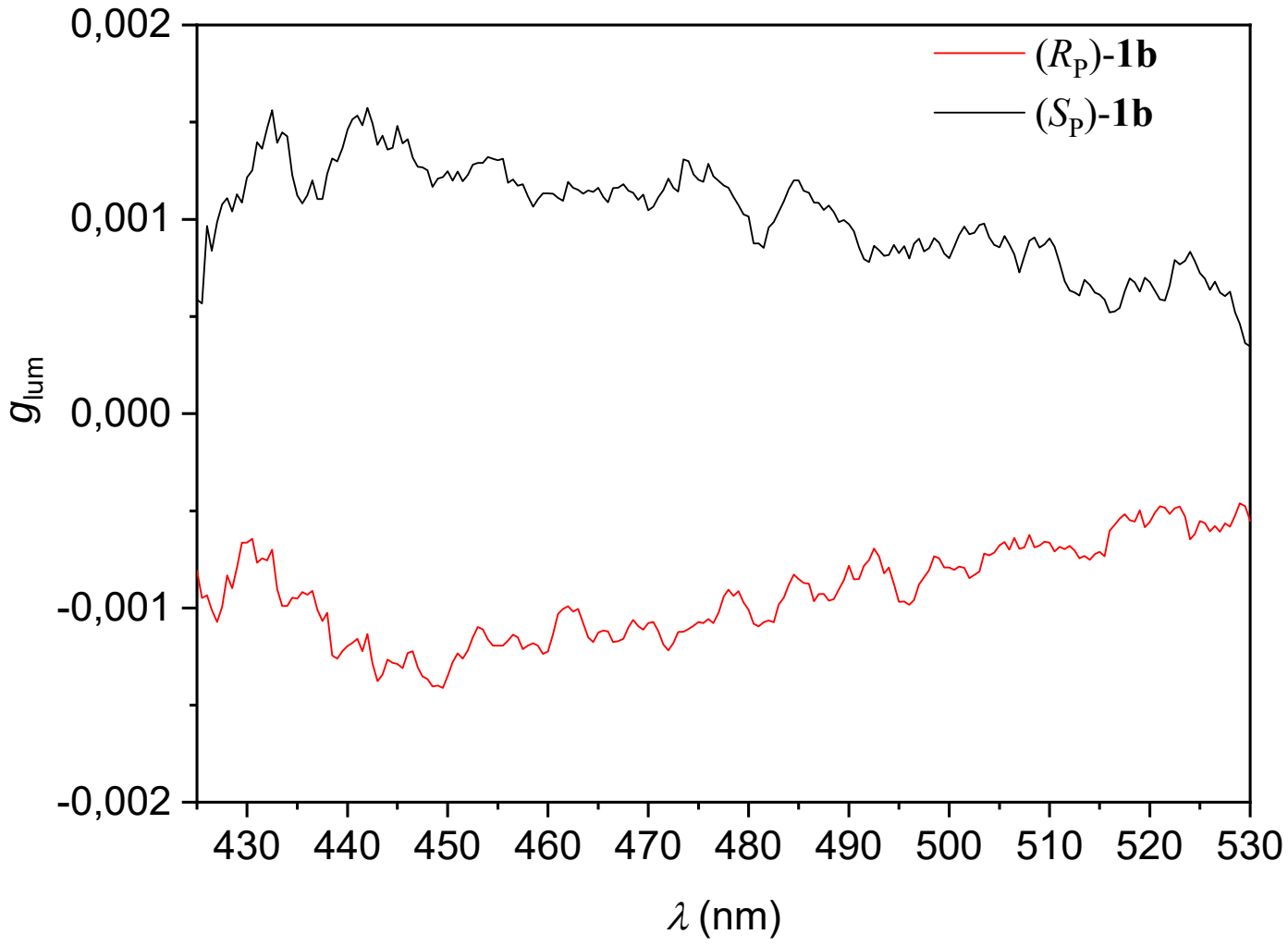

Figure S22. Emission dissymmetry factor spectra for (R_p)-**1b** (red) and (S_p)-**1b** (black).

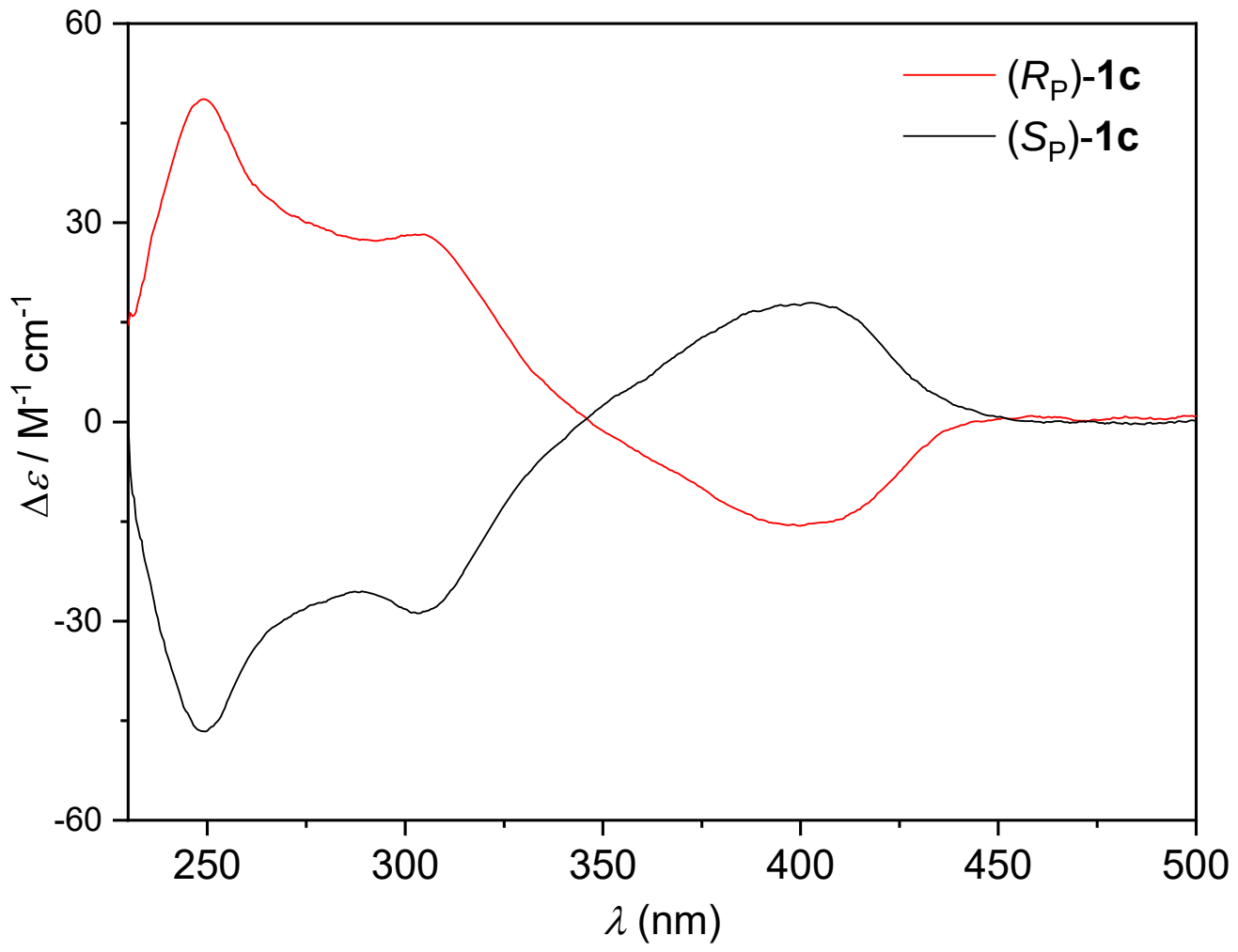

Figure S23. ECD spectra of (R_p)-**1c** (red) and (S_p)-**1c** (black).

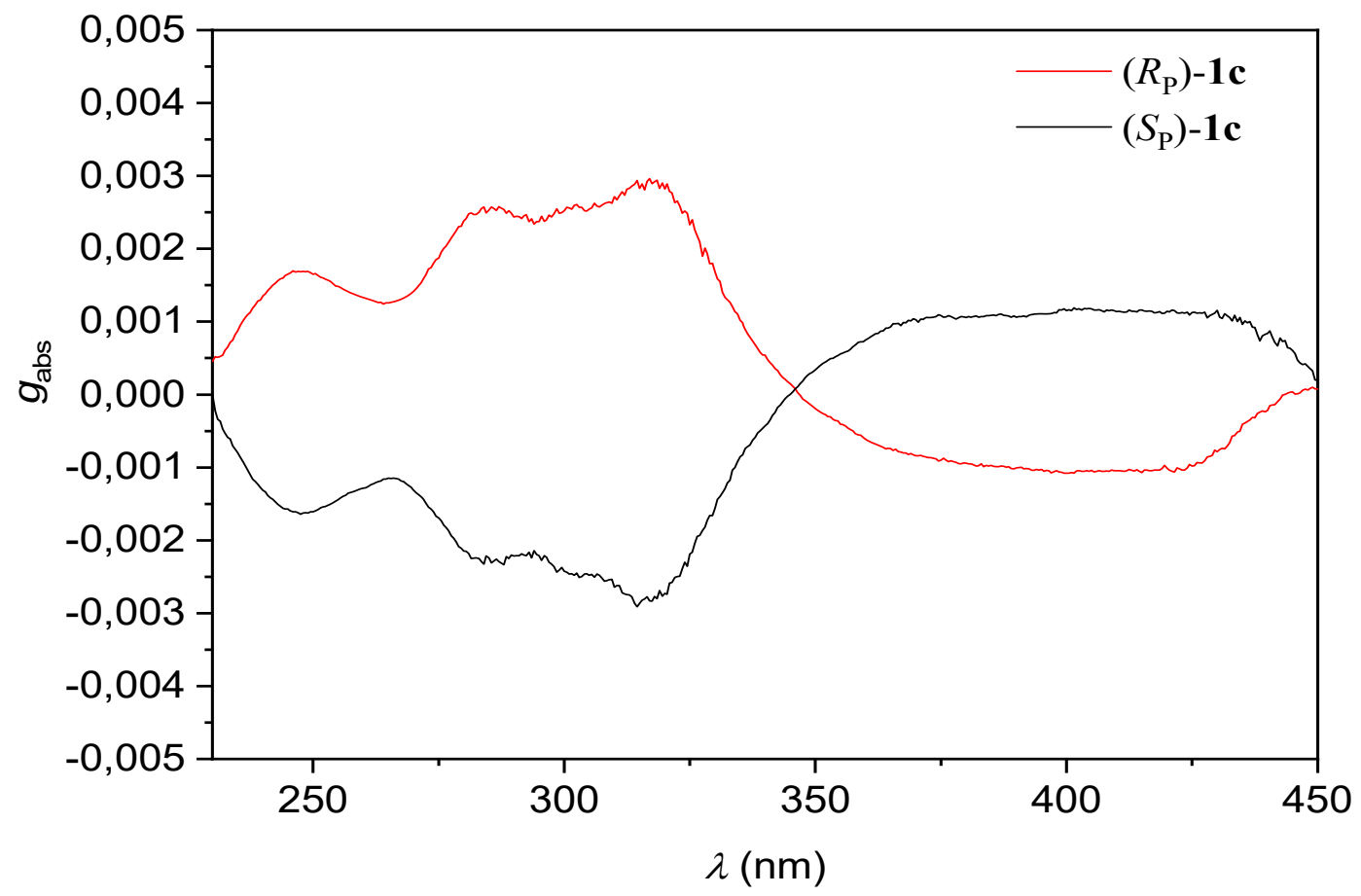

Figure S24. g_{abs} spectra of (R_p)-**1c** (red) and (S_p)-**1c** (black).

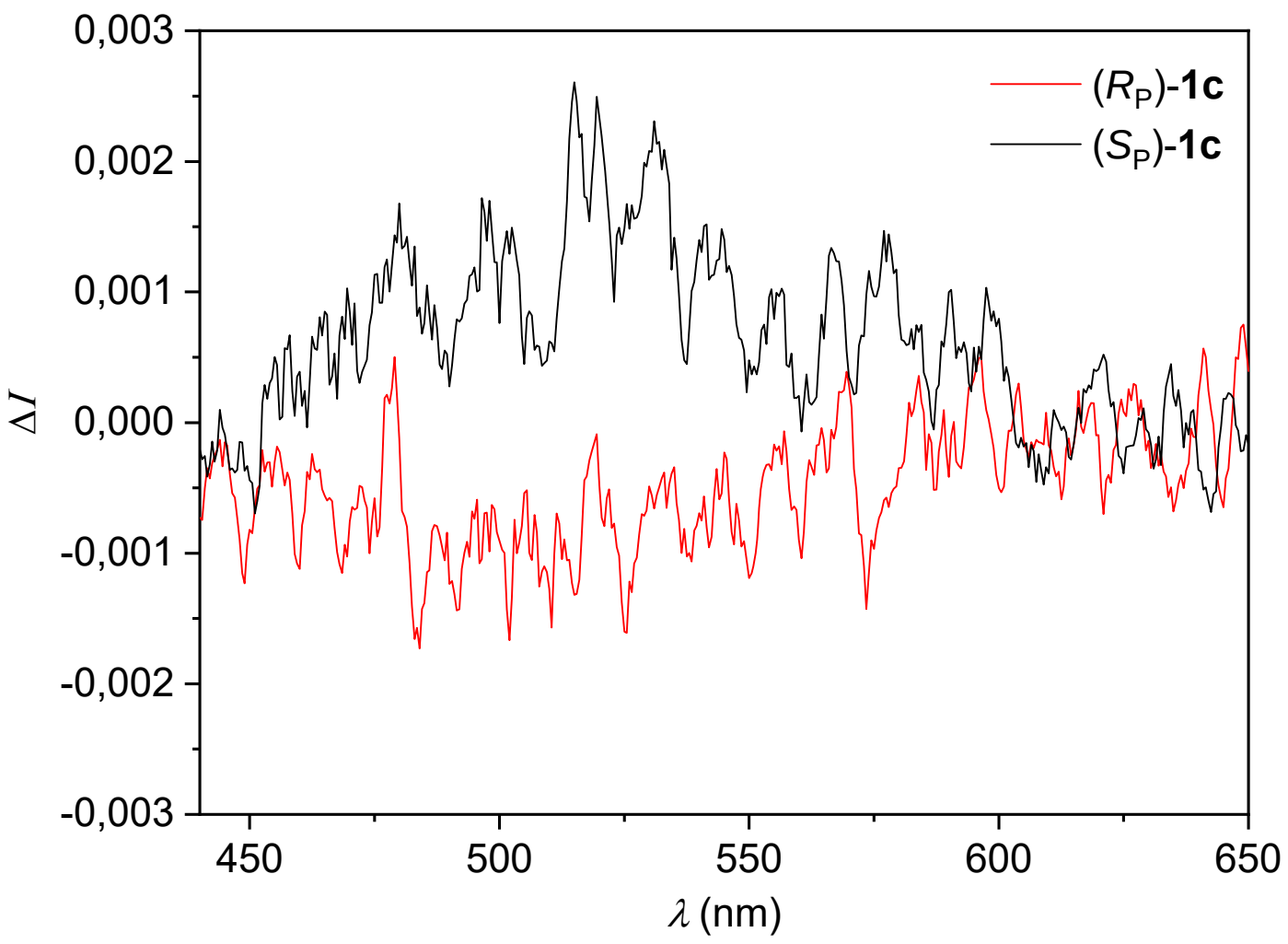

Figure S25. CPL spectra of (R_p)-**1c** (red) and (S_p)-**1c** (black) (λ_{exc}: 400 nm).

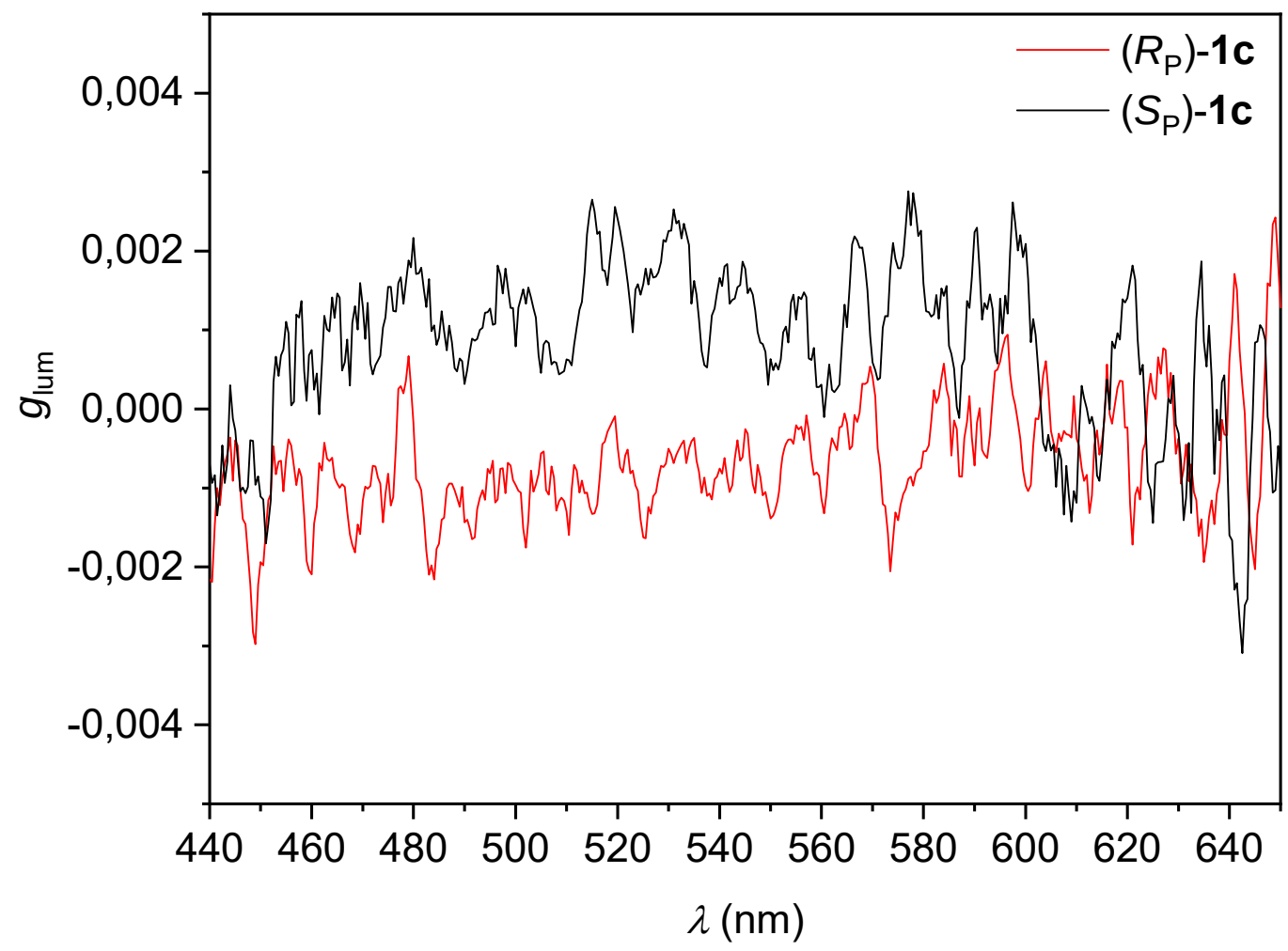

Figure S26. Emission dissymmetry factor spectra for (R_p)-**1c** (red) and (S_p)-**1c** (black).

The performance of chiral emitters can be evaluated by several parameters. The most often used parameters to evaluate chiral emitters are photoluminescence quantum yield (Φ_{PL}), luminescence dissymmetry factor (g_{lum}), and CPL brightness (B_{CPL}), see below:[11, 12]

$$g_{\mathrm{lum}} = 2\frac{I_{\mathrm{L}} - I_{\mathrm{R}}}{I_{\mathrm{L}} + I_{\mathrm{R}}} \quad \text{(Eq. 1)} \qquad \text{or} \qquad g_{\mathrm{lum}} = \frac{4\,|\mu_{ij}|\,|\boldsymbol{m}_{ji}|\,cos\,\theta_{ij}}{|\mu_{ij}^2| + |\boldsymbol{m}_{ji}^2|} = \frac{4R}{D} \quad \text{(Eq. 2)}$$

I_L and I_R = the emission intensities of left and right-handed circularly polarized light.

For $i \rightarrow j$ transition

μ_{ji} and $\boldsymbol{m}_{ji}$ = the electric and magnetic transition dipole vectors

θ_{ij} = the angle between μ_{ji} and m_{ji}.

$$B_{\mathrm{CPL}} = \left| \frac{1}{2} \times \varepsilon_{\lambda} \times \Phi \times g_{\mathrm{lum}} \right|$$

ε_{λ} = molar extinction coefficient at excitation wavelength

Φ = luminescence quantum yield (0–1.0)

g_{lum} = luminescence dissymmetry factor

[11] Mori, T. in Circularly Polarized Luminescence of Isolated Small Organic Molecules. (Ed. Mori, T.), Springer Nature Singapore Pte Ltd, **2020**, Ch. 1, 1-10.

[12] Arrico, L; Di Bari, L.; Zinna, F. *Chem. Eur. J.* **2021**, *27*, 2920–2934.

Calculation of g_{lum} factor for **1a–c**.

Density Functional Theory (DFT) computational studies were carried out using Gaussian 16, Revision C.01. Computations utilized the M06-2X functional and the def2-TZVP basis set on all atoms. Geometry optimization of the first excited-state geometries used time-dependant DFT computations considering eight states. Empirical dispersion was accounted for by the D3 version of Grimme's dispersion correction. Effects of dichloromethane as solvent were included by employing the Solvent Accessible Surface model.

Table S7. Calculated values of **1a–c** (angle between electric and magnetic transition dipole vectors)

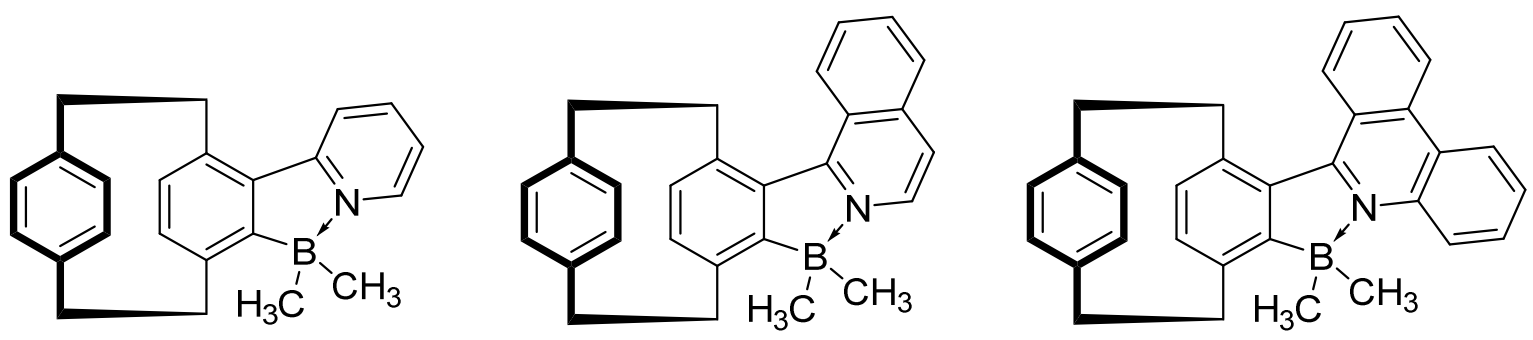

Compound	λ_{abs} (nm)	λ_{lum} (nm)	Calcd. g_{lum}	$\theta_{\mu-m}$ (°)[a]
**1a**	308.65	384.58	$4.20 \cdot 10^{-3}$	66.6
**1b**	344.53	431.11	$5.85 \cdot 10^{-4}$	81.7
**1c**	350.20	429.75	$7.18 \cdot 10^{-4}$	81.6

Table S8. **ESI_QC_TOTAL_ENERGIES**. Computed total energies (in atomic units) for the optimized geometries of **1a**–**1c** in their ground state (E_{GS}) and first excited state (E_{EXC}). Number imaginary excitations is 0.

Compound	E_{GS}	E_{EXC}
**1a**	-970.34850620	-970.33577163
**1b**	-1123.95638486	-1123.94424416
**1c**	-1277.56900480	-1277.55825371

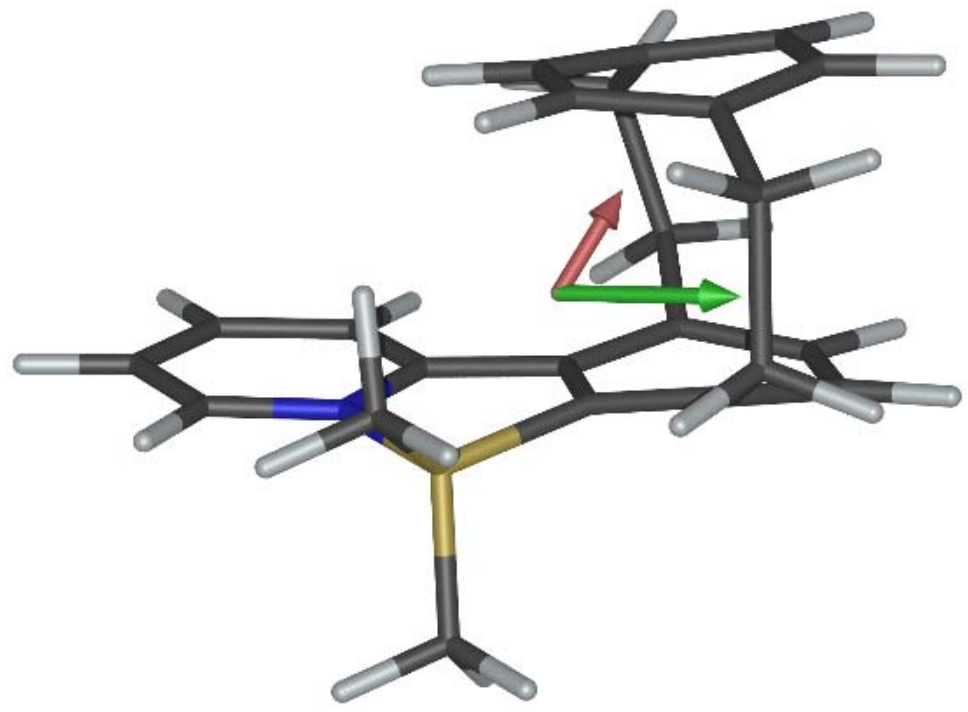

Figure S27. Simulation of electric (green arrow) and magnetic (red arrow) transition dipole moments of **1a**.

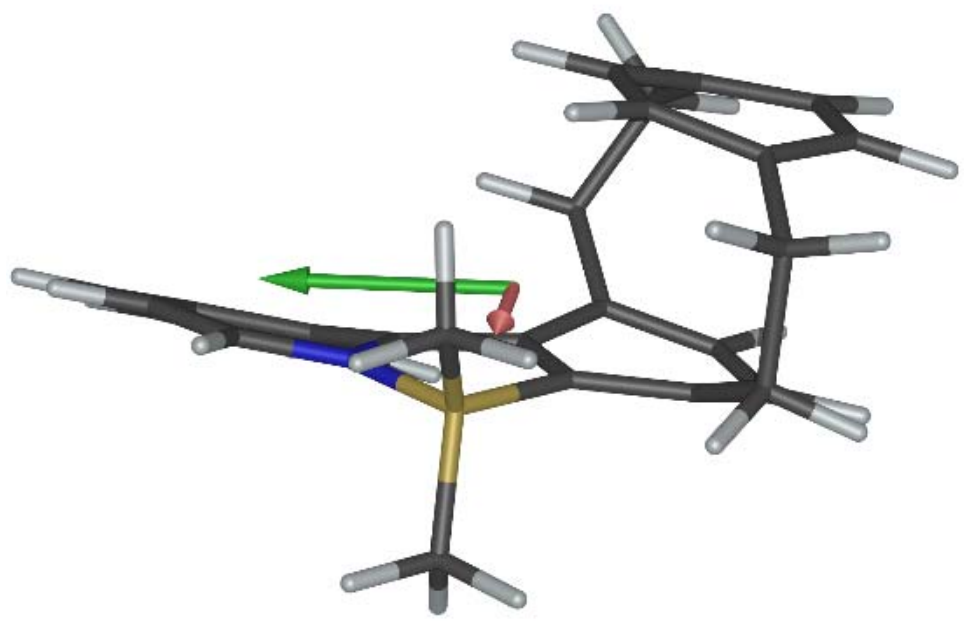

Figure S28. Simulation of electric (green arrow) and magnetic (red arrow) transition dipole moments of **1b**.

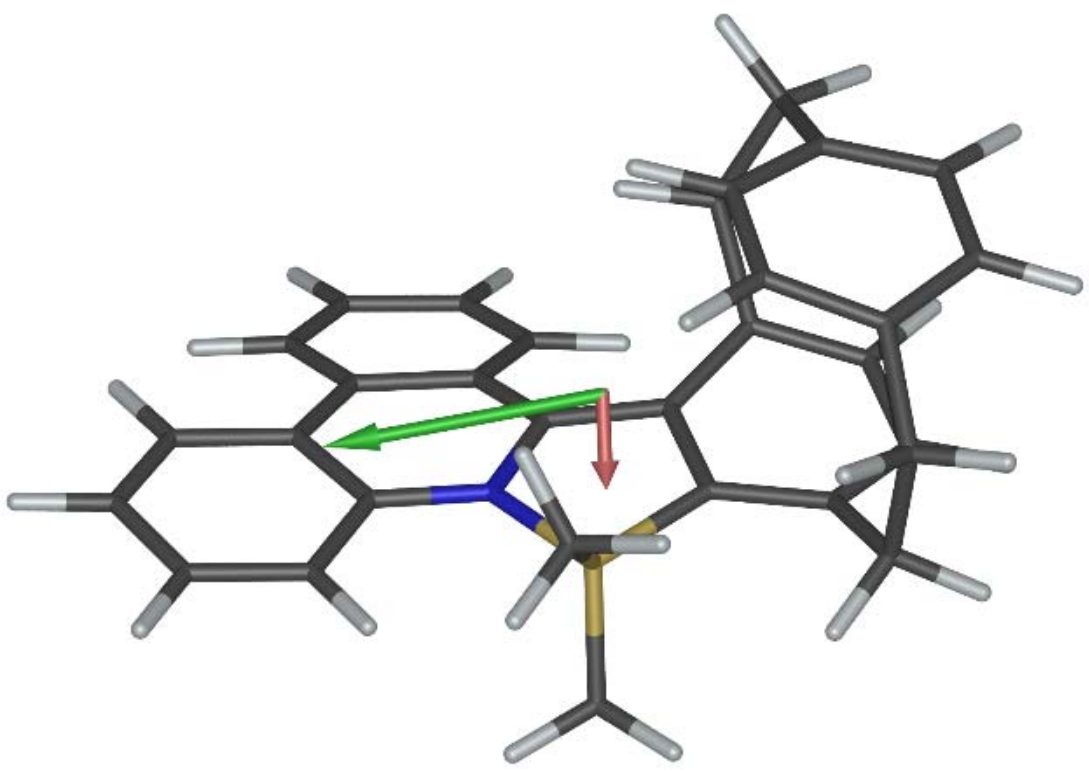

Figure S29. Simulation of electric (green arrow) and magnetic (red arrow) transition dipole moments of **1c**.

6 HOMO-LUMO

Figure S30. Frontier contour diagrams for **1a** and table of its three lowest excitations.

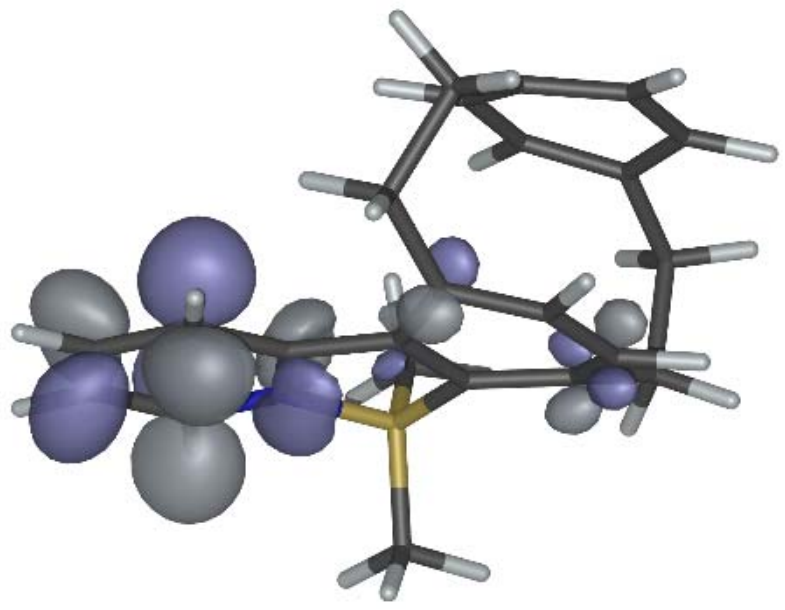

LUMO+1
ε = -0.04 eV

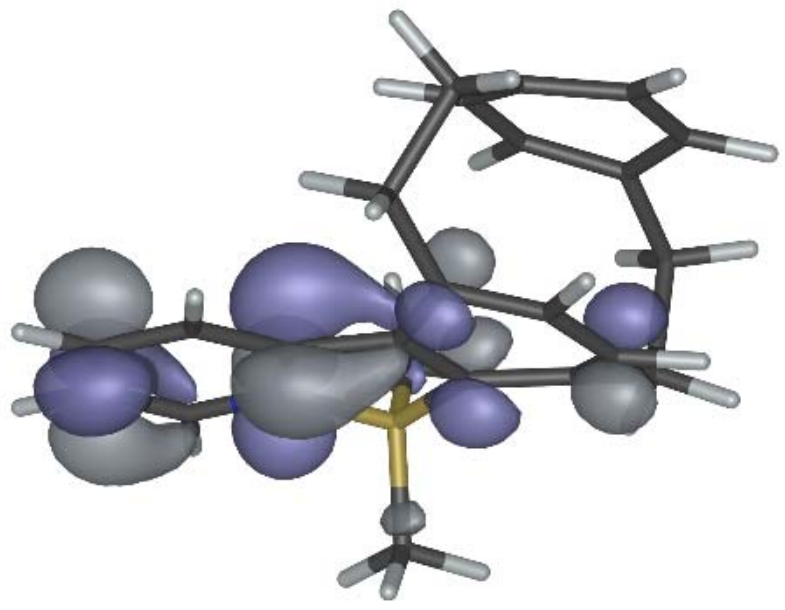

LUMO
ε = -0.59 eV

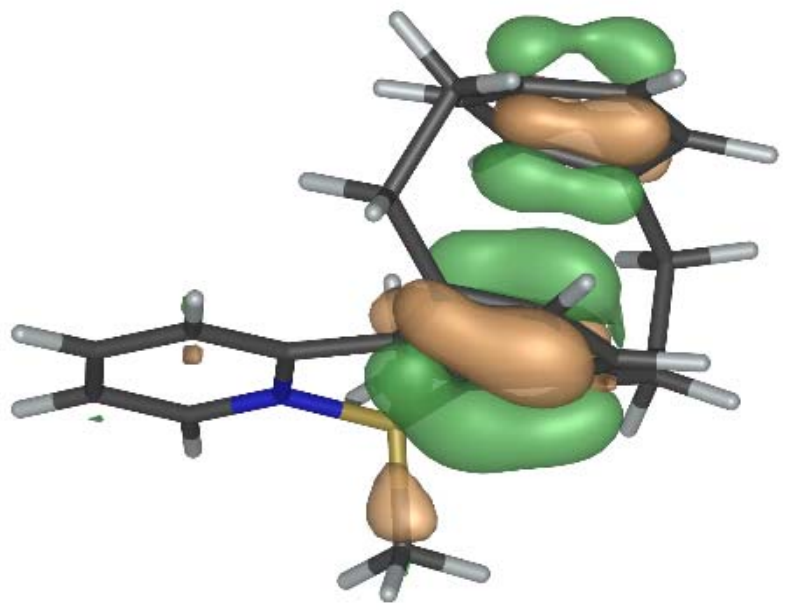

HOMO
ε = -7.23 eV

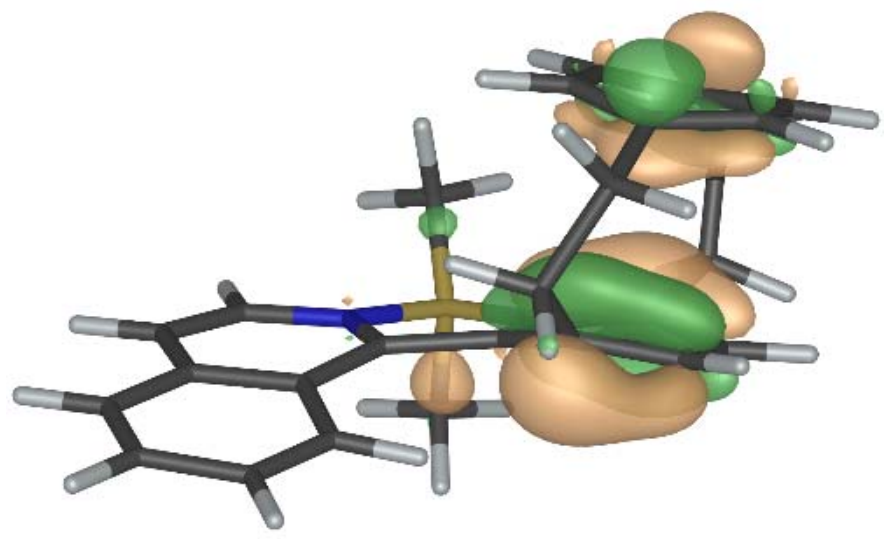

HOMO-1
ε = -7.30 eV

Energy (nm)		Oscillator strength	Major contributions	
1	308.65	0.1885	HOMO → LUMO	(75%)
			HOMO-1 → LUMO	(10%)
2	299.32	0.1880	HOMO-1 → LUMO	(76%)
			HOMO → LUMO	(5%)
			HOMO → LUMO+1	(5%)
3	265.52	0.0578	HOMO-2 → LUMO	(55%)
			HOMO → LUMO+1	(24%)

Figure S31. Frontier contour diagrams for **1b** and table of its three lowest excitations.

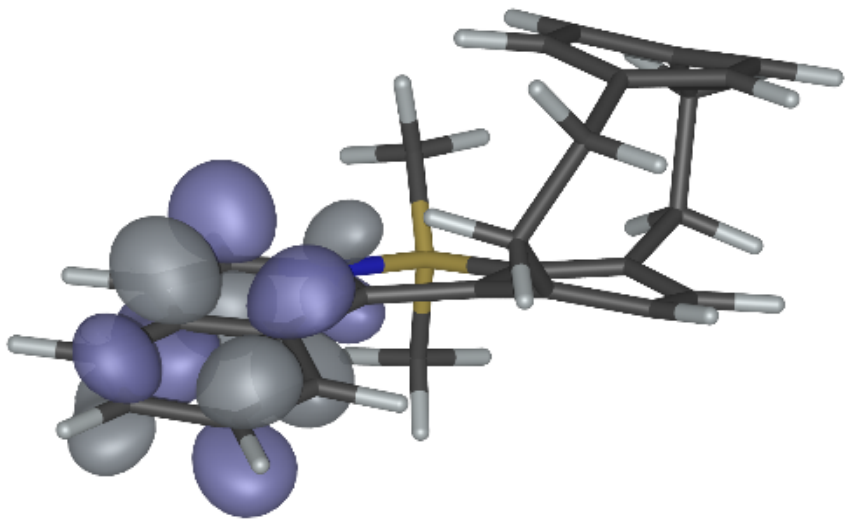

LUMO+1
ε = -0.08 eV

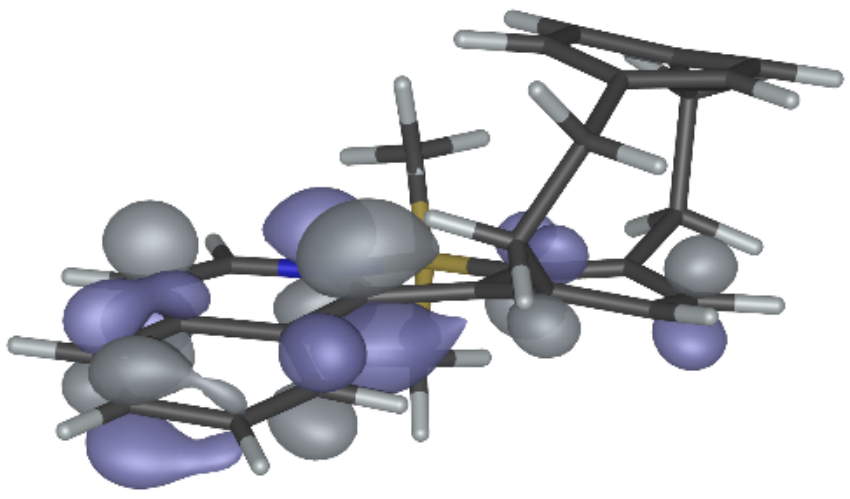

LUMO
ε = -1.09 eV

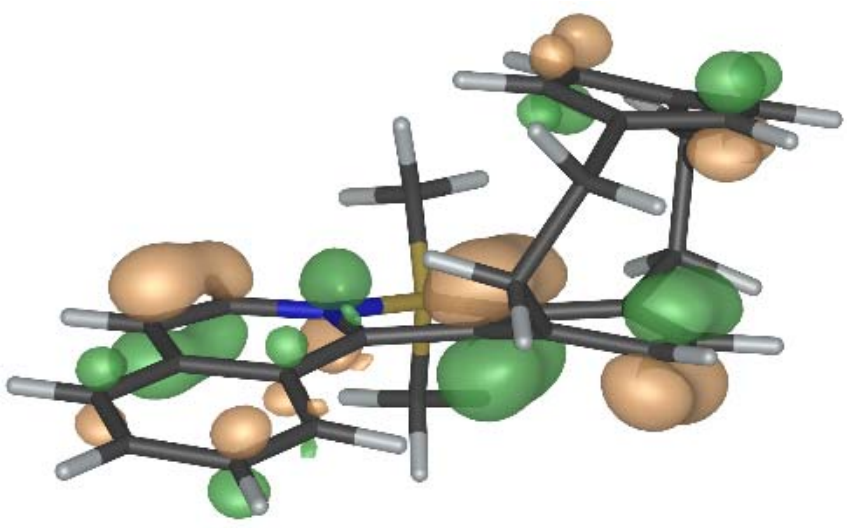

HOMO
ε = -7.21 eV

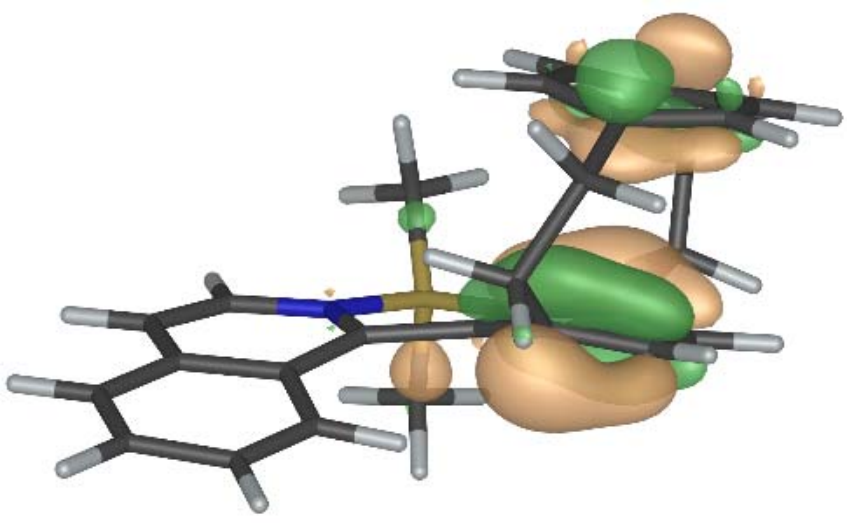

HOMO-1
ε = -7.29 eV

Energy (nm)		Oscillator strength	Major contributions	
1	344.53	0.3863	HOMO → LUMO	(92%)
2	325.51	0.0672	HOMO-1→ LUMO	(83%)
			HOMO-2→ LUMO	(12%)
3	277.41	0.0605	HOMO → LUMO+2	(51%)
			HOMO-3→ LUMO+1	(11%)
			HOMO-4→ LUMO	(10%)
			HOMO-6→ LUMO	(9%)

Figure S32. Frontier contour diagrams for **1c** and table of its three lowest excitations.

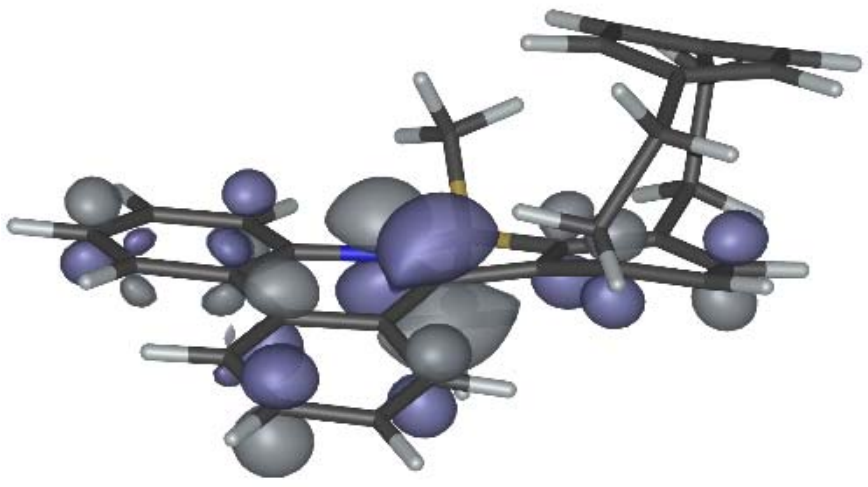

LUMO+1
ε = -0.37 eV

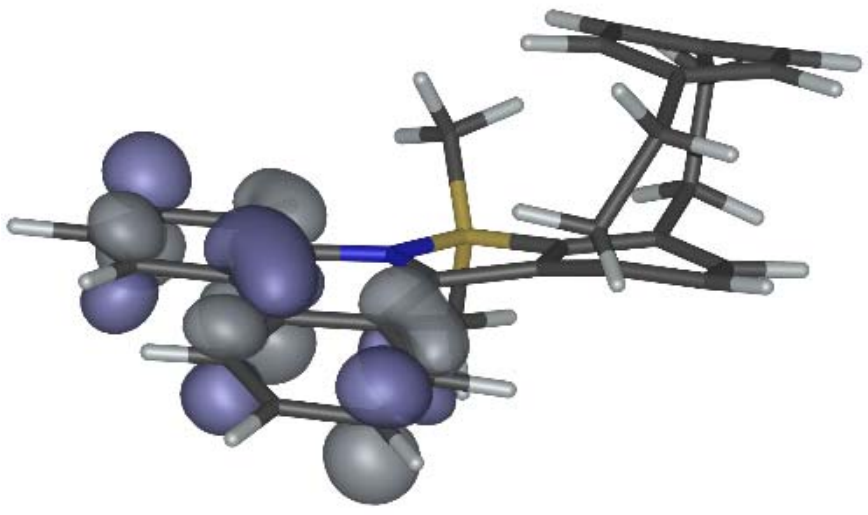

LUMO
ε = -1.23 eV

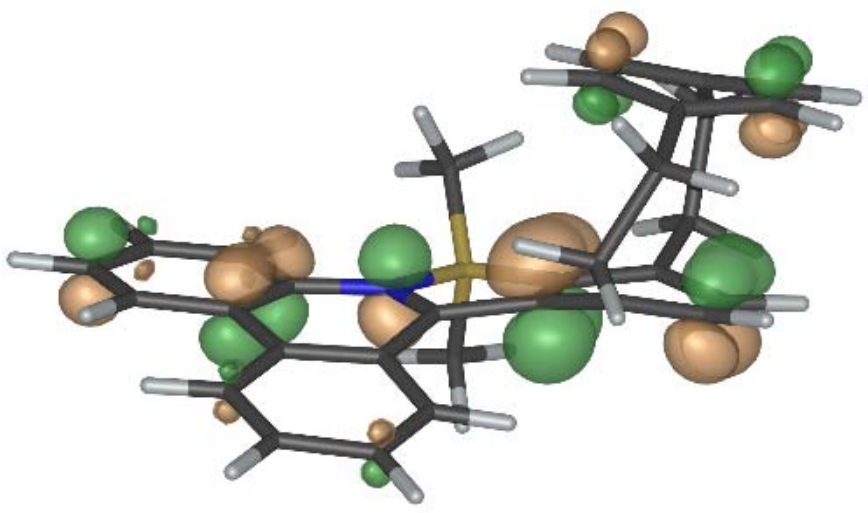

HOMO
ε = -7.26 eV

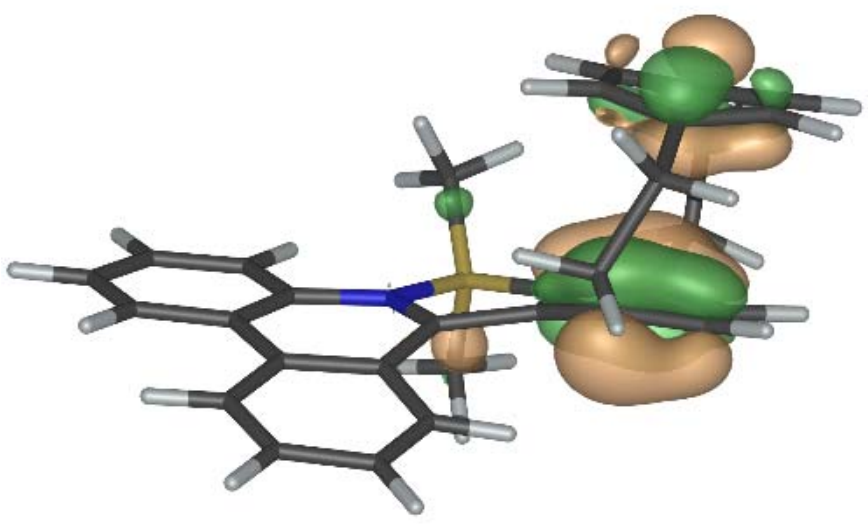

HOMO-1
ε = -7.30 eV

Energy (nm)		Oscillator strength	Major contributions	
1	350.20	0.4297	HOMO → LUMO	(92%)
2	334.91	0.1004	HOMO-1→ LUMO	(84%)
			HOMO-2→ LUMO	(6%)
3	296.38	0.0047	HOMO-3→ LUMO	(57%)
			HOMO → LUMO+1	(22%)
			HOMO-4→ LUMO+1	(7%)

7 NICS

DFT and TD-DFT calculations were performed to obtain a more detailed information on the impact of the azaborole ring on electronic properties of the aromatic conjugated systems in **1a–c**. To the best of our knowledge no such steps have been undertaken so far.

First, NICS(0) calculations indicated low aromaticity of the azaborole ring (ring C) in all cases (1.17–1.65). These values are larger than in those for the six-membered 1,2-azaborine ring incorporated in helical systems (0.6-0.8).[13] Second, NICS(0) values (-8.57 – -8.67) for aromatic ring A of the pCp scaffold almost correspond to previously reported value for the pristine pCp (ca -9).[14] For aromatic rings B these values indicate somewhat lower aromaticity (-7.48 – -7.52), which results obviously from neighboring the azaborole ring.

Table S9. Calculated NICS(0) values for **1a–c**.

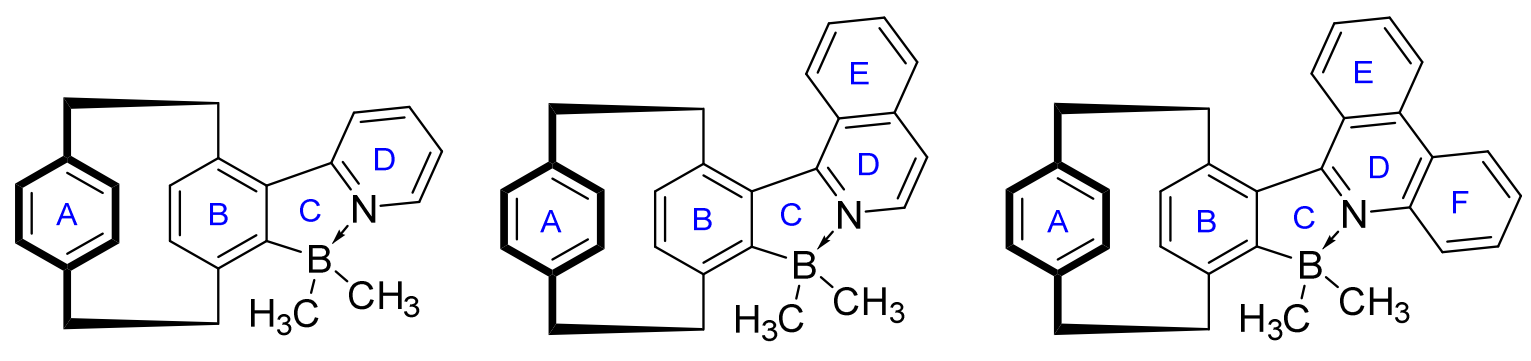

Compound/ring	NICS(0)					
	A	B	C	D	E	F
**1a**	-8.5690	-7.5220	1.1707	-5.3949		
**1b**	-8.6610	-7.4766	1.5455	-5.3949	7.8558	
**1c**	-8.6727	-7.4960	1.6502	-2.7754	-7.6613	-8.3060

[13] Yuan, K.; Volland, D.; Kirschner, S.; Uzelac, M.; Nichol, G. S.; Nowak-Krol', A.; Ingleson, M. J. *Chem. Sci.*, **2022**, *13*, 1136–1145.
[14] Poater, J.; Bofill, J. M.; Alemany, P.; Sola, M. *J. Org. Chem*. **2006**, *71*, 1700-1702.

8 Electrochemistry

Voltammetric measurements were performed using an Autolab PGSTAT204 potentiostat (Metrohm, Switzerland) controlled by Nova 2.1.8 software. A three-electrode system was employed, consisting of a boron-doped diamond (BDD) working disc electrode (geometric area 7.1 mm^2, BioLogic Science Instruments, France), a platinum wire counter electrode (ALS, Japan), and a non-aqueous Ag/Ag^+ reference electrode (ALS, Japan; Ag wire in 0.01 $mol \cdot L^{-1}$ $AgNO_3$ and 5 $mmol \cdot L^{-1}$ tetrabutylammonium hexafluorophosphate in acetonitrile). Prior to analysis, the working electrode was polished with an alumina suspension (particle size 0.5 µm; Elektrochemické detektory, Czech Republic) in acetonitrile.

The electrochemical behavior was investigated in acetonitrile (MeCN; ≥99.9%, Honeywell – Riedel-de Haën, Germany) containing 5 $mmol \cdot L^{-1}$ tetrabutylammonium hexafluorophosphate (Bu_4NPF_6; >99.0%, Merck – Supelco, Germany) as the supporting electrolyte. The concentration of the analyte was 0.25 $mmol \cdot L^{-1}$. All solutions were deoxygenated by purging with nitrogen gas prior to the measurements, and a nitrogen atmosphere was maintained during the experiments.

Cyclic voltammetry (CV) was performed at a scan rate of 100 $mV \cdot s^{-1}$. Differential pulse voltammetry (DPV) was conducted with the following parameters: scan rate 20 $mV \cdot s^{-1}$, pulse height 50 mV, pulse width 100 ms, and step 3 mV. All potentials were calibrated internally against the ferrocene/ferrocenium (Fc^+/Fc) redox couple (ferrocene 98%, Acros Organics, Belgium). CV and DPV measurements were performed at room temperature. The scans were initiated at approximately –1.0 V vs. Fc^+/Fc with the initial CV scan in the oxidative (anodic) direction up to +1.4 V, before reversing to –3.3 V vs. Fc^+/Fc.

The energies of the frontier molecular orbitals (HOMO and LUMO) and the electrochemical band gap (E_{gap}) were calculated from the oxidation (E_{ox}) and reduction (E_{red}) potentials relative to the internal standard, using the vacuum energy level of ferrocene as –4.8 eV.[15]

$$E_{HOMO}\ (eV) = -e(E_{ox} + 4.8)$$

$$E_{LUMO}\ (eV) = -e(E_{red} + 4.8)$$

$$E_{gap}\ (eV) = E_{ox} - E_{red}$$

(alternatively for band gap: $E_{gap} = |E_{HOMO} - E_{LUMO}|$)

[15] Pommerehne, J.; Vestweber, H.; Guss, W.; Mahrt, R. F.; Bässler, H.; Porsch, M.; Daub, J. Efficient two layer leds on a polymer blend basis. *Adv. Mat.* **1995**, *7*, 551–554.

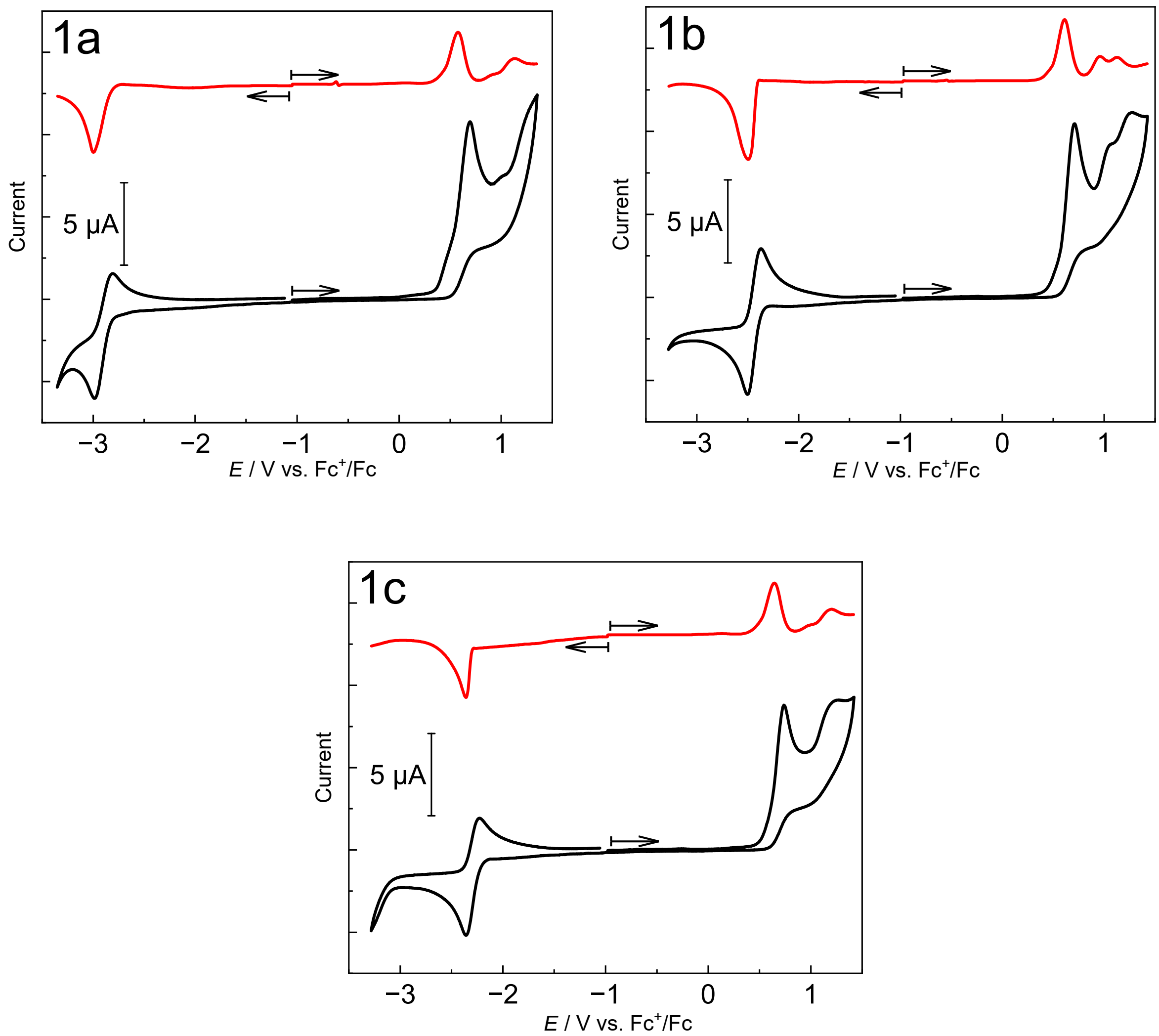

Figure S33. Cyclic (black line) and differential pulse (red line) voltammograms of 0.25 mmol·L^{-1} **1a**, **1b**, and **1c** at boron doped diamond electrode measured in acetonitrile with 5 mmol·L^{-1} Bu_4NPF_6 as supporting electrolyte. Voltammograms are plotted according to the IUPAC convention. Measurements were performed at room temperature. The initial potential was –1.0 V vs. Fc^+/Fc with the first scan in the anodic direction for CV. DPV curves were recorded as two separate scans (anodic and cathodic) starting from the respective initial potentials.

Table S10. Electrochemical properties of **1a–c**.

Cyclic voltammetry (values for onset potentials)

	$E^{ox,1}$ (V)	E^{red} (V)	E_{gap} (eV)	E_{HOMO} (eV)	E_{LUMO} (eV)
**1a**	0.47	-2.83	3.30	-5.27	-1.97
**1b**	0.54	-2.37	2.91	-5.34	-2.43
**1c**	0.58	-2.23	2.81	-5.38	-2.57

Cyclic voltammetry (values for peak potentials)

	$E^{ox,1}$ (V)	$E^{ox,3}$ (V)	E^{red} (V)	E_{gap} (eV)	E_{HOMO} (eV)	E_{LUMO} (eV)
**1a**	0.69	-	-2.99	3.68	-5.49	-1.81
**1b**	0.71	1.26	-2.50	3.21	-5.51	-2.30
**1c**	0.74	1.25	-2.36	3.09	-5.54	-2.44

Differential pulse voltammetry

	$E^{ox,1}$ (V)	$E^{ox,2}$ (V)	$E^{ox,3}$ (V)	E^{red} (V)	E_{gap} (eV)	E_{HOMO} (eV)	E_{LUMO} (eV)
**1a**	0.58	0.90	1.13	-3.00	3.58	-5.38	-1.80
**1b**	0.61	0.96	1.13	-2.50	3.11	-5.41	-2.30
**1c**	0.65	0.97	1.20	-2.36	3.01	-5.45	-2.44

Comments on electrochemistry measurements:

A reduced supporting electrolyte concentration of 5 mmol·L^{-1} was employed due to the limited solubility of the analytes. While cyclic voltammetry (CV) was used to assess the general redox behavior and reversibility of the processes, differential pulse voltammetry (DPV) was employed to obtain highly resolved peak potentials due to its superior sensitivity and to mitigate the resulting iR drop effects. The CV measurements display irreversible oxidation peaks in the narrow range of 0.69 – 0.74 V for all three compounds, suggesting that the HOMO energy level is localized on the common molecular backbone and is minimally affected by the variation of the aromatic system. In contrast, the reduction processes show a clear dependence on the structure. **1a** exhibits a quasi-reversible reduction peak at -2.99 V. Extending the π-conjugation from pyridine **1a** to quinoline **1b** and phenanthridine **1c** results in a significant positive shift of the reduction potential to -2.50 V and -2.36 V, respectively. This is in good accordance with the data measured by DPV. Consequently, the calculated electrochemical band gap narrows progressively from **1a** to **1c** (from 3.58 to 3.01 eV as determined by DPV, and from 3.30 to 2.81 eV derived from CV onset potentials). This trend implies that the expansion of the fused aromatic system primarily stabilizes the LUMO energy level, making the compounds more electron-deficient and the reduction more feasible.

9 Optimized xyz coordinates

Table S11. ESI GEOM 1A GS. Optimized xyz coordinates for **1a** in ground state.

C	-0.044225	0.013306	0.060433
C	0.090351	-0.051743	1.439710
C	1.351653	-0.072534	2.021493
C	2.429780	-0.340443	1.183755
C	2.295473	-0.280554	-0.189645
C	1.074450	0.052956	-0.763853
C	1.605231	0.475625	3.400303
C	1.655709	2.062492	3.426841
C	1.837461	2.676825	2.062077
C	3.075348	2.779724	1.425924
C	3.105285	2.839751	0.023044
C	1.939516	2.732851	-0.756817
C	0.756419	2.977286	-0.082947
C	0.708807	2.958220	1.303801
B	4.566981	2.829726	2.030422
C	5.118314	1.542293	2.841160
C	4.488413	2.932021	-0.436422
N	5.337226	2.909273	0.611428
C	6.653574	3.011943	0.447595
C	7.212815	3.151457	-0.799582
C	6.362251	3.213453	-1.896231
C	4.999057	3.112379	-1.718829
C	1.877047	2.067236	-2.117066
C	1.029760	0.739918	-2.106087
C	4.851178	4.214960	2.826205
H	-0.259982	2.996703	1.788133
H	4.332834	3.197385	-2.561409
H	-0.176299	2.981841	-0.633666
H	7.249202	2.985977	1.348999
H	5.907616	4.324045	3.092969
H	4.281295	4.230518	3.759322
H	4.558970	5.100045	2.253848
H	3.416600	-0.446102	1.611389
H	2.558250	0.095499	3.766108
H	0.835936	0.151788	4.102128
H	0.717858	2.420690	3.852729
H	2.452344	2.363006	4.106042
H	6.162299	1.690084	3.136456
H	5.075219	0.613198	2.269735
H	4.553614	1.386047	3.762449
H	6.766393	3.351653	-2.889872
H	-0.786440	0.081875	2.062555
H	8.283968	3.228530	-0.908115
H	3.184025	-0.331272	-0.808502
H	-1.023447	0.202838	-0.363112
H	2.879343	1.806341	-2.442221
H	1.460555	2.729668	-2.878315
H	-0.007419	0.971689	-2.347885
H	1.408705	0.103843	-2.908136

Table S12. ESI_GEOM_1A_EXC. Optimized xyz coordinates for **1a** in the first excited state.

C	0.451145	0.167845	-0.280083
C	0.408287	-0.022195	1.104336
C	1.581331	-0.070848	1.847993
C	2.784894	-0.160641	1.147211
C	2.825866	0.009347	-0.239713
C	1.665602	0.298927	-0.949179
C	1.586927	0.253383	3.318490
C	1.682123	1.819207	3.582981
C	1.752422	2.592778	2.295695
C	2.949292	2.727863	1.584444
C	2.906085	2.909028	0.118053
C	1.669310	2.994823	-0.551915
C	0.554009	3.158873	0.261150
C	0.582377	2.996983	1.665784
B	4.482849	2.878592	2.070529
C	5.091340	1.910986	3.218236
C	4.223253	2.802918	-0.398548
N	5.136725	2.625059	0.640413
C	6.410471	2.324792	0.377347
C	6.894494	2.224578	-0.906705
C	6.012692	2.483274	-1.984148
C	4.697251	2.774486	-1.727744
C	1.457406	2.594522	-1.993357
C	1.758127	1.064266	-2.243696
C	4.714203	4.455768	2.462559
H	-0.349720	3.032192	2.217006
H	4.019228	2.970748	-2.543491
H	-0.415539	3.272404	-0.212374
H	7.052554	2.186854	1.238068
H	5.779337	4.645546	2.635898
H	4.176992	4.703564	3.382743
H	4.373953	5.142410	1.681813
H	3.712665	-0.284955	1.691531
H	2.438494	-0.226390	3.801855
H	0.681124	-0.125517	3.791679
H	0.802191	2.129692	4.147075
H	2.557898	2.013366	4.197388
H	6.175845	2.048284	3.290841
H	4.908825	0.844599	3.064381
H	4.695471	2.173991	4.202034
H	6.372914	2.455004	-3.003352
H	-0.548755	-0.004353	1.613255
H	7.936180	1.993300	-1.071207
H	3.786128	0.043832	-0.741948
H	-0.475207	0.340552	-0.818002
H	2.035191	3.192447	-2.698395
H	0.408508	2.775172	-2.230092
H	1.047473	0.703204	-2.988061
H	2.759085	0.943750	-2.660437

Table S13. ESI_GEOM_1B_EXC. Optimized xyz coordinates for **1b** in the first excited state.

N	-1.245703	1.736359	-0.213699
C	0.817091	1.009693	0.750026
C	-0.153501	-0.071433	0.686737
C	-2.695509	-0.188609	0.204096
C	-1.392477	0.411876	0.222268
C	2.072240	0.744517	1.262062
C	1.358271	-1.537300	1.767159
C	1.478543	-1.172053	-1.838536
C	-3.044666	-1.317364	0.940938
C	0.238338	-1.416988	0.984093
C	1.850523	-2.309464	-1.130677
C	2.253801	-0.470454	1.926820
C	2.253249	-0.026253	-1.782251
C	-2.189959	2.299618	-0.934206
C	-3.707969	0.442682	-0.584637
C	3.418326	0.015579	-1.023032
C	-3.398227	1.674972	-1.201408
C	0.809925	3.238787	-0.934516
C	-0.285089	-2.612383	0.217611
C	3.136083	-2.342358	-0.608822
C	-4.316873	-1.868955	0.844522
C	3.914652	-1.192284	-0.554728
C	3.275798	1.603396	0.968960
C	-0.167696	3.333449	1.558814
C	-4.977543	-0.142805	-0.676818
C	-5.279139	-1.296126	0.023905
C	0.808343	-3.296902	-0.678144
C	3.891128	1.328371	-0.463767
B	0.133350	2.389016	0.265555
H	3.181904	-0.652326	2.455581
H	-2.324083	-1.761911	1.610478
H	1.672273	-2.519139	2.101055
H	1.803679	3.595547	-0.656767
H	3.480952	-3.234489	-0.100103
H	0.215511	4.132059	-1.153343
H	4.851034	-1.213552	-0.009833
H	0.910393	2.684800	-1.870071
H	0.507363	-1.129741	-2.317578
H	-1.096841	-2.291390	-0.428798
H	1.876797	0.884964	-2.224471
H	-0.700199	-3.371926	0.882952
H	-4.138154	2.186194	-1.800890
H	-4.554639	-2.754499	1.418868
H	-1.988249	3.297965	-1.299176
H	4.044257	1.401078	1.715461
H	3.030351	2.661872	1.038714
H	0.768337	3.713239	1.979209
H	-0.690553	2.799430	2.357755
H	-0.777512	4.200474	1.283577
H	-5.726083	0.333225	-1.297354
H	-6.261953	-1.740072	-0.054785
H	1.295863	-4.090520	-0.112127
H	0.296750	-3.771333	-1.517369
H	3.592218	2.134807	-1.131469

H 4.977767 1.358402 -0.380205

Table S14. ESI_GEOM_1B_EXC. Optimized xyz coordinates for **1b** in the first excited state.

N	-1.245703	1.736359	-0.213699
C	0.817091	1.009693	0.750026
C	-0.153501	-0.071433	0.686737
C	-2.695509	-0.188609	0.204096
C	-1.392477	0.411876	0.222268
C	2.072240	0.744517	1.262062
C	1.358271	-1.537300	1.767159
C	1.478543	-1.172053	-1.838536
C	-3.044666	-1.317364	0.940938
C	0.238338	-1.416988	0.984093
C	1.850523	-2.309464	-1.130677
C	2.253801	-0.470454	1.926820
C	2.253249	-0.026253	-1.782251
C	-2.189959	2.299618	-0.934206
C	-3.707969	0.442682	-0.584637
C	3.418326	0.015579	-1.023032
C	-3.398227	1.674972	-1.201408
C	0.809925	3.238787	-0.934516
C	-0.285089	-2.612383	0.217611
C	3.136083	-2.342358	-0.608822
C	-4.316873	-1.868955	0.844522
C	3.914652	-1.192284	-0.554728
C	3.275798	1.603396	0.968960
C	-0.167696	3.333449	1.558814
C	-4.977543	-0.142805	-0.676818
C	-5.279139	-1.296126	0.023905
C	0.808343	-3.296902	-0.678144
C	3.891128	1.328371	-0.463767
B	0.133350	2.389016	0.265555
H	3.181904	-0.652326	2.455581
H	-2.324083	-1.761911	1.610478
H	1.672273	-2.519139	2.101055
H	1.803679	3.595547	-0.656767
H	3.480952	-3.234489	-0.100103
H	0.215511	4.132059	-1.153343
H	4.851034	-1.213552	-0.009833
H	0.910393	2.684800	-1.870071
H	0.507363	-1.129741	-2.317578
H	-1.096841	-2.291390	-0.428798
H	1.876797	0.884964	-2.224471
H	-0.700199	-3.371926	0.882952
H	-4.138154	2.186194	-1.800890
H	-4.554639	-2.754499	1.418868
H	-1.988249	3.297965	-1.299176
H	4.044257	1.401078	1.715461
H	3.030351	2.661872	1.038714
H	0.768337	3.713239	1.979209
H	-0.690553	2.799430	2.357755
H	-0.777512	4.200474	1.283577
H	-5.726083	0.333225	-1.297354
H	-6.261953	-1.740072	-0.054785
H	1.295863	-4.090520	-0.112127
H	0.296750	-3.771333	-1.517369
H	3.592218	2.134807	-1.131469

H	4.977767	1.358402	-0.380205

Table S15. **ESI_GEOM_1C_GS**. Optimized xyz coordinates for **1c** in ground state.

N	1.296911	-0.925632	0.260236
C	-1.286996	1.618732	0.720842
C	-0.472508	0.464840	0.746522
C	3.529960	-0.284090	-0.426545
C	1.938828	1.379555	0.417108
C	0.944414	0.334993	0.430513
C	2.570391	-1.274495	-0.156969
C	-2.482892	1.504233	1.401573
C	3.222207	1.084676	-0.075413
C	1.689191	2.648058	0.967201
C	-2.349733	-0.912673	1.379835
C	-2.990702	0.260429	1.753434
C	-2.971903	1.591239	-1.554129
C	-1.012092	-0.809189	0.988767
C	4.175735	2.113471	-0.124883
C	-3.799186	-1.025429	-0.998765
C	-2.233452	0.504706	-2.009134
C	2.645843	3.626504	0.939652
C	-1.109549	2.771348	-0.252005
C	-4.257508	1.337176	-1.093413
C	-4.669170	0.041176	-0.817110
C	4.771348	-0.676208	-0.948759
C	2.897326	-2.623261	-0.355433
C	-3.184709	-2.161156	1.227368
C	3.888531	3.363717	0.357913
C	-2.644292	-0.784960	-1.737406
C	-2.292817	2.907710	-1.279289
C	5.068110	-1.997082	-1.163849
C	4.125939	-2.977503	-0.849449
C	-0.267407	-3.009407	-0.463053
C	0.548870	-2.647617	2.100210
C	-3.909632	-2.284453	-0.183544
B	0.094273	-1.941628	0.708517
H	-3.129935	2.368617	1.484700
H	0.744592	2.839558	1.448478
H	-3.991676	0.209732	2.165707
H	5.162528	1.919458	-0.516259
H	-1.252748	0.665597	-2.441866
H	2.444274	4.594638	1.376932
H	-0.191865	2.623841	-0.815188
H	-1.007592	3.728805	0.261537
H	-4.887668	2.164943	-0.790317
H	-5.613063	-0.116551	-0.308530
H	5.514397	0.070959	-1.183314
H	2.171499	-3.382474	-0.128830
H	-3.957455	-2.160124	1.996551
H	-2.583369	-3.054324	1.384259
H	4.641847	4.139203	0.320603
H	-1.983077	-1.606863	-1.969601
H	-3.030150	3.607726	-0.886463
H	-1.883166	3.355857	-2.186316
H	6.031577	-2.276968	-1.567361
H	4.358766	-4.022670	-1.002316

H	-1.320790	-3.281791	-0.431930
H	0.287471	-3.945478	-0.364691
H	-0.068809	-2.623985	-1.466278
H	-0.288338	-3.231976	2.492268
H	0.820755	-1.919920	2.870191
H	1.391396	-3.333375	1.979437
H	-3.467058	-3.116893	-0.729399
H	-4.954954	-2.538216	-0.006985

Table S16. ESI_GEOM_1C_EXC. Optimized xyz coordinates for **1c** in the first excited state.

N	1.279040	-0.975290	0.315125
C	-1.267749	1.674664	0.661623
C	-0.451722	0.496382	0.704553
C	3.477659	-0.317821	-0.442253
C	1.945345	1.377978	0.464187
C	0.932182	0.370037	0.461089
C	2.487698	-1.314749	-0.142839
C	-2.439325	1.589557	1.368322
C	3.231366	1.050861	-0.029946
C	1.740732	2.658966	0.996597
C	-2.360027	-0.854568	1.373242
C	-2.967312	0.347726	1.748196
C	-2.900445	1.593386	-1.612342
C	-1.042236	-0.800107	0.958337
C	4.225899	2.030984	-0.041106
C	-3.779950	-0.995369	-0.980223
C	-2.180056	0.478139	-2.028488
C	2.740240	3.607316	0.975818
C	-1.050262	2.790513	-0.336830
C	-4.181276	1.378840	-1.126252
C	-4.617075	0.097262	-0.808225
C	4.670946	-0.722861	-1.040291
C	2.826670	-2.681630	-0.348189
C	-3.221636	-2.087642	1.268128
C	3.986438	3.302638	0.438977
C	-2.617267	-0.797694	-1.718694
C	-2.203934	2.909734	-1.397186
C	4.946026	-2.048554	-1.286557
C	4.020222	-3.031286	-0.907144
C	-0.302745	-3.062264	-0.390771
C	0.430606	-2.647737	2.180440
C	-3.931982	-2.235262	-0.144913
B	0.060703	-1.959326	0.753469
H	-3.071268	2.464104	1.464881
H	0.795768	2.896204	1.459474
H	-3.949215	0.323958	2.205592
H	5.212510	1.793804	-0.410431
H	-1.201567	0.606855	-2.476382
H	2.554379	4.588210	1.393000
H	-0.118112	2.610845	-0.866025
H	-0.944645	3.759350	0.154609
H	-4.794004	2.226870	-0.845366
H	-5.562262	-0.026602	-0.293141
H	5.407260	0.018555	-1.312764
H	2.118419	-3.439604	-0.067134
H	-3.998151	-2.042957	2.031937
H	-2.638197	-2.985980	1.457756
H	4.771208	4.046050	0.419907
H	-1.977007	-1.639954	-1.936338
H	-2.933231	3.644587	-1.056881
H	-1.765434	3.298474	-2.317645
H	5.879212	-2.331207	-1.754050
H	4.250363	-4.076626	-1.065049
H	-1.363922	-3.306933	-0.367203

H	0.224462	-4.009152	-0.249899
H	-0.078697	-2.717557	-1.403969
H	-0.425083	-3.220960	2.549739
H	0.680730	-1.909589	2.948127
H	1.272224	-3.342257	2.103644
H	-3.499818	-3.088326	-0.665975
H	-4.984894	-2.461150	0.022794

10 Copies of ^{1}H NMR and ^{13}C NMR spectra

rac-4-Formyl[2.2]paracyclophane (*rac*-2)

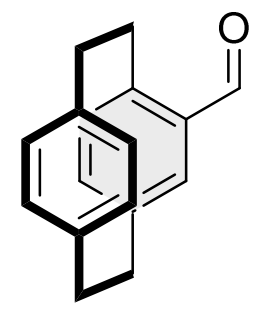

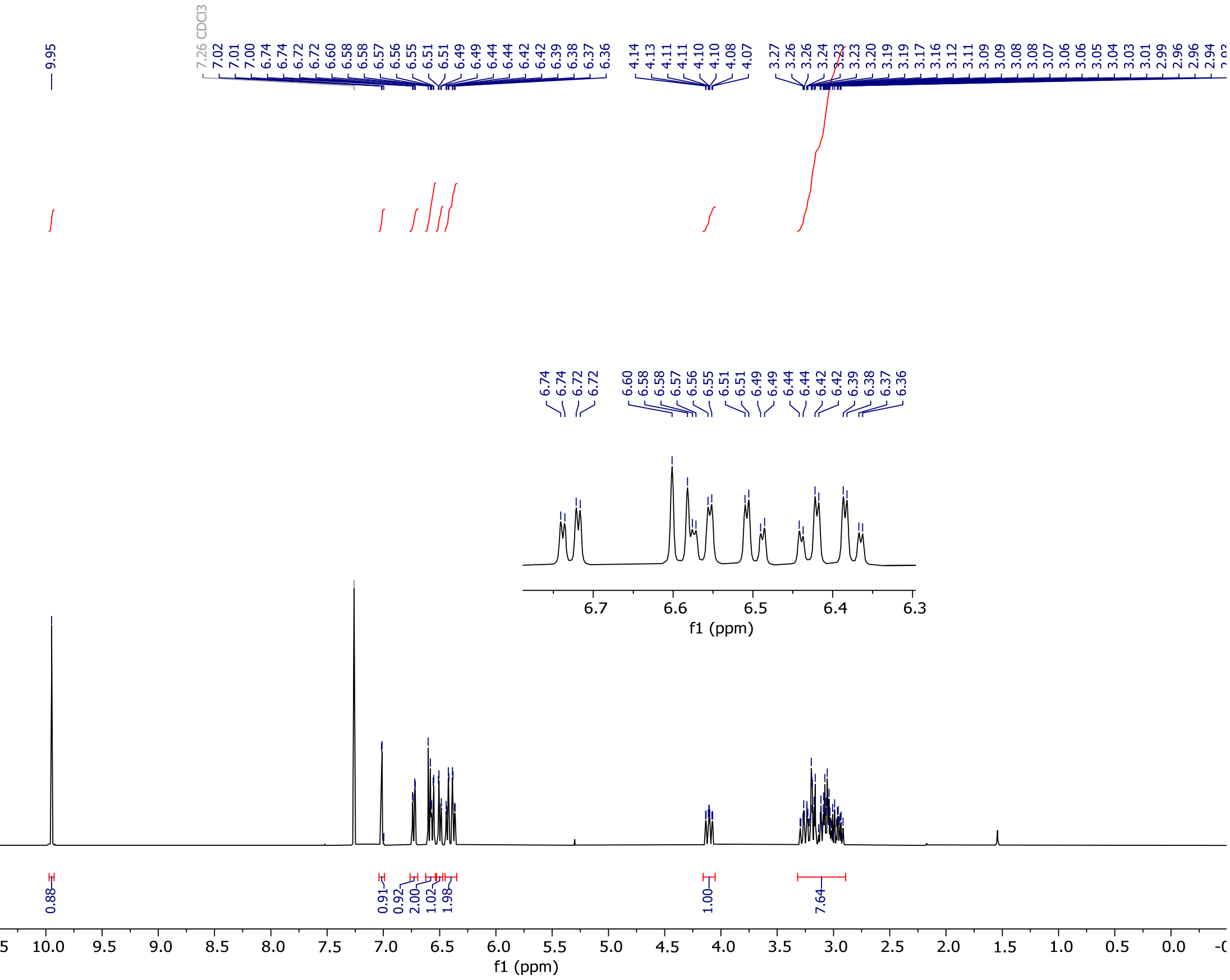

^{1}H NMR (400 MHz, $CDCl_3$)

***rac*-4-Hydroxy[2.2]paracyclophane (*rac*-3)**

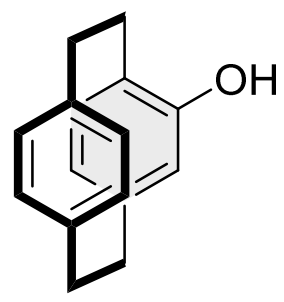

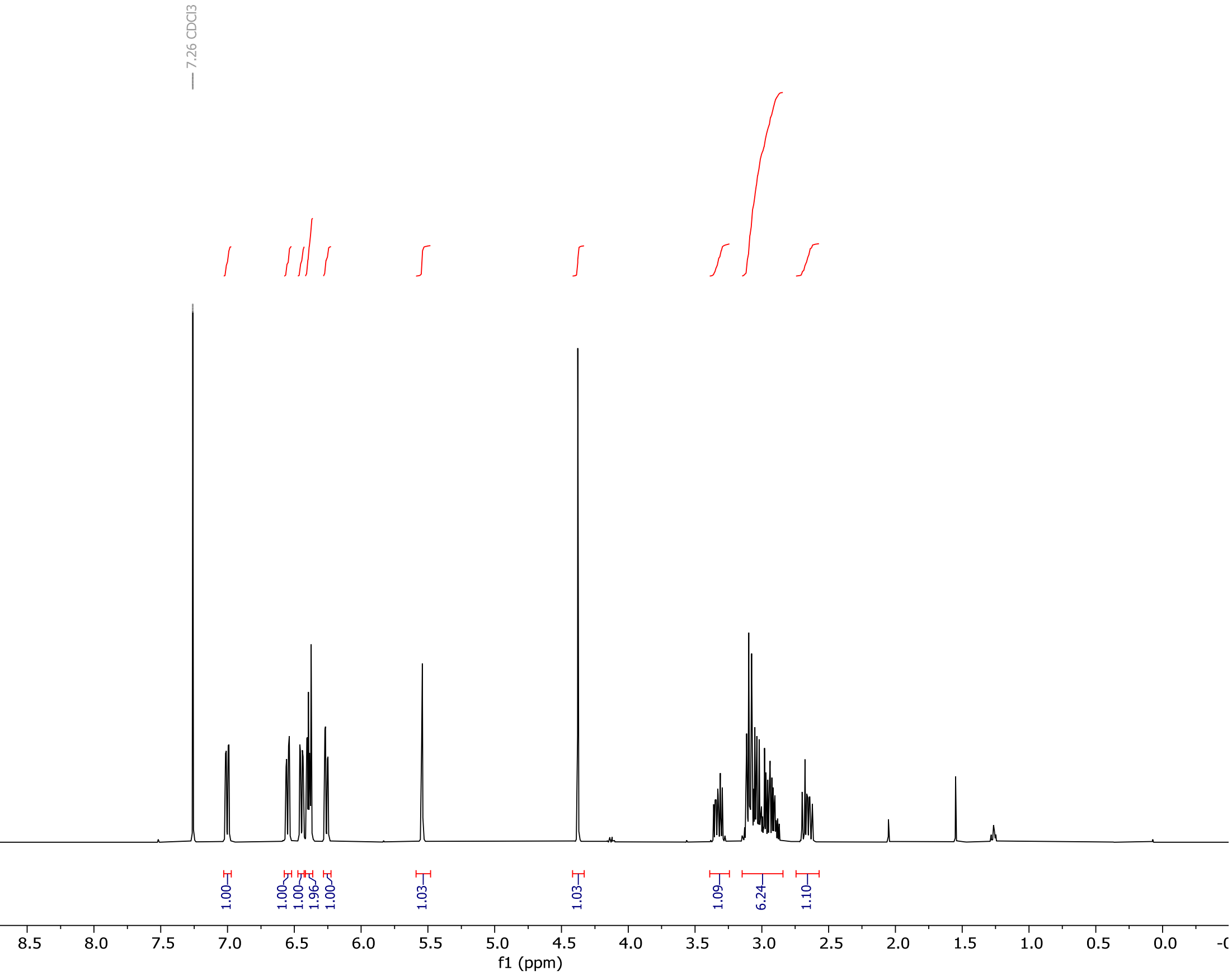

^{1}H NMR (400 MHz, $CDCl_3$)

rac-1,4(1,4)-Dibenzenacyclohexaphane-1^2-yl trifluoromethanesulfonate (_rac_-4).

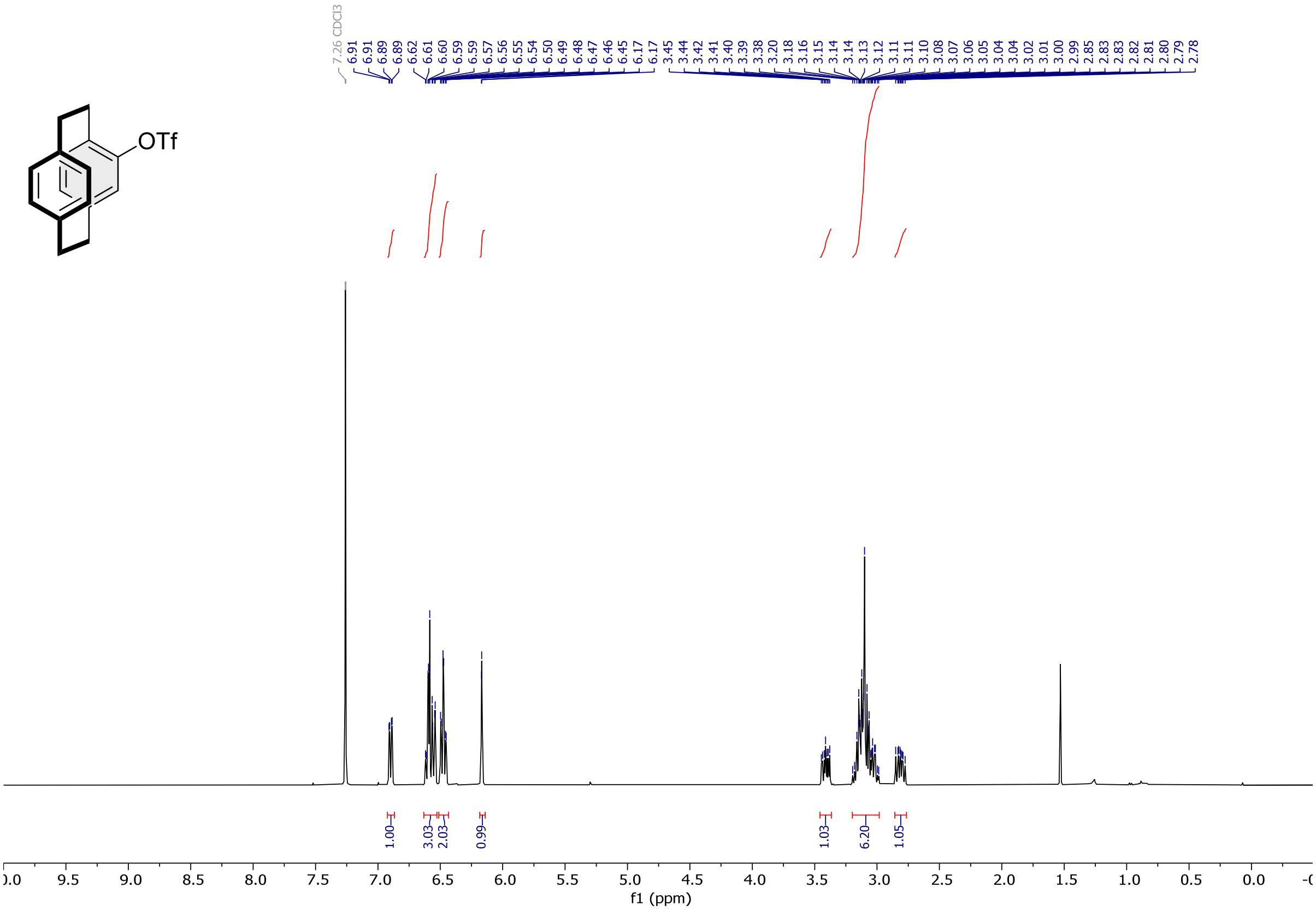

^{1}H NMR (400 MHz, $CDCl_3$)

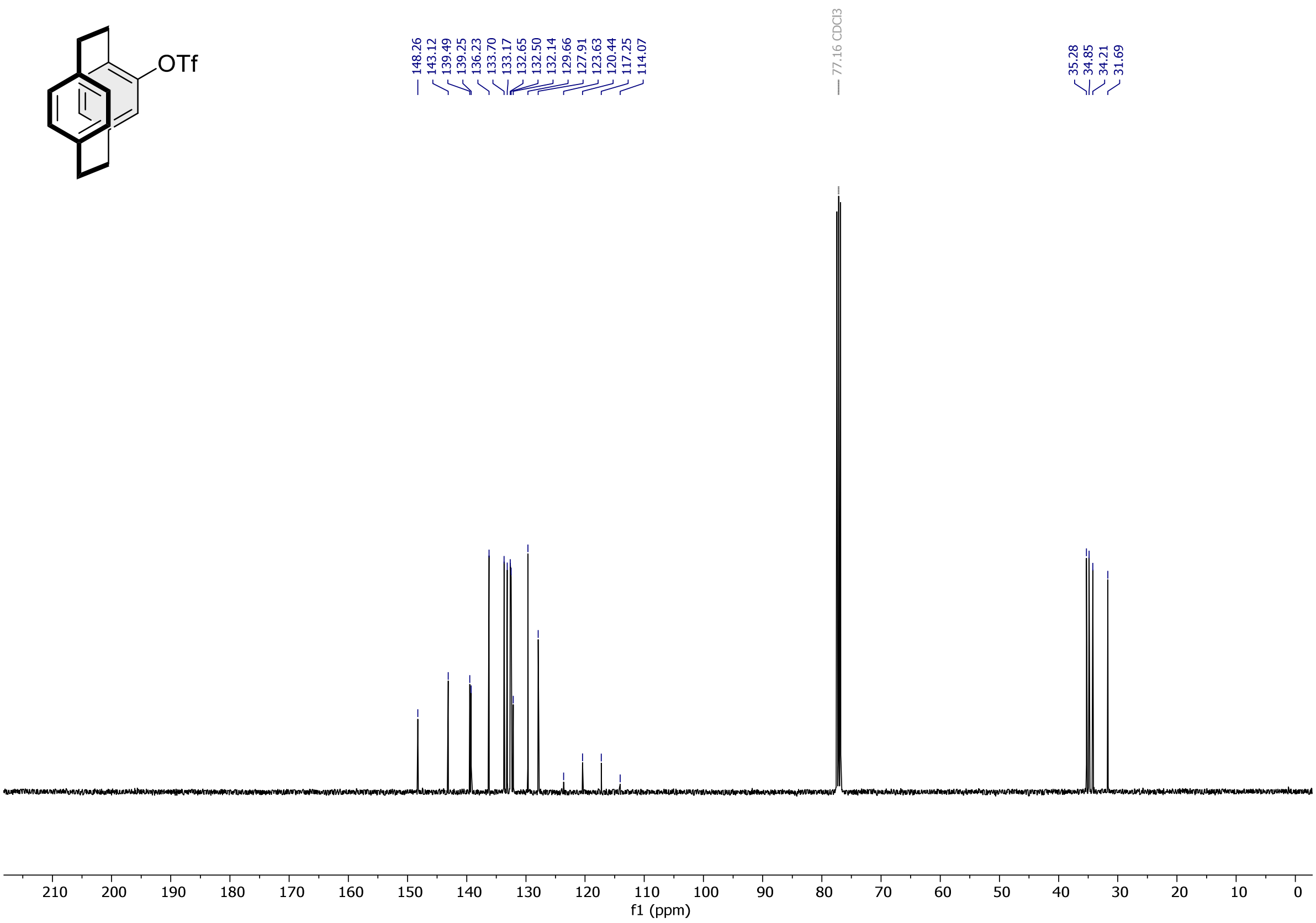

$^{13}C\{^1H\}$ NMR (101 MHz, $CDCl_3$)

rac-1,4(1,4)-dibenzenacyclohexaphane-1²-carbonyl chloride (_rac_-7)

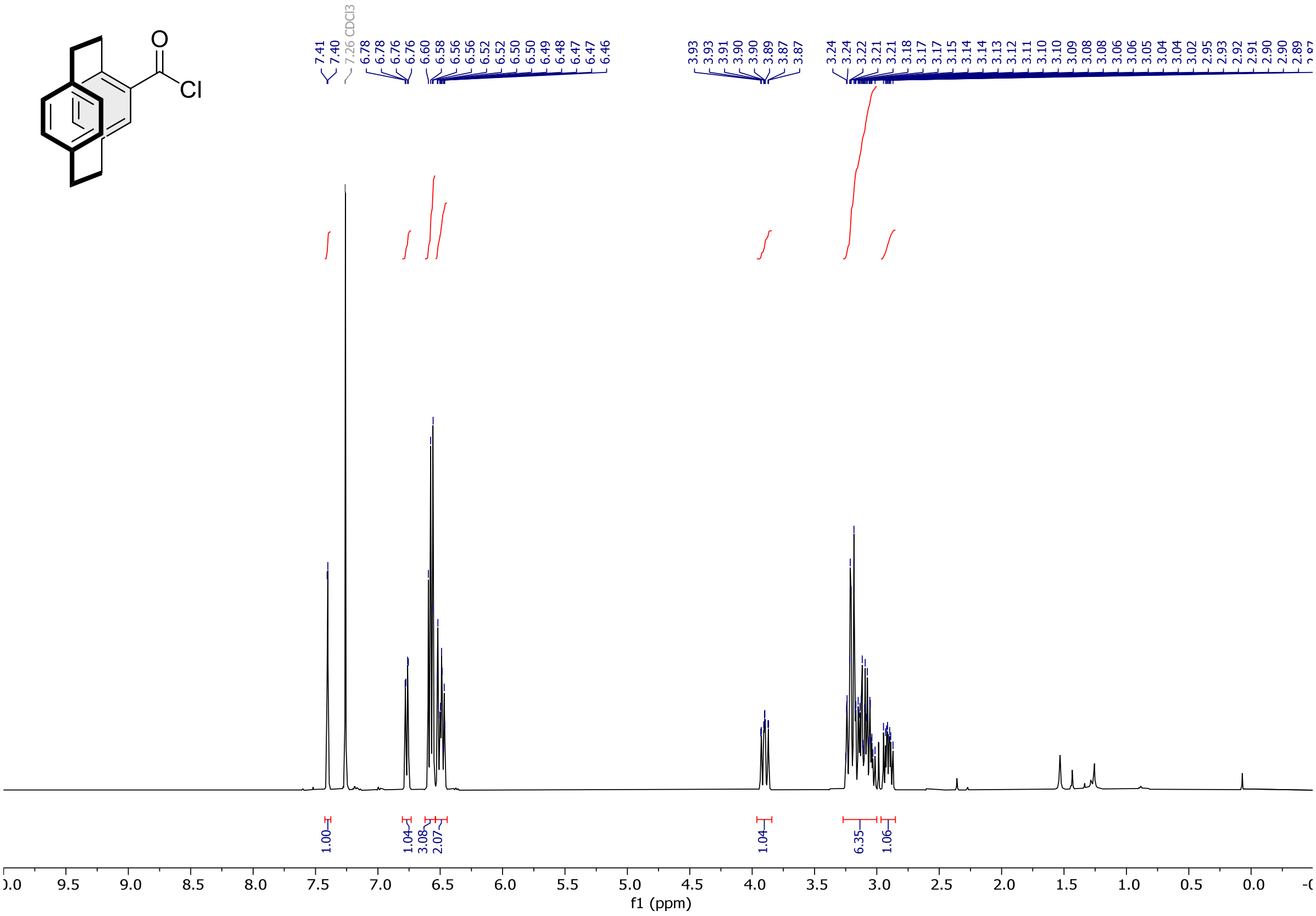

^{1}H NMR (400 MHz, CDCl$_3$)

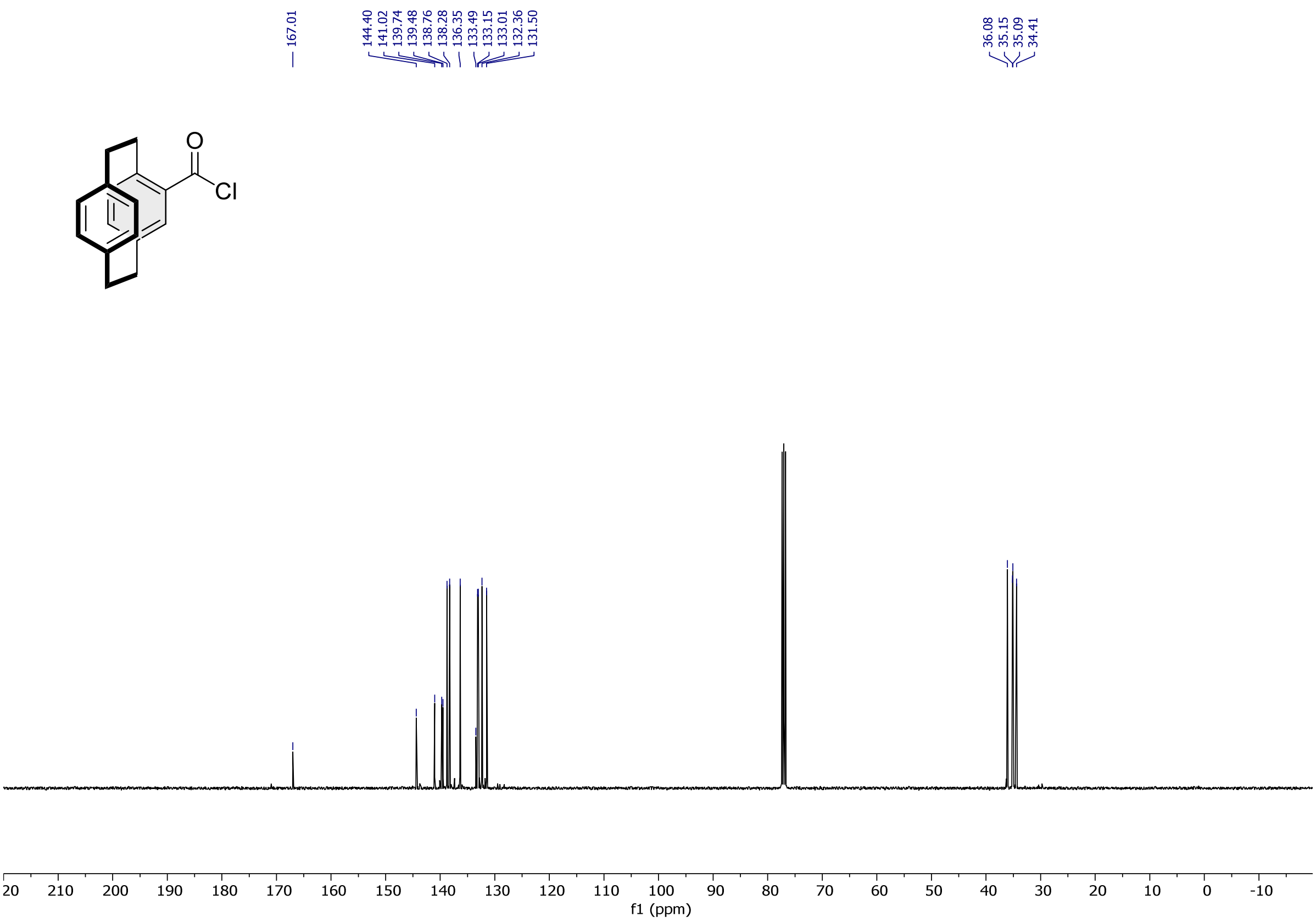

^{13}C{^{1}H} NMR (101 MHz, CDCl$_3$)

rac-4-bromo[2.2]paracyclophane (*rac*-5)

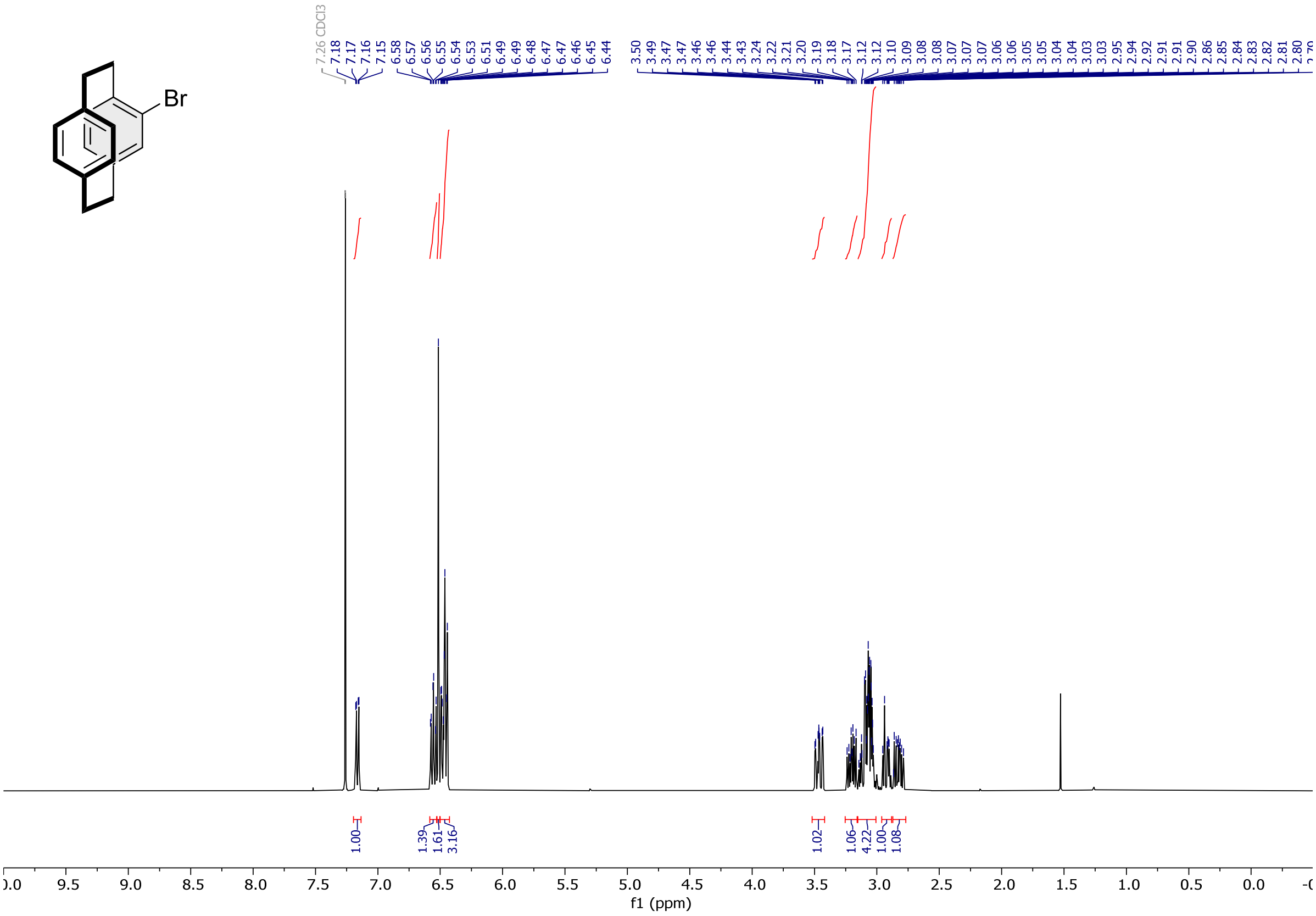

^{1}H NMR (400 MHz, $CDCl_3$)

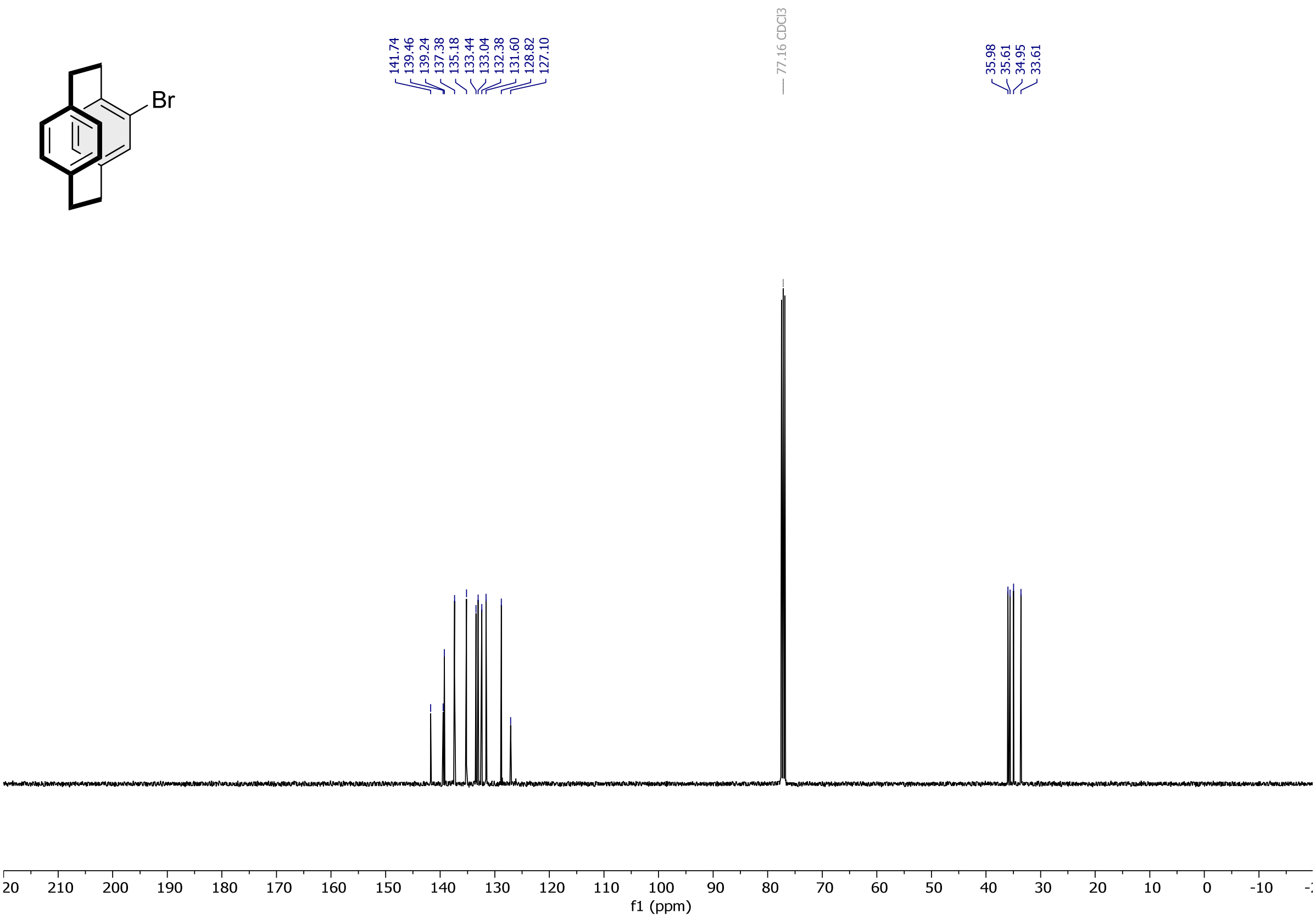

^{13}C{^{1}H} NMR (101 MHz, $CDCl_3$)

rac-2-(1,4(1,4)-dibenzenacyclohexaphane-1²-yl)pyridine (_rac_-9a)

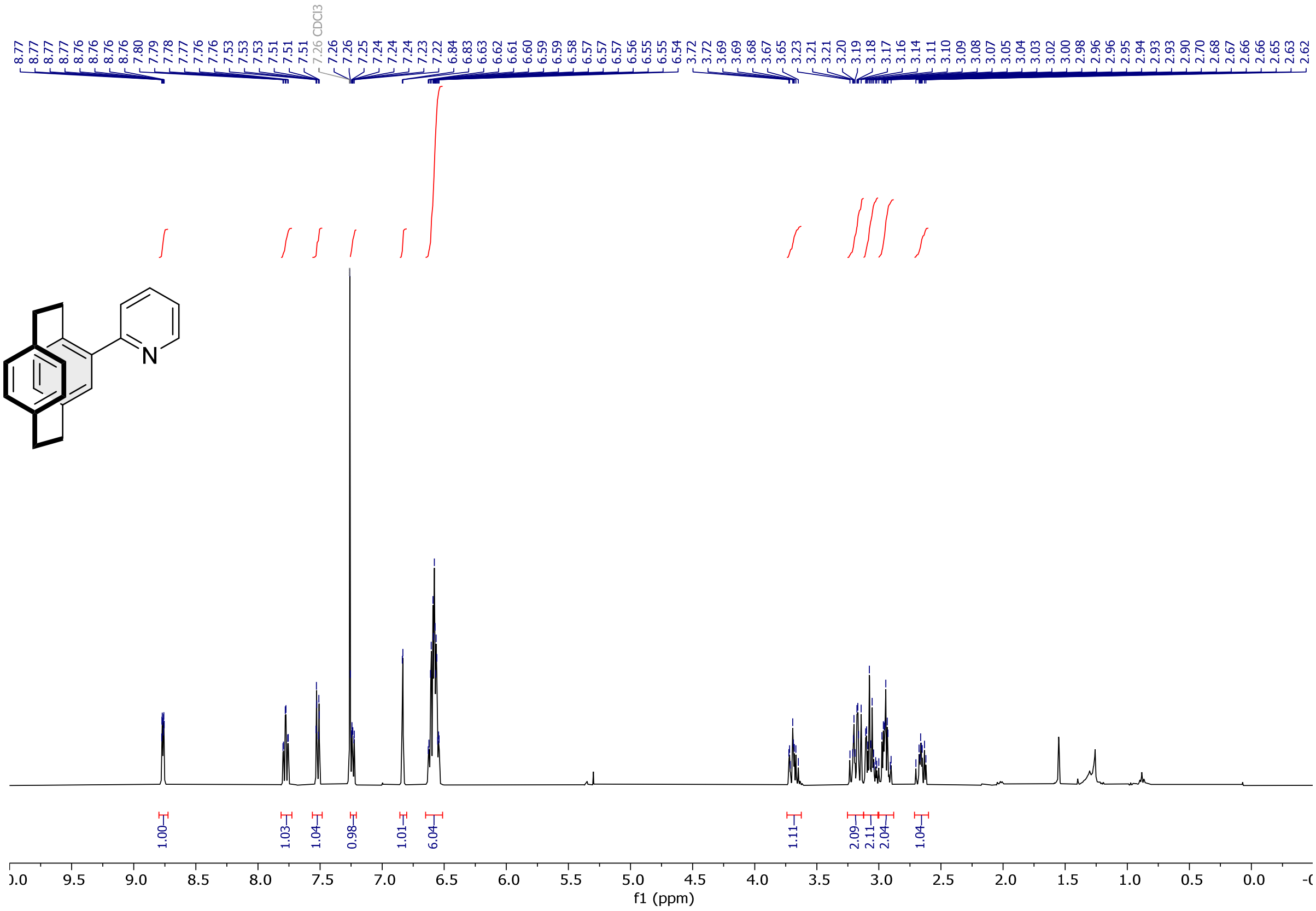

^{1}H NMR (400 MHz, CDCl$_3$)

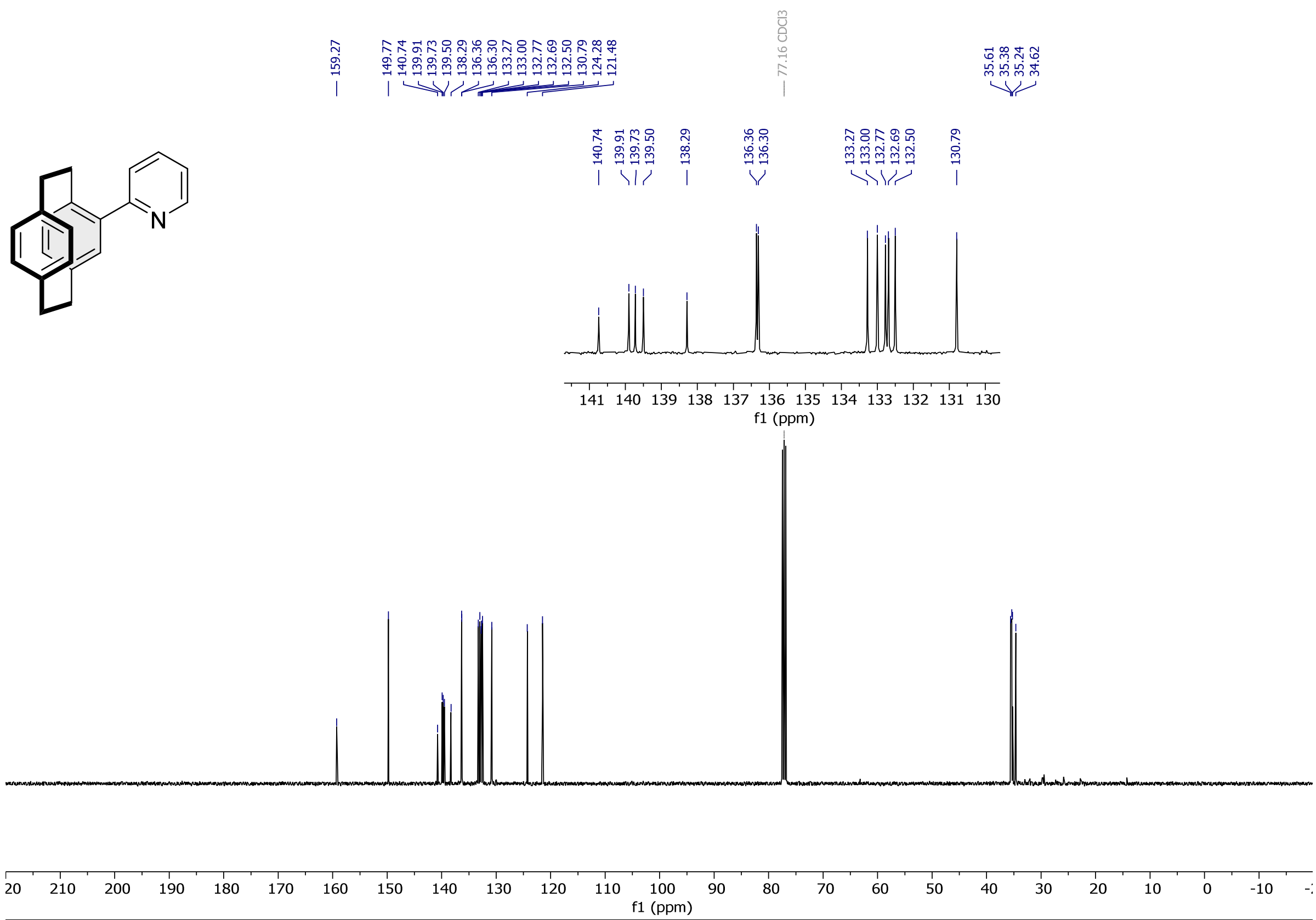

^{13}C{^{1}H} NMR (101 MHz, CDCl$_3$)

rac-1-(1,4(1,4)-dibenzenacyclohexaphane-1²-yl)isoquinoline (rac-9b)

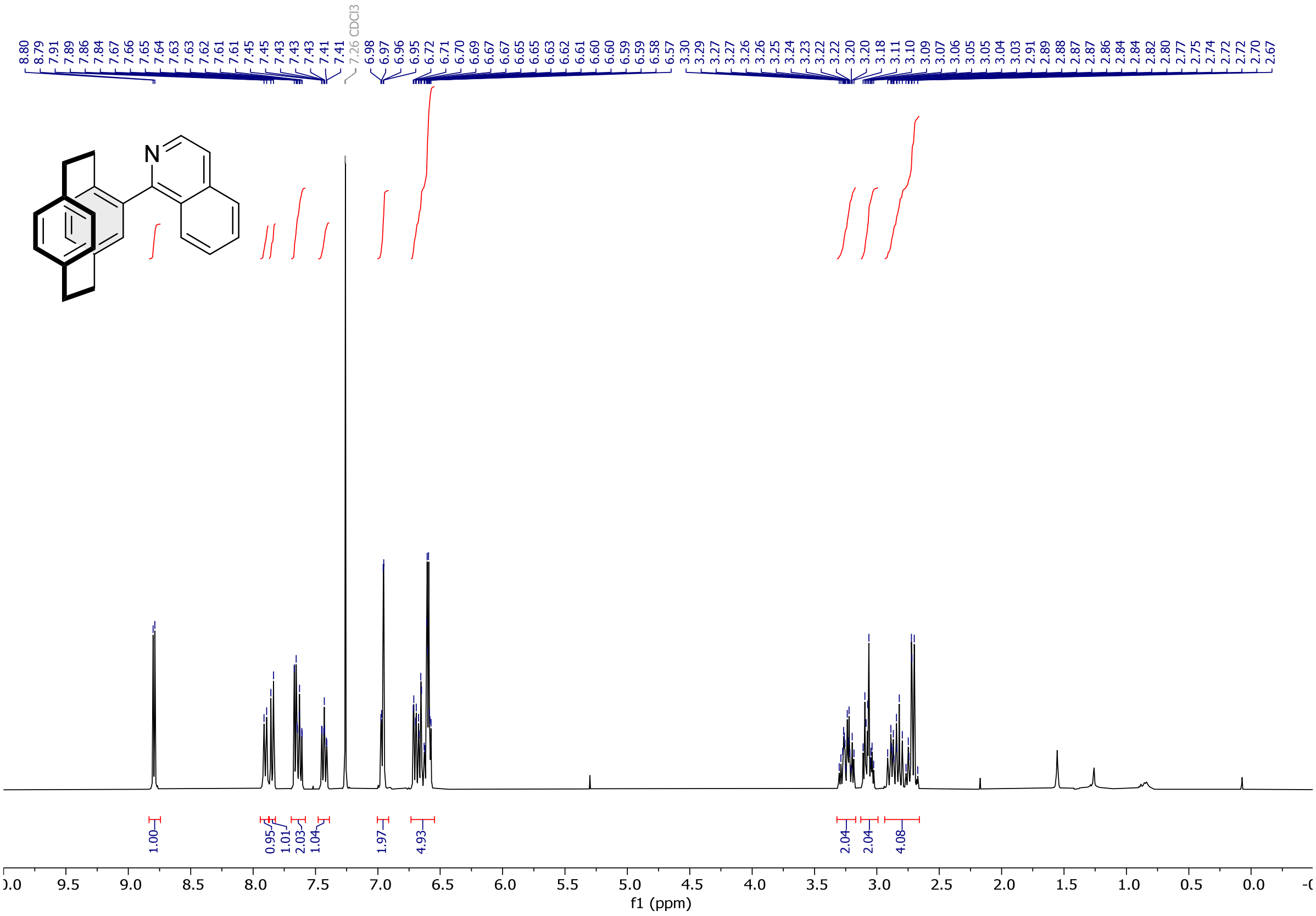

^1H NMR (400 MHz, $CDCl_3$)

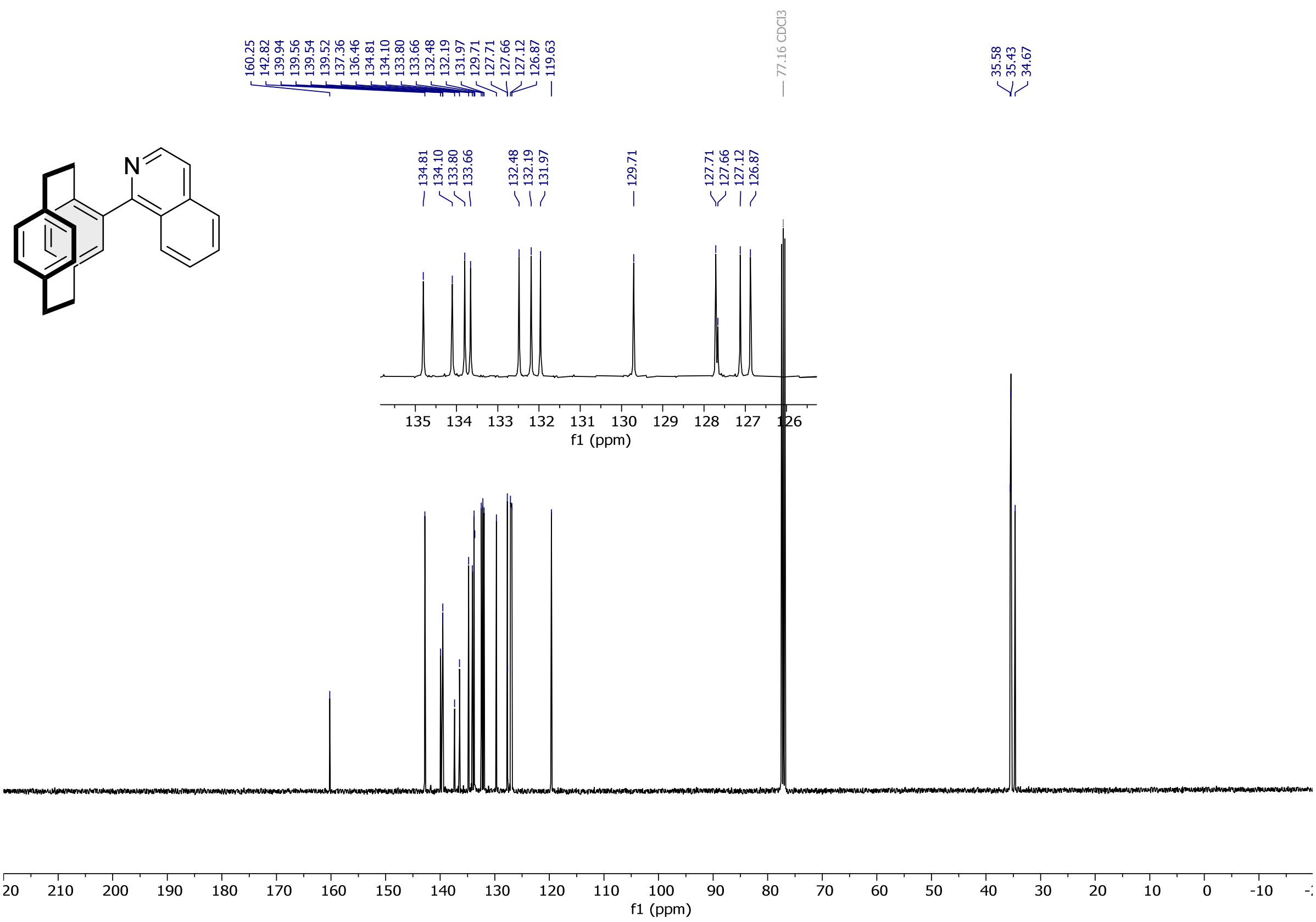

$^{13}C\{^1H\}$ NMR (101 MHz, $CDCl_3$)

***rac*-6-(1,4(1,4)-dibenzenacyclohexaphane-1²-yl)phenanthridine (*rac*-9c)**

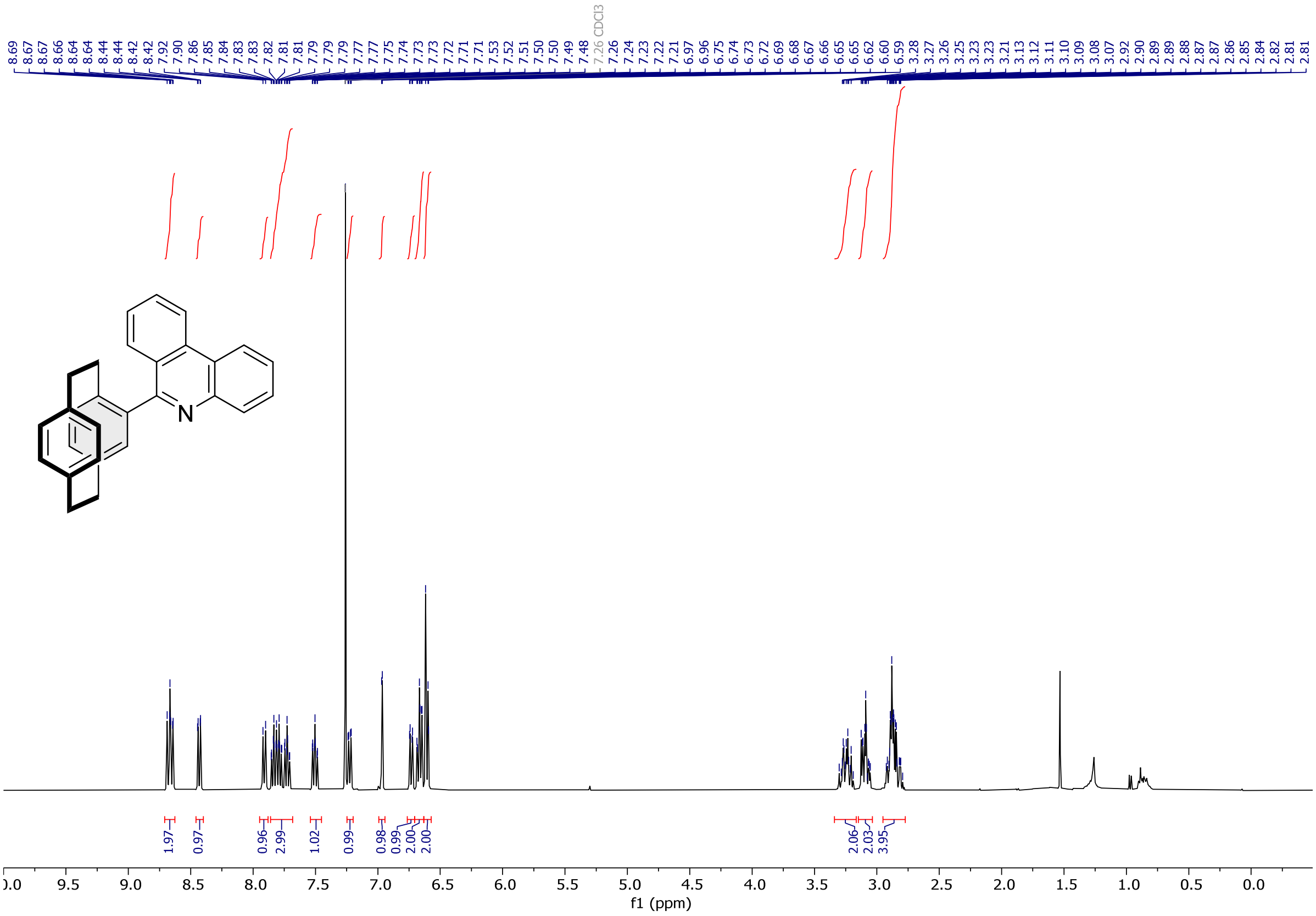

^{1}H NMR (400 MHz, $CDCl_3$)

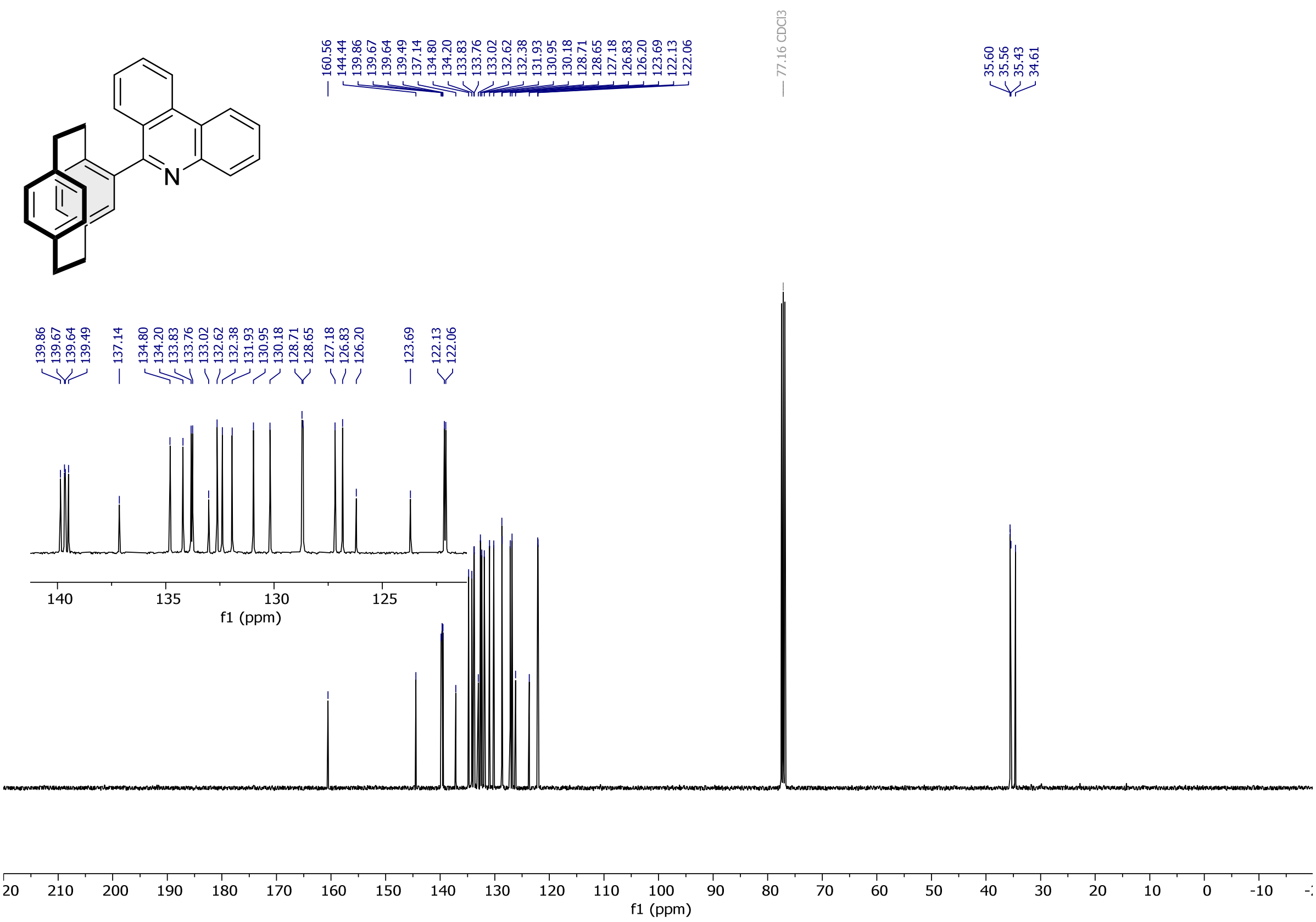

^{13}C{^{1}H} NMR (101 MHz, $CDCl_3$)

rac-1^6,1^6-dimethyl-1^6_H_-$1^5\lambda^4$,$1^6\lambda^4$-1(7,10)-$5\lambda^4$-benzo[3,4][1,2]azaborolo[1,5-_a_]pyridina-4(1,4)-benzenacyclohexaphane (_rac_-1a)

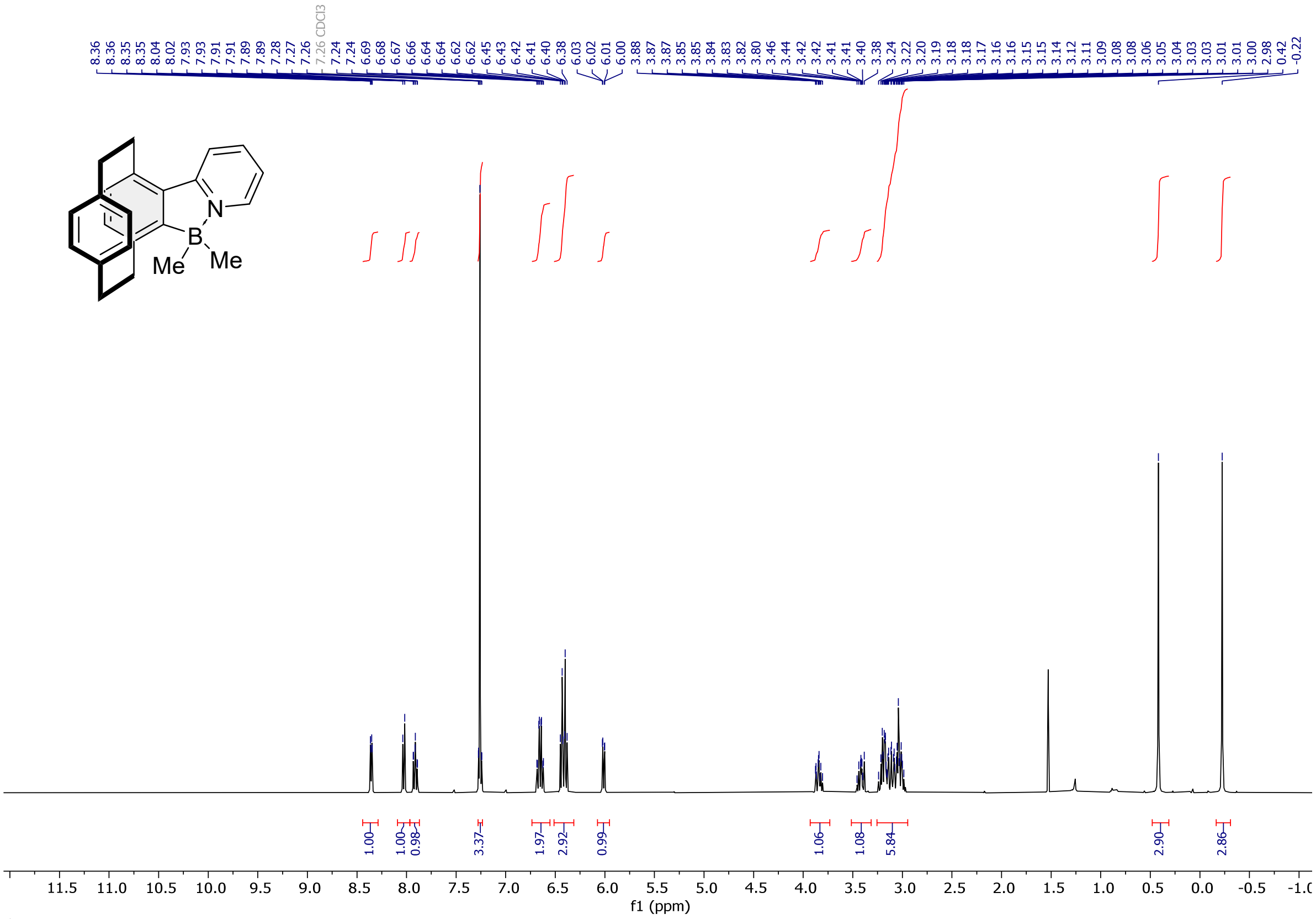

^{1}H NMR (400 MHz, $CDCl_3$)

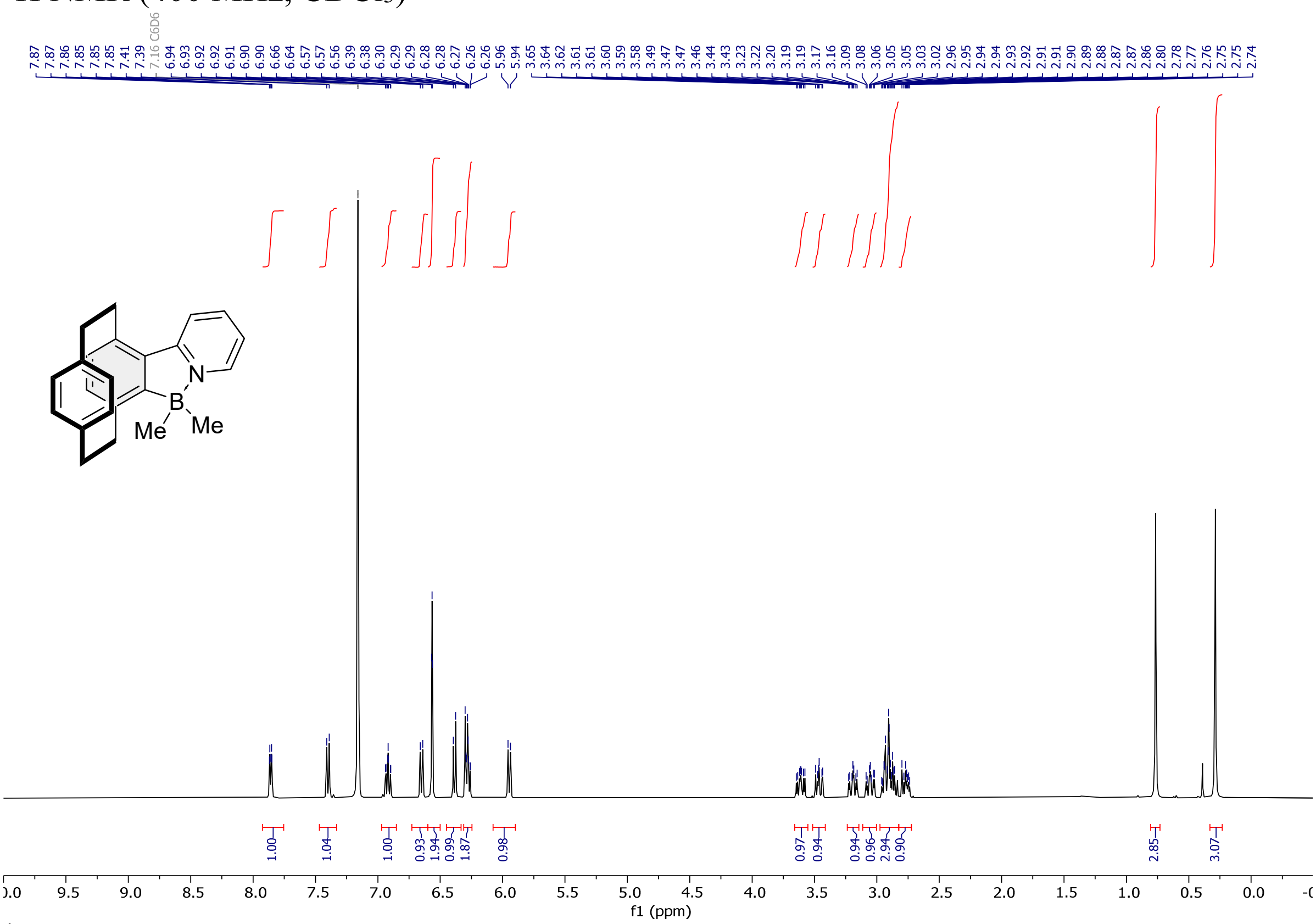

^{1}H NMR (400 MHz, C_6D_6)

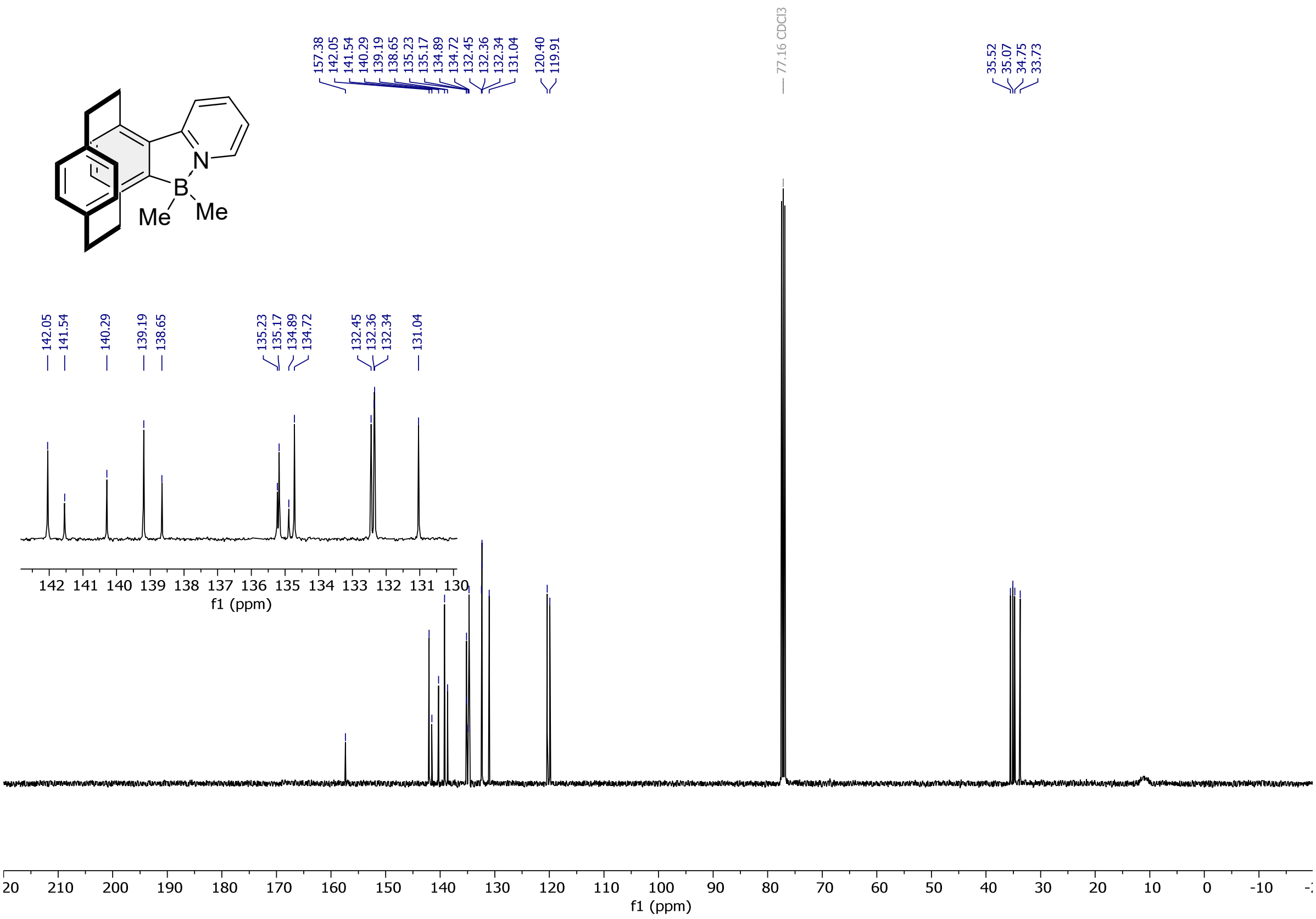

$^{13}C\{^{1}H\}$ NMR (101 MHz, $CDCl_3$)

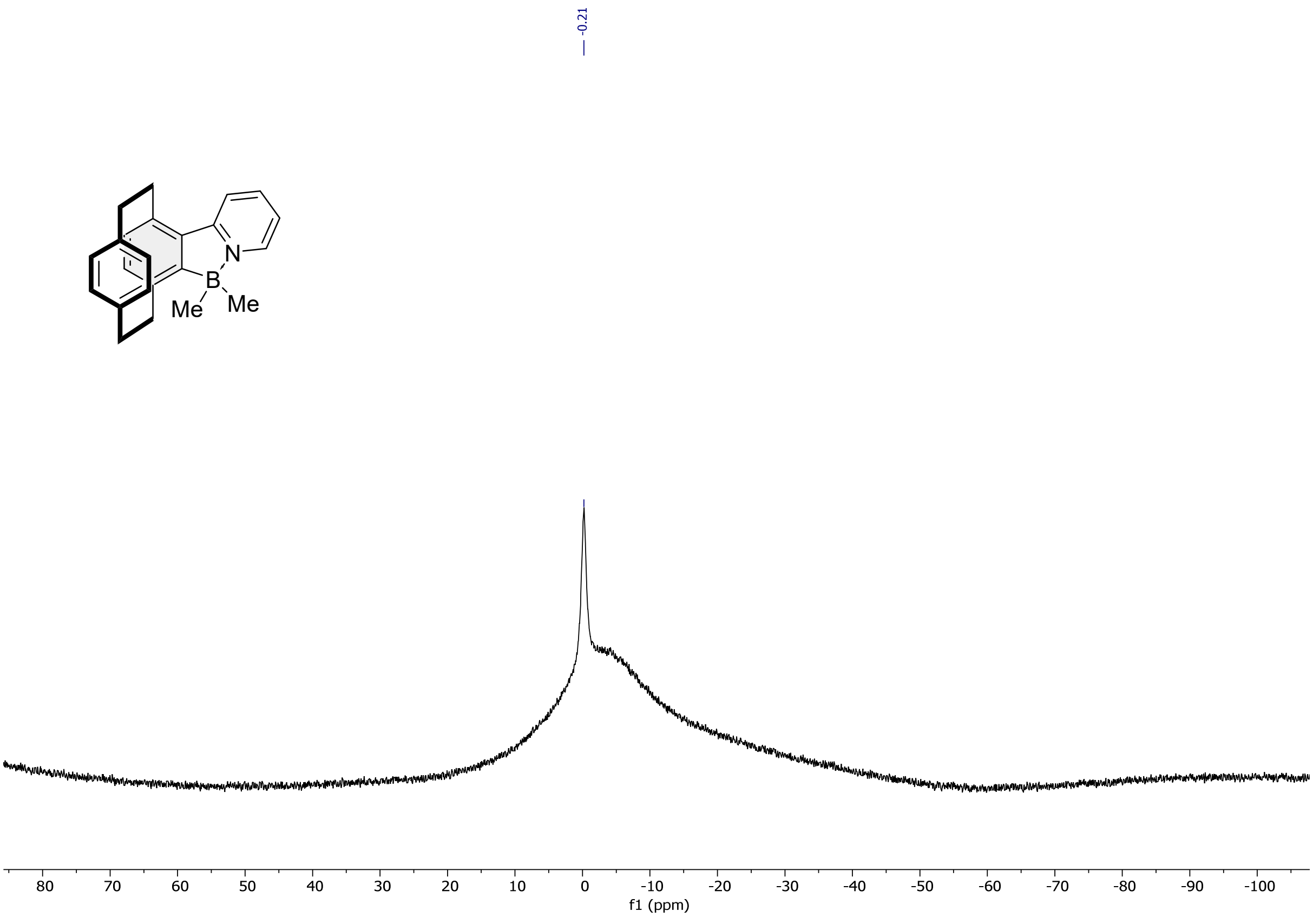

^{11}B NMR (128 MHz, $CDCl_3$)

rac-1^8,1^8-dimethyl-1^8H-1^{7}λ^4,1^{8}λ^4-1(9,12)-7λ^4-benzo[3,4][1,2]azaborolo[5,1-*a*]isoquinolina-4(1,4)-benzenacyclohexaphane (*rac*-1b)

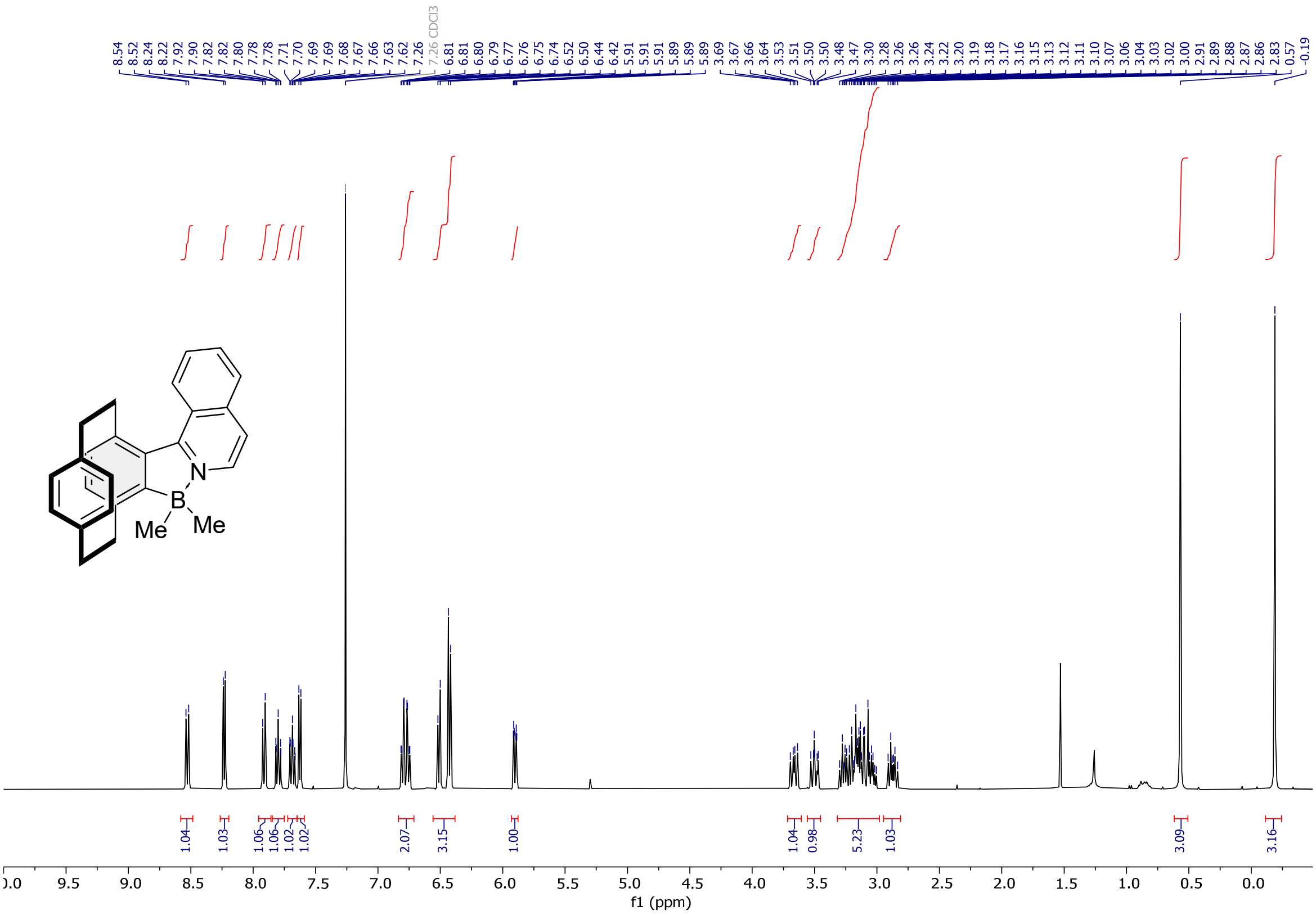

^{1}H NMR (400 MHz, $CDCl_3$)

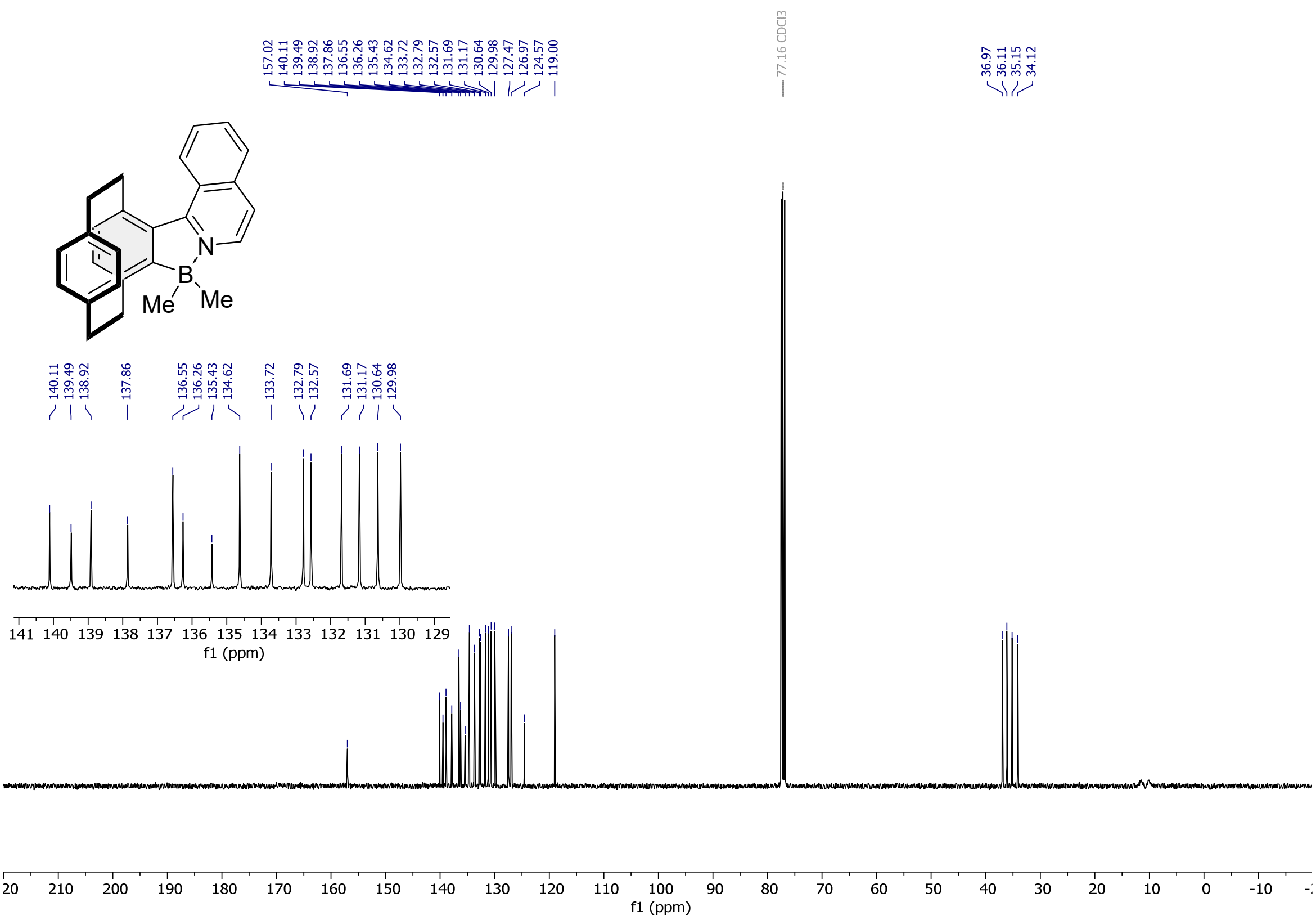

$^{13}C\{^1H\}$ NMR (101 MHz, $CDCl_3$)

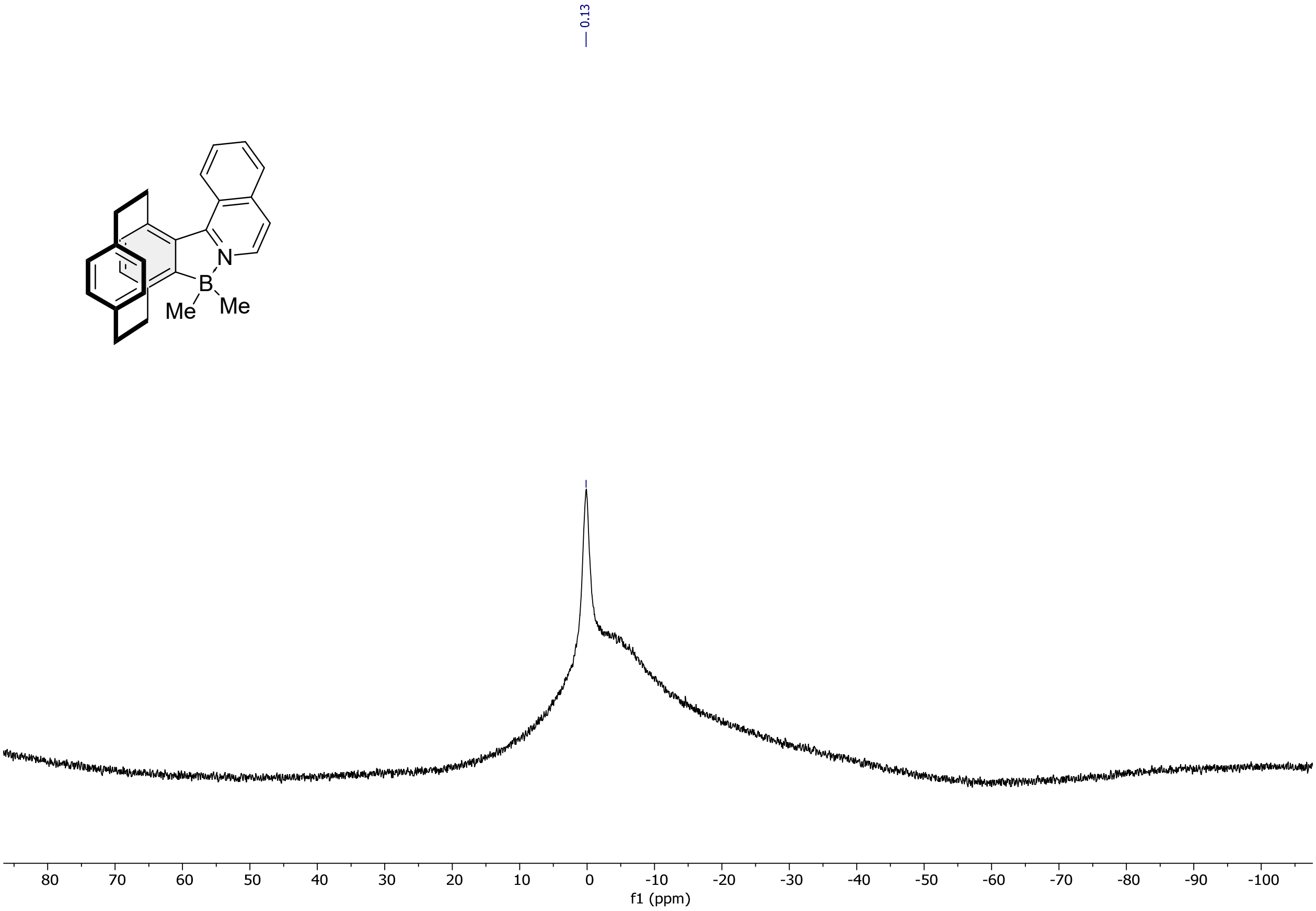

^{11}B NMR (128 MHz, $CDCl_3$)

rac-1¹⁰,1¹⁰-dimethyl-1¹⁰_H_-1⁹λ⁴,1¹⁰λ⁴-1(11,14)-9λ⁴-benzo[3,4][1,2]azaborolo[1,5-_f_]phenanthridina-4(1,4)-benzenacyclohexaphane (_rac_-1c)

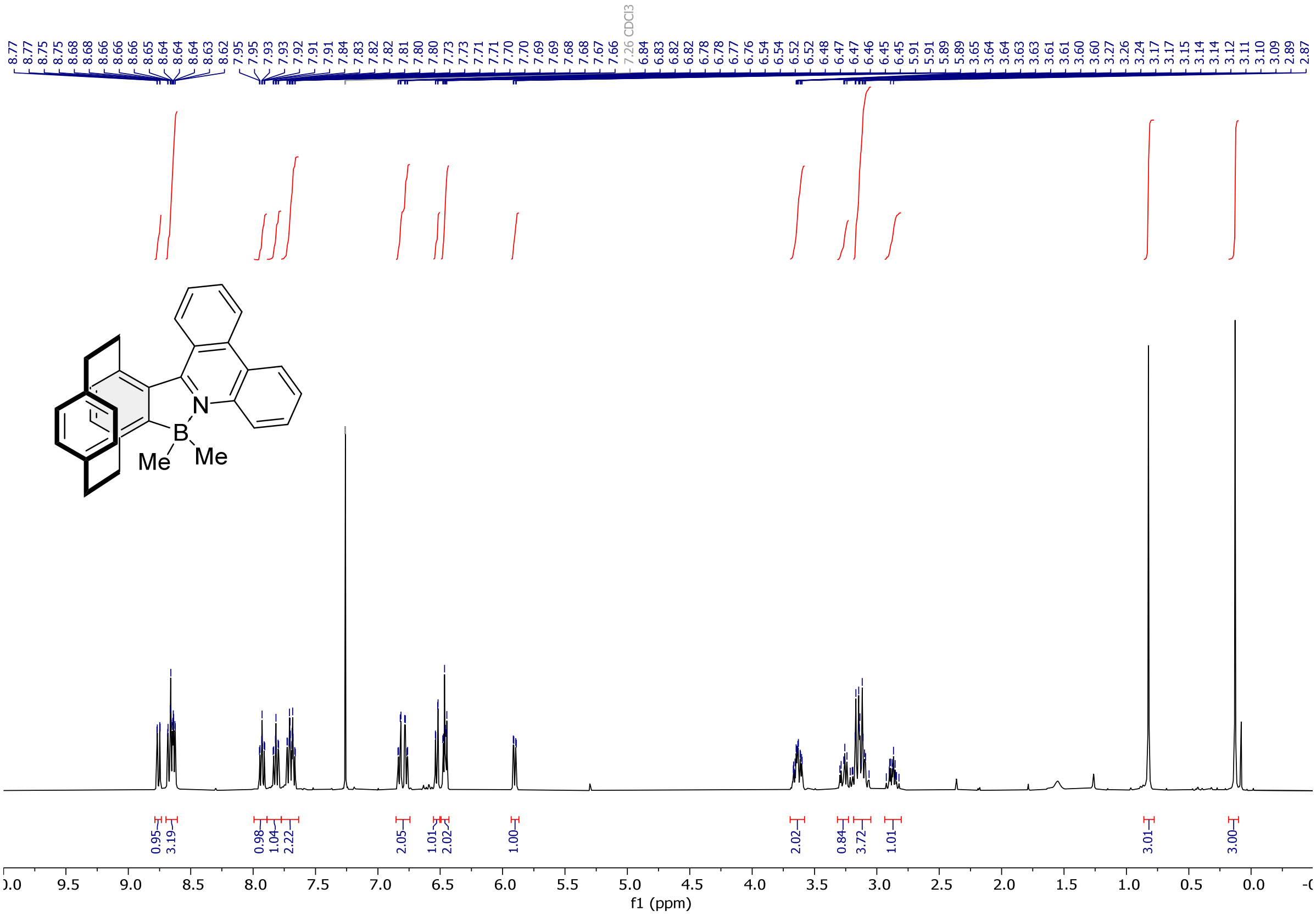

^{1}H NMR (400 MHz, $CDCl_3$)

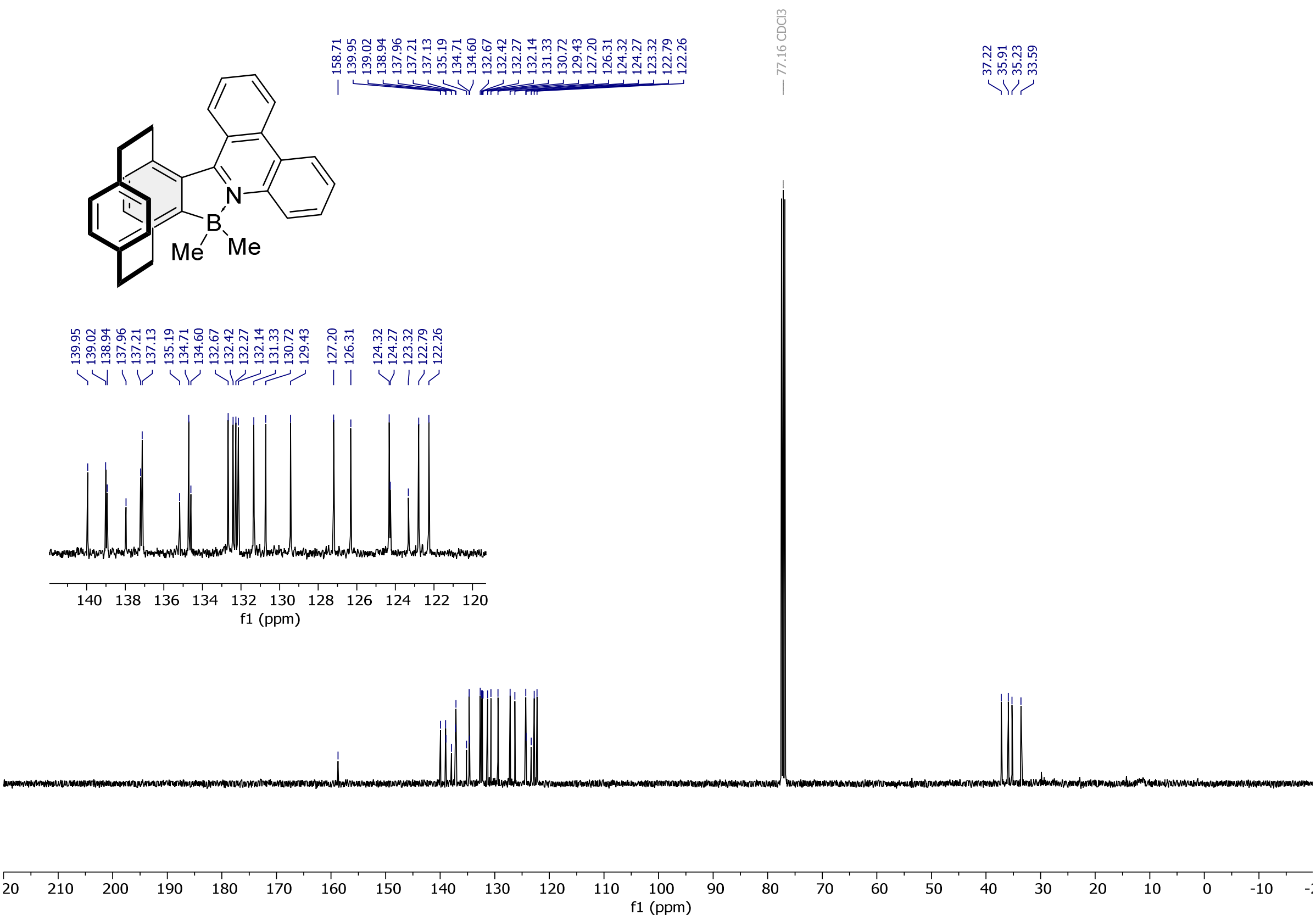

$^{13}C\{^{1}H\}$ NMR (101 MHz, $CDCl_3$)

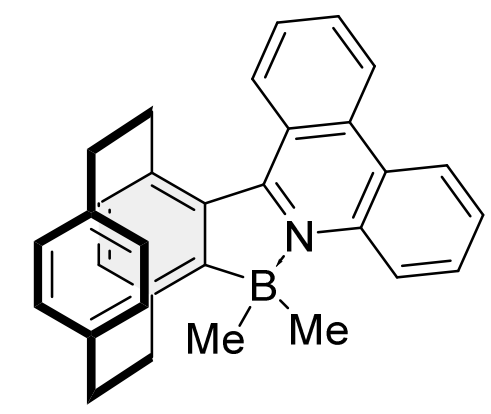

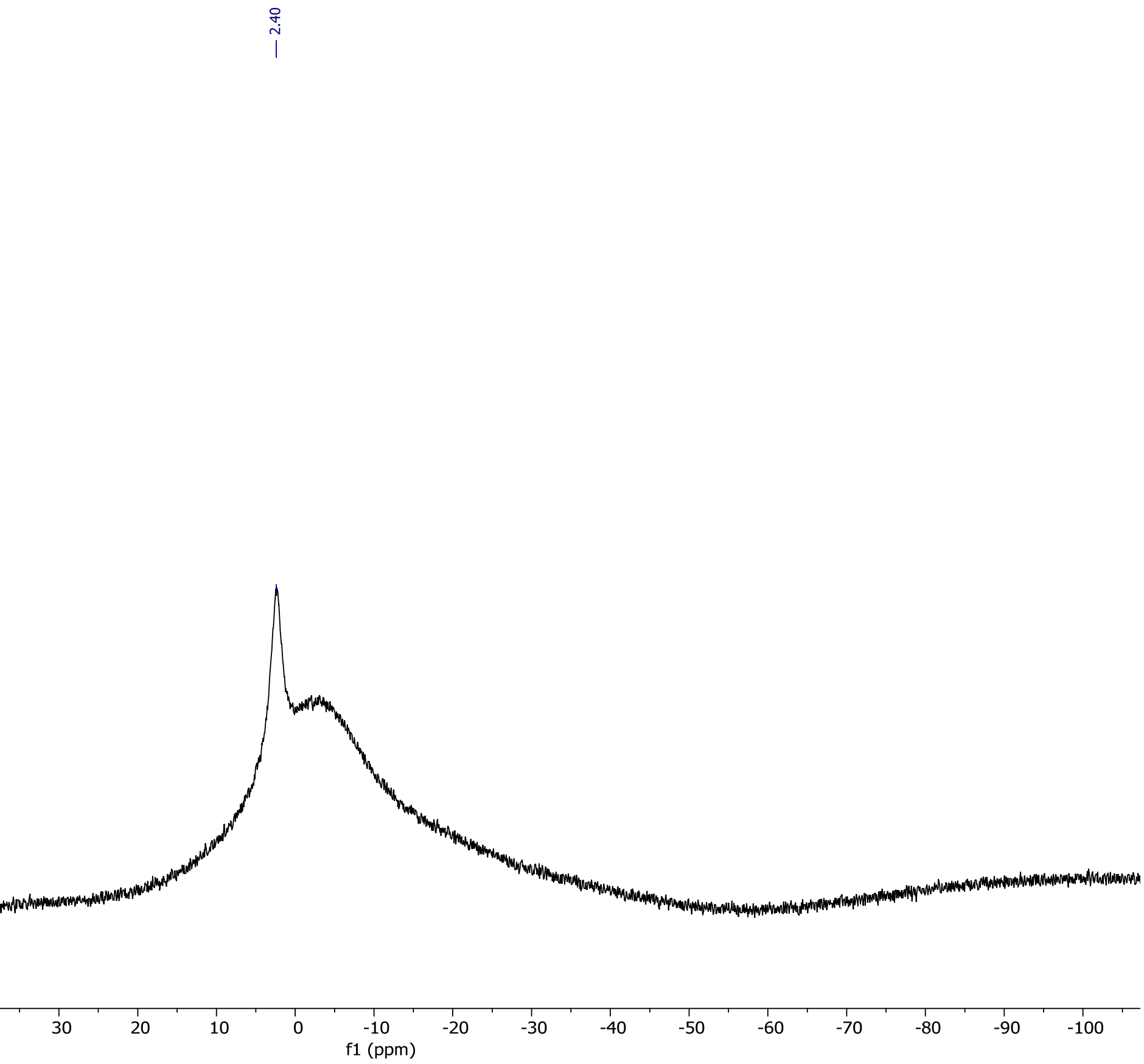

^{11}B NMR (128 MHz, $CDCl_3$)
